# Supplementary material for: Targeting USP10 induces degradation of oncogenic ANLN in esophageal squamous cell carcinoma
Source: Cell Death Differ. 2022 Dec 16;30(2):527–43. doi: 10.1038/s41418-022-01104-x (PMC9950447; doi:10.1038/s41418-022-01104-x)
Supplement: Supplementary file 1 — Supplementary Figures [file 41418_2022_1104_MOESM1_ESM.docx]

**Supplementary Figures**

**Targeting USP10 induces degradation of oncogenic ANLN in esophageal squamous cell carcinoma**

Yu-Fei Cao^1†^, Lei Xie^1†^, Bei-Bei Tong^1^, Man-Yu Chu^1,2^, Wen-Qi Shi^3^, Xiang Li^1,2^, Jian-Zhong He^5^, Shao-Hong Wang^3^, Zhi-Yong Wu^3^, Dan-Xia Deng^1,2^, Ya-Qi Zheng^1,2^, Zhi-Mao Li^1,2^, Xiu-E Xu^1,2^, Lian-Di Liao^1,2^, Yin-Wei Cheng^1,4^, Li-Yan Li^1,2^, Li-Yan Xu^1,2,4⁎^, En-Min Li^1⁎^

**Supplementary Figure 1:** **ANLN is highly expressed in ESCC tissues.**

**Supplementary Figure 2: Effect of USP10 or Cdh1 knockdown on ANLN expression.**

**Supplementary Figure 3: Effects of F806 on protein expression of ESCC cells.**

**Supplementary Figure 4: F806 inhibits the activity of USP10.**

**Supplementary Figure 5: Effects of F806 on ANLN expression and the cell cycle of ESCC.**

**Supplementary Figure 6: Regulatory function of USP10 in ESCC cells.**

**Supplementary Figure 7: Analysis of molecular functionality of ANLN and USP10 in ESCC tissues.**

Supplementary Figure 1


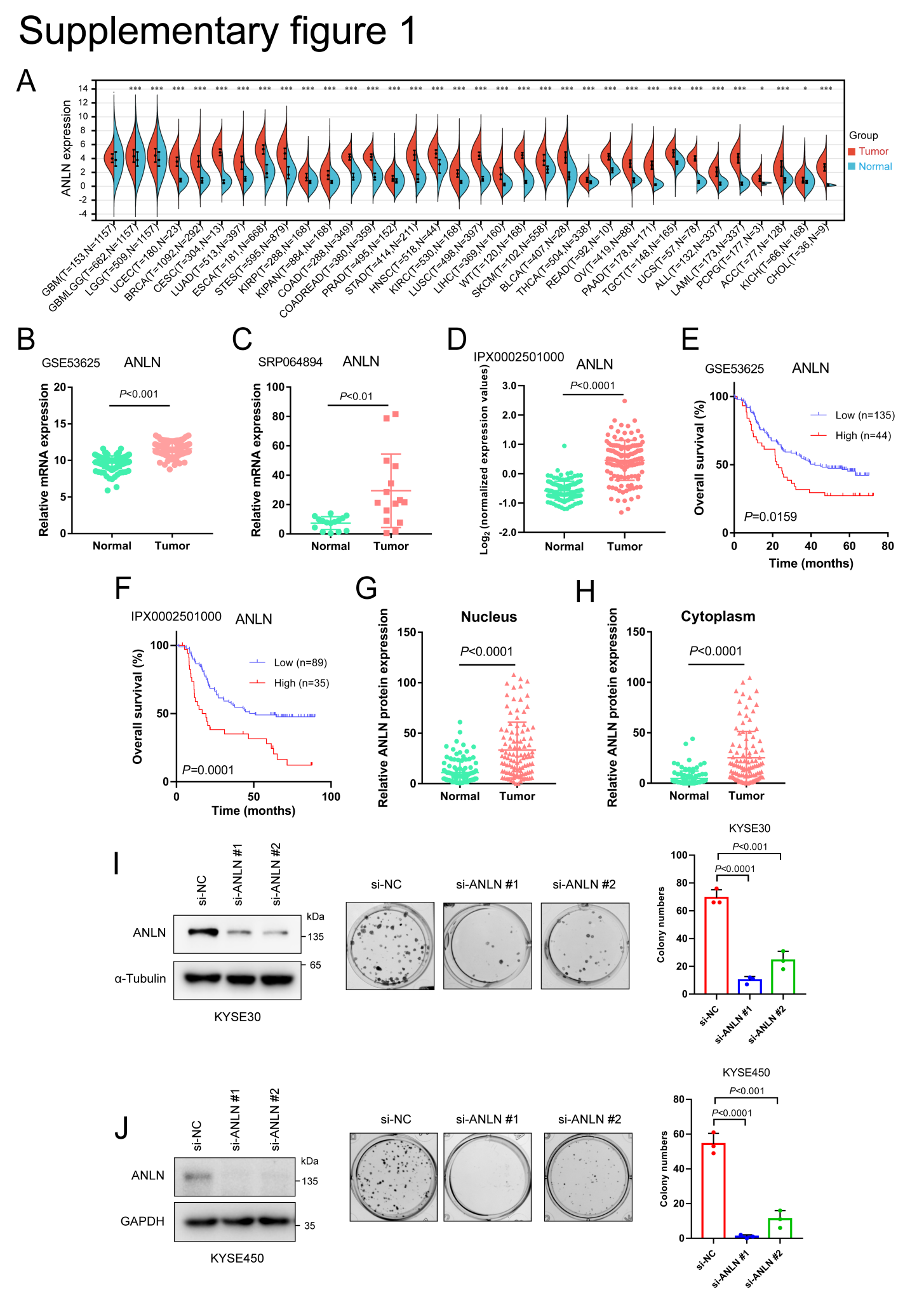
**Supplementary Figure 1:** **ANLN is highly expressed in ESCC tissues.** (A) TCGA, TARGET and GTEx (PANCAN, N=19131, G=60499) datasets were downloaded from the UCSC database (https://xenabrowser.net/). R software (version 3.6.4) was used to analyze the difference in ANLN expression between normal and tumor tissues. Unpaired Wilcoxon rank-sum and signed rank tests were used to analyze the significance of the differences (asterisks * and *** stand for *P*< 0.05 and *P*<0.001, respectively). (B) ANLN expression in ESCC tumors and normal tissues was analyzed based on the GSE53625 GeneChip data from the GEO database. (C) ANLN mRNA levels in ESCC tumors and normal tissues were analyzed based on the SRP064894 dataset from the SRA database. (D) ANLN protein levels in ESCC tissues from 124 patients and normal tissues were analyzed based on the IPX0002501000 dataset from the iProX database. (E) Kaplan-Meier curve analysis of the GEO data showed a relationship between ANLN mRNA expression and prognosis of ESCC patients. (F) Kaplan-Meier curve analysis of the correlation between ANLN protein expression and overall survival in 124 ESCC patients. (G) Nuclear ANLN expression in 104 ESCC tissues and normal tissues. (H) Cytosolic ANLN expression in 104 ESCC tissues and normal tissues. (I-J) Cells were transfected with control or ANLN siRNA for 48 h. ANLN levels were detected using western blotting. Effect of ANLN knockdown on cell proliferation was determined by colony formation assay.


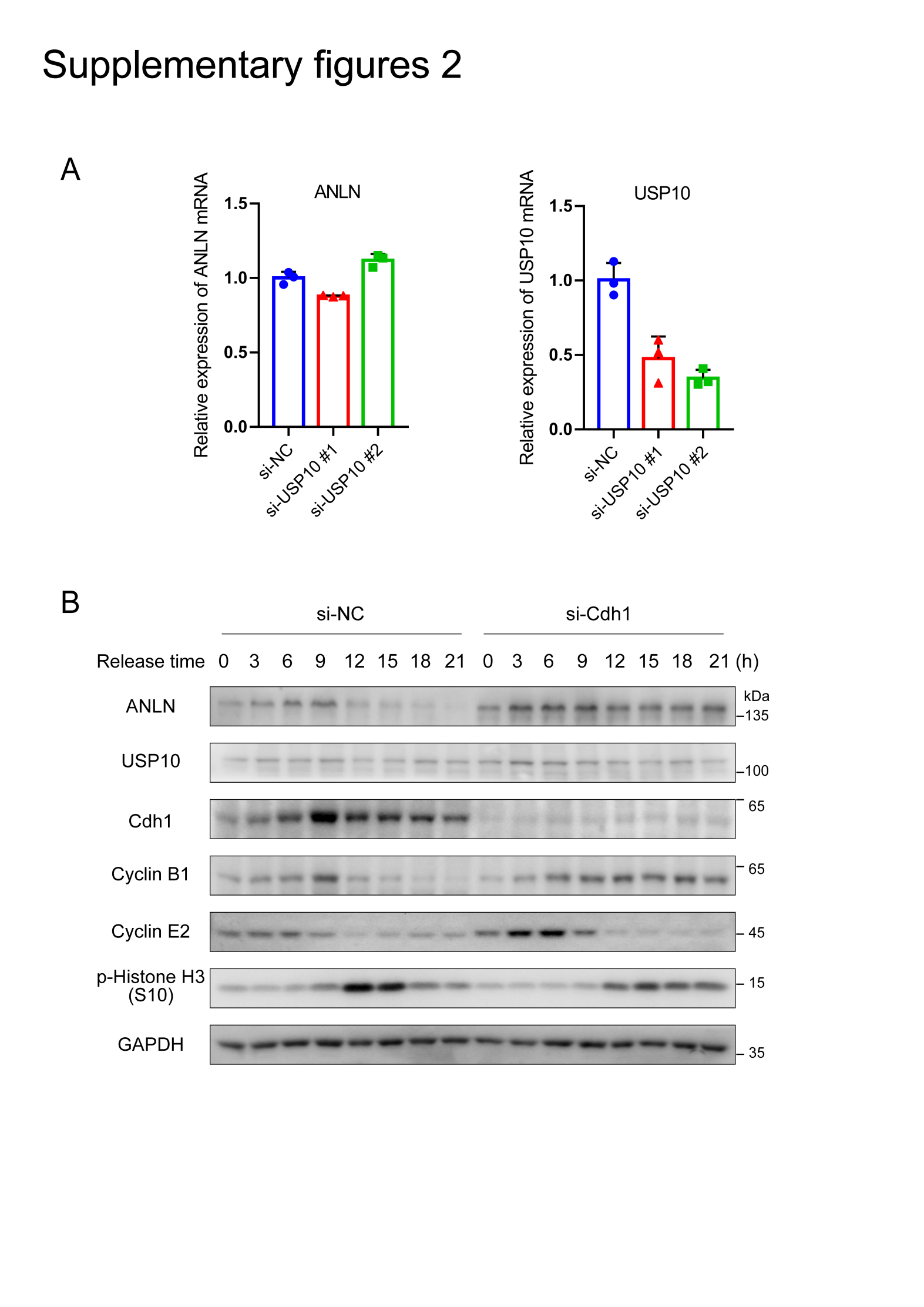
**Supplementary Figure 2: Effect of USP10 or Cdh1 knockdown on ANLN expression.** (A) KYSE150 cells were transfected with siRNAs for 48 hours, and then harvested with TRIzol (Life Technologies, 15596018) to extract total RNA. cDNA was obtained from the total RNA using HiScript III RT SuperMix (Vazyme, R323-01). Quantitative real-time PCR (qRT-PCR) was conducted with ChamQ Universal SYBR qPCR Master Mix (Vazyme, Q711-02). GAPDH was used for normalization. Primer sequences of ANLN (5’ to 3’): TGCACCATTGGCACAAACAG (forward primer); CCAGATTCAGCTCGAGGGAC (reverse primer). Primer sequences of

USP10 (5’ to 3’): TATGTGCGGGCGAGAAGATG (forward primer); TCAAGACGGGACAGAATGGC (reverse primer). (B) KYSE150 cells were transfected with siRNAs for 24 h, then double-thymidine blocked and released for different times. The levels of ANLN and Cdh1 were detected by western blotting. Cyclins B1 and E2 and phospho-histone H3 (S10) were used as markers for different cell cycle phases.


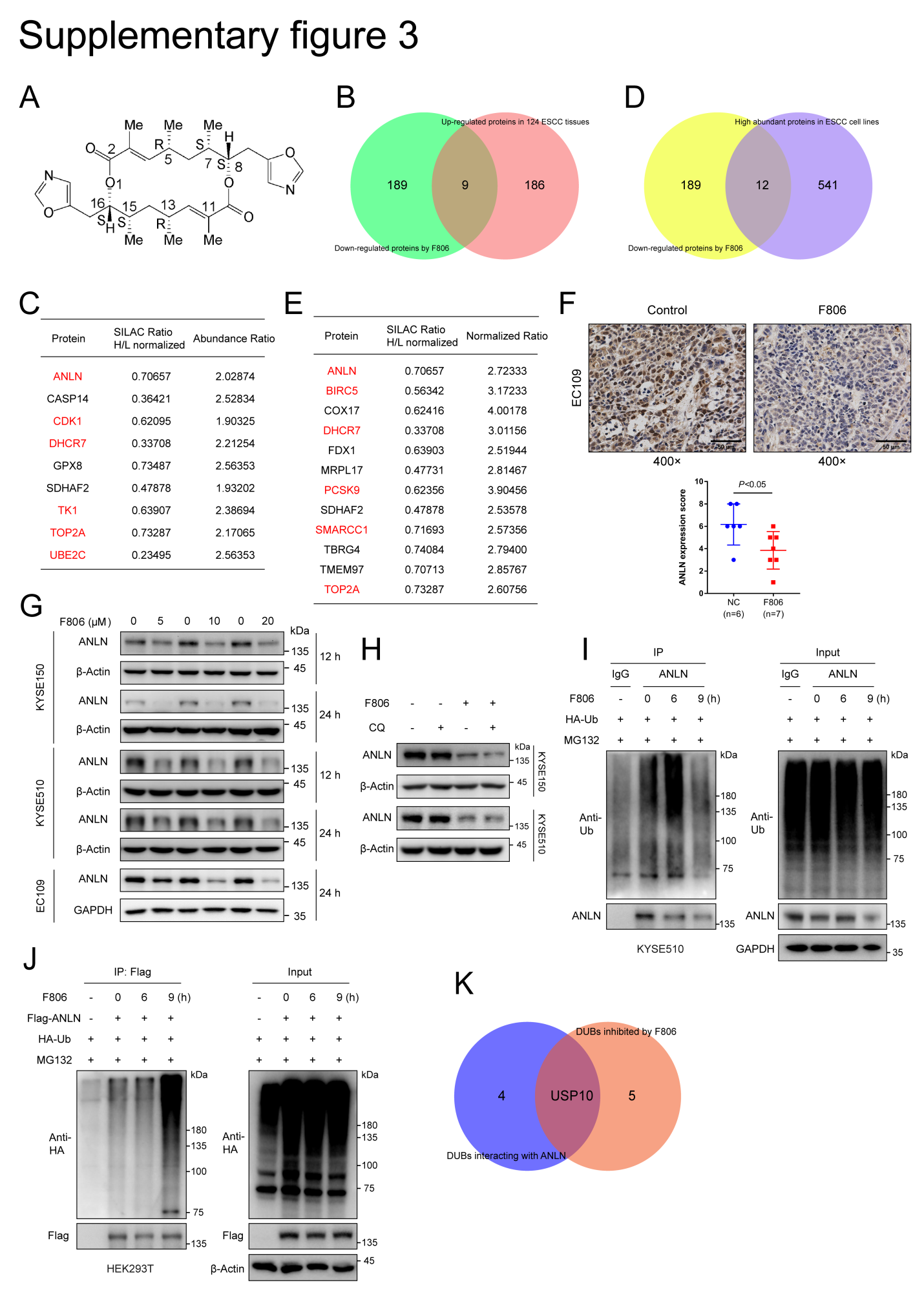
**Supplementary Figure 3: Effects of F806 on protein expression of ESCC cells.** (A) Chemical structure of F806**.** (B) Highly expressed proteins in 124 ESCC tissues and proteins significantly down-regulated by F806 were screened to analyze the effects of F806. (C) Proteins obtained by screening from (B), the proteins marked in red are related to mitosis. (D) Highly abundant proteins in 9 ESCC cell lines and proteins significantly down-regulated by F806 were screened to analyze the effects of F806. (E) Proteins obtained by screening from (D), the proteins marked in red are related to mitosis. (F) Effect of F806 on ANLN expression was evaluated in xenografts (EC109 cells) from tumor-bearing mice. After F806 (4 mg/kg) treatment for 21 days, the level of ANLN protein in xenografts was detected by immunohistochemistry. (G) Cells were treated with F806 for different times and with different doses, and ANLN protein levels were detected by western blotting. (H) Cells were co-treated with chloroquine (40 μM) and F806 (10 μM) for 21 h, and ANLN levels were detected by western blotting. (I) KYSE510 cells transfected with HA-Ub were treated with F806 (10 μM) for different times and then treated with MG132 (20 μM) for 8 h before harvest. Ubiquitination assay was performed to examine the ubiquitination level of ANLN. (J) HEK293T cells co-transfected with Flag-ANLN and HA-Ub were treated with F806 (10 μM) for different times and then treated with MG132 (20 μM) for 8 h before harvest. Ubiquitination assay was performed to examine the ubiquitination level of Flag-ANLN. (K) Screening of ANLN DUBs inhibited by F806 obtained by proteomics profiling.


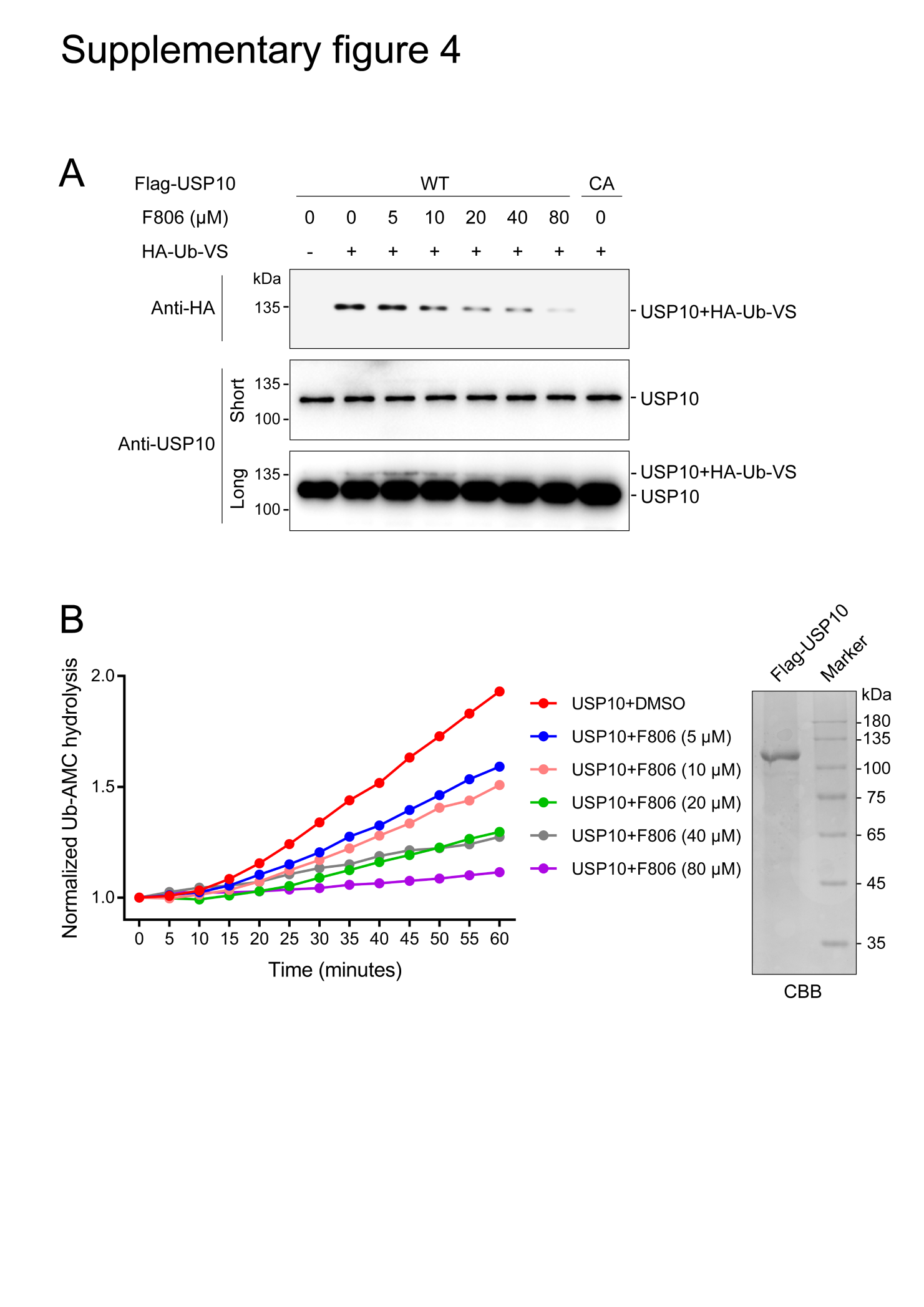
**Supplementary Figure 4: F806 inhibits the activity of USP10.** (A) USP10 protein purified from HEK293T cells was incubated with F806 at 25°C for 2 hours in vitro, and then labeled with HA-Ub-VS for 30 min. The indicated antibodies were used for the western blotting. (B) Flag-USP10 purified from HEK293T cells and different concentrations of F806 were mixed at 25°C for 2 h. Ub-AMC was then added to each well and further incubated at 37°C for 1 h. Ub-AMC hydrolysis was measured in real time.


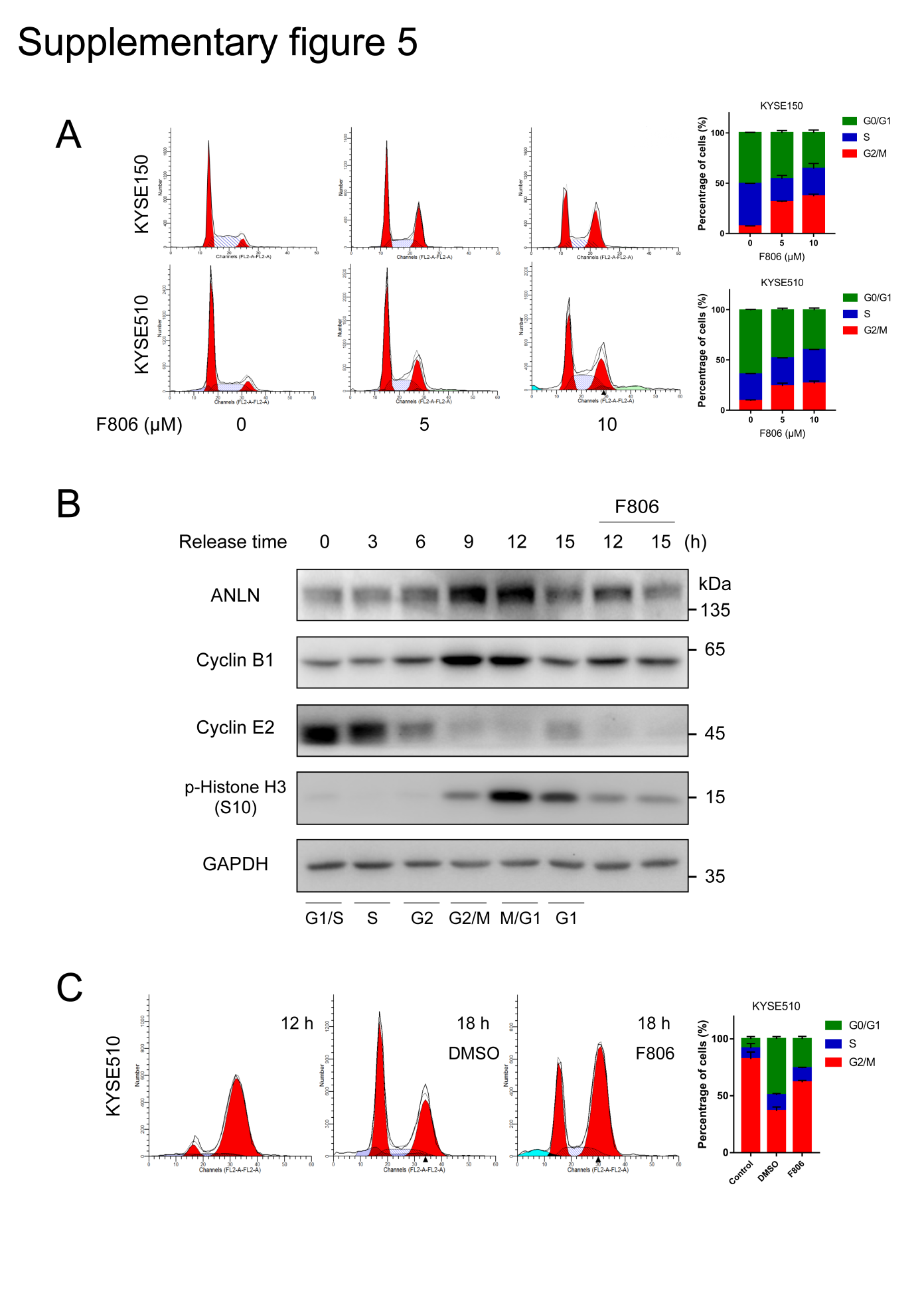
**Supplementary Figure 5: Effects of F806 on ANLN expression and the cell cycle of ESCC.** (A) ESCC cells were treated with different concentrations of F806 for 16 h, and then samples were analyzed by flow cytometry. (B) KYSE150 cells were synchronized by DTB and released for different time periods. F806 (10 μM) was added at 9 h after release. The samples were analyzed by western blotting. (C) KYSE510 cells were synchronized by double thymidine block, then released for 12 h into G2/M phase, treated with or without F806 (10 μM) for 6 h, and then examined by flow cytometry.


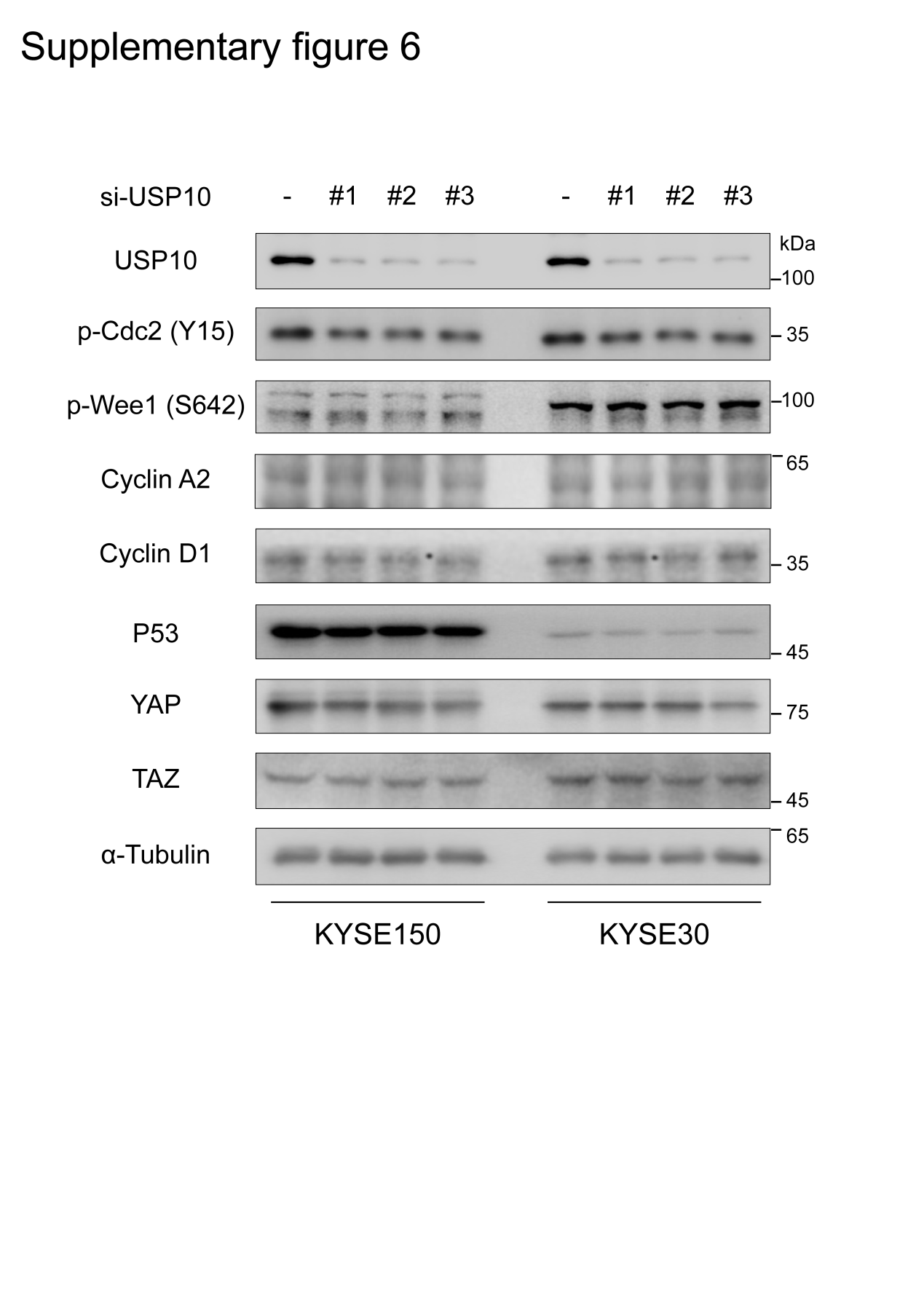
 **Supplementary Figure 6: Regulatory function of USP10 in ESCC cells.** KYSE150 and KYSE30 cells were transfected with siRNAs for 48 h. The indicated antibodies were used for the western blotting.


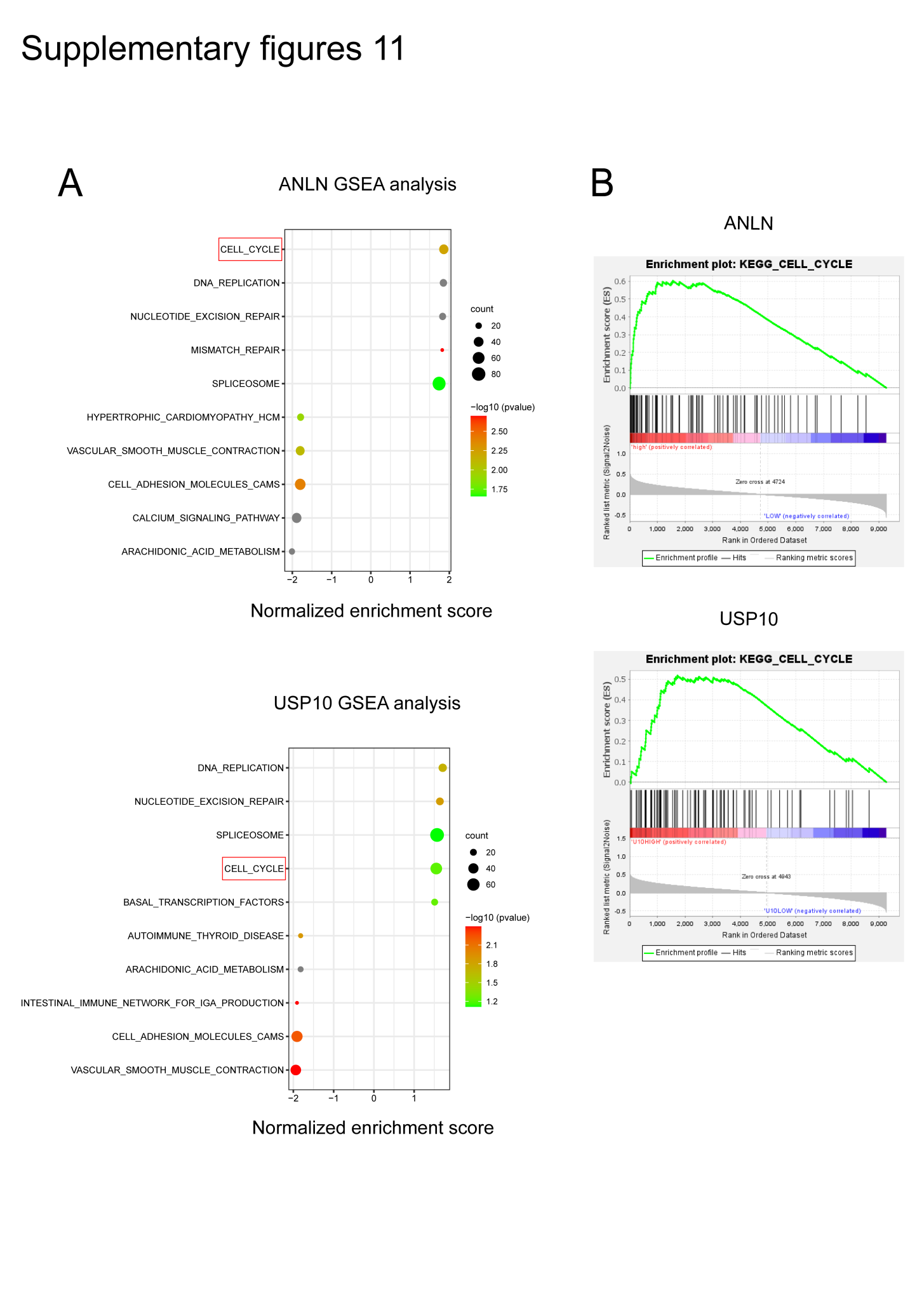
**Supplementary Figure 7: Analysis of molecular functionality of ANLN and USP10 in ESCC tissues.** (A) Potential functions of ANLN and USP10 in 124 ESCC tissues were analyzed by Gene Set Enrichment Analysis (GSEA). (B) Expression levels of ANLN and USP10 were positively correlated with cell cycle regulation.
