# Supplementary material for: Targeting USP10 induces degradation of oncogenic ANLN in esophageal squamous cell carcinoma
Source: Cell Death Differ. 2022 Dec 16;30(2):527–43. doi: 10.1038/s41418-022-01104-x (PMC9950447; doi:10.1038/s41418-022-01104-x)
Supplement: Supplementary file 2 — Supplementary Tables [file 41418_2022_1104_MOESM2_ESM.docx]

**Supplementary Table 1. Full names of tumors in Figure S1A**

| Abbreviations | Full names |
| --- | --- |
| GBM | Glioblastoma multiforme |
| GBMLGG | Glioma |
| LGG | Brain Lower Grade Glioma |
| UCEC | Uterine Corpus Endometrial Carcinoma |
| BRCA | Breast invasive carcinoma |
| CESC | Cervical squamous cell carcinoma and endocervical adenocarcinoma |
| LUAD | Lung adenocarcinoma |
| ESCA | Esophageal carcinoma |
| STES | Stomach and Esophageal carcinoma |
| KIRP | Kidney renal papillary cell carcinoma |
| KIPAN | Pan-kidney cohort (KICH+KIRC+KIRP) |
| COAD | Colon adenocarcinoma |
| COADREAD | Colon adenocarcinoma/Rectum adenocarcinoma Esophageal carcinoma |
| PRAD | Prostate adenocarcinoma |
| STAD | Stomach adenocarcinoma |
| HNSC | Head and Neck squamous cell carcinoma |
| KIRC | Kidney renal clear cell carcinoma |
| LUSC | Lung squamous cell carcinoma |
| LIHC | Liver hepatocellular carcinoma |
| WT | High-risk Wilms tumor |
| SKCM | Skin cutaneous melanoma |
| BLCA | Bladder urothelial carcinoma |
| THCA | Thyroid carcinoma |
| READ | Rectum adenocarcinoma |
| OV | Ovarian serous cystadenocarcinoma |
| PAAD | Pancreatic adenocarcinoma |
| TGCT | Testicular germ cell tumors |
| UCS | Uterine carcinosarcoma |
| ALL | Acute lymphoblastic leukemia |
| LAML | Acute myeloid leukemia |
| PCPG | Pheochromocytoma and paraganglioma |
| ACC | Adrenocortical carcinoma |
| KICH | Kidney chromophobe |
| CHOL | Cholangiocarcinoma |

| **Supplementary Table 2. Potential deubiquitinating enzymes of ANLN screened by protein-protein interaction profiling** | | | |  |
| --- | --- | --- | --- | --- |
| Gene Symbol | | Sum PEP Score | Type |  |
| USP10  USP7  USP39  USP16 | | 11.21  7.4  4.158  3.744 | | DUB  DUB  DUB  DUB |
|  |  | |  |  |

| **Supplementary Table 3. F806-inhibited deubiquitinating enzymes screened by protein profiling** | | | |  |  |
| --- | --- | --- | --- | --- | --- |
| Gene Symbol | | H/L Ratio normalized | Type |  |  |
| USP10  USP24  USP9X  USP39  USP5 | | 0.73915  0.78744  0.9295  0.95299  0.96816 | | DUB  DUB  DUB  DUB  DUB |  |
|  |  | |  |  |  |

| **Supplementary Table 4. Clinicopathological characteristics of patients with ESCC** | | | | | | | |
| --- | --- | --- | --- | --- | --- | --- | --- |
| **Clinical and pathological indexes** | **Case No.** | 5-year OS (%) | *P** |  | 5-year DFS (%) | *P** |  |
| Specimens | 104 |  |  |  |  |  |  |
| Mean age | 58.53 |  |  |  |  |  |  |
| Age (year) |  |  |  |  |  |  |  |
| <59 | 57 | 50.4 | 0.050 |  | 47.2 | 0.059 |  |
| ≥59 | 47 | 25.2 |  |  | 25.5 |  |  |
| Gender |  |  |  |  |  |  |  |
| Male | 82 | 39.8 | 0.860 |  | 36.0 | 0.747 |  |
| Female | 22 | 38.4 |  |  | 40.9 |  |  |
| Tumor size |  |  |  |  |  |  |  |
| ≤3cm | 23 | 46.4 | 0.393 |  | 47.8 | 0.476 |  |
| 3-5cm | 50 | 41.7 |  |  | 37.6 |  |  |
| >5cm | 31 |  |  |  | 29.0 |  |  |
| Tumor location |  |  |  |  |  |  |  |
| upper | 6 | 16.7 | 0.084 |  | 0.0 | 0.052 |  |
| middle | 45 | 46.1 |  |  | 44.1 |  |  |
| lower | 53 | 35.1 |  |  | 34.3 |  |  |
| Histologic grade |  |  |  |  |  |  |  |
| G1 | 9 | 55.6 | 0.099 |  | 55.6 | 0.014 |  |
| G2 | 88 | 39.1 |  |  | 37.0 |  |  |
| G3 | 7 | 14.3 |  |  | 14.3 |  |  |
| Invasive depth |  |  |  |  |  |  |  |
| T1 | 6 | 50.0 | 0.665 |  | 60.0 | 0.396 |  |
| T2 | 16 | 37.5 |  |  | 37.5 |  |  |
| T3 | 82 | 36.9 |  |  | 32.6 |  |  |
|  |  |  |  |  |  |  |  |
| Lymph node metastasis | |  |  |  |  |  |  |
| N0 | 53 | 46.2 | 0.002 |  | 44.7 | 0.058 |  |
| N1 | 29 | 40.6 |  |  | 35.7 |  |  |
| N2 | 18 | 16.7 |  |  | 18.1 |  |  |
| N3 | 4 | 0.0 |  |  | 0.0 |  |  |
| pTNM-stage |  |  |  |  |  |  |  |
| I | 3 | 66.7 | 0.010 |  | 66.7 | 0.060 |  |
| II | 51 | 46.1 |  |  | 44.5 |  |  |
| III | 46 | 32.0 |  |  | 29.6 |  |  |
| Ⅳ | 4 | 0.0 |  |  | 0.0 |  |  |
| * Kaplan-Meier log-rank test; *P* <0.05 was considered to indicate significance. | | | | | | | |
| All patients underwent surgical treatment. | | | | | | | |
| OS: overall survival | | | | | | | |
| DFS: disease-free survival | | | | | | | |

| **Supplementary Table 5. Small interfering RNA (siRNA) sequences.** | | |  |  |
| --- | --- | --- | --- | --- |
| siRNAs | Sense Sequences (5'-3') | Antisense Sequences (5'-3') |  |  |
| siANLN #1  siANLN #2  siANLN #3  siCdh1 #1  siCdh1 #2  siUSP10 #1  siUSP10 #2  siUSP10 #3  siANLN-3’UTR #1  siANLN-3’UTR #2  siANLN-3’UTR #3  siUSP10-3’UTR | | GCUACAUUCUGUUCCCAAATT  CCAGACCUCUGCUUUCAAATT  GCAGAUACCAUCAGUGAUUTT  GCAACGAUGUGUCUCCCUATT  CCCGUUCGACAAAGGUAAATT  GCUUUGGAUGGAAGUUCUATT  GCACACCACGGAAAGCAUATT  CCUUUGAGCCCACAUAUAUTT  GCCAAUAUUCACUACGUAUTT  UCGAAAGGCUGUGAUCAUUTT  GCUUAAAGCAUGAGGCUGUTT  GCUUAGUAGAAUAAAUCCUTT | UUUGGGAACAGAAUGUAGCTT  UUUGAAAGCAGAGGUCUGGTT  AAUCACUGAUGGUAUCUGCTT  UAGGGAGACACAUCGUUGCTT  UUUACCUUUGUCGAACGGGTT  UAGAACUUCCAUCCAAAGCTT  UAUGCUUUCCGUGGUGUGCTT  AUAUAUGUGGGCUCAAAGGTT  AUACGUAGUGAAUAUUGGCTT  AAUGAUCACAGCCUUUCGATT  ACAGCCUCAUGCUUUAAGCTT  AGGAUUUAUUCUACUAAGCTT |  |
| Note: ANLN 3'UTR siRNA pool = siANLN-3’UTR #1+ siANLN-3’UTR #2+ siANLN-3’UTR #3  USP10 siRNA pool = siUSP10 #1+ siUSP10 #2+ siUSP10 #3 |  |  |  |  |

| plasmids | Forward primer (5'-3') | Reverse primer (5'-3') |
| --- | --- | --- |
| pBOBI-C-3×HA-ANLN | AGAGAATTCGGATCCGCCACCATGGATCCGTTTACGGAGAAACTGC | CTTCCATGGCTCGAGAGGCTTTCCAATAGGTTTGTAGCAAGC |
| pBOBI-C-3×HA-ANLN-1-712 | AGAGAATTCGGATCCGCCACCATGGATCCGTTTACGGAGAAACTGC | CTTCCATGGCTCGAGACAAGGAAAGGCATTATTTTTTTCATCCAGTTTTG |
| pBOBI-C-3×HA-ANLN-1-801 | AGAGAATTCGGATCCGCCACCATGGATCCGTTTACGGAGAAACTGC | CTTCCATGGCTCGAGTTTGGATGGCATAAATTCACTTTGGGGA |
| pBOBI-C-3×HA-ANLN-1-986 | AGAGAATTCGGATCCGCCACCATGGATCCGTTTACGGAGAAACTGC | CTTCCATGGCTCGAGTTCTTCAACACTGGAATTCACTTGACATTTTATTTTTAAATAAATATG |
| pBOBI-C-3×HA-ANLN-712-1124 | AGAGAATTCGGATCCGCCACCATGTGTCAAGTTAATATCAAACAGAAAATGCAGGA | CTTCCATGGCTCGAGAGGCTTTCCAATAGGTTTGTAGCAAGC |
| PCMV-N-Flag-USP10 | TGGAGGCCCGAATTCCGATGCCCTGGTTGCCCTCTC | CCGCGGTACCTCGAGTTACAGCAGGTCCACTCGGC |
| PCMV-N-HA-USP10 | TGGAGGCCCGAATTCCGATGCCCTGGTTGCCCTCTC | CCGCGGTACCTCGAGTTACAGCAGGTCCACTCGGC |
| pCMV-USP10 | CCCAGGCCCGAATTCATGCCCTGGTTGCCCTCTC | AGCCGGTACCTCGAGTTACAGCAGGTCCACTCGGC |
| pCMV-USP10-CA | GAACTGGGCCTACATTAATGCTACACTGCAGGCA | TAATGTAGGCCCAGTTCCCTTTATTGATCAGCC |
| pCMV-N-HA-USP10-CA | GAACTGGGCCTACATTAATGCTACACTGCAGGCA | TAATGTAGGCCCAGTTCCCTTTATTGATCAGCC |
| pBOBI-C-3×HA-Cdh1 | AGAGAATTCGGATCCATGGACCAGGACTATGAGCGG | CTTCCATGGCTCGAGCCGGATCCTGGTGAAGAGGTTG |
| pBOBI-N-MYC-Cdh1  PCMV-N-HA-USP10-104-802  PCMV-N-HA-USP10-210-802  PCMV-N-HA-USP10-403-802  PCMV-N-HA-USP10-1-598  pGEX-6P-1-USP10 (GST-USP10)  pET-32a-USP10 (His-USP10)  pET-32a-ANLN (His-ANLN)  pET-32a-ANLN-1-454  pET-32a-ANLN-454-1124 | AGAGAATTCGGATCCATGGACCAGGACTATGAGCGGC  TGGAGGCCCGAATTCCGATGACCCCTGATGGTATCACTAAAGAAG  TGGAGGCCCGAATTCCGATGCCCAGGACTTGTAACAGCC  TGGAGGCCCGAATTCCGATGTTGCTGGAGAATGTAACCCTAATCC  TGGAGGCCCGAATTCCGATGCCCTGGTTGCCCTCTC  GGGCCCCTGGGATCCATGCCCTGGTTGCCCTCTC  GCTGATATCGGATCCATGCCCTGGTTGCCCTCTC  GCTGATATCGGATCCATGGATCCGTTTACGGAGAAACTGC  GCTGATATCGGATCCATGGATCCGTTTACGGAGAAACTGC  AAGGCCATGGCTGATATCGGATCCGCCACCATGGGAAACTCAAAAAGCAAACAACTAGAAAC | CTTCCATGGCTCGAGTTACCGGATCCTGGTGAAGAGGT  CCGCGGTACCTCGAGTTACAGCAGGTCCACTCGGC  CCGCGGTACCTCGAGTTACAGCAGGTCCACTCGGC  CCGCGGTACCTCGAGTTACAGCAGGTCCACTCGGC  CCGCGGTACCTCGAGTTACTGGCGGGTGACGGAAG  ATGCGGCCGCTCGAGTTACAGCAGGTCCACTCGGC  GTGGTGGTGCTCGAGTTACAGCAGGTCCACTCGGC  GTGGTGGTGCTCGAGTTAAGGCTTTCCAATAGGTTTGTAGCAAGC  GTGGTGGTGGTGGTGGTGCTCGAGTCCGCCTTTTTCTGCACTCCA  GTGGTGGTGCTCGAGTTAAGGCTTTCCAATAGGTTTGTAGCAAGC |

**Supplementary Table 6. Primer sequences for plasmid construction**
