# Supplementary figures and images for: Targeting USP10 induces degradation of oncogenic ANLN in esophageal squamous cell carcinoma
Source: Cell Death Differ. 2022 Dec 16;30(2):527–43. doi: 10.1038/s41418-022-01104-x (PMC9950447; doi:10.1038/s41418-022-01104-x)

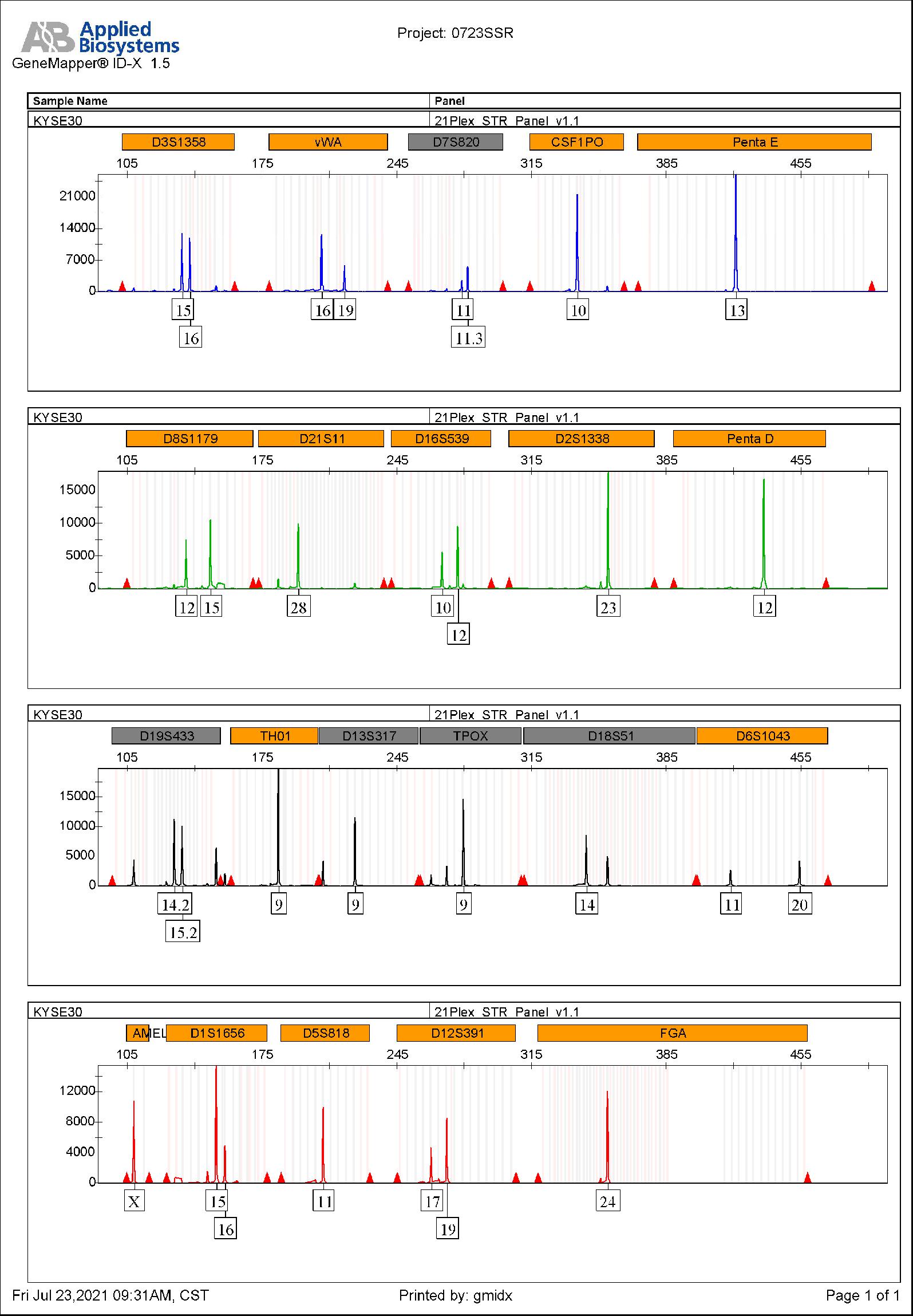


KYSE30 cell line


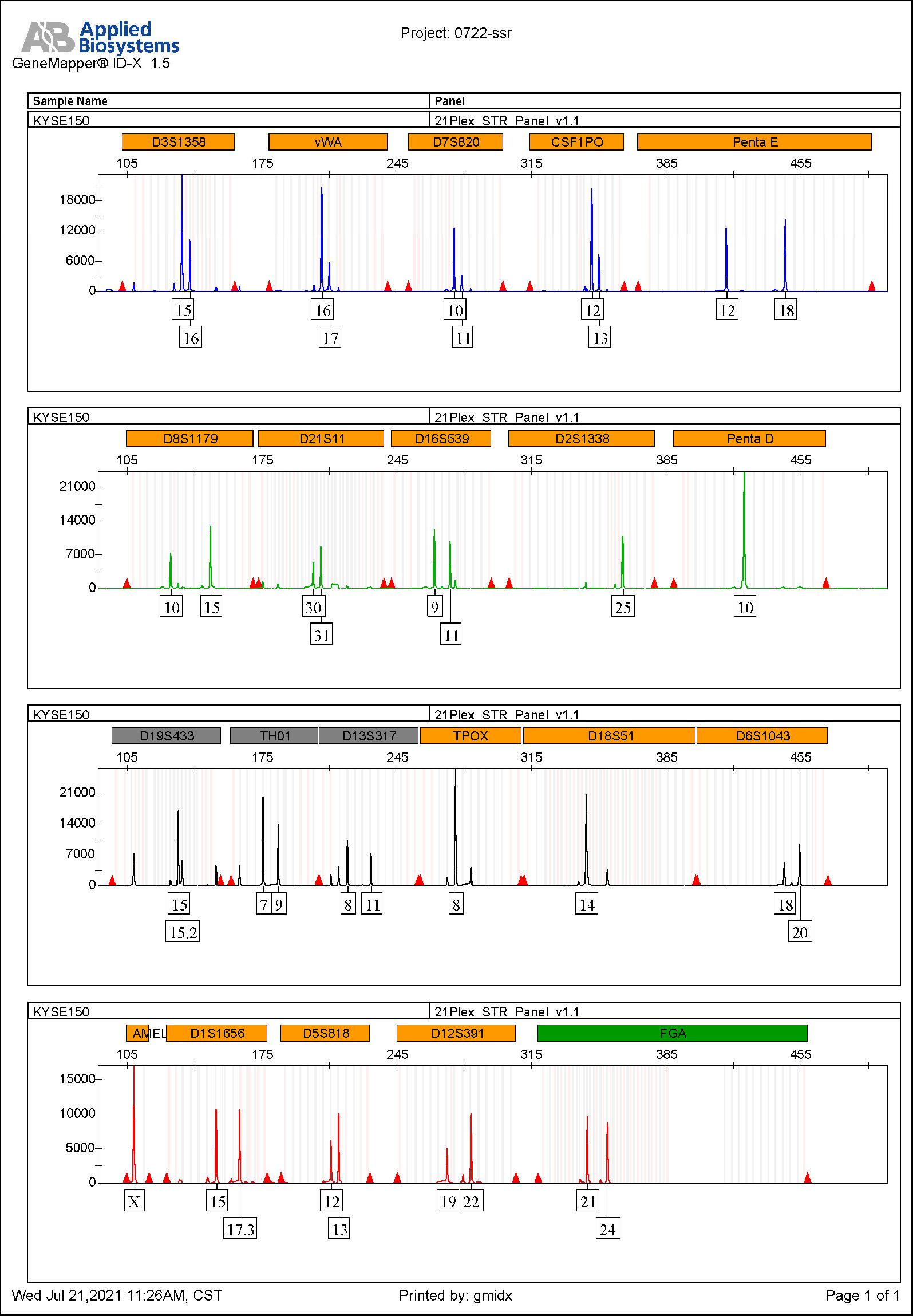


KYSE150 cell line


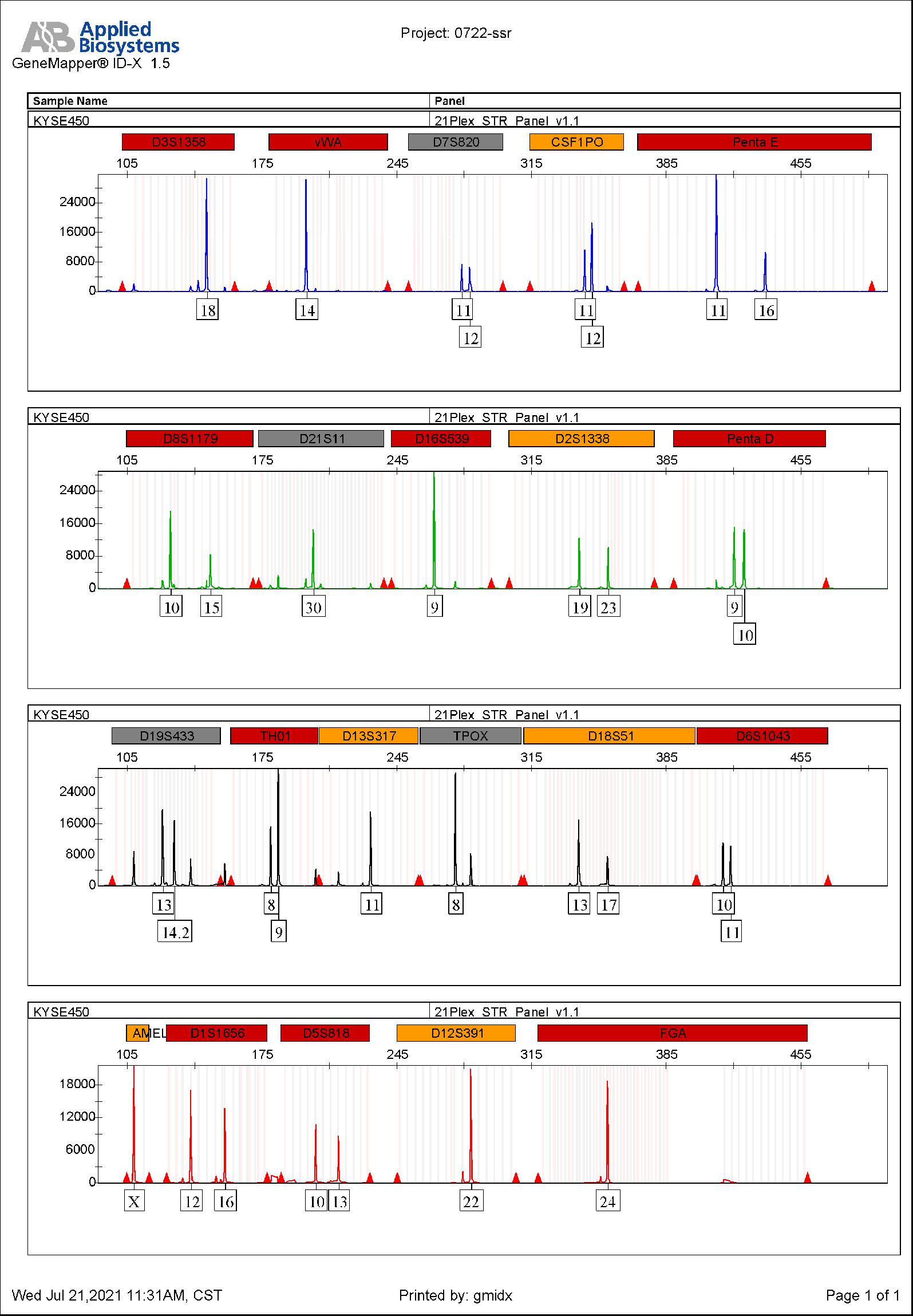


KYSE450 cell line


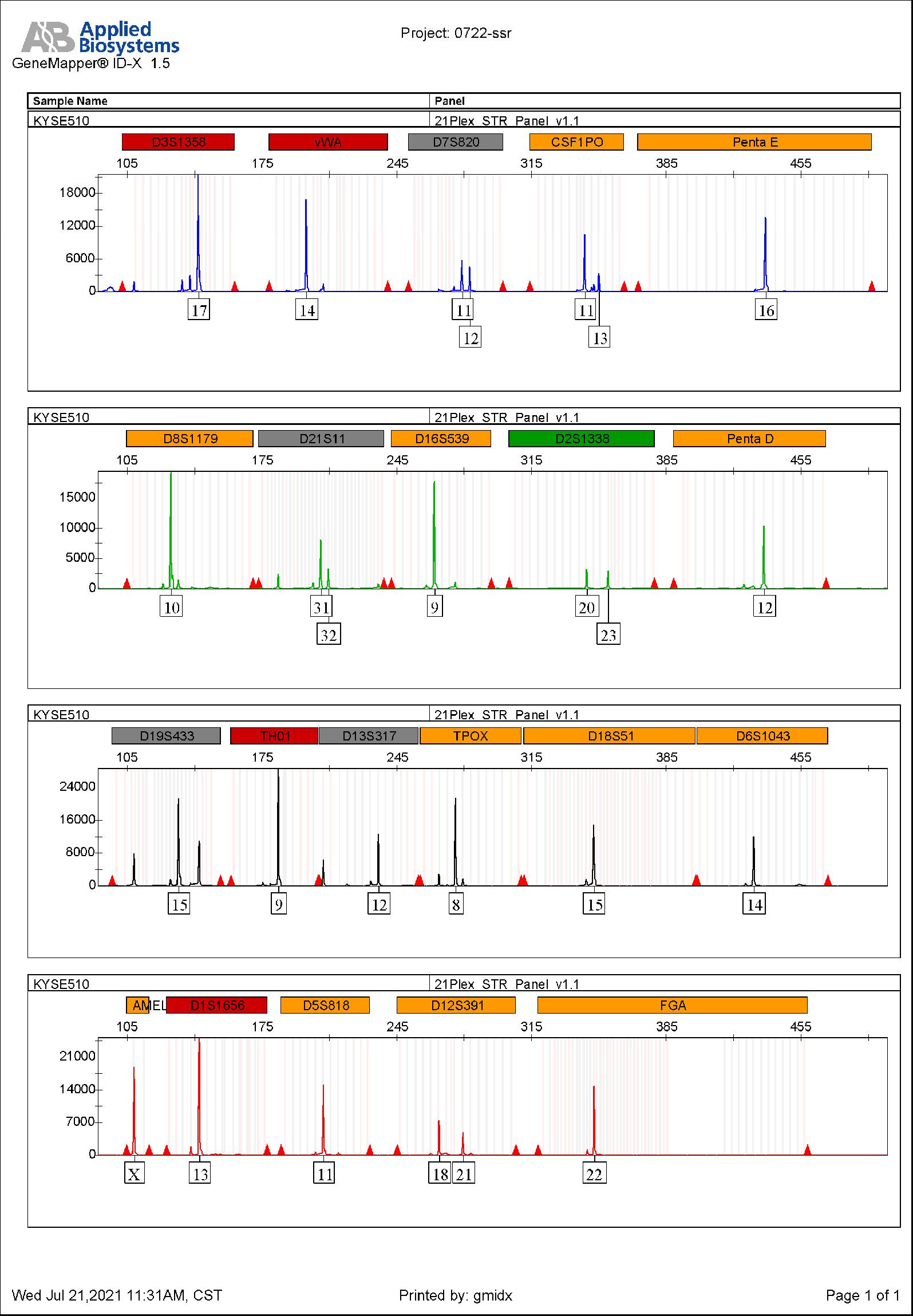


KYSE510 cell line

Supplement: Supplementary file 7 — Supplementary Data 5 [file 41418_2022_1104_MOESM7_ESM.docx]
