# Supplementary material for: Targeting USP10 induces degradation of oncogenic ANLN in esophageal squamous cell carcinoma
Source: Cell Death Differ. 2022 Dec 16;30(2):527–43. doi: 10.1038/s41418-022-01104-x (PMC9950447; doi:10.1038/s41418-022-01104-x)
Supplement: Supplementary file 9 — Manuscript-Marke up version [file 41418_2022_1104_MOESM9_ESM.docx]

**Targeting USP10 induces degradation of oncogenic ANLN in esophageal squamous cell carcinoma**

Yu-Fei Cao^1†^, Lei Xie^1†^, Bei-Bei Tong^1^, Man-Yu Chu^1,2^, Wen-Qi Shi^3^, Xiang Li^1,2^, Jian-Zhong He^5^, Shao-Hong Wang^3^, Zhi-Yong Wu^3^, Dan-Xia Deng^1,2^, Ya-Qi Zheng^1,2^, Zhi-Mao Li^1,2^, Xiu-E Xu^1,2^, Lian-Di Liao^1,2^, Yin-Wei Cheng^1,4^, Li-Yan Li^1,2^, Li-Yan Xu^1,2,4^*****, En-Min Li^1^*****

^1^ The Key Laboratory of Molecular Biology for High Cancer Incidence Coastal Chaoshan Area, Department of Biochemistry and Molecular Biology, Shantou University Medical College, Shantou 515041, Guangdong, P. R. China.

^2^ Guangdong Provincial Key Laboratory of Infectious Diseases and Molecular Immunopathology, Institute of Oncologic Pathology, Shantou University Medical College, Shantou 515041, Guangdong, P. R. China.

^3^ Clinical Research Center, Shantou Central Hospital, Shantou 515041, Guangdong, P. R. China.

^4^ Cancer Research Center, Shantou University Medical College, Shantou 515041, Guangdong, P. R. China.

^5^ Department of Pathology, the Fifth Affiliated Hospital, Sun Yat-sen University, Zhuhai 519000, Guangdong, P. R. China.

^†^These authors contributed equally: Yu-Fei Cao, Lei Xie

***Corresponding Authors**: Dr. En-Min Li, Department of Biochemistry and Molecular Biology, Shantou University Medical College, No. 22, Xinling Road, Shantou 515041, Guangdong, P.R. China. Phone: 86-754-88900413; Fax: 86-754-88900847; E-mail: nmli@stu.edu.cn; Dr. Li-Yan Xu, Institute of Oncologic Pathology, Shantou University Medical College, No. 22, Xinling Road, Shantou 515041, Guangdong, P.R. China. Phone: 86-754-88900464; Fax: 86-754-88900847; E-mail: lyxu@stu.edu.cn.

Running title: Targeting the USP10-ANLN axis inhibits ESCC

**Abstract**

Anillin (ANLN) is a mitosis-related protein that promotes contractile ring formation and cytokinesis, but its cell cycle-dependent degradation mechanisms in cancer cells remain unclear. Here, we show that high expression of ANLN promotes cytokinesis and proliferation in esophageal squamous cell carcinoma (ESCC) cells and is associated with poor prognosis in ESCC patients. Furthermore, the findings of the study showed that the deubiquitinating enzyme USP10 interacts with ANLN and positively regulates ANLN protein levels. USP10 removes the K11- and K63-linked ubiquitin chains of ANLN through its deubiquitinase activity and prevents ANLN ubiquitin-mediated degradation. Importantly, USP10 promotes contractile ring assembly at the cytokinetic furrow as well as cytokinesis by stabilizing ANLN. Interestingly, USP10 and the E3 ubiquitin ligase APC/C co-activator Cdh1 formed a functional complex with ANLN in a non-competitive manner to balance ANLN protein levels. In addition, the macrolide compound FW-04-806 (F806), a natural compound with potential for treating ESCC, inhibited the mitosis of ESCC cells by targeting USP10 and promoting ANLN degradation. F806 selectively targeted USP10 and inhibited its catalytic activity but did not affect the binding of Cdh1 to ANLN and alters the balance of the USP10-Cdh1-ANLN complex. Additionally, USP10 expression was positively correlated with ANLN level and poor prognosis of ESCC patients. Overall, targeting the USP10-ANLN axis can effectively inhibit ESCC cell-cycle progression.

**Introduction**

Mitosis involves the accurate separation of genetic material and daughter cells [[1](#_ENREF_1)]. Mitotic defects are closely related to tumor progression, and in most cases, are related to specific changes in the regulation of mitotic kinases [[2](#_ENREF_2)]. Therefore, mitotic kinase inhibitors have been developed and used in cancer treatment. However, resistance to mitotic kinase inhibitors and side effects have been reported in patients [[3](#_ENREF_3), [4](#_ENREF_4)]. Exploring targets other than kinases to develop new mitotic inhibitors is a novel strategy.

In addition to phosphorylation, mitosis mainly depends on the strict regulation of proteins by ubiquitination [[5](#_ENREF_5)]. Ubiquitination is a post-translational modification, which involves tagging proteins for degradation, and is mediated by ubiquitin-activating (E1), -conjugating (E2), or -ligating (E3) enzymes [[6](#_ENREF_6)]. The E3 ligase APC/C, which mediates the degradation of mitotic proteins, is the main regulator of mitosis. APC/C works in combination with the co-activator Cdc20 or Cdh1 to specifically recognize substrates [[5](#_ENREF_5), [7](#_ENREF_7)]. Abnormalities in the ubiquitination system during mitosis often lead to tumorigenesis. Cdc20 overexpression can maintain tumor-initiating cells by degrading p21^CIP1/WAF1^ [[8-10](#_ENREF_8)]. Thus, targeting the ubiquitination system could be an effective strategy for cancer treatment. Tosyl-L-arginine methyl ester (TAME), an APC/C inhibitor, preferentially inhibits APC/C-Cdc20 instead of APC/C-Cdh1 to inhibit mitosis in cancer cells [[8](#_ENREF_8)].

Although the basic role of ubiquitination has been established in mitosis, deubiquitination is still poorly understood. Deubiquitinating enzymes (DUBs), a class of cysteine proteases or metalloproteases that counteract the activities E3 ligases, also play important roles in cell-cycle and cancer progression [[11](#_ENREF_11)]. The human genome encodes more than 100 DUBs, which are widely involved in key regulatory processes, so they may provide new therapeutic targets [[11-13](#_ENREF_11)]. However, roles of DUBs and their substrates in regulating mitosis are poorly understood.

Anillin (ANLN) is a mitotic protein that promotes cytokinesis by recruiting contractile ring components [[14](#_ENREF_14), [15](#_ENREF_15)]. ANLN expression in lung cancer is regulated by the PI3K pathway and promotes proliferation by activating RhoA [[16](#_ENREF_16)]. ANLN is known as an M phase marker and is degraded by APC/C-Cdh1 during M/G1 transition [[17](#_ENREF_17), [18](#_ENREF_18)]. However, it is not clear whether ANLN ubiquitination is related to tumor progression or treatment; there are no reports indicating whether ANLN is regulated by deubiquitination.

Here, we reveal ANLN plays a crucial role in cell division of esophageal squamous cell carcinoma (ESCC) and identify USP10 as a novel DUB for ANLN. USP10 removes the K11- and K63-linked ubiquitin chains of ANLN and prevents ANLN ubiquitin-mediated degradation. USP10 promotes contractile ring assembly at the cytokinetic furrow by stabilizing ANLN. USP10, Cdh1 and ANLN form a complex to balance ANLN levels, and the macrolide compound FW-04-806 (F806) selectively targets and inhibits USP10 to induce ANLN degradation. Moreover, USP10 is highly expressed in clinical ESCC samples, showing a positive correlation with ANLN levels and poor prognosis in patients. Therefore, targeting the USP10-ANLN axis can effectively inhibit the mitosis of ESCC.

**Results**

**Highly expressed ANLN participates in ESCC malignant progression by promoting cytokinesis and proliferation**

The role of ANLN was first examined in ESCC. Based on the standardized pan-cancer dataset in the UCSC database, we analyzed ANLN gene expression in the samples. Significant up-regulation of ANLN was observed in 33 types of tumors (Supplementary Figure S1A and Supplementary Data 6) (full names of the various tumors are shown in Supplementary Table 1). Additionally, analyses of ESCC GeneChip data (GSE53625) and RNA-seq data (SRP064894) confirmed an increase in ANLN mRNA expression in ESCC tissues compared with that in normal tissues (Supplementary Figure S1B, C) [[19](#_ENREF_19), [20](#_ENREF_20)]. Similarly, ANLN protein expression was higher in ESCC tissues of 124 patients (IPX0002501000) than in normal tissues (Supplementary Figure S1D and Supplementary Data 1) [[21](#_ENREF_21)]. Survival analysis on the basis of ANLN expression, which was conducted using the Kaplan–Meier method, in ESCC (GSE53625 dataset) showed that the overall survival of patients with high ANLN expression was lower than that of patients with low ANLN expression (Supplementary Figure S1E, log-rank test, *P*=0.0159). Similar results were obtained in another cohort (IPX0002501000) (Supplementary Figure S1F and Supplementary Data 1, log-rank test, *P*=0.0001). Furthermore, ANLN expression was confirmed by immunohistochemical staining in 104 ESCC and normal tissues. Results showed that ANLN expression in ESCC tissues was higher than that in normal tissues (Figure 1A, B), regardless of ANLN localization in the nucleus or cytoplasm (Supplementary Figure S1G, H). The same survival analysis using ANLN levels from immunohistochemical experiments also yielded the same results as the previous two independent cohorts (Figure 1C). To determine the role of ANLN in ESCC, KYSE150 and KYSE510 ESCC cells were used to verify the pathological function of ANLN. Results showed that downregulating ANLN expression inhibited the proliferation of cells (Figure 1D-F). Consistently, ANLN knockdown inhibited the proliferation of KYSE30 and KYSE450 cells (Supplementary Figure S1I, J). Additionally, reduction of ANLN induced G2/M phase arrest in ESCC cells (Figure 1G). At the molecular level, ANLN knockdown downregulated the expression of cell-cycle markers, such as cyclin B1 and E2 (Figure 1H). To further confirm the role of ANLN in the cytokinesis of ESCC cells, we generated cells stably expressing HA-ANLN and depleted endogenous ANLN with 3’UTR siRNA. Results showed that reintroduction of HA-ANLN rescued the furrow localization defect of the contractile ring caused by ANLN knockdown (Figure 1I, J). Similarly, both cyclin and cell proliferation reductions caused by ANLN knockdown were rescued by HA-ANLN overexpression (Figure 1K, L). Therefore, we concluded that ANLN is abnormally highly expressed in ESCC and associated with poor patient prognosis, and promotes ESCC proliferation by regulating contractile ring localization and cytokinesis.

**USP10 interacts with ANLN and positively regulates ANLN protein level**

To explore the mechanism by which ANLN expression is regulated, we employed affinity purification and mass spectrometry to interrogate ANLN interactome. Mass spectrometry analysis of the endogenous ANLN-containing protein complex revealed that ANLN was associated with a number of DUBs, including USP10, USP7, USP16, USP39 and PRPF8, of which USP10 had the highest score (Figure 2A, B, Supplementary Table 2 and Supplementary Data 2). Following co-immunoprecipitation, ANLN was found to physically interact with USP10 (Figure 2C). Similarly, endogenous ANLN also interacted with USP10 in ESCC cells (Figure 2D, E). Furthermore, ANLN was found to interact with amino acids 210-802 of USP10, but not the amino acid 403-802 of USP10 (Figure 2F, G). Therefore, amino acids 210-402 of USP10 are necessary for the interaction between ANLN and USP10. We also show that the C-terminal RBD, C2 and PH domains of ANLN did not mediate the interaction with USP10, and demonstrate that amino acids 1-454 of ANLN directly interacts with USP10 based on in vitro GST pull down assays (Figure 2H-J). Since USP10 is a deubiquitinase, we hypothesized that USP10 could regulate the protein stability of ANLN. To confirm the ANLN regulatory effect by USP10, we treated cells with spautin-1 or HBX19818 (inhibitors of USP10). USP10 inhibition reduced ANLN protein levels (Figure 2K, L). Consistently, knockdown of USP10 down-regulated ANLN protein levels, but not mRNA levels (Figure 2M and Supplementary Figure S2A). Overexpression of USP10 wild-type but not the C428A (USP10 inactive mutation) could rescue the reduction of ANLN (Figure 2N).

**USP10 removes K11- and K63-linked ubiquitin chains of ANLN through its deubiquitinase activity**

To further identify USP10 as a novel DUB of ANLN, the half-life of ANLN protein in USP10 knockdown or USP10 overexpressing cells was analyzed by a cycloheximide (CHX) chase assay. Results showed that USP10 knockdown reduced the protein stability of ANLN, whereas USP10 overexpression enhanced the protein stability of ANLN (Figure 3A, B). Additionally, the ANLN levels decreased by USP10 knockdown were restored by MG132 treatment (Figure 3C). Ubiquitination assays showed that USP10 knockdown enhanced the ubiquitination of endogenous ANLN (Figure 3D). Ubiquitination assays also indicated that USP10 wild-type but not the C428A removed the polyubiquitin chain of Flag-ANLN (Figure 3E). Importantly, USP10 could remove the K11- and K63-linked ubiquitin chains from ANLN (Figure 3F). In aggregate, these results show that USP10 maintains ANLN stability and cleaves the polyubiquitin chains of ANLN through its deubiquitinase activity.

**USP10 promotes contractile ring localization and cytokinesis by stabilizing ANLN protein**

We have shown that ANLN functions in cytokinesis in ESCC cells and therefore explored whether USP10 is involved in cytokinesis by stabilizing ANLN. USP10 knockdown cells were synchronized with thymidine and then tested for ANLN expression in different cell-cycle phases. Results showed that USP10 knockdown mainly downregulated ANLN levels in M and M/G1 phase (Figure 4A). Co-immunoprecipitation experiments showed that USP10 and ANLN also interacted mainly in the M and M/G1 phases (Figure 4B). Similarly, immunofluorescence assays confirmed the co-localization of USP10 and ANLN in dividing cells, with no co-localization in interphase cells (Figure 4C). To further confirm the role of USP10 on the cell-cycle, synchronized USP10 knockdown cells were examined by flow cytometry. Results showed that depletion of USP10 delayed the M/G1 transition (Figure 4D). Furthermore, USP10 knockdown resulted in a decrease in cyclin B1 and E2, similar to ANLN knockdown (Figure 4E and 1H). Next, we asked whether USP10 could influence contractile ring localization by stabilizing ANLN. USP10 was depleted in cells stably expressing HA-ANLN, and the localization of the contractile ring component was examined by immunofluorescence. USP10 knockdown inhibited the enrichment of contractile rings at the furrow, and reintroduction of HA-ANLN rescued the furrow localization of contractile rings (Figure 4F, G). Biochemical studies also showed that the decrease in cyclin caused by USP10 knockdown could be restored by overexpression of HA-ANLN (Figure 4H). Tthese results indicate that USP10 promotes correct furrow localization of contractile rings and the completion of cytokinesis by stabilizing ANLN.

**USP10 and Cdh1 form a complex with ANLN in a non-competitive manner to balance ANLN levels**

It has been reported that the E3 ligase complex, APC/C-Cdh1 regulates the degradation of ANLN [[15](#_ENREF_15), [17](#_ENREF_17)]. We were able to observe that Cdh1 knockdown prevented the degradation of ANLN during mitotic exit in ESCC cells (Supplementary Figure S2B). Interestingly, we found that USP10 and Cdh1 can also interact (Figure 5A). E3 ligases and DUBs can balance the protein levels of substrates in a competitive or non-competitive manner [[11](#_ENREF_11), [22](#_ENREF_22), [23](#_ENREF_23)]. Therefore, the interaction of the three proteins was further explored. Results show that ANLN, USP10 and Cdh1 can form a large complex in vivo and in vitro (Figure 5B-D). In addition, the interaction of USP10 and Cdh1 is independent of ANLN expression levels, suggesting that they may recognize substrates in a non-competitive manner (Figure 5B). Similarly, the interaction of ANLN and Cdh1 was not affected by USP10 expression levels (Figure 5C). The interaction between endogenous USP10 and Cdh1 was examined in ANLN knockdown cells. Consistently, the interaction between USP10 and Cdh1 was not dependent on the cellular ANLN level (Figure 5E). USP10 knockdown did not affect the interaction between Cdh1 and ANLN, and Cdh1 knockdown did not affect the interaction between USP10 and ANLN (Figure 5F, G). Next, as expected, it was found that USP10 and Cdh1 could balance each other to regulate ANLN ubiquitination and protein levels (Figure 5H, I). To explore the cell-cycle in which the complex is formed, we synchronized the cells with a double thymidine block (DTB) and examined the interaction. Interestingly, the interaction of ANLN and USP10 or Cdh1 showed the same trend 9–15 h after thymidine release, indicating that the ternary complex was mainly formed in the M and M/G1 phases (Figure 5J). Consistently, Cdh1 knockdown restored USP10 knockdown-mediated ANLN reduction when the cells were in the M phase, but there was no such regulation in the G1/S phase (Figure 5K). Several DUBs interact with E3 ligases to regulate the stability of E3 ligases [[11](#_ENREF_11)]. We showed that USP10 knockdown did not reduce Cdh1 protein level, and Cdh1 depletion did not affect USP10 protein level (Figure 5L, M). Several substrates may also regulate the stability of DUBs [[24](#_ENREF_24)]. However, the results indicated that ANLN knockdown did not affect the protein level of USP10 (Figure 5N). In summary, these results indicate that USP10 and Cdh1 form a complex with ANLN in the M and M/G1 phases, and balance ANLN levels in a non-competitive manner.

**FIM-04-806 induces ANLN degradation by inhibiting USP10 deubiquitinase activity**

Previous studies have shown that FIM-04-806 (F806), a macrolide analog, is effective in treating ESCC [[25](#_ENREF_25), [26](#_ENREF_26)]. Proteomic analysis showed that F806 mainly inhibited ESCC by reducing the expression of mitosis-related proteins, including ANLN (Supplementary Figure S3A-E and Supplementary Data 1, 3 and 4). ANLN was previously considered a promising therapeutic target [[27](#_ENREF_27)]. However, a suitable inhibitor against ANLN has yet to be identified. To explore an ANLN inhibitory ability of F806, paraffin-embedded tumor tissues from tumor-bearing mice treated with F806 were analyzed by immunohistochemistry. ANLN expression in tumor tissues of F806-treated mice was lower than that in the control group (Figure 6A and Supplementary Figure S3F). At the cellular level, F806 also suppressed ANLN protein levels in ESCC cells (Supplementary Figure S3G). Interestingly, we found that F806 downregulated ANLN expression through the proteasome pathway but not the lysosomal pathway and was able to enhance the level of ANLN ubiquitination (Figure 6B-D and Supplementary Figure S3H-J). Drugs, particularly anti-tumor drugs, are more likely to act as inhibitors rather than activators [[24](#_ENREF_24), [28](#_ENREF_28)]. Therefore, we examined the inhibitory effect of F806 on ANLN deubiquitination. Proteomic analysis indicated that USP10 was the deubiquitinase most likely to be inhibited by F806 (Supplementary Figure S3K, Supplementary Table 3 and Supplementary Data 2, 3). To further examine the relationship between USP10 and F806 in ANLN degradation, cells overexpressing USP10 were treated with F806. Results showed that overexpression of USP10 wild-type but not CA reversed F806-induced ANLN reduction (Figure 6E, F). F806-mediated ANLN degradation was almost lost when USP10 was silenced by siRNA (Figure 6G). USP10 overexpression-mediated reduction of K11- and K63-linked ubiquitin chains of ANLN could be reversed by F806 treatment (Figure 6H). Next, we asked whether F806 affected the deubiquitinase activity of USP10 by using the suicide probe hemagglutinin-ubiquitin-vinyl sulfone (HA-Ub-VS). Results showed that the Ub-VS-labeled USP10 in cells treated with F806 was significantly reduced, indicating that F806 inhibited the activity of USP10 (Figure 6I). The activity of purified USP10 was also inhibited in a dose-dependent manner by F806 in vitro (Figure 6J and Supplementary Figure S4A). To corroborate the HA-Ub-VS assays, Ub-AMC, a fluorogenic substrate for Ub hydrolases, was used to examine the activity of USP10. Results showed that F806 significantly inhibited the activity of USP10 in vitro in a dose-dependent manner with a half-maximal inhibitory concentration (IC50) of 3.462 μM in a 60 min assay (Figure 6K, L and Supplementary Figure S4B).

**FIM-04-806 alters the balance of the USP10-ANLN-Cdh1 complex by targeting USP10 and inhibits the M/G1 transition of ESCC cells**

To further confirm that F806 directly targets and binds USP10, a Biacore platform was used for surface plasmon resonance (SPR) analysis. Results showed that purified USP10 interacted directly with F806 with an equilibrium dissociation constant (KD) of about 2.751E-5 M (Figure 7A). Next, we asked whether APC/C-Cdh1 is involved in F806-induced ANLN degradation. Cdh1 reduction could prevent F806-induced ANLN degradation (Figure 7B). Additionally, F806 treatment decreased the interaction between ANLN and USP10, but did not affect the interaction between ANLN and Cdh1 (Figure 7C). We then examined the regulatory effect of F806 on the USP10-ANLN-Cdh1 complex in the M and M/G1 phases and observed similar results (Figure 7D). Furthermore, the cellular fate resulting from F806 targeting the USP10-ANLN axis was explored in ESCC cells. At the molecular level, F806 treatment decreased the expression of the cyclin, similar to ANLN or USP10 knockdown (Figure 7E, 1H and 4E). Flow cytometry indicated that treatment with F806 resulted in G2/M phase arrest, similar to ANLN or USP10 knockdown (Supplementary Figure S5A, Figure 1G and 4D). To further examine the effect of F806 on M phase, we used a DTB. As expected, KYSE150 cells released for 9 h were in G2/M phase with increased ANLN expression, whereas F806 inhibited M/G1 transition and reduced ANLN levels (Figure 7F and Supplementary Figure S5B). Similarly, M/G1 transition in KYSE510 cells was suppressed by F806 (Supplementary Figure S5C). Immunofluorescence assays also showed that contractile ring localization was blocked by F806 (Figure 7G, H). The re-entry of ANLN into the nucleus signifies the end of the M/G1 transition [[15](#_ENREF_15)]; thus, we calculated the nuclear localization ratio of ANLN in cells released for 15 h and found that F806 inhibited the entry of ANLN into the nucleus (Figure 7I). These data indicate that F806 targetes USP10 but does not affect the binding of Cdh1 to ANLN, thus altering the ubiquitination-deubiquitination balance of ANLN and inducing M-phase delay in ESCC cells (Figure 7J).

**Expression of USP10 is positively correlates with ANLN level and poor prognosis of ESCC patients**

Since USP10 enhances ANLN expression and is a regulator of mitosis, the clinical significance of USP10 and the relationship between USP10 and ANLN expression in ESCC were verified. Immunohistochemical staining of 104 samples showed that USP10 expression in ESCC tissues was higher than that in normal tissues (Figure 8A, B). Similarly, USP10 expression in ESCC tissues of 124 patients (IPX0002501000) was higher than that in normal tissues (Figure 8C and Supplementary Data 1) [[21](#_ENREF_21)]. At the mRNA level, GSE53625 GeneChip data and RNA-seq data (SRP064894) showed that USP10 mRNA levels were highly expressed in ESCC tissues (Figure 8D, E) [[19](#_ENREF_19), [20](#_ENREF_20)]. Moreover, expression of USP10 and ANLN was positively correlated in ESCC (Figure 8F-I and Supplementary Data 1), and USP10 staining intensity in 104 ESCC tissues was significantly associated with overall survival of patients (Figure 8J). Analysis of the GSE53625 GeneChip data and tissue samples (IPX0002501000) confirmed that USP10 expression was correlated with overall survival of patients (Figure 8K, L). Overall, it can be concluded that high USP10 expression is associated with the progression of ESCC, and that the USP10-ANLN axis is a key factor affecting the survival of patients. Furthermore, from the perspective of the efficacy of F806, the USP10-ANLN axis is expected to become an effective target for treating ESCC.

**Discussion**

ESCC is the most common histological subtype of esophageal cancer, with a high incidence in eastern Asian countries, and with a 5-year survival rate of less than 20% [[29](#_ENREF_29)]. Several molecular markers of ESCC have been identified. However, owing to the lack of drugs and pharmacological molecular mechanisms, these markers are rarely used as therapeutic targets. Here, USP10-ANLN axis was identified as both a molecular marker and a potential therapeutic target for treating ESCC. Previous study found that F806 can preferentially inhibit ESCC cells, with low toxicity to normal cells and tissues [[26](#_ENREF_26)]. F806 inhibits ESCC cell growth by regulating Grb2 and the Rho family [[25](#_ENREF_25), [30](#_ENREF_30)]. However, the direct target of F806 in ESCC has not been reported, and its anti-cancer mechanism is not yet fully understood. Here, F806 was identified as a potential mitotic inhibitor, which targets USP10 and induces ANLN degradation, providing novel insights into the anticancer mechanism of macrolide antibiotics. Future studies should attempt to analyze the structure of USP10 and identify the binding sites of F806 between USP10.

It was reported that USP10 functions in YAP and p53 pathways [[24](#_ENREF_24), [31](#_ENREF_31), [32](#_ENREF_32)]. However, we found that USP10 regulates mitotic markers in ESCC cells, such as ANLN, cyclin B1, and p-Cdc2 (Y15) but not p53 or YAP (Figure 4E and Supplementary Figure S6). Based on gene set enrichment analysis (GSEA), we found that USP10 functions in the cell-cycle (Supplementary Figure S7). USP10 knockdown induced a contractile ring localization defect and a delay in M/G1 transition (Figure 4D, F), providing insight into the role of USP10 in the cell-cycle. USP10 acts as a cancer-promoting factor in liver cancer and acute myeloid leukemia through stabilization of YAP and FLT3 [[31](#_ENREF_31), [33](#_ENREF_33)]. In contrast, USP10 exerts a tumor suppressor effect by stabilizing KLF4 in lung cancer [[34](#_ENREF_34)]. Since USP10 positively regulates oncoproteins, it is more likely to act as a tumor-promoting factor for ESCC. Consistently, there is an increase in USP10 expression in ESCC tissues, which is positively correlated with ANLN levels and poor patient prognosis (Figure 8). This indicates that USP10 regulates specific substrates in different tumors, thus performing different functions.

The operation of contractile rings is a highly dynamic process, with repeated turnover of disassembly and self-assembly to maintain a stable contractile force [[35](#_ENREF_35)]. Results showed that cytosolic ANLN could still be observed even if ANLN was located in the contractile ring (Figure 4C). Since the membrane localization of ANLN are regulated by phosphorylation [[36](#_ENREF_36)], so we speculate that membrane ANLN and cytosolic ANLN have different phosphorylation states. Phosphorylation of a substrate is usually crosstalk with ubiquitination [[37](#_ENREF_37), [38](#_ENREF_38)]. Since the phosphorylation and ubiquitination of ANLN occur during the same period, phosphorylation may mediate the recognition of ANLN by APC/C-Cdh1 and USP10. Excessive increases in ANLN ubiquitination may accelerate the disassembly of contractile rings and delay self-assembly. This model explains the kinetic process of ANLN-mediated contractile ring assembly and the role of ANLN degradation in cytokinesis. Moreover, phosphorylation and DUB activity can crosstalk. Dietachmayr et al. reported that phosphorylation controls the activity of USP9X to regulate the stability of WT1 [[39](#_ENREF_39)]. Results show that there is no cell cycle-dependent expression of USP10, even though it is a mitotic regulator (Figure 5J). We speculate that kinase-mediated phosphorylation regulates the activity of USP10, allowing USP10 to regulate mitotic proteins independent of its expression.

E3–DUB interactions allow fine-tuning of the ubiquitylation status of a common substrate, which generally involves a non-competitive binding mechanism (antagonistic pattern) [[11](#_ENREF_11), [22](#_ENREF_22), [23](#_ENREF_23), [40](#_ENREF_40)]. We show that either knockdown or overexpression of ANLN does not affect the interaction of USP10 and Cdh1, further confirming this non-competitive molecular mechanism (Figure 5B, E). Interestingly, a substrate may contain multiple ubiquitination sites or be modified by different types of ubiquitin chains [[6](#_ENREF_6)]. Therefore, regulation of the same substrate by multiple DUBs is a common mechanism. Tumor-related proteins, such as c-Myc, p53 and Beclin1, are regulated by at least two DUBs [[24](#_ENREF_24), [41-43](#_ENREF_41)]. Since USP10 overexpression cannot completely remove the ubiquitin chains of ANLN (Figure 3E), there may be unidentified DUBs involved in regulating ANLN.

Overall, we identify USP10 as a novel DUB for ANLN that promotes contractile ring localization by stabilizing ANLN. USP10, Cdh1 and ANLN form a complex to balance ANLN levels. Furthermore, F806 targets the USP10-ANLN axis to inhibit mitosis in ESCC cells.

**Materials and methods**

**Patients and samples**

In total, 104 ESCC tissues and adjacent normal esophageal tissues were excised from ESCC patients at the Shantou Central Hospital (Shantou, China) from 2007–2010. Samples were embedded in paraffin wax blocks and used for tissue microarray construction before immunohistochemical staining. All cases were classified according to the eight edition of the tumor-node-metastasis (TNM) classification system of the American Joint Committee on Cancer (AJCC) and Union for International Cancer Control (UICC). The samples were confirmed by pathologists in the Clinical Pathology Department of the Hospital [[44](#_ENREF_44)].

Information on various clinicopathological characteristics listed in Supplementary Table 4 were obtained from medical records. Samples from patients who had other tumors or died of other causes were excluded. Tissues were obtained with the approval of the Committee for Ethical Reviews of Research Involving Human Subjects at the Shantou University Medical College and the Shantou Central Hospital, and only samples from patients who provided informed consent were included in the study [[45](#_ENREF_45)].

**Immunohistochemistry**

Tissue microarrays of human ESCC samples and tumor tissues from F806-treated mice were serially sectioned (4 μm) and baked for 4 h at 60°C. After dewaxing in xylene, slides were dehydrated in an ethanol gradient and rinsed three times in distilled water for 3 min each. Samples were then heated in citrate buffer until boiling, thawed for 10 min, cooled for 1 h at 25°C, and incubated with 3% H_2_O_2_ for 10 min at room temperature to inactivate peroxidase activity. Thereafter, slides with tissue microarrays were incubated with 0.1 ml serum blocking solution for 10 min, followed by incubation with anti-ANLN (1:100, Santa Cruz, sc-271814) or anti-USP10 (1:100, Abcam, ab109219) overnight at 4°C. Immunostaining was performed using a 2-step protocol and a PV-9000 Polymer Detection System (ZSGB-BIO, Beijing, China), according to the manufacturer’s instructions. Subsequently, the slides were incubated with 0.1 ml DAB for 2–5 min and thoroughly washed with distilled water. Finally, the samples were counterstained with Mayer’s hematoxylin, dehydrated, and mounted. ANLN or USP10 expression was scored using an automatic multispectral section analysis system (Perkin Elmer, Waltham, MA, USA). The staining intensity was classified as 0 (no staining), 1 (weak staining), 2 (moderate staining), and 3 (strong staining). The immunohistochemistry score was then calculated by adding the staining intensity to the product of the corresponding percentage of positive tumor cells [[46](#_ENREF_46)].

**Cell culture**

KYSE150, KYSE510 and KYSE30 ESCC cells were maintained in RPMI1640 medium (Thermo Fisher Scientific) supplemented with 10% fetal bovine serum (GIBCO), 100 U/ml penicillin G, and streptomycin. Dulbecco’s modified Eagle’s medium (GIBCO) supplemented with 10% fetal bovine serum was used for HEK293T, KYSE450 and EC109 cell culture. Cells were routinely cultured at 37°C in a humidified atmosphere containing 5% CO_2_ [[47](#_ENREF_47)]. Cell lines were authenticated by short tandem repeat profiling and were routinely tested for mycoplasma contamination (Supplementary Data 5).

**Mouse xenograft model**

A xenograft model was performed as previously described [[26](#_ENREF_26), [45](#_ENREF_45)]. Five-week-old male nude/nude mice were purchased from Vital River Laboratories (Beijing, China). On the first day, 1 million ESCC cells were inoculated subcutaneously into the right flank of the mouse. The next day, mice were randomized into two groups with 6 mice in each group, and tumor growth was monitored daily. On Day 12, when the tumor volume reached approximately 0.5 mm in diameter, drug administration was initiated. A 100 mg/ml F806 solution was diluted in 0.9% NaCl containing 5% Tween-80 and 5% polyethylene glycol-400 (Sigma, St Louis MO, USA) to a final concentration such that a dose of 4 mg/kg, in 200 μL solution, was administered to each mouse in the treatment group. F806 or control solution was intraperitoneally administered daily for 21 days. The mice were then euthanized, and the tumors were excised, weighed, and preserved in formalin for histological analysis.

**Small interfering RNA (siRNA) knockdown**

Single siRNA oligonucleotides targeting human ANLN, Cdh1, and USP10 (GenePharma, Supplementary Table 5) and negative control siRNA were diluted in siRNA transfection medium (31985-070, Life Technologies) and mixed with siRNA Transfection Reagent (13778150, Life Technologies), according to the manufacturer’s instructions [[48](#_ENREF_48)]. Cells were incubated with the transfection complexes for 8 h, followed by incubation in normal growth medium for 40 h.

**Plasmids**

pCMV-N-HA, pCMV-N-Flag, pBOBI-C-3×HA, pBOBI-N-MYC, pGEX-6P-1 and pET-32a vectors were used to construct the expression plasmids. ANLN and its mutants were cloned into pBOBI-C-3×HA or pET-32a vectors. USP10 and its mutants were cloned into pCMV-N-Flag, pCMV-N-HA or pET-32a vectors. A stop codon was inserted before the HA-tag of pCMV-C-HA to construct pCMV-USP10 and pCMV-USP10-CA (C428A, USP10 inactive mutation). pCMV-HA-Ub (P0554) and pEnCMV-3×Flag-ANLN (P10675) were purchased from the MiaoLing Plasmid Sharing Platform. FZR1 (Cdh1) was cloned into the pBOBI-C-3×HA and pBOBI-N-MYC vectors. PEF-HA-Ub-K6, PEF-HA-Ub-K11, PEF-HA-Ub-K27, PEF-HA-Ub-K29, PEF-HA-Ub-K33, PEF-HA-Ub-K48, PEF-HA-Ub-K63 plasmids were kindly provided by Professor Ceshi Chen (Chinese Academy of Sciences, Kunming Institute of Zoology, China) [[49](#_ENREF_49)]. All plasmids were sequenced at the Beijing Genomics Institute (BGI). Primers used in the study are listed in Supplementary Table 6.

**Colony formation assay**

Transfected cells were cultured for 48 h, then 1000 cells were incubated at 37°C for 1–2 weeks under 5% CO_2_. When visible cell clones appeared, the culture was terminated by washing the cells twice with 1×PBS, then fixing in a mixture of methanol and glacial acetic acid (3:1) for 15 min, and staining with hematoxylin for 30 min. The cells were rinsed with water and dried in air. Images were obtained using an imaging system, and the cell clones were counted using Image J software [[50](#_ENREF_50), [51](#_ENREF_51)].

**Real-time cell analysis (RTCA)**

RTCA was performed as previously described [[47](#_ENREF_47)]. Cells were transfected with siRNA for 48 h, then 10,000 cells per well were seeded in a 16-well E-plate and placed in an xCELLigence Real-Time Cell Analysis DP instrument. The software was run, and cell proliferation was examined in real time.

**Western blotting**

Cells were lysed in Laemmli sample buffer (Bio-Rad, 1610747). Proteins were quantified using a Pierce 660 nm Protein Assay Kit (Thermo Scientific, 22662) according to the protocol provided by the manufacturer [[52](#_ENREF_52), [53](#_ENREF_53)]. Proteins were separated by sodium dodecyl sulfate-polyacrylamide gel electrophoresis and transferred to polyvinylidene difluoride membranes [[54](#_ENREF_54)]. The membranes were blocked with 5% non-fat milk and incubated with antibodies against ANLN (1:1000, Santa Cruz, sc-271814), ANLN (1:1000, Abcam, ab99352), USP10 (1:3000, Proteintech, 19374-1-AP), FZR1 (Cdh1) (1:500, Santa Cruz, sc-56312), cyclin B1 (1:1000, Santa Cruz, sc-245), cyclin A2 (1:1000, Cell Signaling Technology, #91500), cyclin E2 (1:1000, Cell Signaling Technology, #4132), cyclin D1 (1:1000, Cell Signaling Technology, #2978), p-Cdc2 (Tyr15) (1:1000, Cell Signaling Technology, #4539), phospho-histone H3 (S10) (1:3000, Abcam, mAbcam 14955), ubiquitin (1:1000, Proteintech, 10201-2-AP), YAP1 (1:1000, Proteintech, 13584-1-AP), TAZ (1:1000, Proteintech, 66500-1-Ig), p53 (1:1000, DAKO, M356701), GAPDH (1:3000, Proteintech, 60004-1-Ig), β-actin (1:1000, Santa Cruz, sc-47778), HA-tag rabbit antibody (1:3000, Cell Signaling Technology, C29F4), HA-tag monoclonal antibody (1:3000, Proteintech, 66006-2-Ig) and Flag M2 monoclonal antibody (1:5000, Sigma, F3165-1MG). Proteins were detected by incubation with horseradish peroxidase-conjugated secondary antibodies and visualized using Western Blot Luminol Reagent (Santa Cruz). The raw data of western blotting has been submitted to the source data file.

**Double thymidine block**

A 2 mg/ml thymine (Sigma) solution was prepared by dissolving 0.2 g thymine in 100 ml of RPMI 1640 medium containing 10% FBS. Cells were blocked for 12 h with the 2 mg/ml thymine solution, washed three times with PBS, and released with 2 ml fresh culture medium for 8 h. Thereafter, a second block was performed using the 2 mg/ml thymine solution for 20 h. Cells were washed three times with PBS and released with fresh culture medium for different times [[17](#_ENREF_17), [55](#_ENREF_55)].

**Immunofluorescence assay**

Cells were fixed with 4% paraformaldehyde for 15 min, permeabilized with 0.1% Triton X-100 for 8 min, then blocked with 5% donkey serum for 1 h prior to incubation with ANLN (1: 50), USP10 (1: 50) or HA antibody (1: 500) overnight [[56](#_ENREF_56)]. Cells were incubated with Alexa fluor 647 donkey anti-mouse IgG (Jackson, 715-605-150, 1:200), Alexa fluor 488 donkey anti-rabbit IgG (Jackson, 711-545-152, 1:200) and Acti-stain 555 fluorescent phalloidin (Cytoskeleton, PHDH1-A, 1:200) in the dark for 1 h. After washing, cells were counterstained with DAPI (Beyotime, C1005, 1:2000) in the dark for 5 min, then observed under an LSM880 with Airyscan confocal laser scanning microscope (Carl Zeiss, Oberkochen, Germany) [[54](#_ENREF_54)].

**Flow cytometry analysis**

Cells were harvested and fixed in 70% cold ethanol at 4°C overnight. Subsequently, cells were permeabilized in PBS using 0.1% Triton X-100 and stained using 5 μg/ml propidium iodide (PI) and 50 μg/ml RNase for 30 min at 37℃ in the dark. After washing, the cells were immediately analyzed by flow cytometry using an Accuri C6 flow cytometer (BD) [[57](#_ENREF_57)].

**Immunoprecipitation assay**

Cells were harvested in EBC buffer (50 mM Tris pH 7.5, 120 mM NaCl, 0.5% NP-40, 1% protease inhibitor cocktail (MedChemExpress, HY-K0010), and incubated at 4°C for 8 h with 2 μg of ANLN antibody or normal mouse IgG (Santa Cruz, sc-2025) [[58](#_ENREF_58)]. Thereafter, 25 μl of protein A/G magnetic beads (MedChemExpress, HY-K0202) was added to the mixture, which was then incubated for an additional 8 h at 4°C. The immunoprecipitated protein complexes were washed four times with EBC buffer, supernatant was discarded, and the antibody/protein complexes were resuspended in 100 μl of loading buffer, boiled for 10 min, and assayed by western blotting.

**Ubiquitination assay**

ESCC cells transfected with HA-Ub were treated with MG132 (MedChemExpress, HY-13259) with or without F806, and then ubiquitination assays were performed using denature-IP [[34](#_ENREF_34), [59](#_ENREF_59)]. Briefly, cells were harvested with EBC buffer containing 4% SDS and 20 μM MG132, boiled at 95°C for 15 min, sonicated and centrifuged. The supernatant was diluted with SDS-free EBC buffer till its concentration became one tenth of the original value, and then was used for ANLN immunoprecipitation with ANLN antibody and protein A/G magnetic beads. For immunoprecipitation using HEK293T cells, plasmids encoding HA-Ub and Flag-ANLN were co-transfected and cells were treated with MG132 for 8 h before harvest, after which Flag-ANLN was immunoprecipitated with Flag magnetic beads (Thermo Fisher Scientific, UI294205).

**Protein purification and quantification**

The expression and purification of GST-tagged and His-tagged protein was performed as previously described [[60](#_ENREF_60)]. Transetta (DE3) Chemically Competent Cells (TransGen Biotech, CD801-03) were transformed with plasmids overnight, Cells were grown in LB medium (10g/l tryptone, 5g/l yeast extract, 10g/l NaCl) at 37 °C to an OD_600 nm_ value of 0.7, induced with 0.5 mM isopropyl β-D-1-thiogalactopyranoside (Amresco, 0487) and incubated at 16°C for 16 h. The bacteria were centrifuged and collected, and then lysed with GST lysis buffer (4.3 mM Na_2_HPO_4_, 1.47 mM KH_2_PO_4_ 137 mM NaCl, pH 7.3, 0.1% Triton X-100, 1mM PMSF) and 1 mg/ml lysozyme (Sigma, 62971). Lysates were centrifuged at 10000 rpm for 10 min, then the supernatant was incubated with GST Bind Resin (Millipore, 70541-5) or His Bind Resin (Millipore, 70666-3) at 4℃ for 3 hours, and then washed 6 times with GST buffer or His wash buffer (50 mM sodium phosphate, 300 mM NaCl, 20 mM imidazole). To obtain purified protein, the resin was eluted using GST (50 mM Tris-HCl pH 8.0, and 50 mM reduced form of glutathione) or His elution buffer (50 mM sodium phosphate, 300 mM NaCl, and 200 mM imidazole) [[60](#_ENREF_60)]. GST-USP10, His-USP10, His-ANLN and its mutants used in this study were purified according to the above protocol, and GST-Cdh1 was purchased from Abnova (H00051343-P01). For purified eukaryotic Flag-USP10 protein, HEK293T cells were transfected with an expression plasmid for 48 hours, and then EBC buffer and Flag magnetic beads were used for immunoprecipitation. To obtain the purified protein, beads containing Flag-USP10 were eluted with l mg /ml Flag peptide (MedChemExpress, HY-P0223) at 4℃ for 4 hours [[24](#_ENREF_24)]. All purified proteins were quantified using a Pierce BCA Protein Assay Kit (Thermo Scientific, 23225) according to the protocol provided by the manufacturer [[61](#_ENREF_61)]. Then the purity and concentration of the proteins were further confirmed by SDS-PAGE and Coomassie brilliant blue staining.

**In vitro GST pull down assay**

GST-tagged proteins were bound with GST Bind Resin in GST lysis buffer at 4°C for 2 h. Resins were washed twice with GST lysis buffer, then His-tagged proteins were added and the mixture was rotated at 4℃ for 4 h. Resins were washed six times with PBST buffer, boiled in SDS loading buffer and analyzed by western blotting [[60](#_ENREF_60)].

**Ub-AMC assay**

The USP10 deubiquitinating activity assay was conducted in Ub-AMC buffer (50 mM Tris-HCl, pH 7.5, 1 mM EDTA, 1 mg/ml ovalbumin, 5 mM MgCl_2_, 1 mM DTT, 2 mM ATP) with 1 μM Ub–7-amino-4-methylcoumarin (AMC)-conjugated protein (R&D Systems, U-550). Proteins were incubated for 1 h, and excitation at a wavelength of 345 nm and emission at a wavelength of 445 nm were measured at 37℃ in real time [[24](#_ENREF_24), [28](#_ENREF_28)].

**HA-Ub-VS assay**

Cells treated with F806 were lysed, on ice, in Ub-VS buffer (50 mM HEPES, pH 7.4, 250 mM sucrose, 10 mM MgCl_2_, 1% NP-40) for 5 min, sonicated and centrifuged. The supernatants (100 μg) were incubated with 2 mM ATP, 1 mM DTT and 2 μM HA-Ub-VS probe (R&D Systems, U-212-025) for 30 min at 37℃. The samples were then subjected to western blot analysis. For the in vitro HA-Ub-VS assay, 50 nM purified USP10 protein, 2 mM ATP, 1 mM DTT and 1 μM HA-Ub-VS probe was added into the Ub-VS buffer. The mixture was incubated at 37°C for 30 min, and then analyzed by western blotting [[62](#_ENREF_62), [63](#_ENREF_63)].

**Surface plasmon resonance (SPR) analysis**

A Biacore T200 (Cytiva) was used to determine the interaction between USP10 and F806. His-USP10 was diluted to 10 mM coupling buffer (Cytiva, BR100349) and was coupled to CM5 sensor chips (Cytiva, BR100530) by using an amine-coupling kit (Cytiva, BR100050). A series of F806 concentrations was made by dissolving in PBS running buffer (2 mM KH_2_PO_4_, 137 mM NaCl, 10 mM Na_2_HPO_4_, 2.7 mM KCl, 5% DMSO, pH 7.4). F806 solution was injected into the flow system at a flow rate of 30 μl/min at 25°C, a contact time of 120 s, and a dissociation time of 180 s. The binding kinetics were analyzed by Evaluation Software [[28](#_ENREF_28)].

**LC-MS/MS analysis**

Proteomic analysis was performed using a protocol described previously [21]. For global proteomic analysis of 9 ESCC cell lines, samples were processed according to the filter-aided sample preparation (FASP) method. The tryptic peptides were desalted with StageTips and lyophilized followed by labeling with TMT-11plex (Pierce) according to the manufacturer’s instructions. TMT MS experiments were performed on a nanoscale EASY-nLC 1200UHPLC system or nanoU3000UHPLC system (Thermo Fisher Scientific) connected to an Orbitrap Fusion Lumos equipped with a nanoelectrospray source (Thermo Fisher Scientific). For ANLN interaction proteomic analysis, ANLN in KYSE150 cells was enriched by immunoprecipitation. Samples were separated by sodium dodecyl sulfate-polyacrylamide gel electrophoresis, then the gel slices were cut to cubes and transferred to LoBind tubes (Eppendorf). Samples were treated with liquid chromatography–mass spectrometry (LC-MS) water and LC-MS ACN, dried in a Speedvac (Eppendorf), and reduced by mixing with ammonium bicarbonate and DTT. The samples were incubated at 56°C for 30 minutes and then the liquid was removed, ammonium bicarbonate and iodoacetamide were added to the gel pieces and incubated at 25°C for 30 minutes in the dark. The liquid was removed and washed with ammonium bicarbonate, ACN was added to dehydrate the gel pieces, then the solution was removed and the samples were dried in a Speedvac. Trypsin was used to digest the samples and ZipTips were used to purify and concentrate peptides for LC-MS/MS analysis. MS experiments were performed on a nanoscale UHPLC system (EASY-nLC1000, Proxeon Biosystems, Odense, Denmark) connected to an Orbitrap Q-Exactive mass spectrometer equipped with a nanoelectrospray source (Thermo Fisher Scientific, Waltham, MA, USA). The proteomic data of ESCC cells treated with F806 was obtained from our previous research, and the methods of SILAC cell culture and Nano HPLC/MS analysis have been described previously [[25](#_ENREF_25)].

**Bioinformatics analyses**

ANLN expression at the mRNA level in 33 different tumors was derived from the UCSC database (https://xenabrowser.net/): TCGA, TARGET and GTEx (PANCAN, N=19131, G=60499). The ANLN and USP10 mRNA expression of 15 ESCC patients was obtained from the SRP064894 dataset in the SRA (Sequence Read Archive) database (<https://www.ncbi.nlm.nih.gov/sra/>) [[20](#_ENREF_20)]. The expression matrix and clinical information of ANLN and USP10 were obtained from the GSE53625 dataset in the GEO (Gene Expression Omnibus) database (<https://www.ncbi.nlm.nih.gov/geo/>) [[19](#_ENREF_19), [44](#_ENREF_44)]. Proteomics data of ANLN and USP10 were obtained from the iProX database (IPX0002501000). To reveal the effect of F806 on ESCC cells, we analyzed the proteomic data of ESCC cells treated with F806, and selected the proteins that were significantly down regulated (SILAC ratio H/L normalized < 0.75, Supplementary Data 3). Then, the highly expressed proteins were screened from the proteomic data of ESCC tissues (Top 2% of T/N, IPX0002501000, Supplementary Data 1), and these proteins were overlapped with F806 down-regulated proteins (Supplementary Figure 3B). Similarly, we repeated the above analysis with highly expressed proteins from 9 ESCC cell lines (normalized ratio>2.5, Supplementary Data 4) (Supplementary Figure 3D). To screen ANLN DUBs suppressed by F806, we selected the DUBs with the most significant inhibition from the proteomic data of ESCC cells treated by F806 (Supplementary Table 3, Supplementary Data 3), and then selected the DUB with the highest score from the ANLN-interacting proteins (Supplementary Table 2, Supplementary Data 2). Overlapping the data, it was found that USP10 was a potential F806 target (Supplementary Figure 3K).

**Statistical analysis**

Each experiment was repeated independently at least three times. Data are expressed as mean ± standard deviation. Statistical analysis of data obtained during the study was conducted using SPSS software (version 13.0; SPSS, Inc.). The Pearson’s or Spearman’s correlation coefficient was used for evaluating the relationship between the two groups. Unpaired two-tailed Student’s *t*-tests and one-way analyses of variance were performed using GraphPad Prism 7 software (GraphPad Software, Inc.), and differences in means were considered statistically significant at *P*< 0.05.

**Data availability**

The data that support the findings of this study—including clinical information, and proteome data—are available within the paper and its Supplementary Information. ANLN mRNA expression in different tumors was obtained from the UCSC database (https://xenabrowser.net/). The ANLN and USP10 mRNA expression of 15 ESCC patients was obtained from the SRA database (accession number SRP064894). The expression matrix and clinical information of ANLN and USP10 were obtained from the GEO database (accession number GSE53625). The raw files of 124 ESCC proteome datasets can be obtained from iProX database (accession number IPX0002501000). Other proteome datasets can be obtained from Supplementary data.

**References**

1. Batty P, Gerlich DW. Mitotic Chromosome Mechanics: How Cells Segregate Their Genome. *Trends Cell Biol* **29**, 717-726 (2019)

2. Perez de Castro I, de Carcer G, Montoya G, Malumbres M. Emerging cancer therapeutic opportunities by inhibiting mitotic kinases. *Curr Opin Pharmacol* **8**, 375-383 (2008)

3. Henriques AC, Ribeiro D, Pedrosa J, Sarmento B, Silva PMA, Bousbaa H. Mitosis inhibitors in anticancer therapy: When blocking the exit becomes a solution. *Cancer Lett* **440-441**, 64-81 (2019)

4. Yan VC, Butterfield HE, Poral AH, Yan MJ, Yang KL, Pham CD *et al*. Why Great Mitotic Inhibitors Make Poor Cancer Drugs. *Trends Cancer* **6**, 924-941 (2020)

5. Sivakumar S, Gorbsky GJ. Spatiotemporal regulation of the anaphase-promoting complex in mitosis. *Nat Rev Mol Cell Biol* **16**, 82-94 (2015)

6. Hoeller D, Hecker CM, Dikic I. Ubiquitin and ubiquitin-like proteins in cancer pathogenesis. *Nat Rev Cancer* **6**, 776-788 (2006)

7. Lara-Gonzalez P, Westhorpe FG, Taylor SS. The spindle assembly checkpoint. *Curr Biol* **22**, R966-980 (2012)

8. Schrock MS, Stromberg BR, Scarberry L, Summers MK. APC/C ubiquitin ligase: Functions and mechanisms in tumorigenesis. *Semin Cancer Biol* **67**, 80-91 (2020)

9. Xie. Q, Wu. Q. CDC20 maintains tumor initiating cells. *Oncotarget* **6**, 13241-13254 (2015)

10. Amador V, Ge S, Santamaria PG, Guardavaccaro D, Pagano M. APC/C(Cdc20) controls the ubiquitin-mediated degradation of p21 in prometaphase. *Mol Cell* **27**, 462-473 (2007)

11. Komander D, Clague MJ, Urbe S. Breaking the chains: structure and function of the deubiquitinases. *Nat Rev Mol Cell Biol* **10**, 550-563 (2009)

12. He M, Zhou Z, Wu G, Chen Q, Wan Y. Emerging role of DUBs in tumor metastasis and apoptosis: Therapeutic implication. *Pharmacol Ther* **177**, 96-107 (2017)

13. Fraile JM, Quesada V, Rodriguez D, Freije JM, Lopez-Otin C. Deubiquitinases in cancer: new functions and therapeutic options. *Oncogene* **31**, 2373-2388 (2012)

14. Zhang L, Maddox AS. Anillin. *Curr Biol* **20**, R135-136 (2010)

15. Piekny AJ, Maddox AS. The myriad roles of Anillin during cytokinesis. *Semin Cell Dev Biol* **21**, 881-891 (2010)

16. Suzuki C, Daigo Y, Ishikawa N, Kato T, Hayama S, Ito T *et al*. ANLN plays a critical role in human lung carcinogenesis through the activation of RHOA and by involvement in the phosphoinositide 3-kinase/AKT pathway. *Cancer Res* **65**, 11314-11325 (2005)

17. Zhao WM, Fang G. Anillin is a substrate of anaphase-promoting complex/cyclosome (APC/C) that controls spatial contractility of myosin during late cytokinesis. *J Biol Chem* **280**, 33516-33524 (2005)

18. D'Avino PP. How to scaffold the contractile ring for a safe cytokinesis - lessons from Anillin-related proteins. *J Cell Sci* **122**, 1071-1079 (2009)

19. Guo JC, Xie YM, Ran LQ, Cao HH, Sun C, Wu JY *et al*. L1CAM drives oncogenicity in esophageal squamous cell carcinoma by stimulation of ezrin transcription. *J Mol Med (Berl)* **95**, 1355-1368 (2017)

20. Chen. Y, Liao. L-D, Wu. Z-Y. Identification of key genes by integrating DNA methylation and nextgeneration transcriptome sequencing for esophageal squamous cell carcinoma. **12**, 1332-1365 (2020)

21. Liu W, Xie L, He Y, Wu Z, Liu L, Bai X *et al*. Large-scale and high-resolution mass spectrometry-based proteomics profiling defines molecular subtypes of esophageal cancer for therapeutic targeting. *Nature communications* **12**, 4961 (2021)

22. Li X, Yuan J, Song C, Lei Y, Xu J, Zhang G *et al*. Deubiquitinase USP39 and E3 ligase TRIM26 balance the level of ZEB1 ubiquitination and thereby determine the progression of hepatocellular carcinoma. *Cell Death Differ* **28**, 2315-2332 (2021)

23. Nicklas S, Hillje AL, Okawa S, Rudolph IM, Collmann FM, van Wuellen T *et al*. A complex of the ubiquitin ligase TRIM32 and the deubiquitinase USP7 balances the level of c-Myc ubiquitination and thereby determines neural stem cell fate specification. *Cell Death Differ* **26**, 728-740 (2019)

24. Liu J, Xia H, Kim M, Xu L, Li Y, Zhang L *et al*. Beclin1 controls the levels of p53 by regulating the deubiquitination activity of USP10 and USP13. *Cell* **147**, 223-234 (2011)

25. Li LY, Zhang K, Jiang H, Xie YM, Liao LD, Chen B *et al*. Quantitative proteomics reveals the downregulation of GRB2 as a prominent node of F806-targeted cell proliferation network. *J Proteomics* **117**, 145-155 (2015)

26. Li. L-Y, Jiang. H. Macrolide analog F806 suppresses esophageal squamous cell carcinoma (ESCC) by blocking β1 integrin activation. *Oncotarget* **6**, 15940-15952 (2015)

27. Zhang S, Nguyen LH, Zhou K, Tu HC, Sehgal A, Nassour I *et al*. Knockdown of Anillin Actin Binding Protein Blocks Cytokinesis in Hepatocytes and Reduces Liver Tumor Development in Mice Without Affecting Regeneration. *Gastroenterology* **154**, 1421-1434 (2018)

28. Liu Y, Liu X, Zhang N, Yin M, Dong J, Zeng Q *et al*. Berberine diminishes cancer cell PD-L1 expression and facilitates antitumor immunity via inhibiting the deubiquitination activity of CSN5. *Acta Pharm Sin B* **10**, 2299-2312 (2020)

29. Lagergren J, Smyth E, Cunningham D, Lagergren P. Oesophageal cancer. *The Lancet* **390**, 2383-2396 (2017)

30. Xie L, Li LY, Zheng D, Xie YM, Xu XE, Tao LH *et al*. F806 Suppresses the Invasion and Metastasis of Esophageal Squamous Cell Carcinoma via Downregulating F-Actin Assembly-Related Rho Family Proteins. *Biomed Res Int* **2018**, 2049313 (2018)

31. Zhu H, Yan F, Yuan T, Qian M, Zhou T, Dai X *et al*. USP10 Promotes Proliferation of Hepatocellular Carcinoma by Deubiquitinating and Stabilizing YAP/TAZ. *Cancer Res* **80**, 2204-2216 (2020)

32. Yuan J, Luo K, Zhang L, Cheville JC, Lou Z. USP10 regulates p53 localization and stability by deubiquitinating p53. *Cell* **140**, 384-396 (2010)

33. Weisberg EL, Schauer NJ, Yang J, Lamberto I, Doherty L, Bhatt S *et al*. Inhibition of USP10 induces degradation of oncogenic FLT3. *Nat Chem Biol* **13**, 1207-1215 (2017)

34. Wang X, Xia S, Li H, Wang X, Li C, Chao Y *et al*. The deubiquitinase USP10 regulates KLF4 stability and suppresses lung tumorigenesis. *Cell Death Differ* **27**, 1747-1764 (2020)

35. Pollard TD, O'Shaughnessy B. Molecular Mechanism of Cytokinesis. *Annu Rev Biochem* **88**, 661-689 (2019)

36. Kim H, Johnson JM, Lera RF, Brahma S, Burkard ME. Anillin Phosphorylation Controls Timely Membrane Association and Successful Cytokinesis. *PLoS Genet* **13**, e1006511 (2017)

37. Hunter T. The age of crosstalk: phosphorylation, ubiquitination, and beyond. *Mol Cell* **28**, 730-738 (2007)

38. Swaney DL, Beltrao P, Starita L, Guo A, Rush J, Fields S *et al*. Global analysis of phosphorylation and ubiquitylation cross-talk in protein degradation. *Nat Methods* **10**, 676-682 (2013)

39. Dietachmayr M, Rathakrishnan A, Karpiuk O, von Zweydorf F, Engleitner T, Fernandez-Saiz V *et al*. Antagonistic activities of CDC14B and CDK1 on USP9X regulate WT1-dependent mitotic transcription and survival. *Nat Commun* **11**, 1268 (2020)

40. Zhang Q, Zhang ZY, Du H, Li SZ, Tu R, Jia YF *et al*. DUB3 deubiquitinates and stabilizes NRF2 in chemotherapy resistance of colorectal cancer. *Cell Death Differ* **26**, 2300-2313 (2019)

41. Jochemsen AG, Shiloh Y. USP10: Friend and Foe. *Cell* **140**, 308-310 (2010)

42. Sun XX, He X, Yin L, Komada M, Sears RC, Dai MS. The nucleolar ubiquitin-specific protease USP36 deubiquitinates and stabilizes c-Myc. *Proc Natl Acad Sci U S A* **112**, 3734-3739 (2015)

43. Kim D, Hong A, Park HI, Shin WH, Yoo L, Jeon SJ *et al*. Deubiquitinating enzyme USP22 positively regulates c-Myc stability and tumorigenic activity in mammalian and breast cancer cells. *J Cell Physiol* **232**, 3664-3676 (2017)

44. Zhan XH, Jiao JW, Zhang HF, Li CQ, Zhao JM, Liao LD *et al*. A three-gene signature from protein-protein interaction network of LOXL2- and actin-related proteins for esophageal squamous cell carcinoma prognosis. *Cancer Med* **6**, 1707-1719 (2017)

45. Zhan XH, Jiao JW, Zhang HF, Xu XE, He JZ, Li RL *et al*. LOXL2 Upregulates Phosphorylation of Ezrin to Promote Cytoskeletal Reorganization and Tumor Cell Invasion. *Cancer Res* **79**, 4951-4964 (2019)

46. Xu QX, Li EM, Zhang YF, Liao LD, Xu XE, Wu ZY *et al*. Overexpression of sigma1 receptor and its positive associations with pathologic TNM classification in esophageal squamous cell carcinoma. *J Histochem Cytochem* **60**, 457-466 (2012)

47. Zhang XD, Huang GW, Xie YH, He JZ, Guo JC, Xu XE *et al*. The interaction of lncRNA EZR-AS1 with SMYD3 maintains overexpression of EZR in ESCC cells. *Nucleic Acids Res* **46**, 1793-1809 (2018)

48. Lu XF, Li EM, Du ZP, Xie JJ, Guo ZY, Gao SY *et al*. Specificity protein 1 regulates fascin expression in esophageal squamous cell carcinoma as the result of the epidermal growth factor/extracellular signal-regulated kinase signaling pathway activation. *Cell Mol Life Sci* **67**, 3313-3329 (2010)

49. Li F, Li Y, Liang H, Xu T, Kong Y, Huang M *et al*. HECTD3 mediates TRAF3 polyubiquitination and type I interferon induction during bacterial infection. *J Clin Invest* **128**, 4148-4162 (2018)

50. Peng L, Guo JC, Long L, Pan F, Zhao JM, Xu LY *et al*. A Novel Clinical Six-Flavoprotein-Gene Signature Predicts Prognosis in Esophageal Squamous Cell Carcinoma. *Biomed Res Int* **2019**, 3869825 (2019)

51. Long L, Pang XX, Lei F, Zhang JS, Wang W, Liao LD *et al*. SLC52A3 expression is activated by NF-kappaB p65/Rel-B and serves as a prognostic biomarker in esophageal cancer. *Cell Mol Life Sci* **75**, 2643-2661 (2018)

52. Andersen ND, Srinivas S, Pinero G, Monje PV. A rapid and versatile method for the isolation, purification and cryogenic storage of Schwann cells from adult rodent nerves. *Sci Rep* **6**, 31781 (2016)

53. Ma L, Zhou Y, Khan MAS, Yasuhara S, Martyn JAJ. Burn-Induced Microglia Activation is Associated With Motor Neuron Degeneration and Muscle Wasting in Mice. *Shock* **51**, 569-579 (2019)

54. Zheng ZY, Yang PL, Luo W, Yu SX, Xu HY, Huang Y *et al*. STAT3beta Enhances Sensitivity to Concurrent Chemoradiotherapy by Inducing Cellular Necroptosis in Esophageal Squamous Cell Carcinoma. *Cancers (Basel)* **13**, (2021)

55. Chuang C, Lin SH, Huang F, Pan J, Josic D, Yu-Lee LY. Acetylation of RNA processing proteins and cell cycle proteins in mitosis. *J Proteome Res* **9**, 4554-4564 (2010)

56. Fang WK, Liao LD, Gu W, Chen B, Wu ZY, Wu JY *et al*. Down-regulated gamma-catenin expression is associated with tumor aggressiveness in esophageal cancer. *World J Gastroenterol* **20**, 5839-5848 (2014)

57. Zeng RJ, Zheng CW, Gu JE, Zhang HX, Xie L, Xu LY *et al*. RAC1 inhibition reverses cisplatin resistance in esophageal squamous cell carcinoma and induces downregulation of glycolytic enzymes. *Mol Oncol* **13**, 2010-2030 (2019)

58. Inuzuka H, Gao D, Finley LW, Yang W, Wan L, Fukushima H *et al*. Acetylation-dependent regulation of Skp2 function. *Cell* **150**, 179-193 (2012)

59. Zhang J, Chen Y, Chen X, Zhang W, Zhao L, Weng L *et al*. Deubiquitinase USP35 restrains STING-mediated interferon signaling in ovarian cancer. *Cell Death Differ* **28**, 139-155 (2021)

60. Cheng YW, Zeng FM, Li DJ, Wang SH, He JZ, Guo ZC *et al*. P300/CBP-associated factor (PCAF)-mediated acetylation of Fascin at lysine 471 inhibits its actin-bundling activity and tumor metastasis in esophageal cancer. *Cancer Commun (Lond)* **41**, 1398-1416 (2021)

61. Schretter CE, Vielmetter J, Bartos I, Marka Z, Marka S, Argade S *et al*. A gut microbial factor modulates locomotor behaviour in Drosophila. *Nature* **563**, 402-406 (2018)

62. Wang X, Mazurkiewicz M, Hillert EK, Olofsson MH, Pierrou S, Hillertz P *et al*. The proteasome deubiquitinase inhibitor VLX1570 shows selectivity for ubiquitin-specific protease-14 and induces apoptosis of multiple myeloma cells. *Sci Rep* **6**, 26979 (2016)

63. Davies CW, Vidal SE, Phu L, Sudhamsu J, Hinkle TB, Chan Rosenberg S *et al*. Antibody toolkit reveals N-terminally ubiquitinated substrates of UBE2W. *Nat Commun* **12**, 4608 (2021)

**ACKNOWLEDGEMENTS**

We thank Dr. Stanley Li Lin, Department of Cell Biology and Genetics, Shantou University Medical College, to edit the language of the manuscript. We thank Professor Qingsong Qin, Laboratory of Human Virology and Oncology, Shantou University Medical College and Professor Fenfei Gao, Departmentof Pharmacology, Shantou University Medical College for technical support in Ub-AMC. We are indebted to Dr. Yaohui He from the Fujian Provincial Key Laboratory of Innovative Drug Target Research, School of Pharmaceutical Sciences, Xiamen University for technical support in proteomics. We thank Professor Ceshi Chen (Chinese Academy of Sciences, Kunming Institute of Zoology, China) for providing ubiquitin mutants.

**AUTHOR CONTRIBUTIONS**

LYX and EML conceived and designed the project. YFC and LX wrote the manuscript and performed most of the experiments. SHW and ZYW provided clinical samples. Immunohistochemistry for ANLN and USP10 was performed by XEX. MYC, YQZ, ZML, JZH and BBT generated the immunohistochemistry data and labelled the image. WQS, BBT and DXD performed bioinformatics analyses. BBT and WQS constructed majority of the plasmids. XL and LYL performed the animal experiments. LDL contributed to cell culture. LYX, EML, and YWC revised the manuscript, and all authors read and approved the final manuscript.

**FUNDING**

This work was supported by the National Natural Science Foundation of China (81602630, 81872372, 81902469 and 82173034), the Innovative Team Grant of Guangdong Department of Education (2021KCXTD005), the Educational Commission of Guangdong Province (No.2017KQNCX070), the Special Fund for Science and Technology Innovation Strategy of Guangdong Province (No. 2018A030310220) and 2020 Li Ka Shing Foundation Cross-Disciplinary Research Grant (2020LKSFG07B).

**COMPETING INTERESTS**

The authors declare no competing interests.

**Ethical approval**

Human tissue acquisition and subsequent use were approved by the Medical Ethics Committee of Shantou Central Hospital, and informed consent was obtained from patients/family members.

**Figure Legends**

**Fig. 1 Highly expressed ANLN participates in ESCC malignant progression by promoting cytokinesis and proliferation.**

**A** Representative image of ANLN immunohistochemical staining in ESCC and normal tissues (scale 200 μm and 50 μm). **B** ANLN expression in 104 ESCC tissues and normal tissues. **C** Kaplan-Meier curve analysis of the correlation between ANLN protein expression and overall survival of 104 ESCC patients. **D** Western blot analysis of ANLN knockdown by different siRNAs in KYSE150 and KYSE510 cells. **E-F** Effect of ANLN on ESCC cell proliferation was determined by colony formation (**E**) and an xCELLigence Real-Time Cell Analyzer (RTCA) system (**F**). **G** Cells after ANLN knockdown for 60 h were analyzed by flow cytometry. **H** KYSE150 cells were transfected with control or ANLN siRNA for 48 h. ANLN and cell-cycle proteins were detected using western blotting. **I** KYSE150 cells stably expressing HA-vector or HA-ANLN was transfected with an ANLN 3'UTR siRNA pool, then treated with thymidine for 24 h, and released for 12 h to obtain cytokinesis cells. Cells were fixed and detected by immunofluorescence. **J** Mean intensity ratio of individual cells were plotted (F-Actin fluorescence at the equatorial cortex: pole). *P*<0.0001 by Student’s t-test. Left: diagram showing locations of regions of calculation. **K-L** KYSE150 cells stably expressing HA-vector or HA-ANLN were transfected with an ANLN 3'UTR siRNA pool, then cyclin B1 and E2 expression was detected by western blotting, and cell proliferation was detected by colony formation. All data are representative of at least three independent experiments and the results were statistically analyzed using a *t*-test.

**Fig. 2 USP10 interacts with ANLN and positively regulates ANLN protein level.**

**A** Flow chart of an in vivo screen to identify candidates that interact with ANLN in KYSE150 cells. **B** Potential ANLN-interacting DUBs were identified by mass spectrometry analysis. **C** The indicated plasmids were transfected into HEK293T cells for 48 hours, and then harvested with EBC buffer for co-immunoprecipitation. **D-E** Interaction of endogenous ANLN and USP10 in KYSE150 and KYSE30 cells was detected by co-immunoprecipitation. **F-G** Schematic illustration of USP10 structure (**F**). The indicated plasmids were transfected into HEK293T cells for 48h, then cells were lysed and subjected to immunoprecipitation with HA magnetic beads (Thermo Fisher Scientific, 88837) (**G**). **H-I** Schematic illustration of ANLN structure (**H**). The indicated plasmids were transfected into HEK293T cells for 48h, then cells were lysed and subjected to immunoprecipitation with HA magnetic beads (**I**). **J** The interaction between recombinant GST-USP10 and His-ANLN mutants was examined using an in vitro GST pull down assay. **K-L** ESCC cells were treated with different concentrations of spautin-1 (MedChemExpress, HY-12990) or HBX19818 (MedChemExpress, HY-17540) for 24 h, and then western blotting was used to detect the protein levels of ANLN and USP10. **M** KYSE150 and KYSE510 cells were transfected with siRNAs for 48 h. The indicated antibodies were used for the western blotting. **N** KYSE150 cells stably expressing HA-vector, HA-USP10 or HA-USP10-CA were transfected with USP10 3' UTR siRNA, and the indicated antibodies were used for the western blotting. All data are representative of at least three independent experiments.

**Fig. 3 USP10 removes K11- and K63-linked ubiquitin chains of ANLN through its deubiquitinase activity.**

**A** KYSE150 cells were transfected with siRNAs for 24 h and then treated with cycloheximide (CHX) (10 μg/ml) for different times. ANLN protein level was detected by western blotting. **B** KYSE150 cells stably expressing HA-vector or HA-USP10 were treated with CHX (10 μg/ml) for different times. ANLN protein level was detected by western blotting. **C** KYSE150 cells transfected with siRNAs were treated with MG132 (20 μM) for 10 h before harvesting, and ANLN protein levels were detected by western blotting. **D** KYSE150 cells transfected with siRNAs and HA-Ub were treated with MG132 (20 μM) for 10 h before harvesting, and the ubiquitination level of endogenous ANLN was measured using an ubiquitination assay. **E** HEK293T cells were co-transfected with Flag-ANLN, HA-Ub, USP10-WT, or CA, and treated with MG132 (20 μM) for 8 h before harvesting. Ubiquitination assays were used to detect the ubiquitination level of Flag-ANLN. **F** HEK293T cells were transfected with the indicated plasmids and treated with MG132 (20 μM) for 8 h before harvesting. An ubiquitination assay was used to detect the ubiquitination level of Flag-ANLN. All data are representative of at least three independent experiments.

**Fig. 4 USP10 promotes contractile ring localization and cytokinesis by stabilizing ANLN protein.**

**A** KYSE150 cells transfected with siRNAs were synchronized to different time periods by thymidine. ANLN and USP10 levels were detected by western blotting. Cyclin B1, E2, and phospho-histone H3 (S10) were used as markers for the different cell-cycle phases. **B** KYSE150 cells were subjected to DTB and released for different time periods. Interaction between ANLN and USP10 was detected by immunoprecipitation. Cyclin B1 and E2 were used as markers for the different cell-cycle phases. **C** The localization of ANLN and USP10 was detected by immunofluorescence in synchronized KYSE150 cells. **D** KYSE150 cells transfected with siRNAs were synchronized in G2/M phase, released for 3 h, and analyzed by flow cytometry. **E** KYSE150 and KYSE30 cells were transfected with siRNAs for 48 h. The indicated antibodies were used for western blotting. **F** KYSE150 cells stably expressing HA-vector or HA-ANLN was transfected with a USP10 siRNA pool, then treated with thymidine for 24 h, and released for 12 h to obtain cytokinesis cells. Cells were fixed and detected by immunofluorescence. **G** Mean intensity ratio of individual cells were plotted (F-Actin fluorescence at the equatorial cortex: pole). *P*<0.0001 by Student’s t-test. **H** KYSE150 cells stably expressing HA-vector or HA-ANLN were transfected with a USP10 siRNA pool. The indicated antibodies were used for western blotting. All data are representative of at least three independent experiments and the results were statistically analyzed using a t-test.

**Fig. 5 USP10 and Cdh1 form a complex with ANLN in a non-competitive manner to balance ANLN levels.**

**A-C** The indicated plasmids were transfected into HEK293T cells for 48 h, then cells were harvested with EBC buffer and co-immunoprecipitated using HA or Flag magnetic beads. **D** The interaction between the recombinant GST-Cdh1 and His-ANLN or His-USP10 was examined using an in vitro GST pull down assay. **E-G** KYSE150 cells were transfected with siRNAs for 40 h, and then treated with MG132 (20 μM) for 8 h. Immunoprecipitation was used to detect the interaction between endogenous USP10, Cdh1, and ANLN. **H** HEK293T cells were transfected with the indicated plasmids and treated with MG132 (20 μM) for 8 h before harvesting. Ubiquitination assay was used to detect the ubiquitination level of Flag-ANLN. **I** KYSE150 cells were transfected with siRNAs for 48 h. The indicated antibodies were used for the western blotting. **J** KYSE150 cells were treated with DTB and released for different time periods. Interaction between endogenous ANLN and USP10 or Cdh1 was detected by immunoprecipitation. Cyclin B1, E2, and phospho-histone H3 (S10) were used as markers for the different cell-cycle phases. **K** KYSE150 cells transfected with siRNAs were synchronized and released for different time periods. ANLN levels were detected by western blotting. Cyclin B1 and phospho-histone H3 (S10) were used as markers for mitosis. **L-N** KYSE150 cells were transfected with siRNAs for 48 h. The indicated antibodies were used for the western blotting. All data are representative of at least three independent experiments.

**Fig. 6 FIM-04-806 induces ANLN degradation by inhibiting USP10 deubiquitinase activity.**

**A** Effect of F806 on ANLN expression was evaluated in xenografts (KYSE510 cells) from tumor-bearing mice. After F806 (4 mg/kg) treatment for 21 days, the level of ANLN protein in xenografts was detected by immunohistochemistry. **B** Cells were treated with F806 (10 μM) for 15 h and then co-treated with MG132 (10 μM) for 6 h, ANLN levels were detected by western blotting. **C** Cells were co-treated with cycloheximide (CHX) (10 μg/ml) and F806 (10 μM), and ANLN levels were examined by western blotting. **D** KYSE150 cells transfected with HA-Ub were treated with F806 (10 μM) for different times and then treated with MG132 (20 μM) for 8 h before harvest. Ubiquitination assays were performed to examine the ubiquitination level of ANLN. **E** KYSE150 cells transfected with Flag-vector or Flag-USP10 were treated with F806 (10 μM), and ANLN levels were detected by western blotting. **F** KYSE150 cells transfected with HA-USP10 or HA-USP10-CA were treated with F806 (10 μM), and ANLN levels were detected by western blotting. **G** KYSE150 cells were transfected with USP10 or control siRNA for 24 h and then treated with F806 (10 μM) for 16 h, and ANLN levels were detected by western blotting. **H** HEK293T cells were transfected with the indicated plasmids for 24 h, then treated with MG132 (10 μM) with or without F806 (10 μM) for 6 h. Subsequently, cells were harvested for ubiquitination assay. **I** KYSE150 cells were treated with F806 (10 μM) for 24 h. Cell lysates were subsequently labeled with HA-Ub-VS for 30 min. The indicated antibodies were used for the western blotting. **J** Purified USP10 protein was incubated with F806 at 25℃ for 2 hours in vitro, and then labeled with HA-Ub-VS for 30 min. The indicated antibodies were used for the western blotting. **K-L** USP10 purified from prokaryotic cells and different concentrations of F806 were mixed at 25°C for 2 h. Ub-AMC was then added to each well and further incubated at 37°C for 1 h. Ub-AMC hydrolysis was measured in real time (**K**), and the biochemical IC50 of F806 was measured using GraphPad Prism 7 (**L**). All data are representative of at least three independent experiments and the results were statistically analyzed using a t-test.

**Fig. 7 FIM-04-806 alters the balance of the USP10-ANLN-Cdh1 complex by targeting USP10 and inhibits the M/G1 transition of ESCC cells.**

**A** The binding between F806 and USP10 was analyzed by SPR. His-USP10 protein was immobilized on an activated CM5 sensor chip, F806 solution was then flowed across the chip. **B** KYSE150 cells were transfected with Cdh1 or control siRNA for 24 h and then treated with F806 (10 μM) for 12 h, and ANLN level was detected by western blotting. **C** KYSE150 cells were treated with F806 (10 μM) for different times and MG132 (20 μM) for 8 h before harvest. The interaction between ANLN and Cdh1 or USP10 was detected by immunoprecipitation. **D** KYSE150 cells were synchronized in M phase and released for different time periods with DMSO or F806 (10 μM). The interaction between ANLN and USP10 or Cdh1 was detected by immunoprecipitation. **E** Cells were treated with different concentrations of F806 for 16 h. The indicated antibodies were used for the western blotting. **F-G** Experimental protocol for DTB. KYSE150 cells were synchronized by DTB and released for different time periods. F806 (10 μM) was added at 9 h after release. The samples were analyzed by flow cytometry (**F**) and immunofluorescence (**G**). **H** Cells 9 h after DTB release were treated with or without F806 (10 μM) for 3 h. The contractile ring localization of ANLN was also determined. Nine fields were counted for each group. **I** Cells after 9 h of release were treated with or without F806 (10 μM) for 6 h. The nuclear localization of ANLN was determined. Five fields were counted in each group. All data are representative of at least three independent experiments and the results were statistically analyzed using a *t*-test. **J** Model: USP10 and Cdh1 form a functional complex with ANLN in a non-competitive manner to regulate ANLN abundance. The steady state of ANLN levels promotes contractile ring assembly and cytokinesis. Moreover, F806 selectively targets USP10 to alter the ubiquitination-deubiquitination balance of ANLN. Reduction of ANLN leads to a slowdown in the turnover of contractile ring components and a delay in M phase.

**Fig. 8 Expression of USP10 is positively correlated with ANLN level and poor prognosis of ESCC patients.**

**A** Representative images of USP10 immunohistochemical staining of ESCC and normal tissues (scale 200 μm and 50 μm). **B** Expression analysis of USP10 in 104 ESCC and normal tissue samples. **C** USP10 protein levels in 124 ESCC and normal tissues were analyzed based on the IPX0002501000 dataset from the iProX database. **D-E** The mRNA levels of USP10 in tumor and adjacent normal tissues of ESCC patients were analyzed based on the GeneChip data of GSE53625 from the GEO database (**D**). The mRNA levels of USP10 in ESCC tumors and adjacent normal tissues were analyzed based on the SRP064894 dataset in the SRA database (**E**). **F-I** The relation between USP10 and ANLN was determined using Pearson’s correlation analysis. **J-L** Kaplan-Meier curve analysis of the correlation between USP10 protein expression and overall survival in ESCC patients.
