# Supplementary material for: Targeting USP10 induces degradation of oncogenic ANLN in esophageal squamous cell carcinoma
Source: Cell Death Differ. 2022 Dec 16;30(2):527–43. doi: 10.1038/s41418-022-01104-x (PMC9950447; doi:10.1038/s41418-022-01104-x)
Supplement: Supplementary file 10 — Source data for western boltting [file 41418_2022_1104_MOESM10_ESM.pdf]

Fig.1D

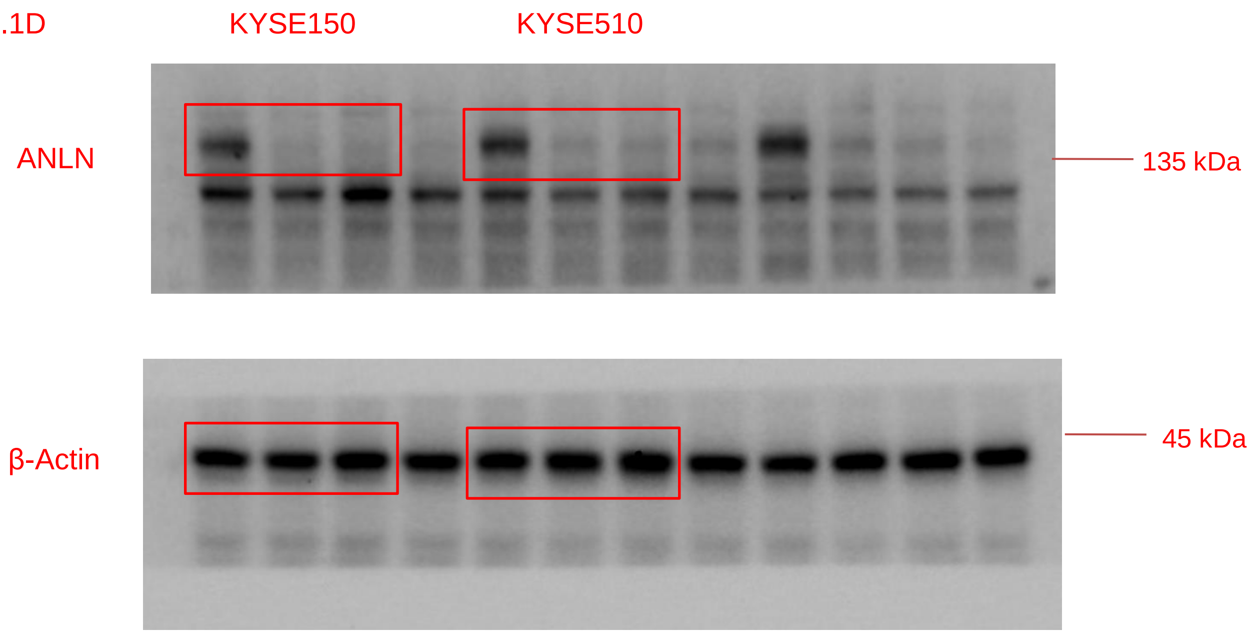
KYSE150
KYSE510
ANLN
135 kDa
β-Actin
45 kDa

Fig.1H

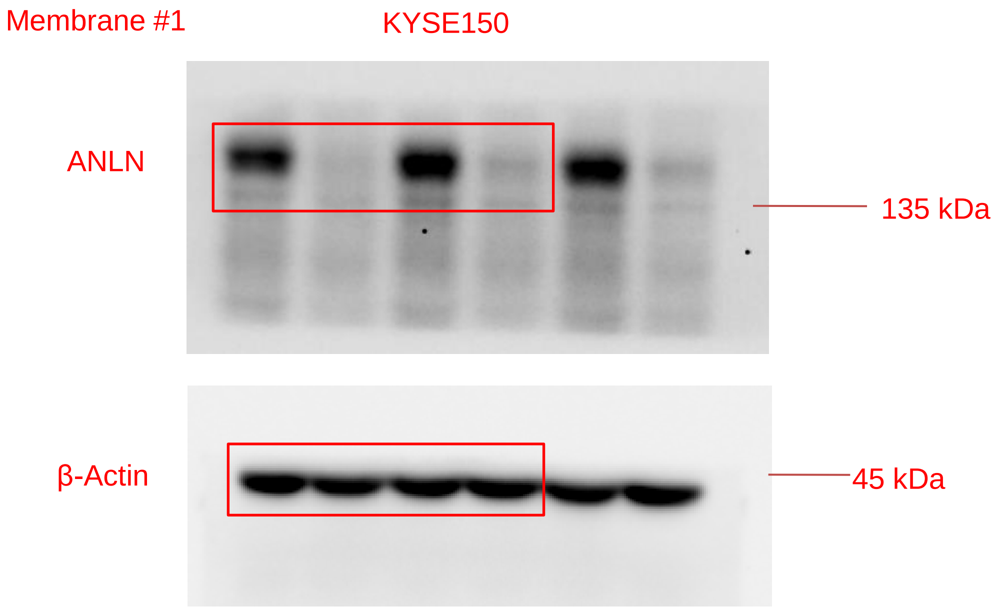
Membrane #1
KYSE150
ANLN
135 kDa
β-Actin
45 kDa

Fig.1H

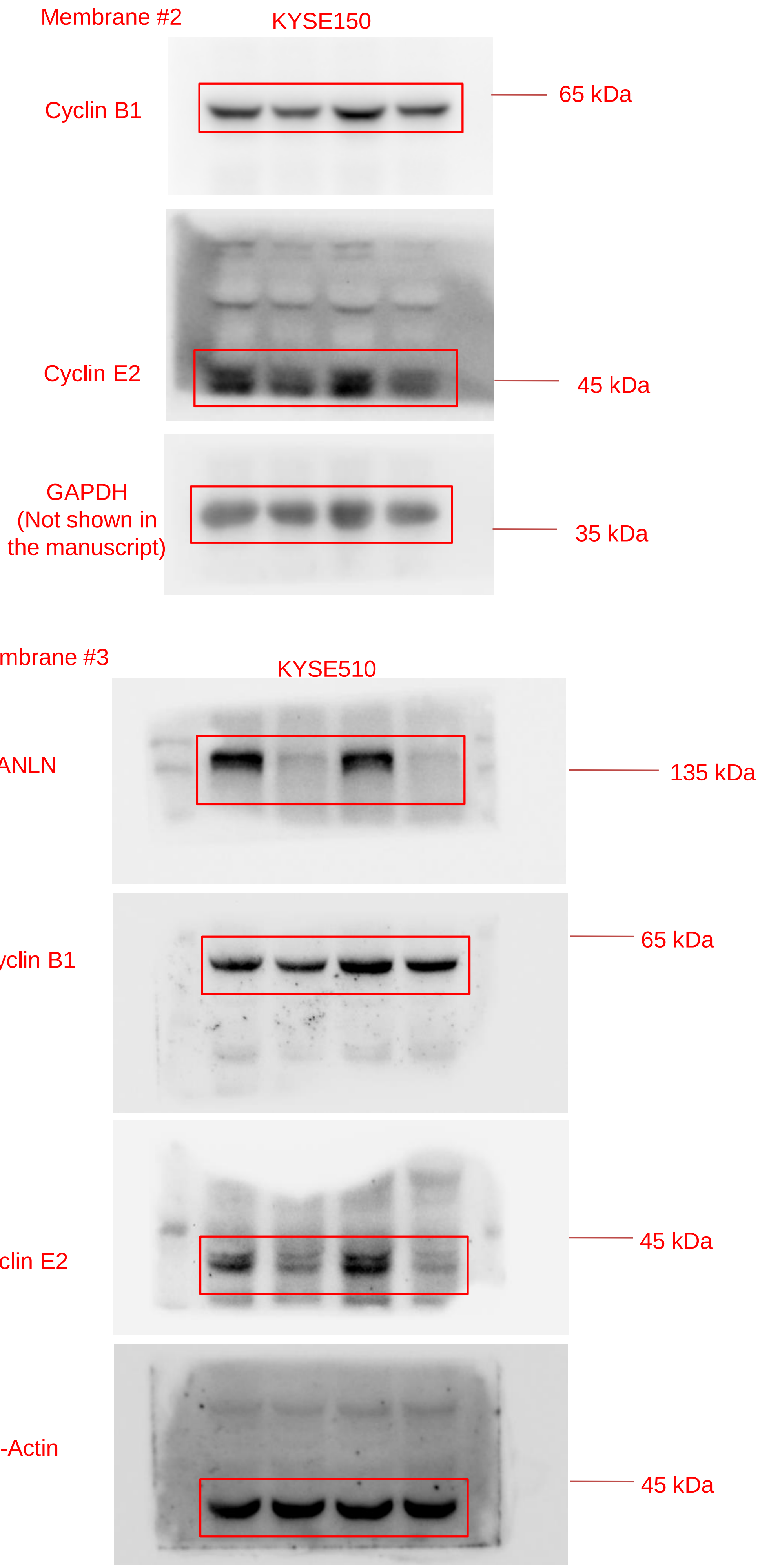
Membrane #2
KYSE150
Cyclin B1
65 kDa
Cyclin E2
45 kDa
GAPDH
(Not shown in
the manuscript)
35 kDa
Membrane #3
KYSE510
ANLN
135 kDa
Cyclin B1
65 kDa
Cyclin E2
45 kDa
β-Actin
45 kDa

Fig.1K

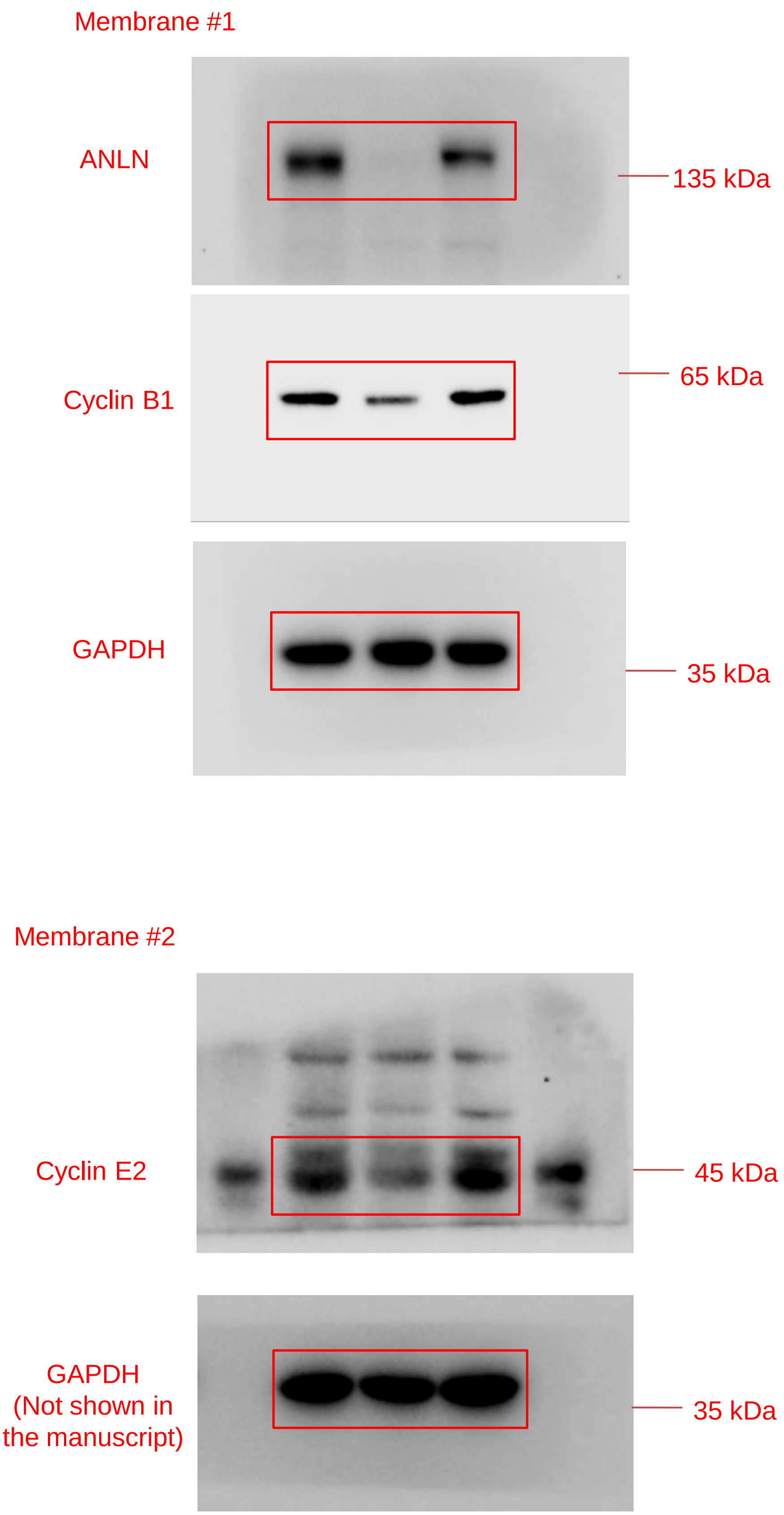

Membrane #1
ANLN
135 kDa
Cyclin B1
65 kDa
GAPDH
35 kDa
Membrane #2
Cyclin E2
45 kDa
GAPDH
(Not shown in
the manuscript)
35 kDa

Fig.2C

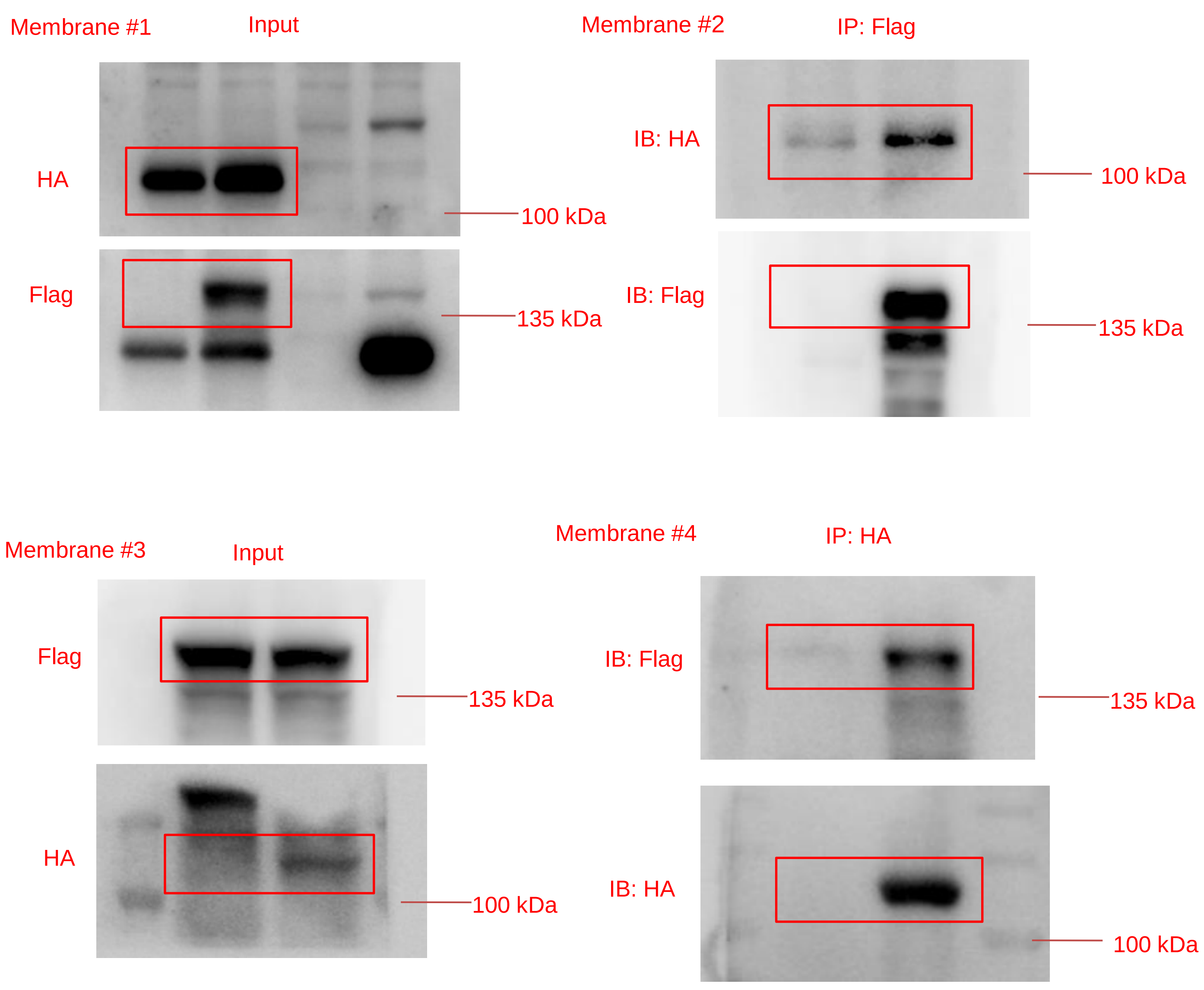
Membrane #1
Input
HA
100 kDa
Flag
135 kDa
Membrane #2
IP: Flag
IB: HA
100 kDa
IB: Flag
135 kDa
Membrane #3
Input
Flag
135 kDa
HA
100 kDa
Membrane #4
IP: HA
IB: Flag
135 kDa
IB: HA
100 kDa

Fig.2D

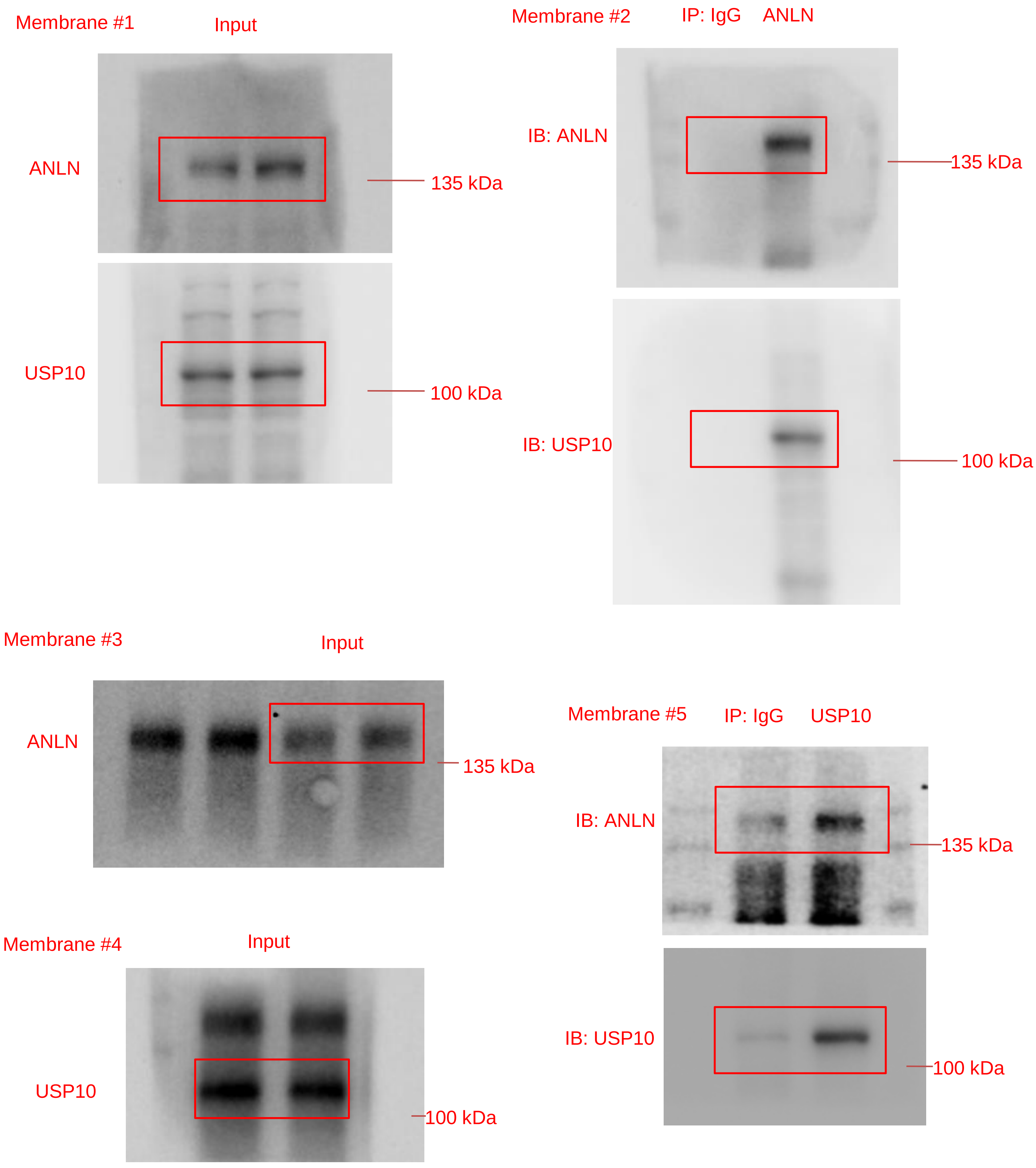
Membrane #1
Input
ANLN
135 kDa
USP10
100 kDa
Membrane #2
IP: IgG
ANLN
IB: ANLN
135 kDa
IB: USP10
100 kDa
Membrane #3
Input
ANLN
135 kDa
Membrane #4
Input
USP10
100 kDa
Membrane #5
IP: IgG
USP10
IB: ANLN
135 kDa
IB: USP10
100 kDa

Fig.2E

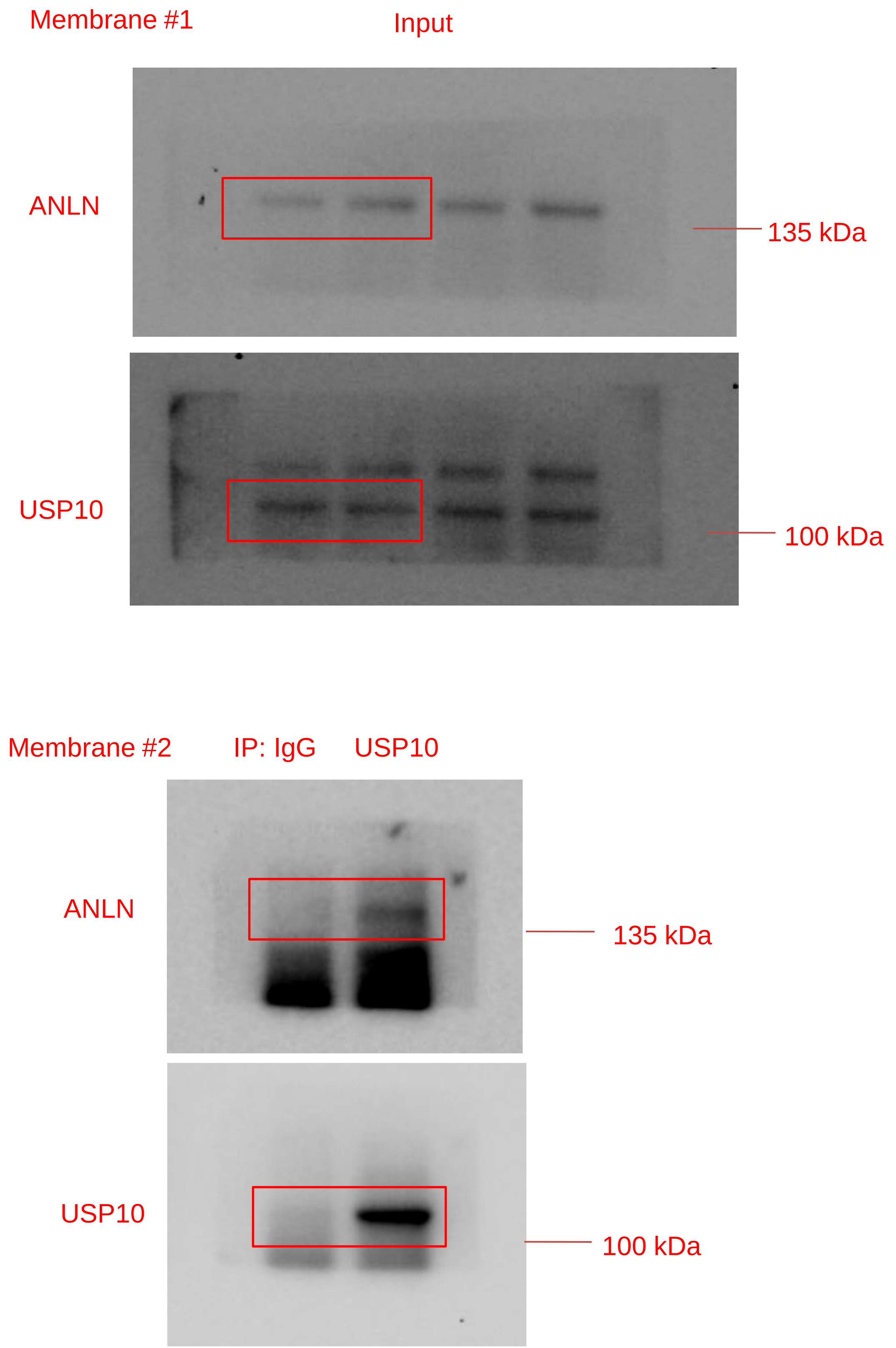
Membrane #1
Input
ANLN
135 kDa
USP10
100 kDa
Membrane #2
IP: IgG
USP10
ANLN
135 kDa
USP10
100 kDa

Fig.2E Membrane #3

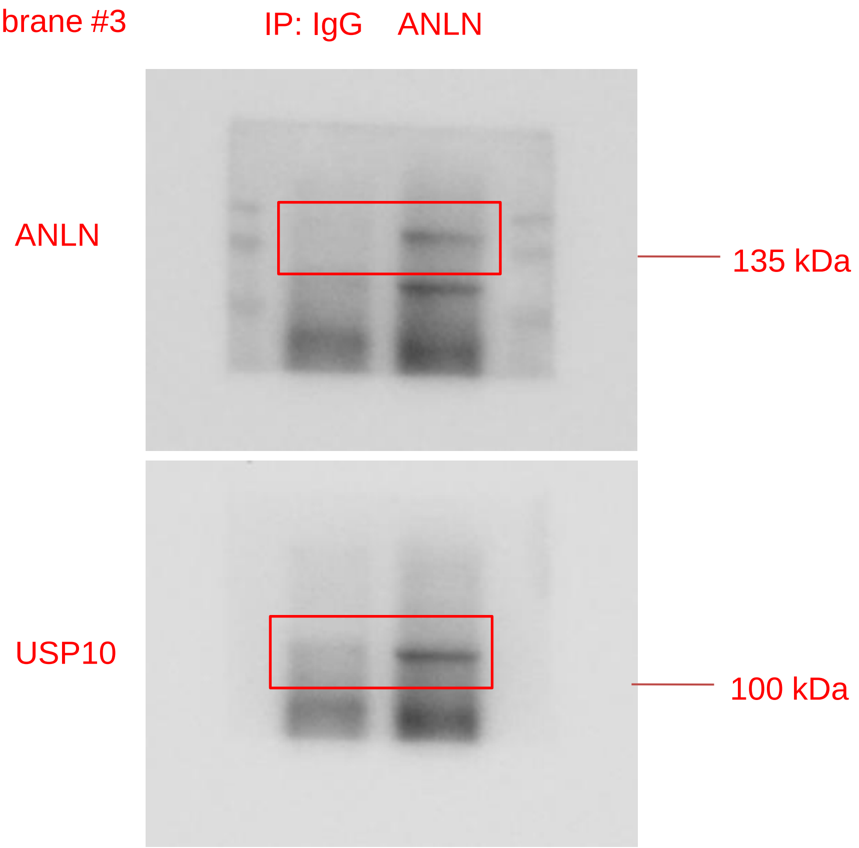


Fig.2G Membrane #1

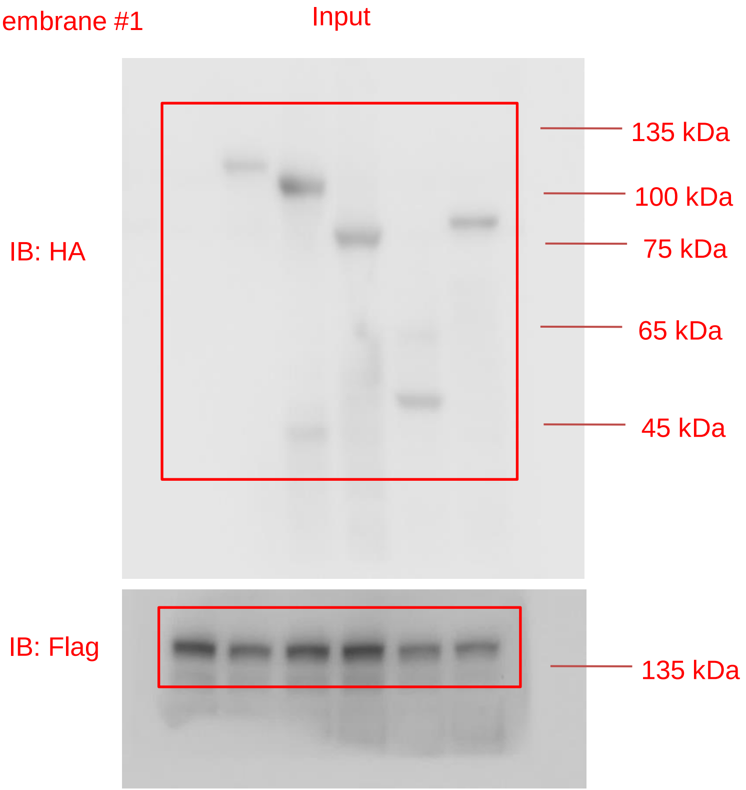

Fig.2G Membrane #2 IP: HA

IB: HA

IB: Flag

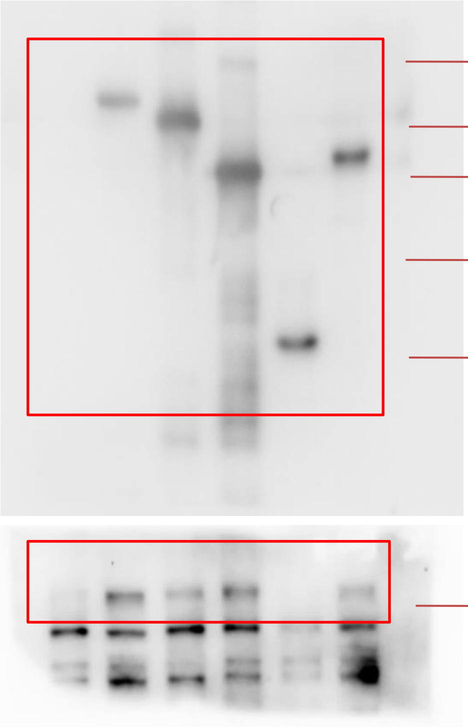

135 kDa
100 kDa
75 kDa
65 kDa
45 kDa
135 kDa

Fig.2I Membrane #1 Input

HA

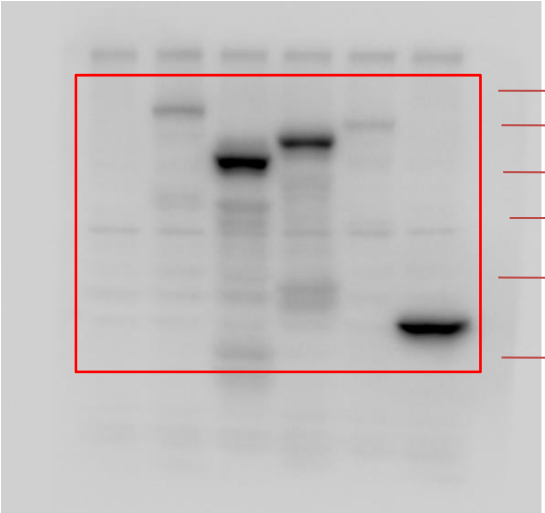

180 kDa
135 kDa
100 kDa
75 kDa
65 kDa
45 kDa

Membrane #2 Input

Flag

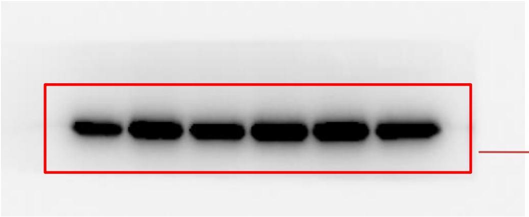

100 kDa

Fig.2I Membrane #3

IP: HA

IB: HA

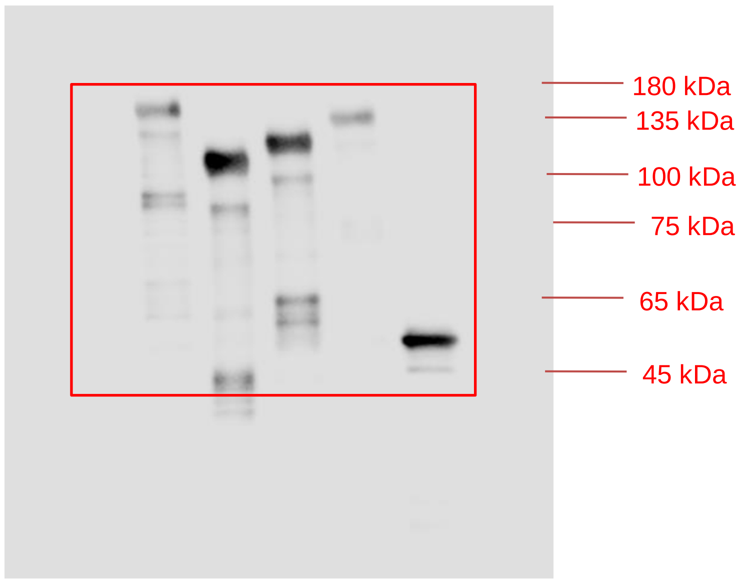


Membrane #4

IP: HA

IB: Flag

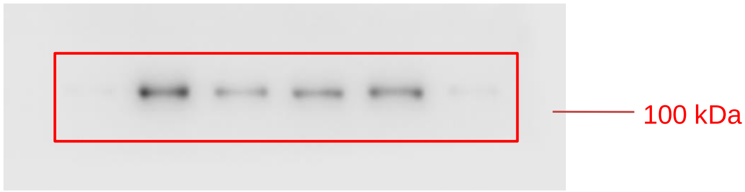


Fig.2J

Input

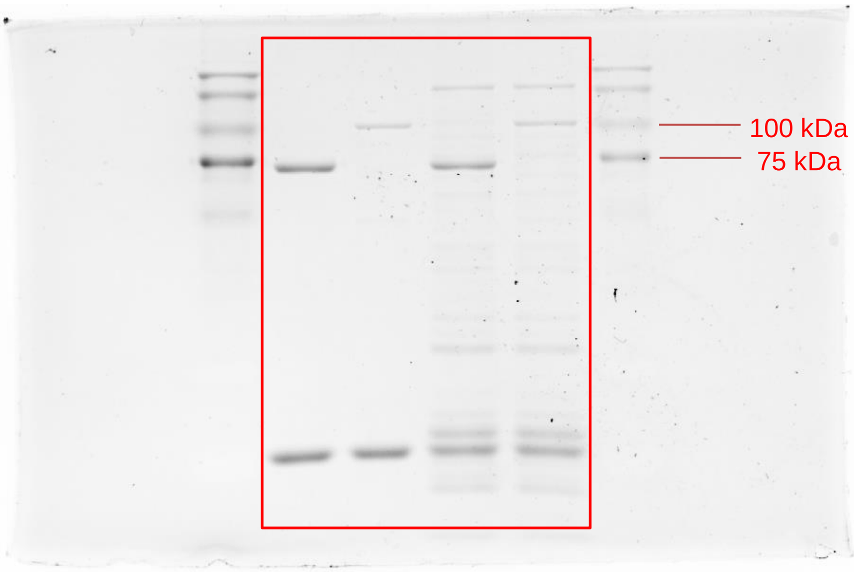


CBB

Fig.2J

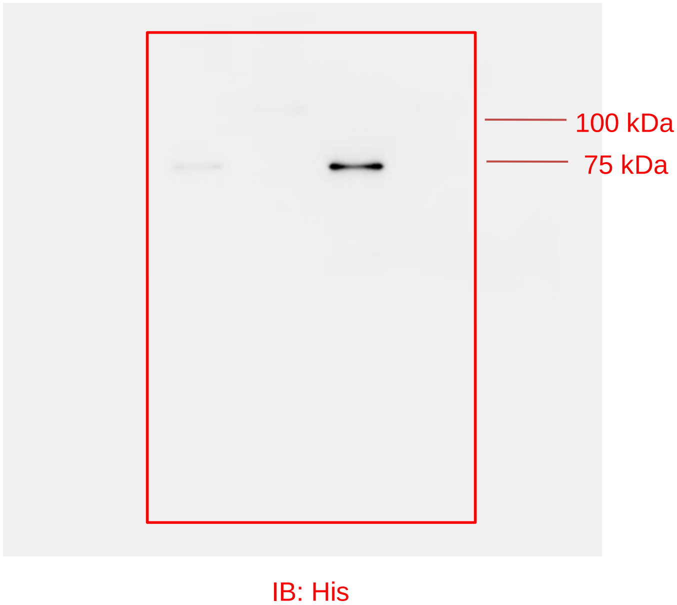

GST-pulldown
100 kDa
75 kDa
IB: His


Fig.2K

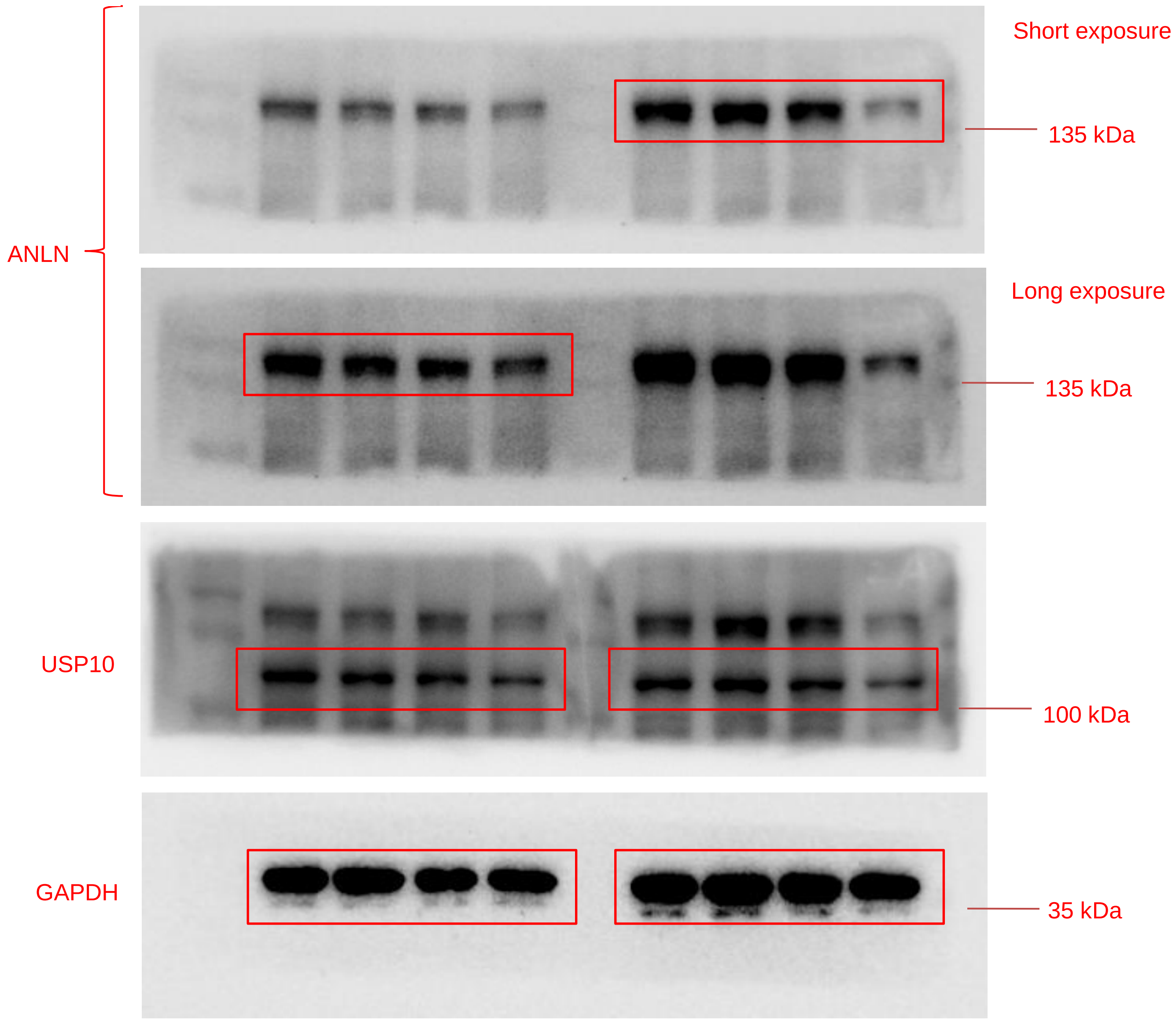

KYSE150
KYSE510
Short exposure
135 kDa
Long exposure
135 kDa
ANLN
USP10
100 kDa
GAPDH
35 kDa

Fig.2L

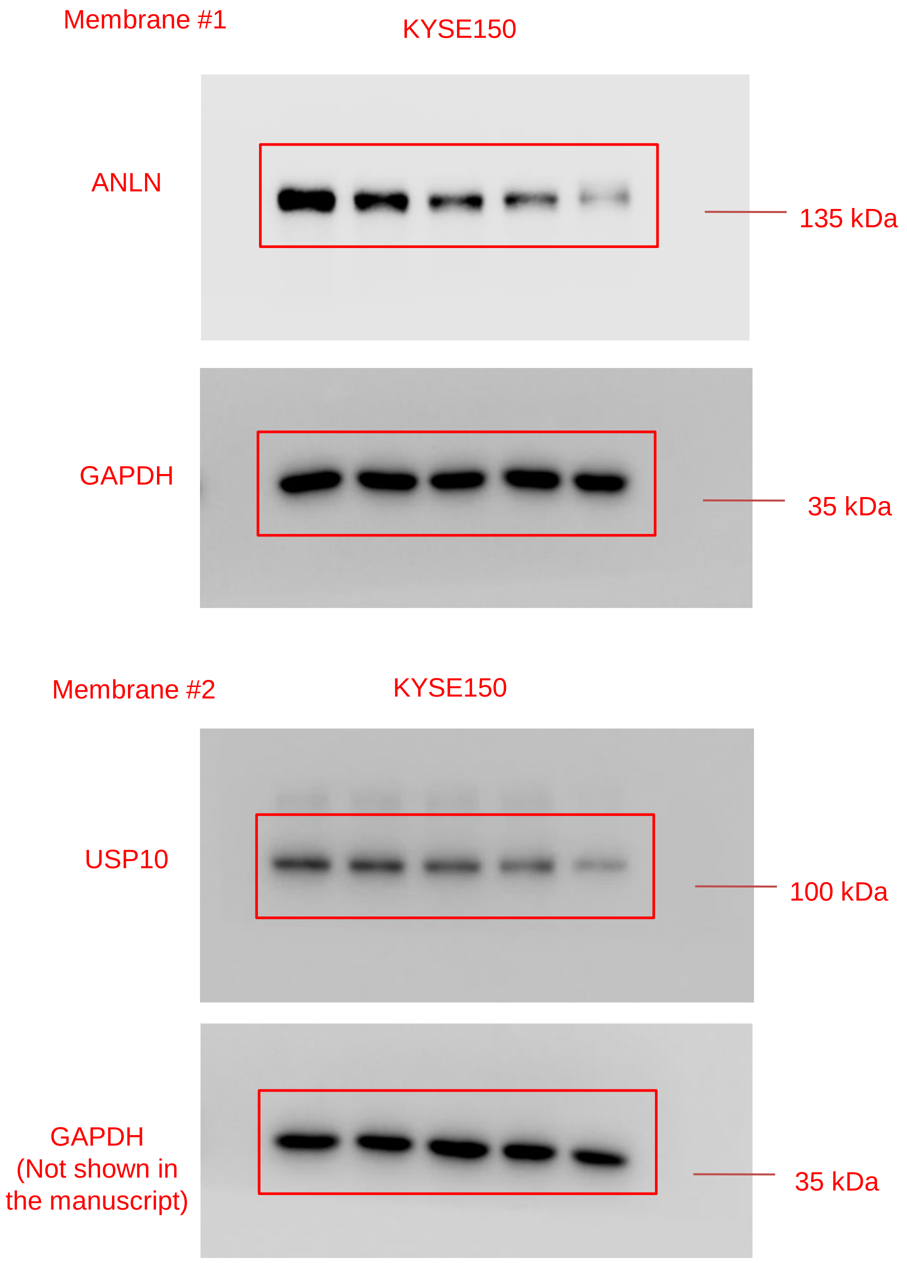

Membrane #1
KYSE150
ANLN
135 kDa
GAPDH
35 kDa
Membrane #2
KYSE150
USP10
100 kDa
GAPDH
(Not shown in
the manuscript)
35 kDa

Fig.2L Membrane #3 KYSE510

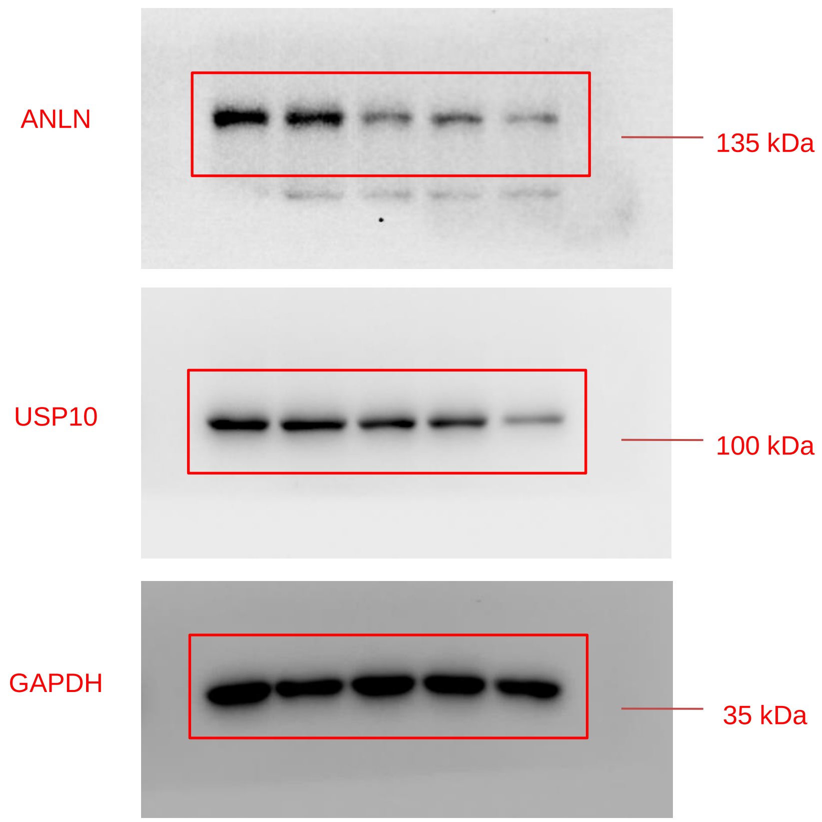


Fig.2M Membrane #1

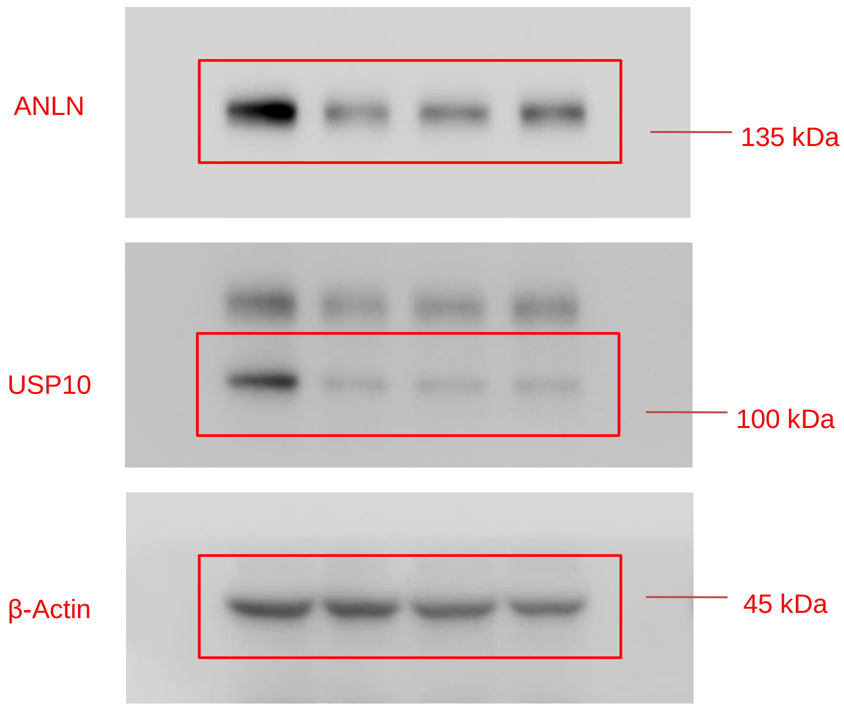


KYSE150

Fig.2M

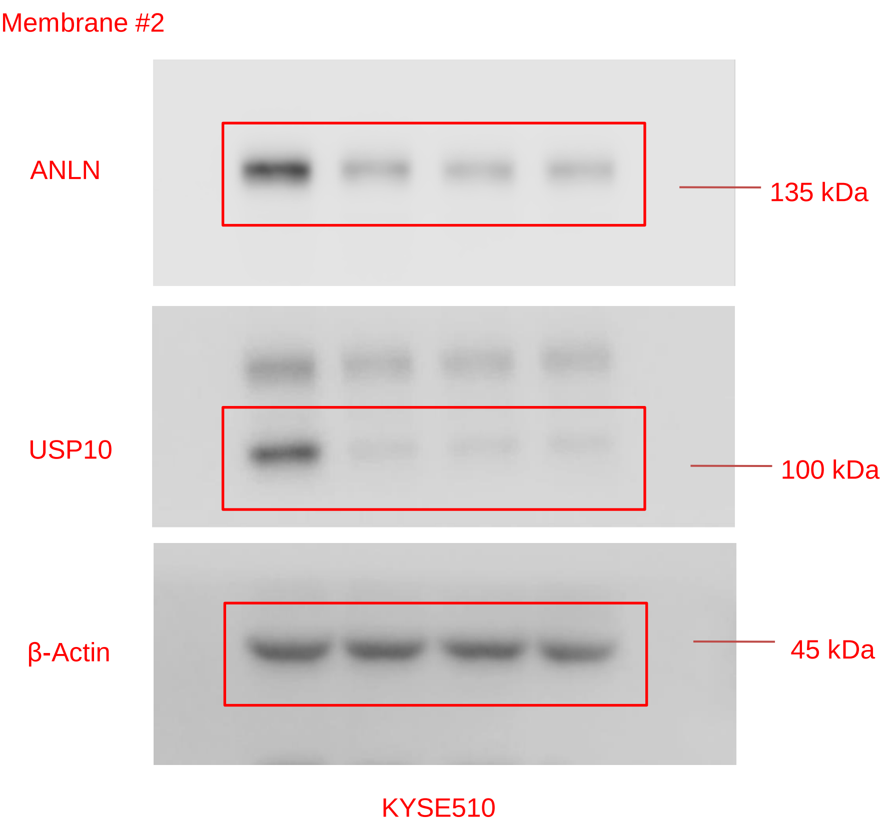
Membrane #2
ANLN
135 kDa
USP10
100 kDa
β-Actin
45 kDa
KYSE510

Fig.2N

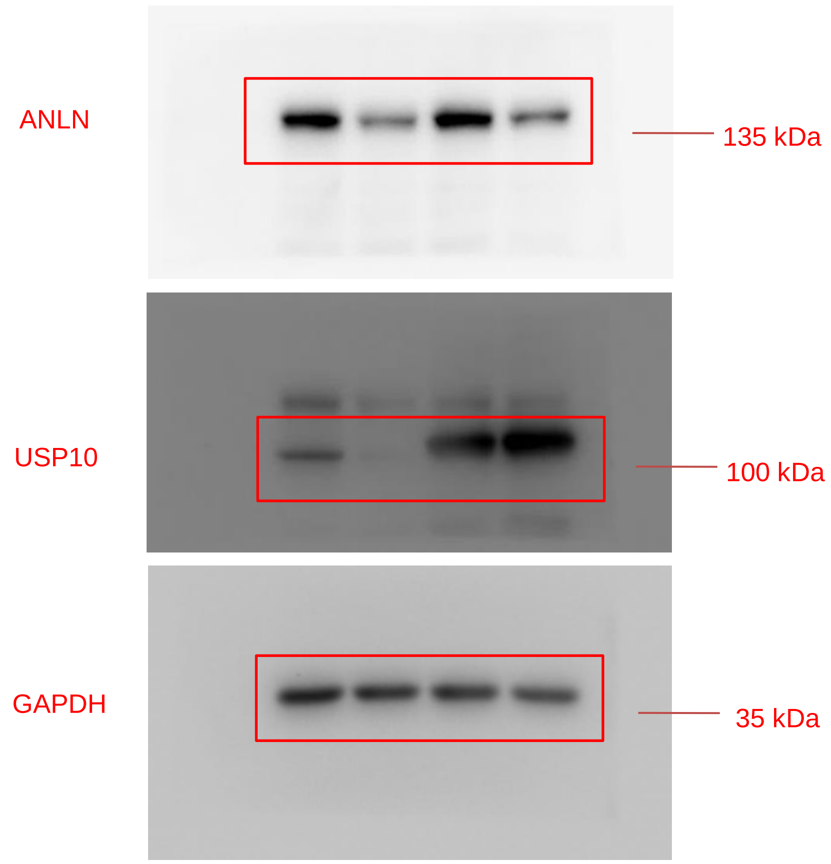
ANLN
135 kDa
USP10
100 kDa
GAPDH
35 kDa

Fig.3A

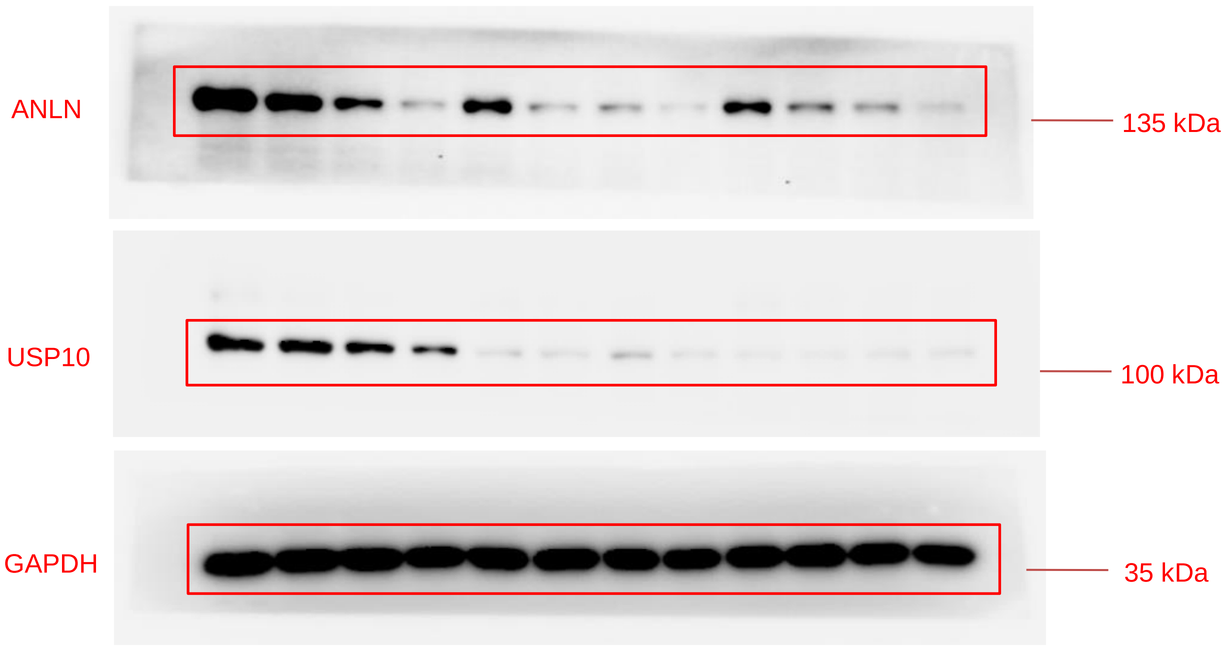
ANLN
135 kDa
USP10
100 kDa
GAPDH
35 kDa

Fig.3B

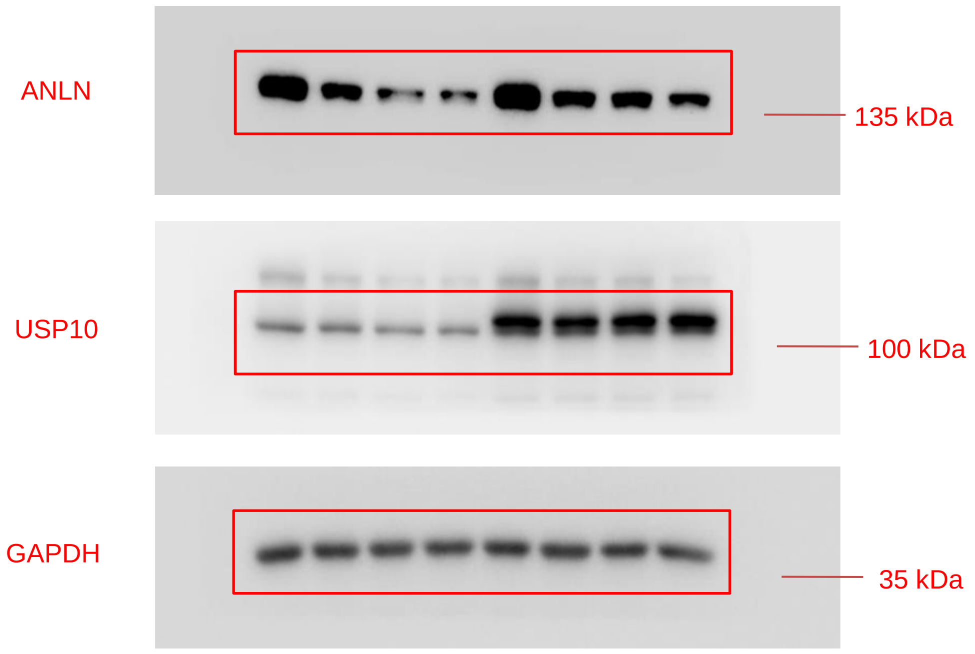
ANLN
135 kDa
USP10
100 kDa
GAPDH
35 kDa

Fig.3C

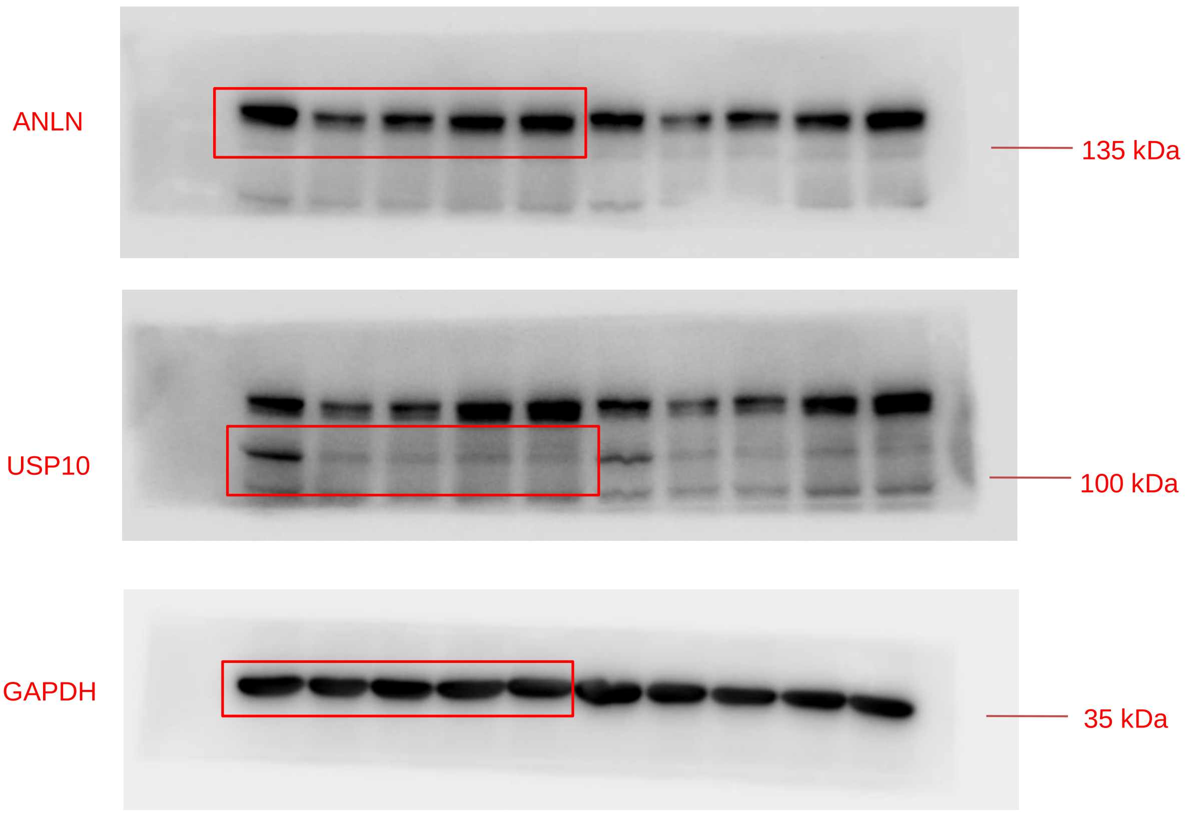
ANLN
135 kDa
USP10
100 kDa
GAPDH
35 kDa

Fig.3D

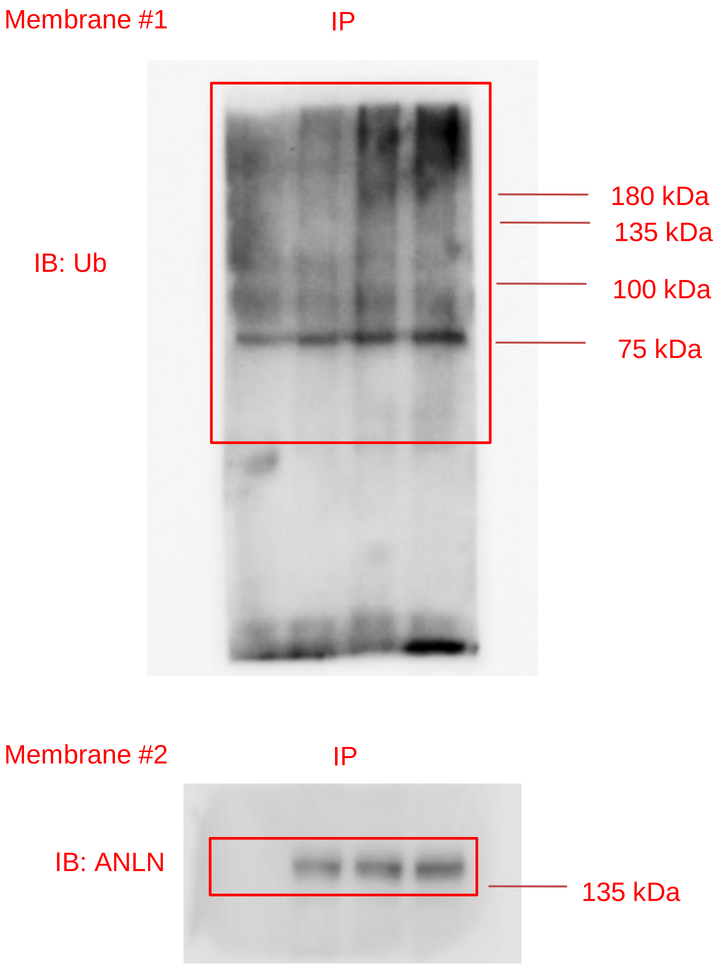
Membrane #1
IP
IB: Ub
180 kDa
135 kDa
100 kDa
75 kDa
Membrane #2
IP
IB: ANLN
135 kDa

Fig.3D

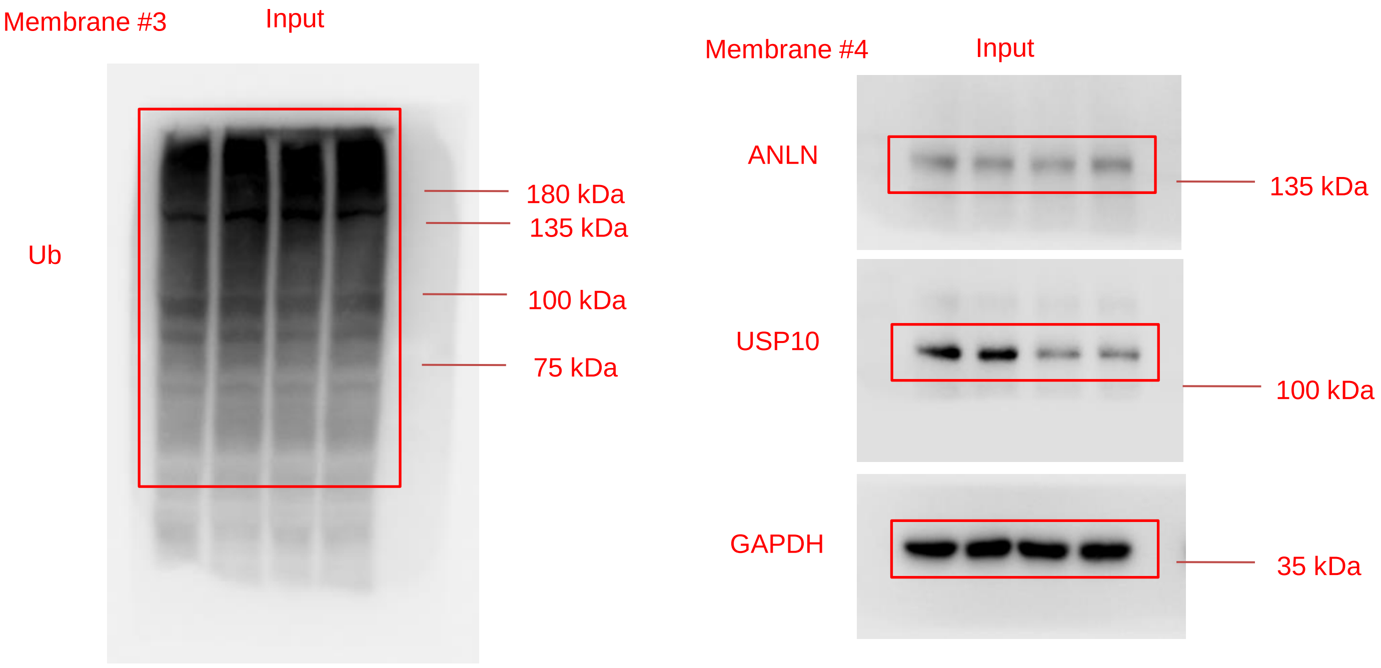
Membrane #3
Input
Ub
180 kDa
135 kDa
100 kDa
75 kDa
Membrane #4
Input
ANLN
135 kDa
USP10
100 kDa
GAPDH
35 kDa

Fig.3E

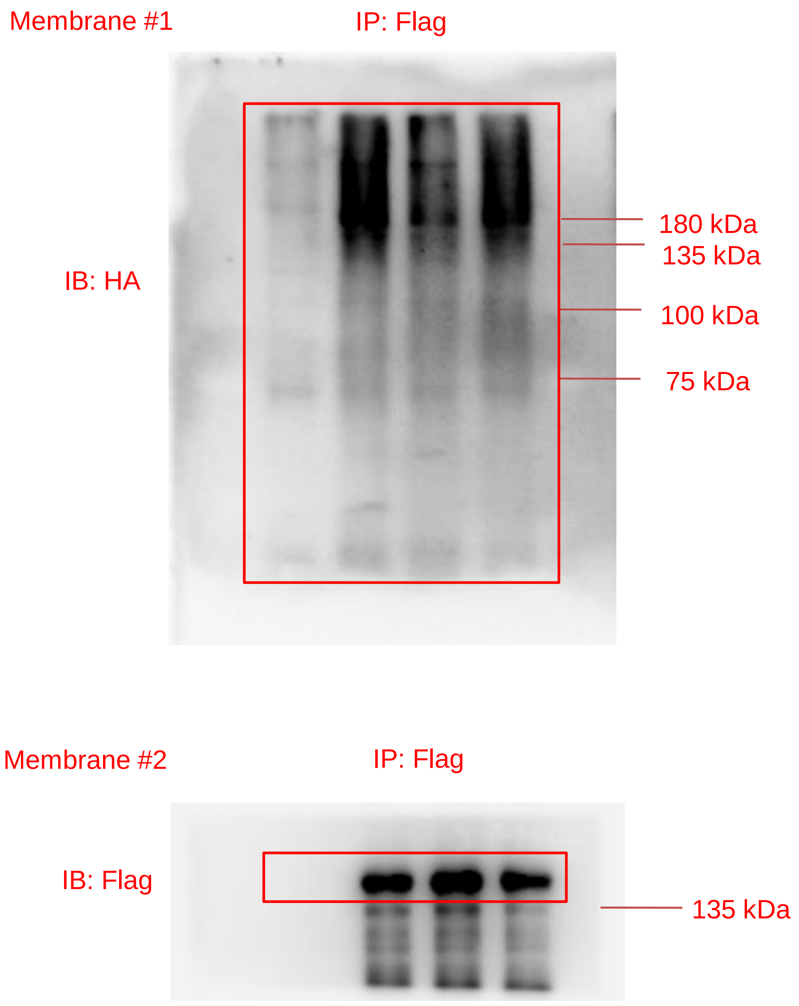
Membrane #1
IP: Flag
IB: HA
180 kDa
135 kDa
100 kDa
75 kDa
Membrane #2
IP: Flag
IB: Flag
135 kDa

Fig.3E

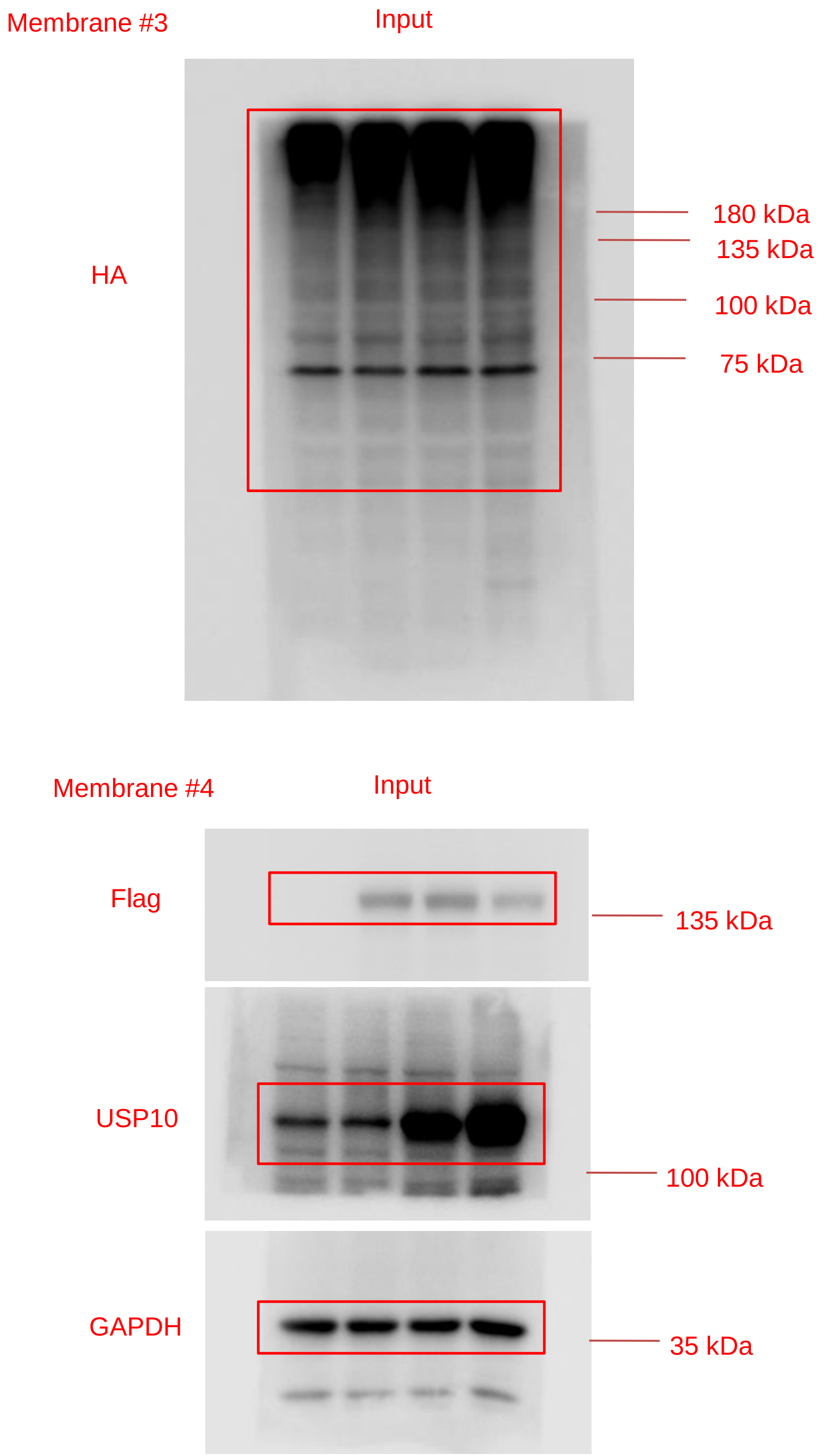
Membrane #3
Input
HA
180 kDa
135 kDa
100 kDa
75 kDa
Membrane #4
Input
Flag
135 kDa
USP10
100 kDa
GAPDH
35 kDa

Fig.3F

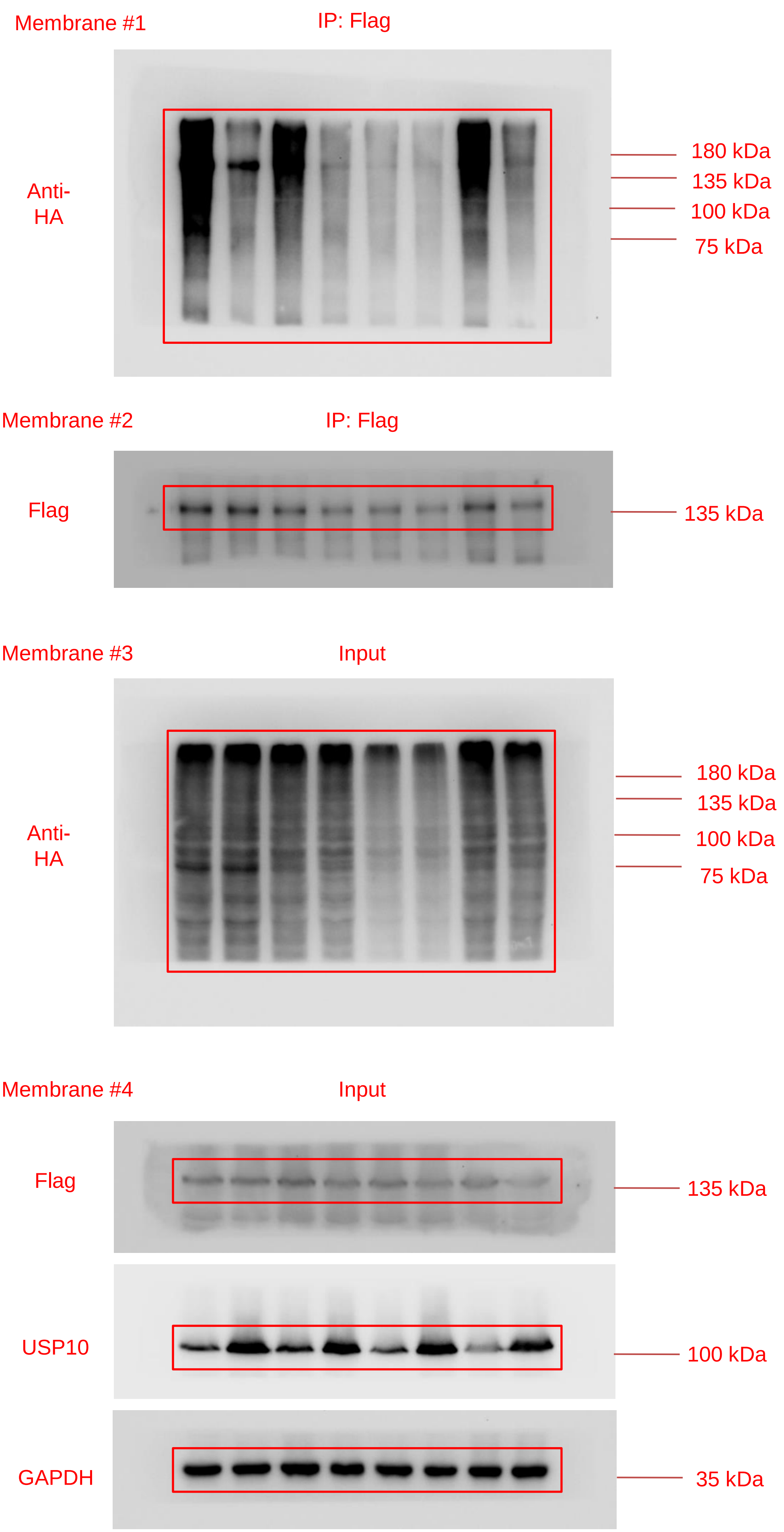

Membrane #1
IP: Flag
Anti-HA
180 kDa
135 kDa
100 kDa
75 kDa
Membrane #2
IP: Flag
Flag
135 kDa
Membrane #3
Input
Anti-HA
180 kDa
135 kDa
100 kDa
75 kDa
Membrane #4
Input
Flag
135 kDa
USP10
100 kDa
GAPDH
35 kDa

Fig.3F

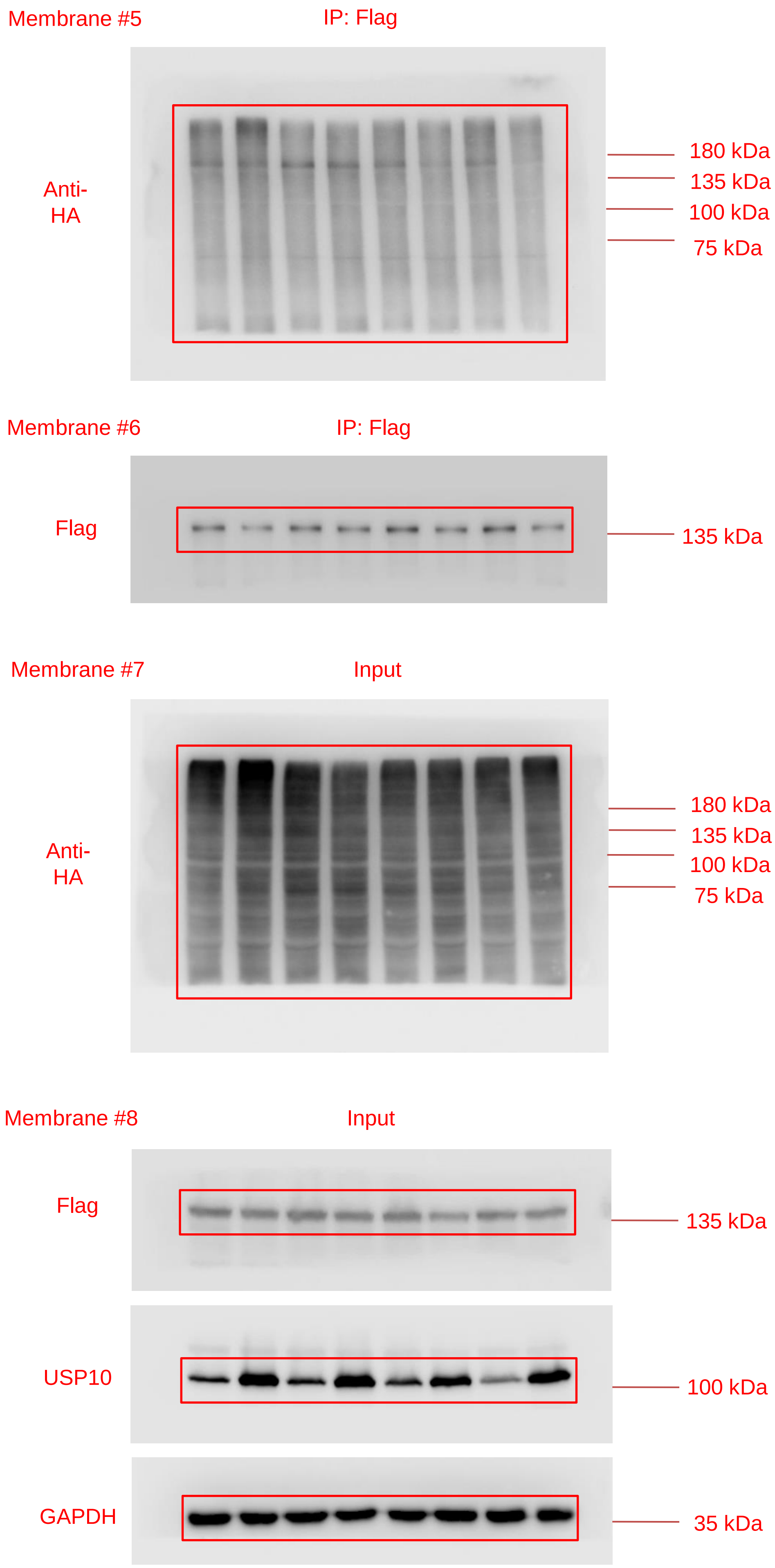
Membrane #5
IP: Flag
Anti-HA
180 kDa
135 kDa
100 kDa
75 kDa
Membrane #6
IP: Flag
Flag
135 kDa
Membrane #7
Input
Anti-HA
180 kDa
135 kDa
100 kDa
75 kDa
Membrane #8
Input
Flag
135 kDa
USP10
100 kDa
GAPDH
35 kDa

Fig.4A

Membrane #1

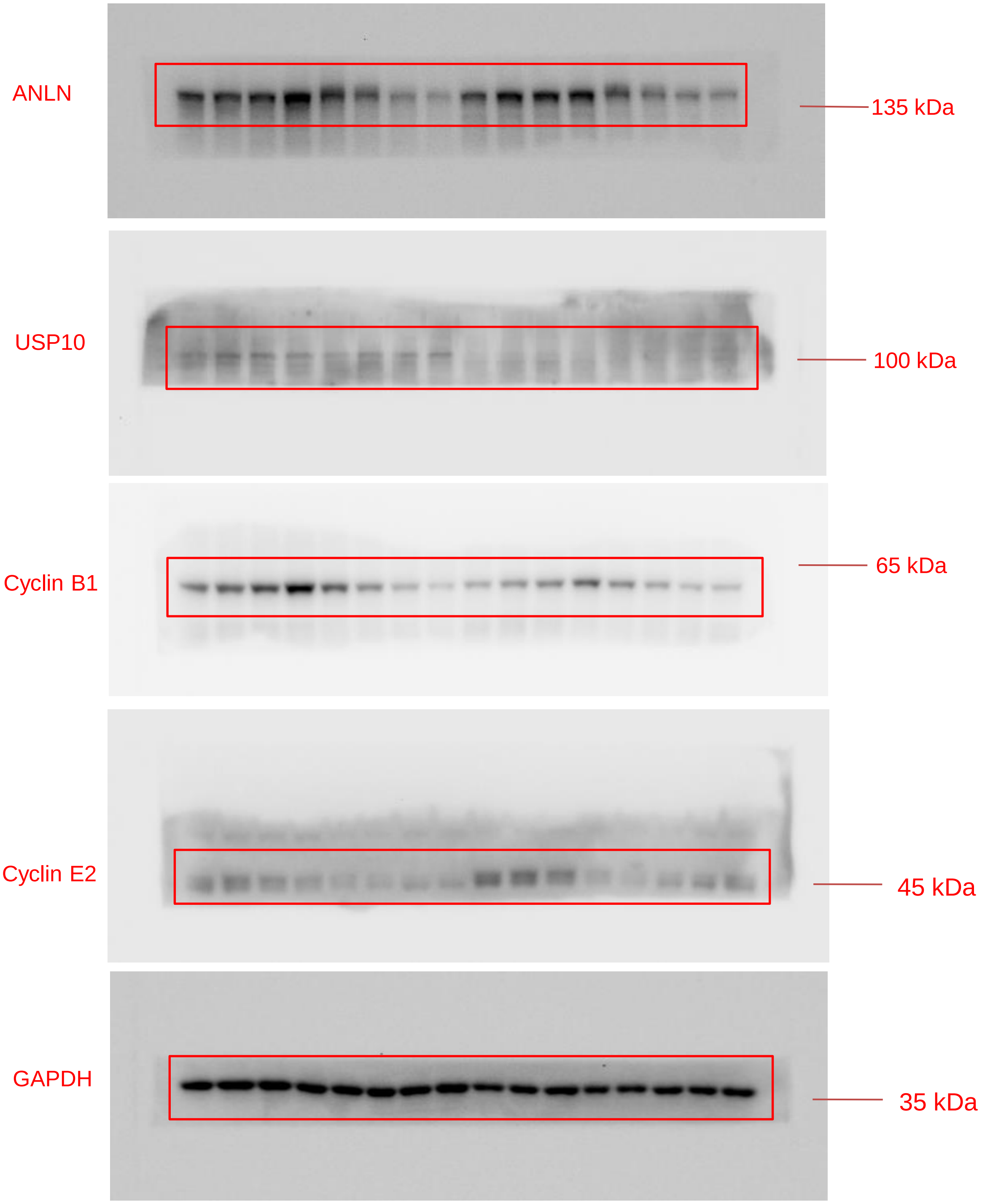

Fig.4A

Membrane #2

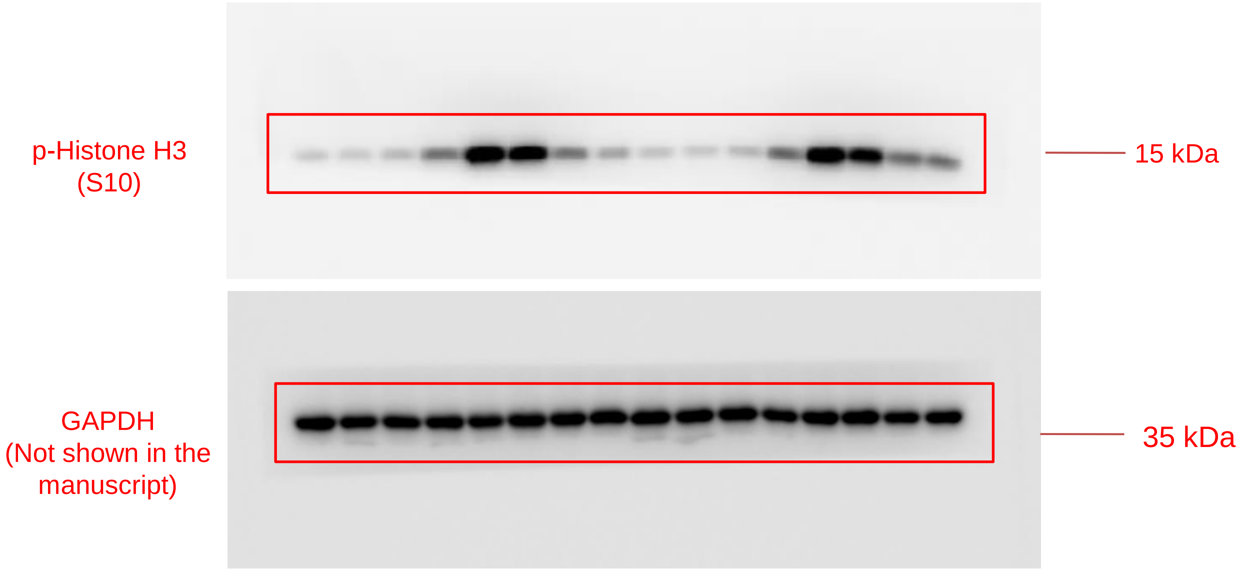


Fig.4B

Membrane #1

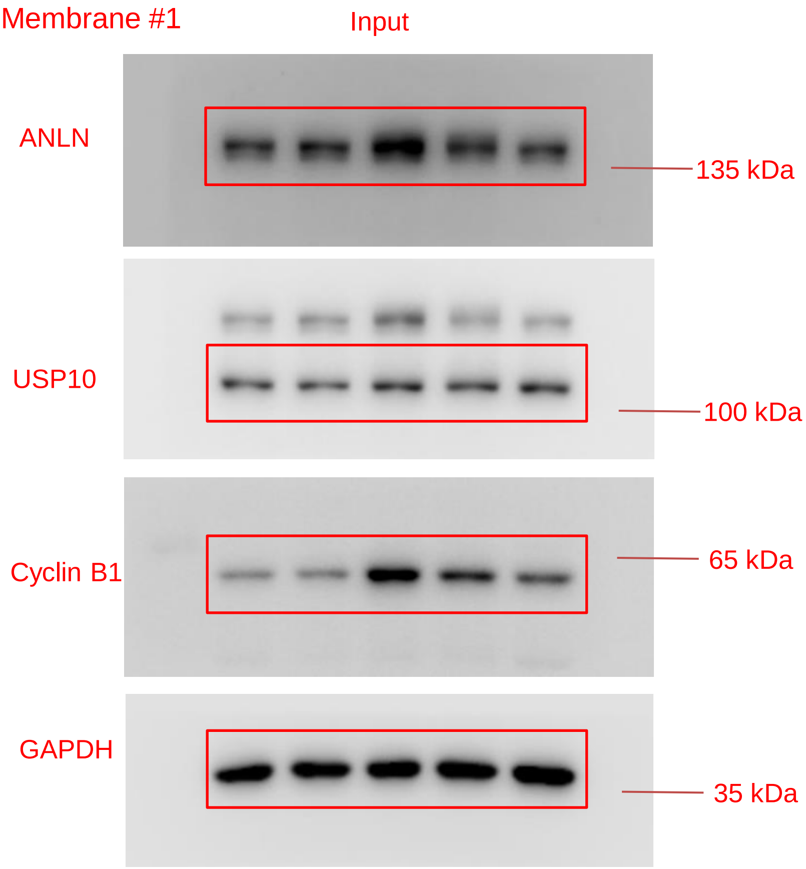

Fig.4B

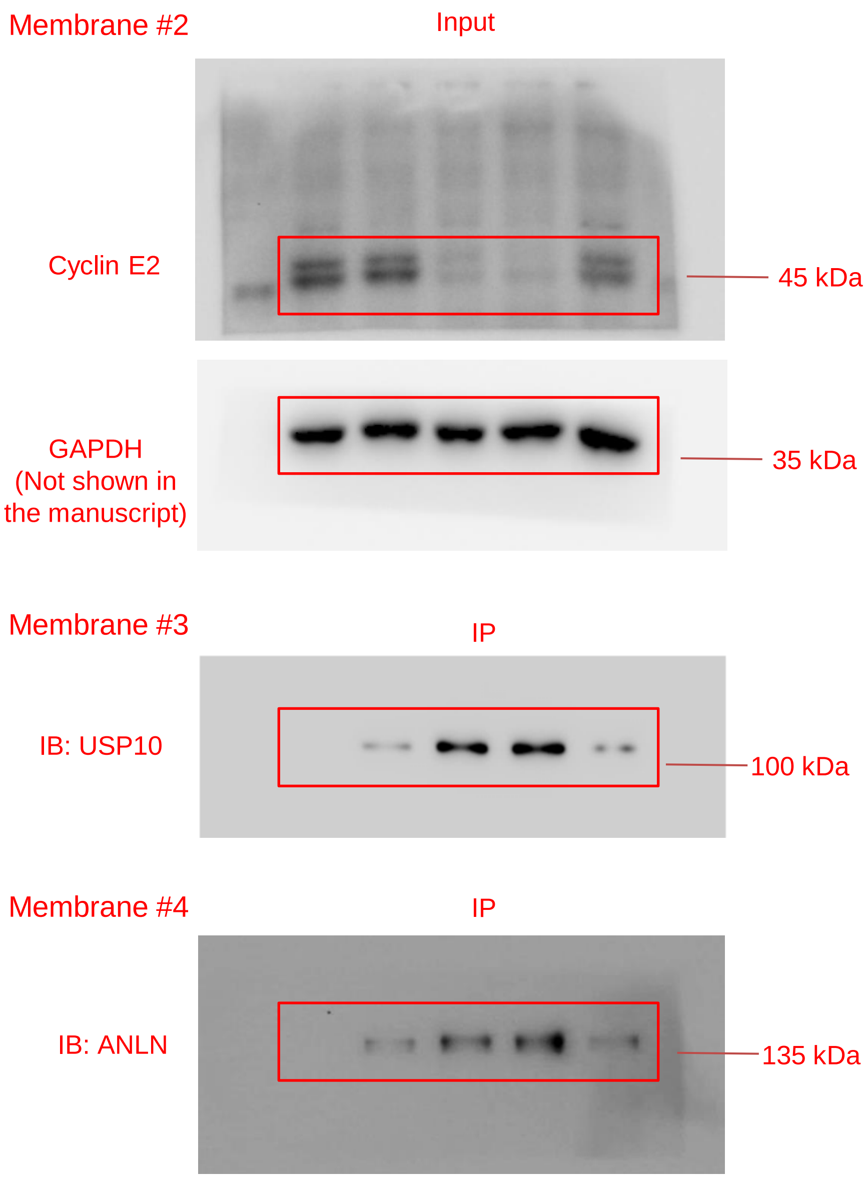
Membrane #2
Input
Cyclin E2
45 kDa
GAPDH
(Not shown in
the manuscript)
35 kDa
Membrane #3
IP
IB: USP10
100 kDa
Membrane #4
IP
IB: ANLN
135 kDa

Fig.4E

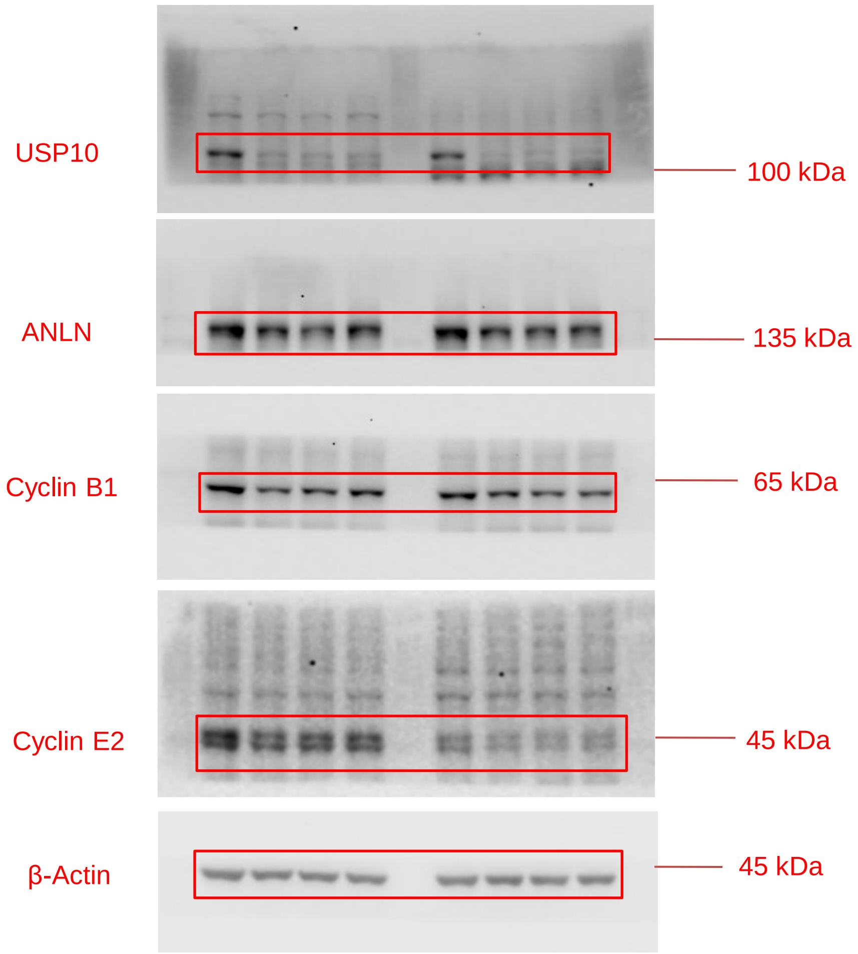


Fig.4H

Membrane #1

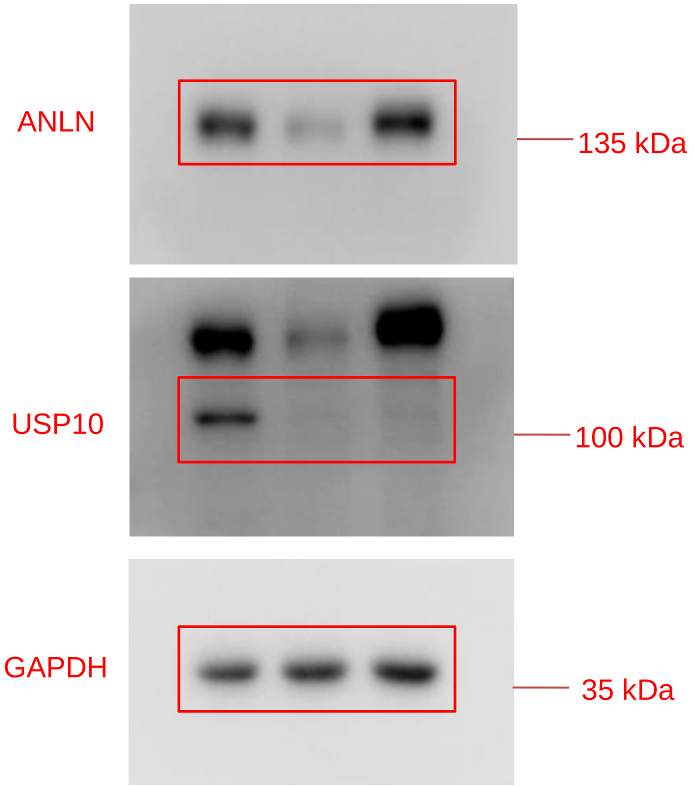

Fig.4H

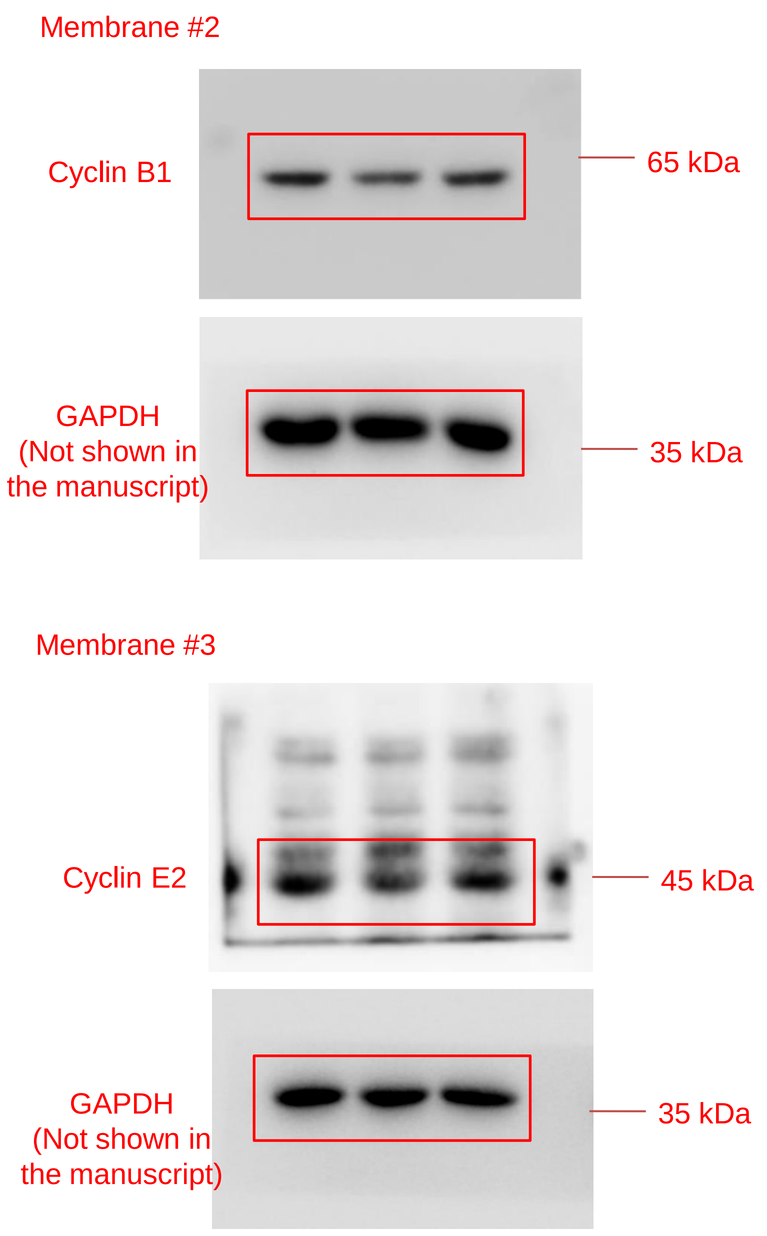
Membrane #2
Cyclin B1
65 kDa
GAPDH
(Not shown in
the manuscript)
35 kDa
Membrane #3
Cyclin E2
45 kDa
GAPDH
(Not shown in
the manuscript)
35 kDa

Fig.5A

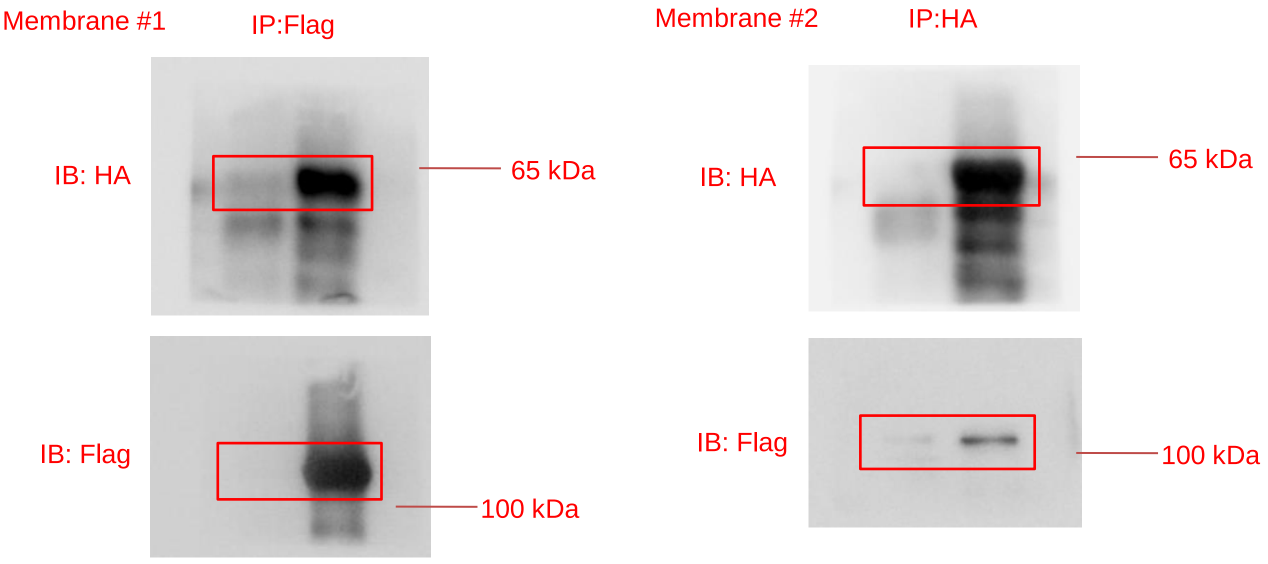
Membrane #1
IP:Flag
IB: HA
65 kDa
IB: Flag
100 kDa
Membrane #2
IP:HA
IB: HA
65 kDa
IB: Flag
100 kDa

Fig.5A

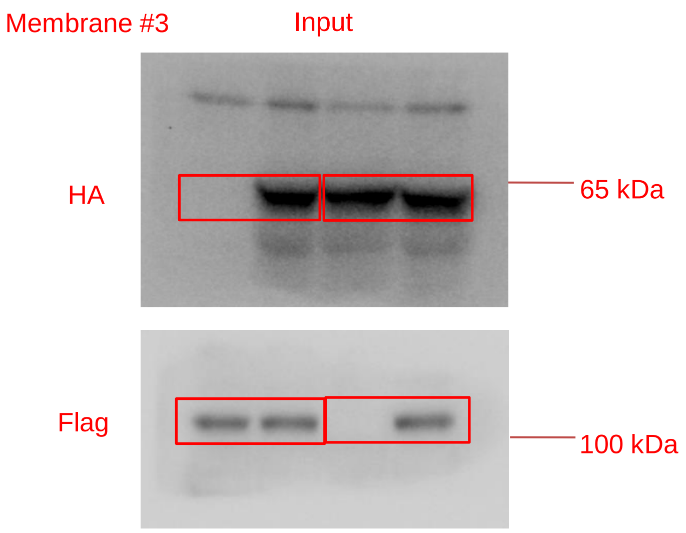
Membrane #3
Input
HA
65 kDa
Flag
100 kDa

Fig.5B

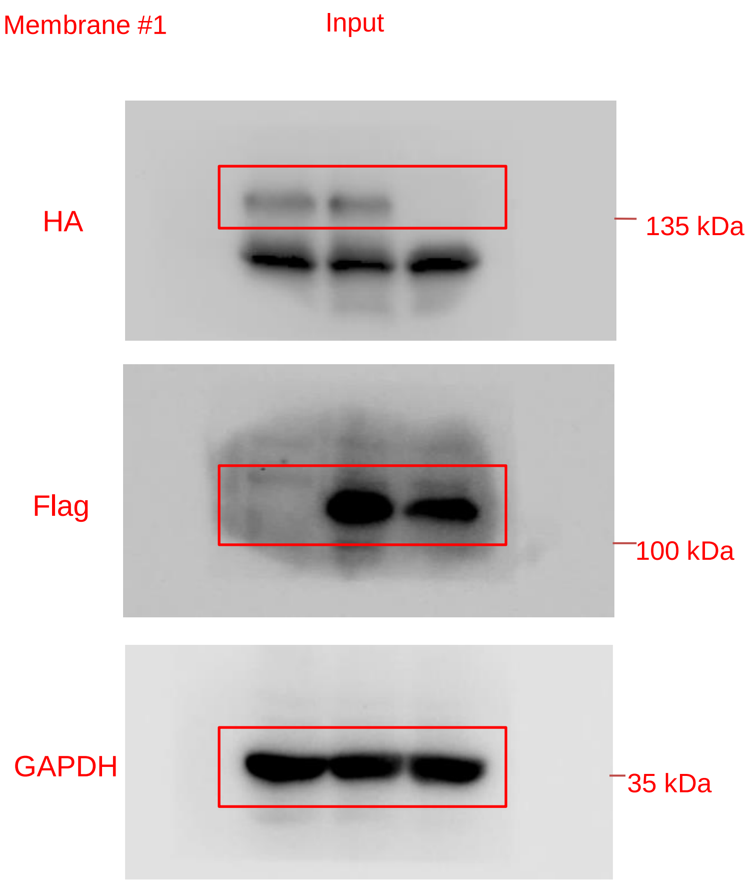
Membrane #1
Input
HA
135 kDa
Flag
100 kDa
GAPDH
35 kDa

Fig.5B

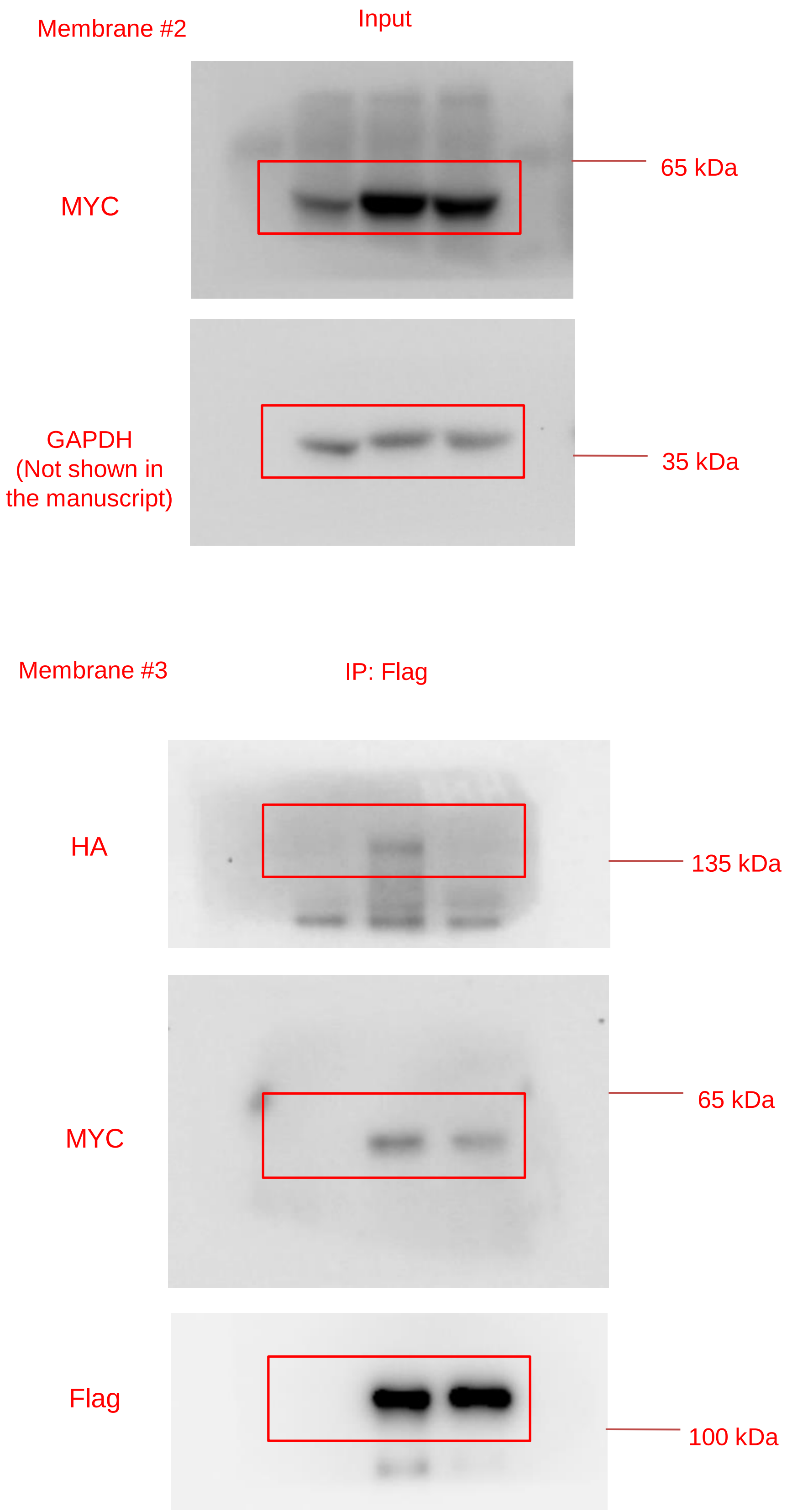
Membrane #2
Input
MYC
65 kDa
GAPDH
(Not shown in
the manuscript)
35 kDa
Membrane #3
IP: Flag
HA
135 kDa
MYC
65 kDa
Flag
100 kDa

Fig.5C

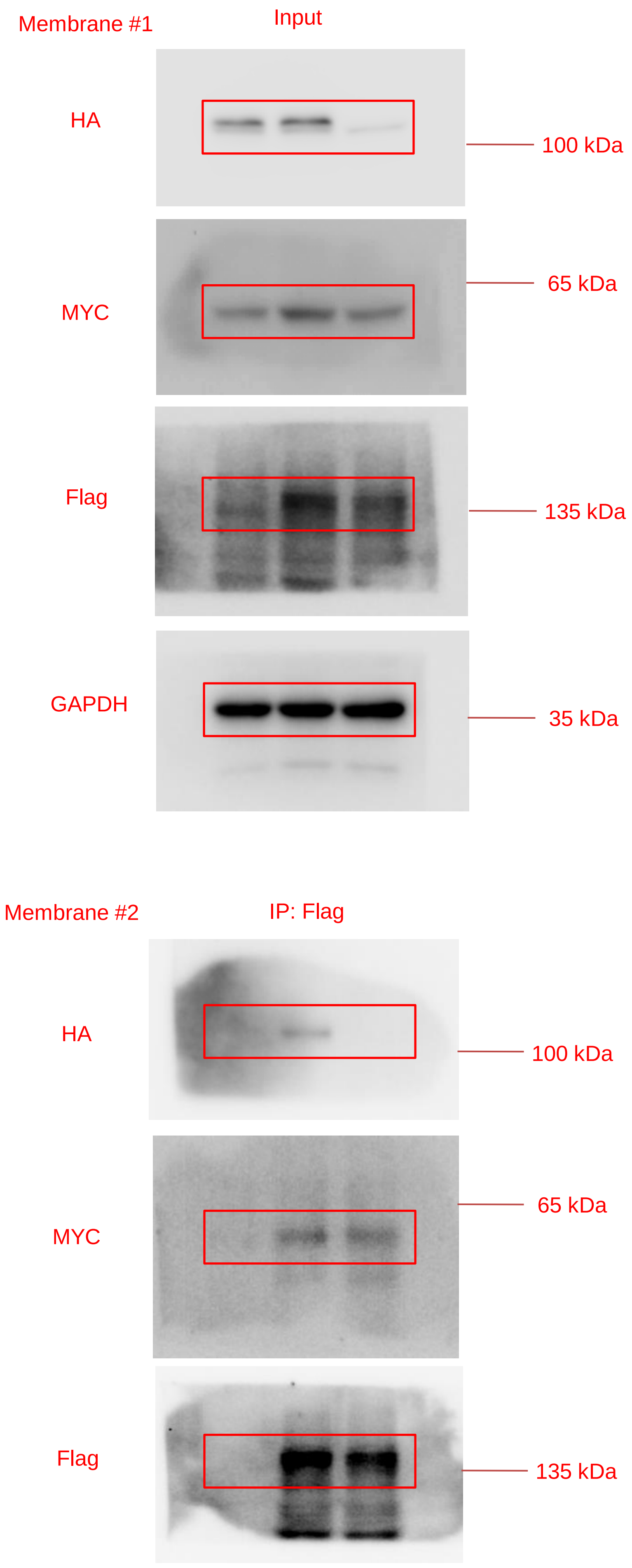

Membrane #1
Input
HA
100 kDa
MYC
65 kDa
Flag
135 kDa
GAPDH
35 kDa
Membrane #2
IP: Flag
HA
100 kDa
MYC
65 kDa
Flag
135 kDa

Fig.5D

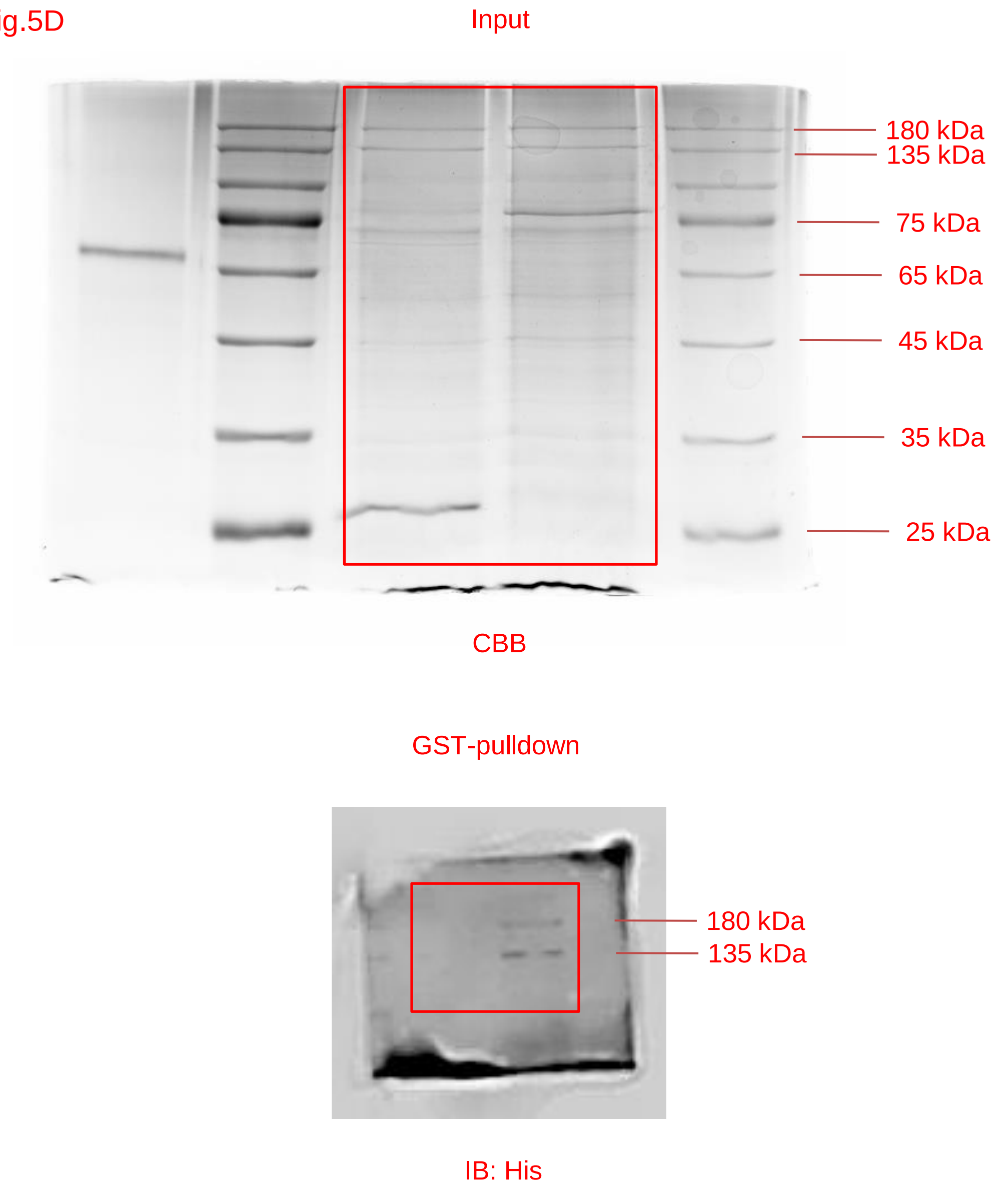
Input
180 kDa
135 kDa
75 kDa
65 kDa
45 kDa
35 kDa
25 kDa
CBB
GST-pulldown
180 kDa
135 kDa
IB: His

Fig.5E

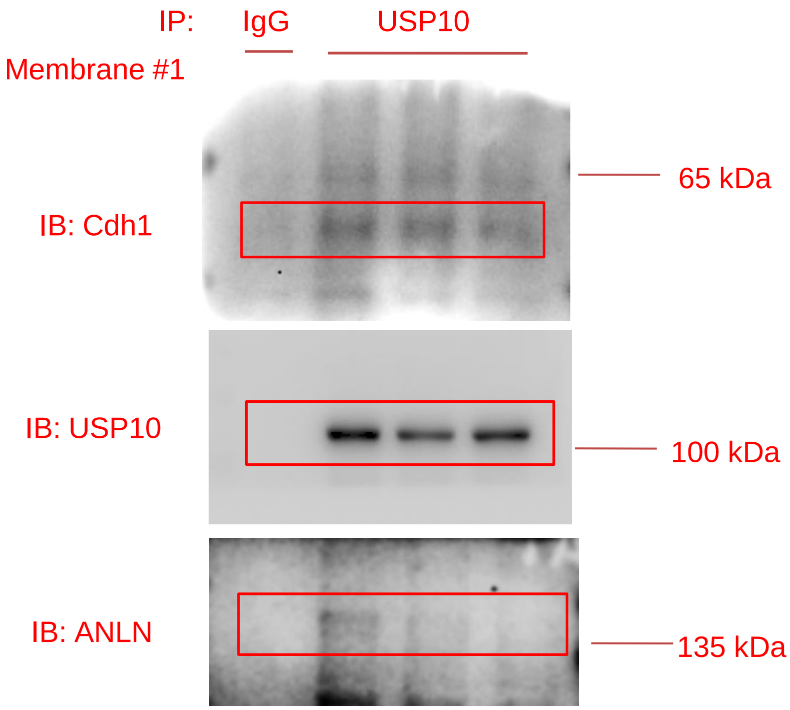
IP:
IgG
USP10
Membrane #1
IB: Cdh1
65 kDa
IB: USP10
100 kDa
IB: ANLN
135 kDa

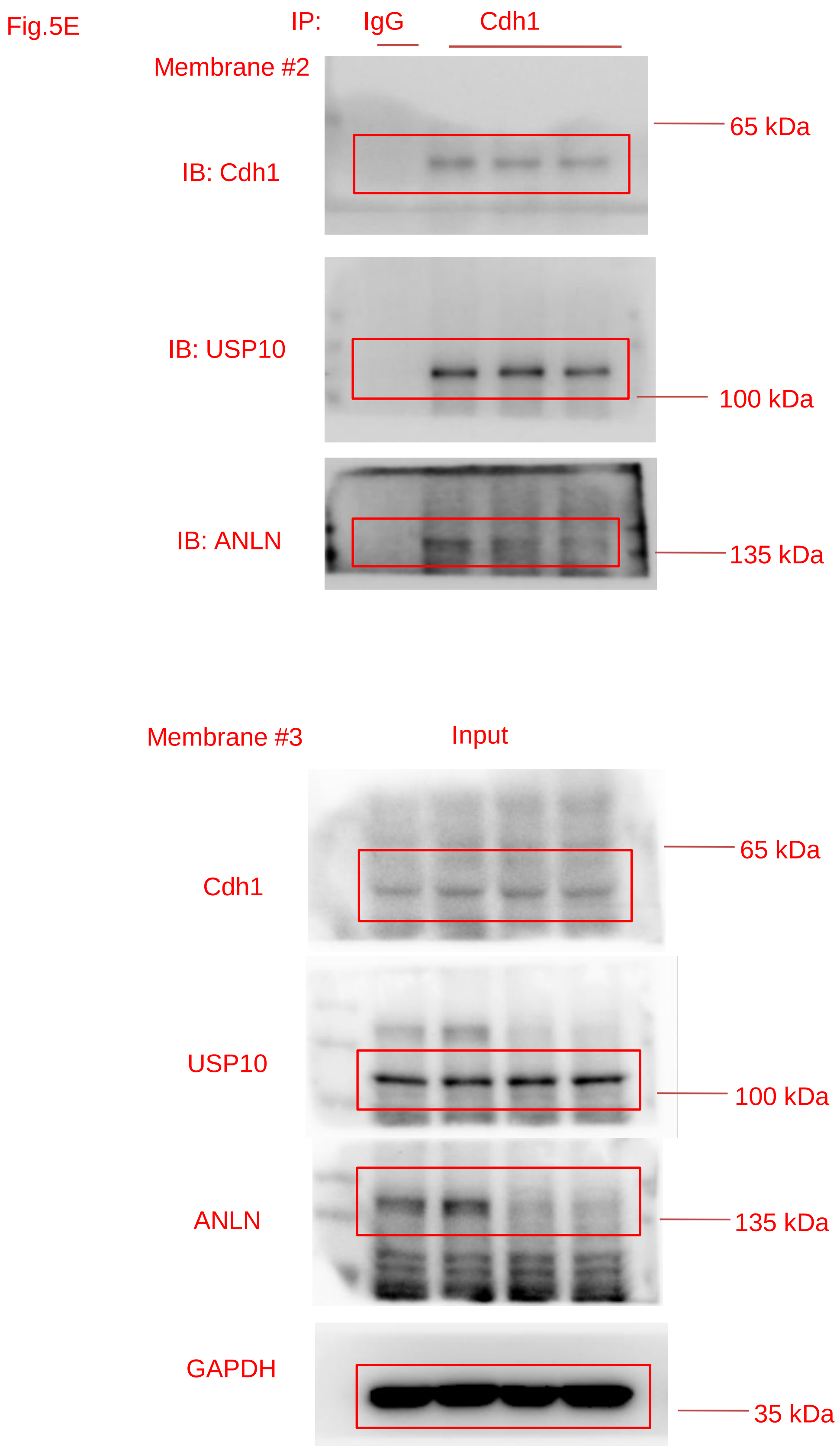

Fig.5E
IP:
IgG
Cdh1
Membrane #2
IB: Cdh1
65 kDa
IB: USP10
100 kDa
IB: ANLN
135 kDa
Membrane #3
Input
Cdh1
65 kDa
USP10
100 kDa
ANLN
135 kDa
GAPDH
35 kDa

Fig.5F

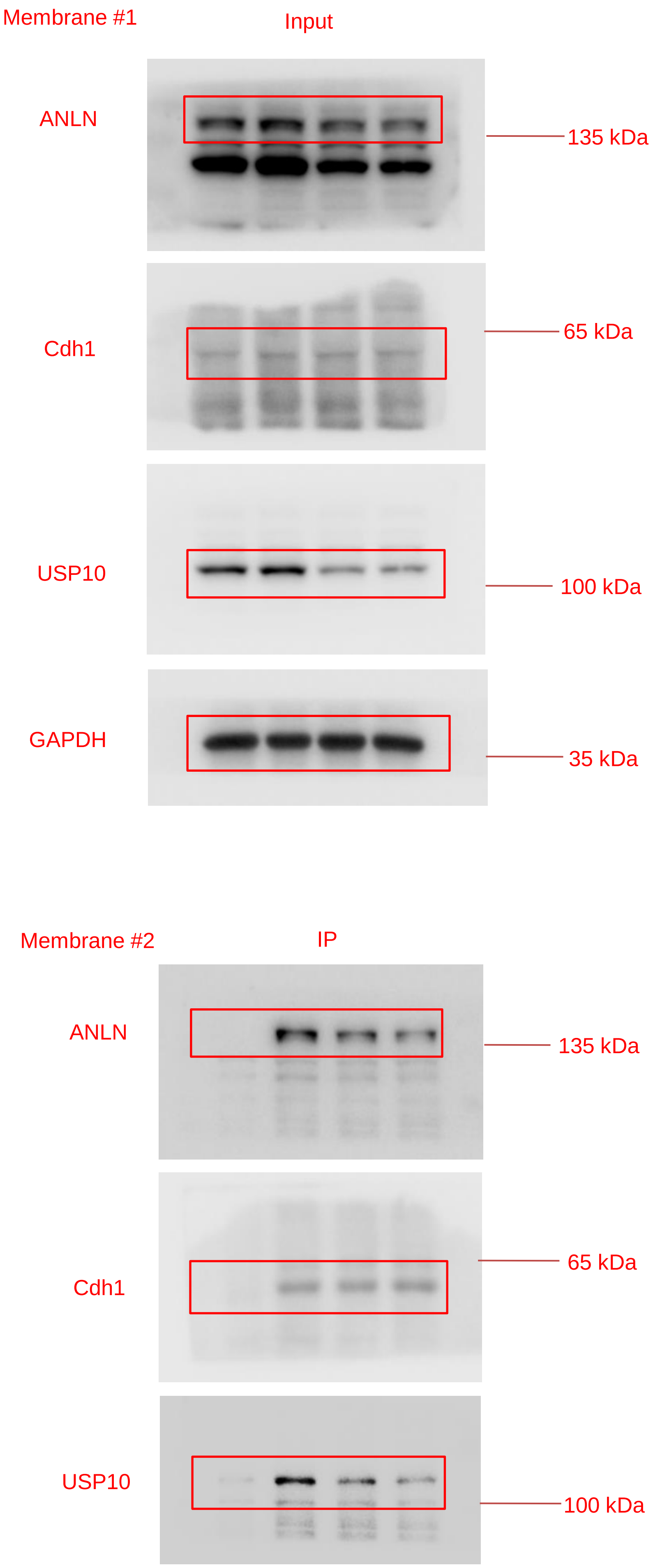

Membrane #1
Input
ANLN
135 kDa
Cdh1
65 kDa
USP10
100 kDa
GAPDH
35 kDa
Membrane #2
IP
ANLN
135 kDa
Cdh1
65 kDa
USP10
100 kDa

Fig.5G

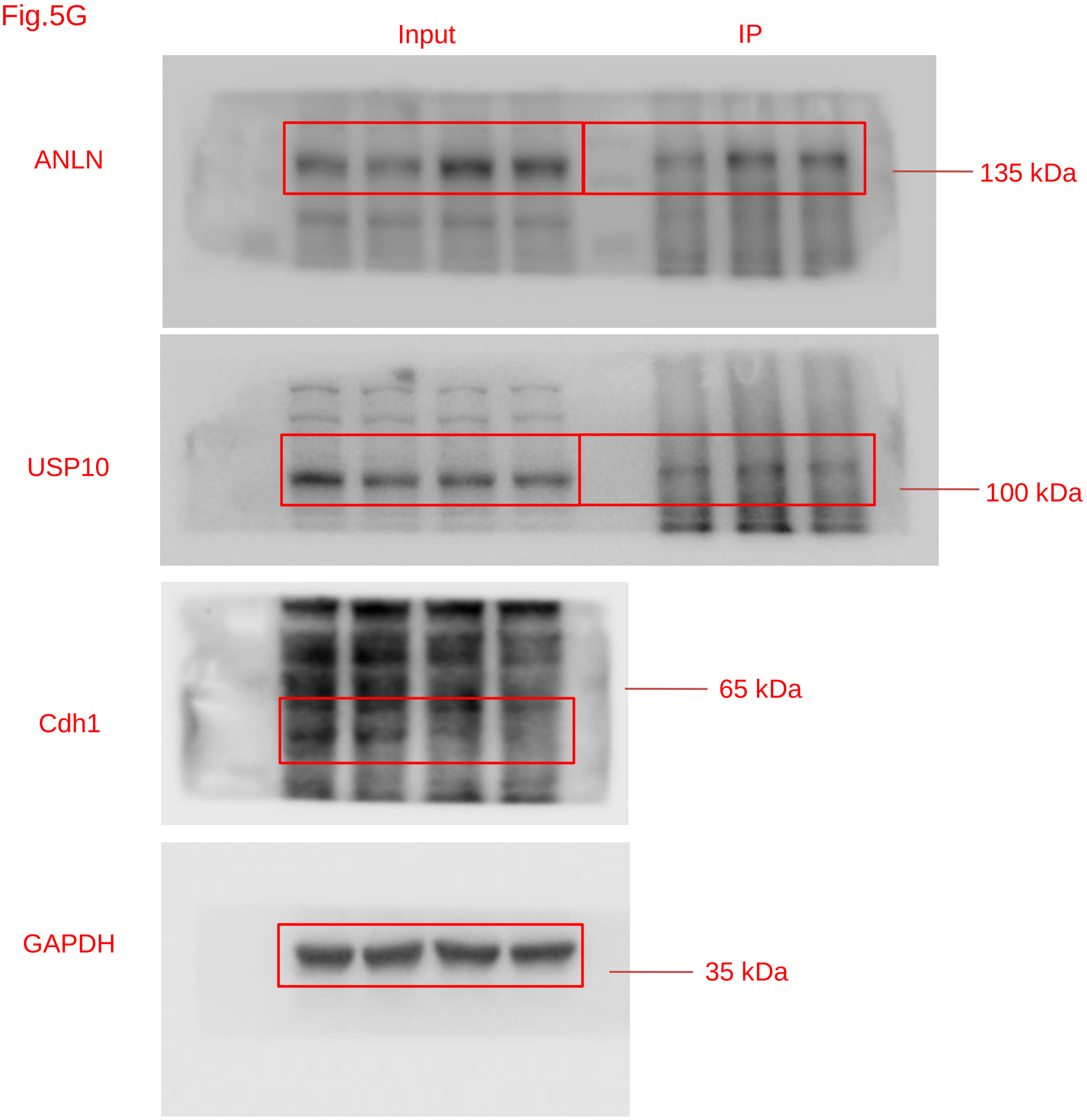
Input
IP
ANLN
135 kDa
USP10
100 kDa
Cdh1
65 kDa
GAPDH
35 kDa

Fig.5H

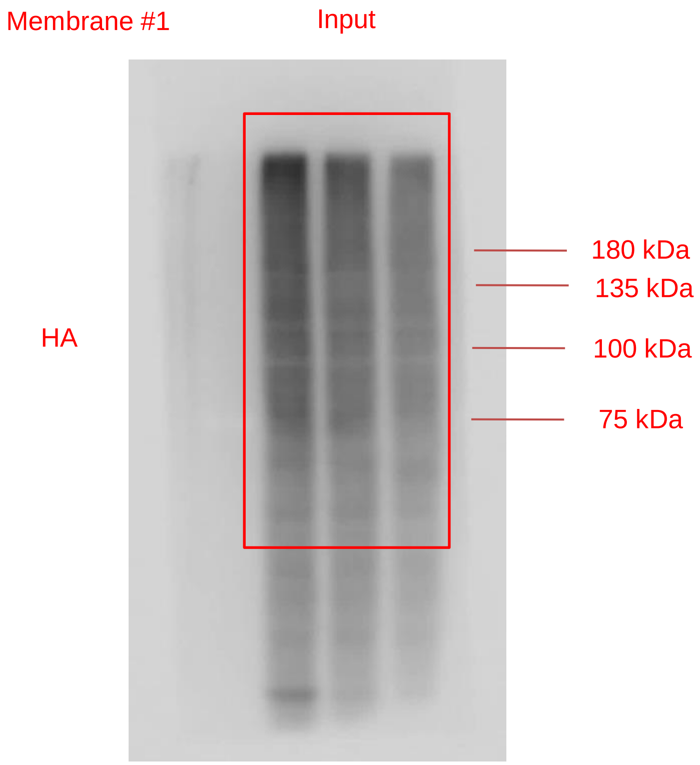
Membrane #1
Input
HA
180 kDa
135 kDa
100 kDa
75 kDa

Fig.5H

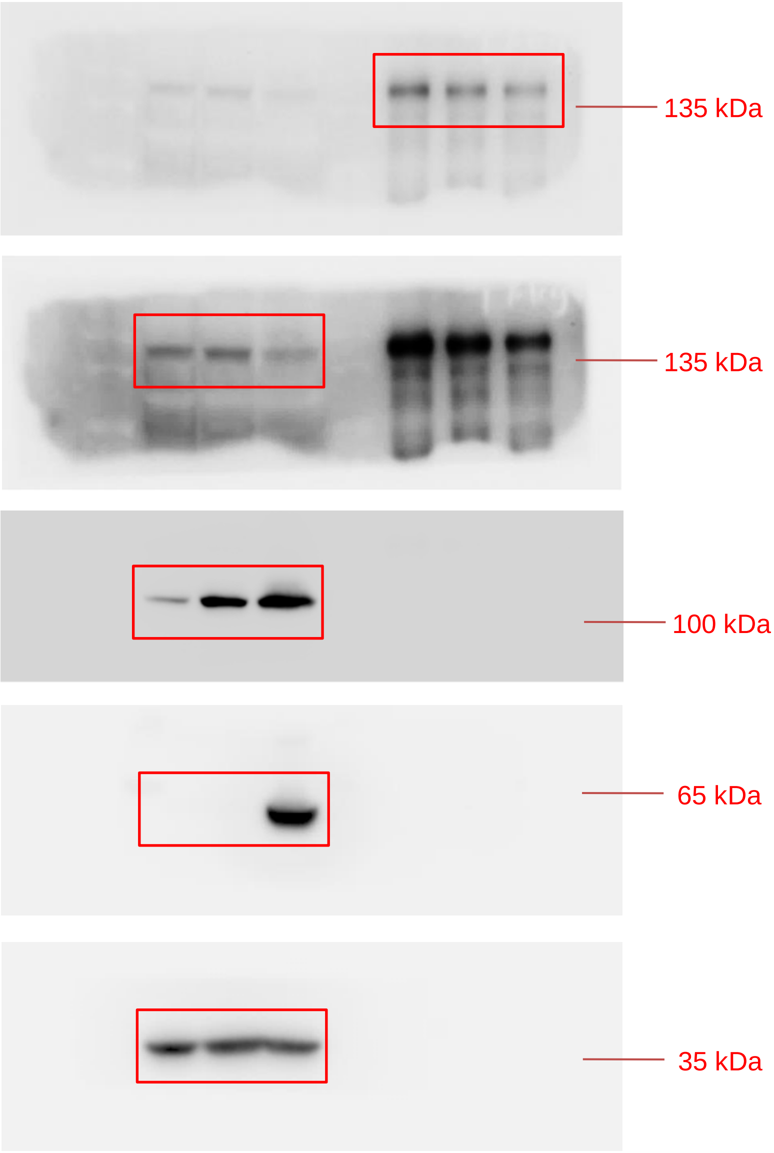
Membrane #2
Input
IP:Flag
Flag
Short exposure
135 kDa
Flag
Long exposure
135 kDa
USP10
100 kDa
MYC
65 kDa
GAPDH
35 kDa

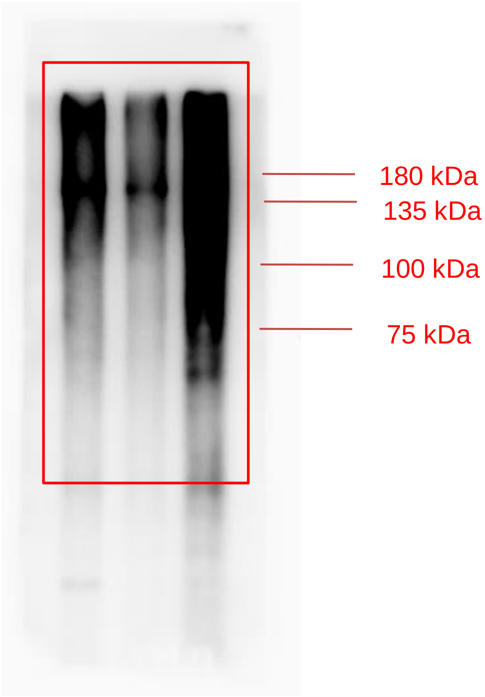
Membrane #3
IP:Flag
IB:HA
180 kDa
135 kDa
100 kDa
75 kDa

Fig.5I

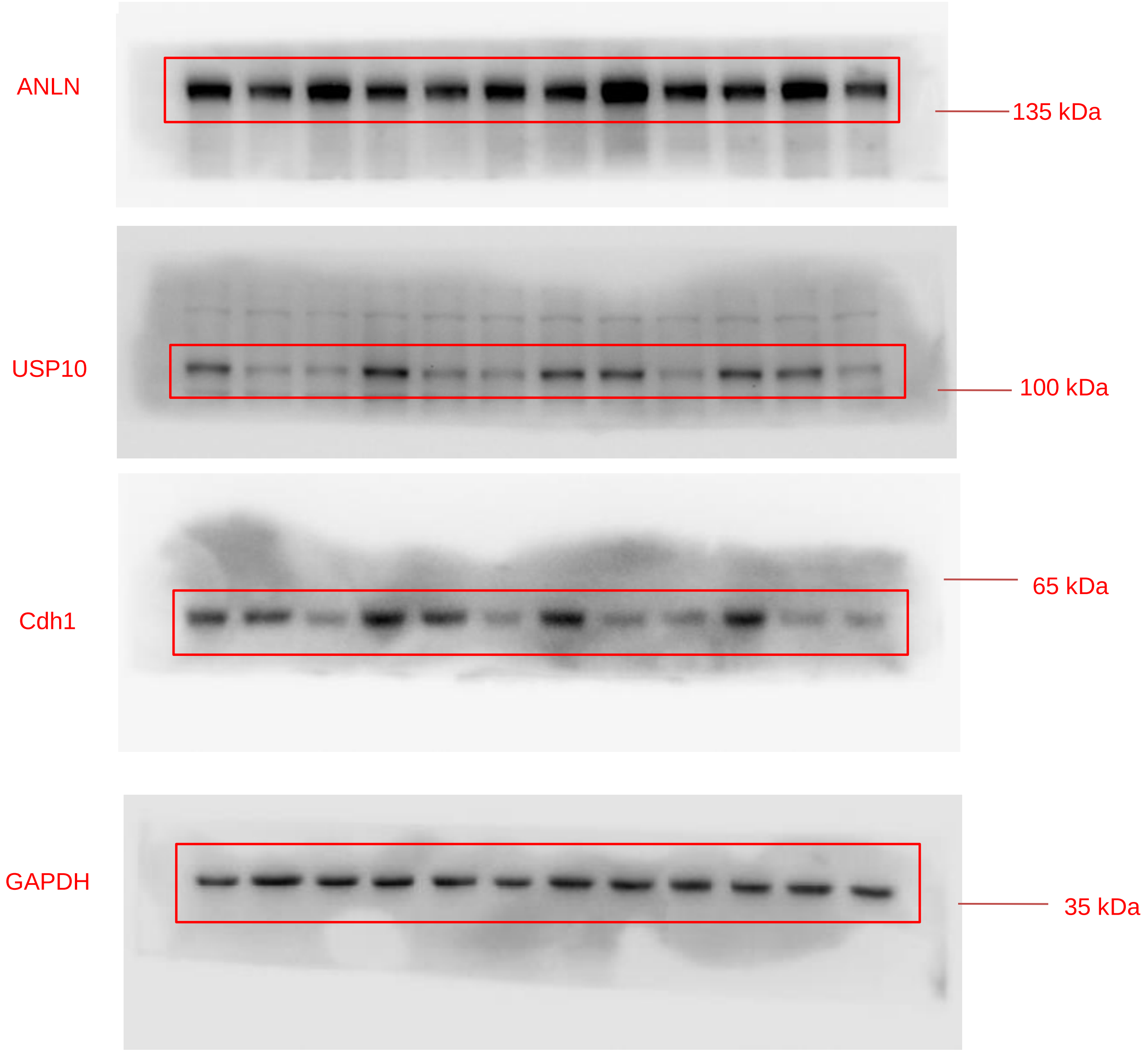

Fig.5J

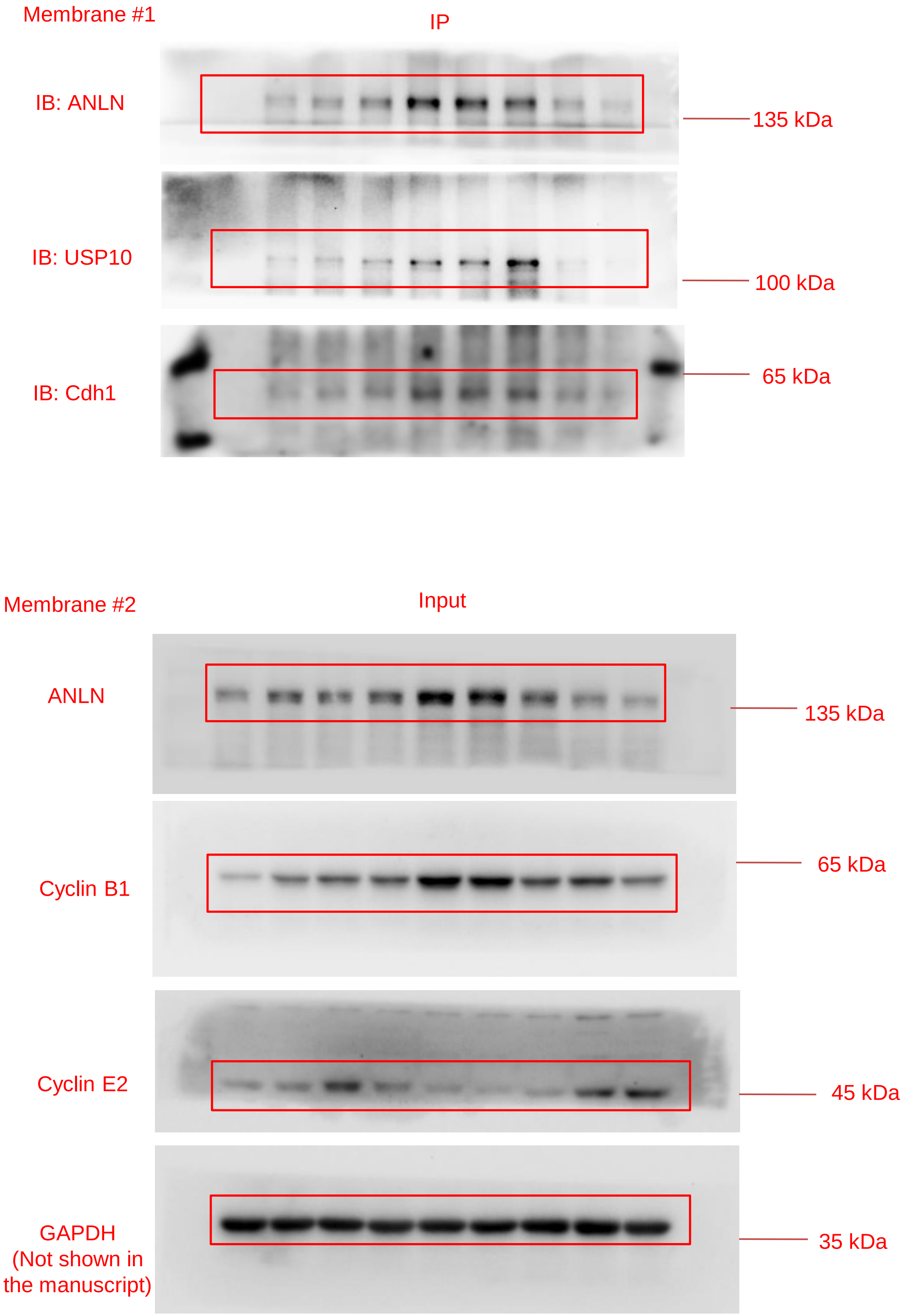

Membrane #1
IP
IB: ANLN
135 kDa
IB: USP10
100 kDa
IB: Cdh1
65 kDa
Membrane #2
Input
ANLN
135 kDa
Cyclin B1
65 kDa
Cyclin E2
45 kDa
GAPDH
(Not shown in
the manuscript)
35 kDa

Fig.5J

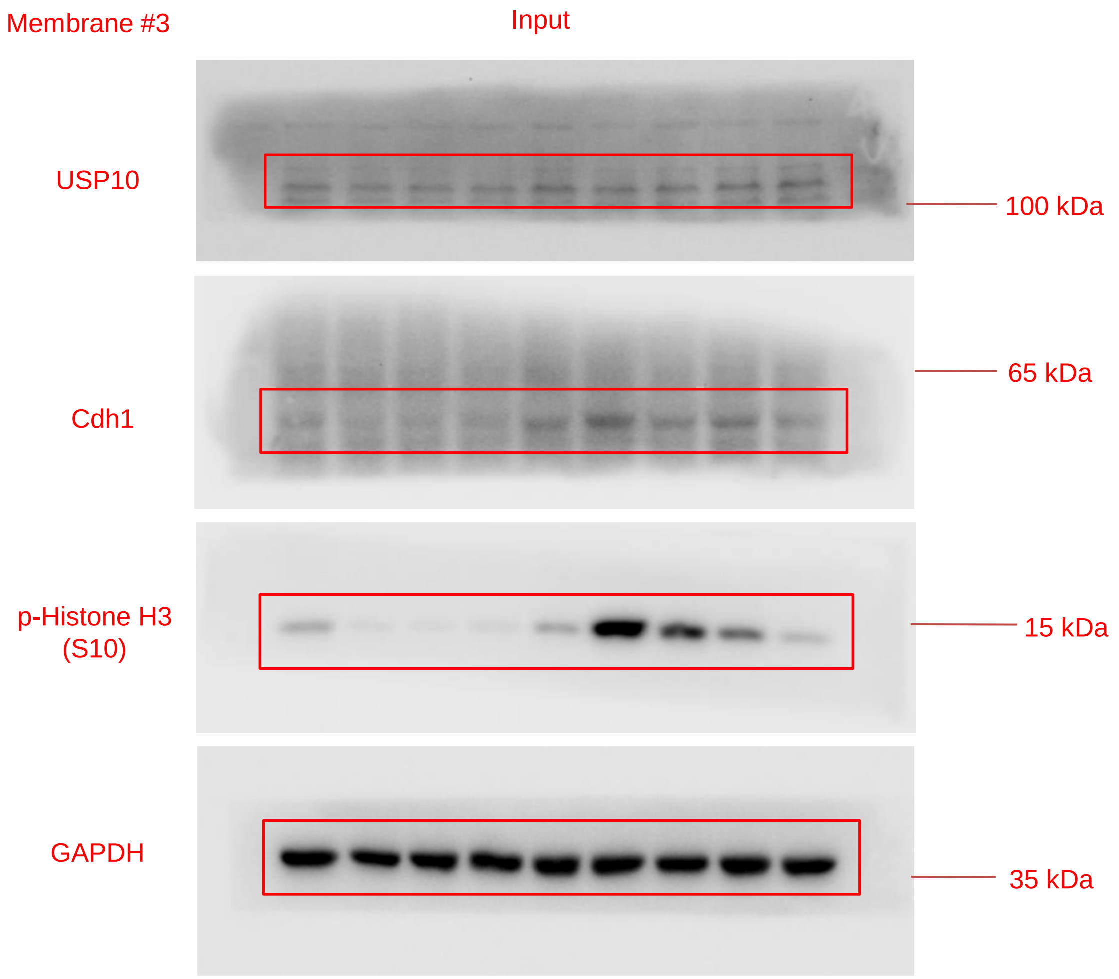
Membrane #3
Input
USP10
Cdh1
p-Histone H3
(S10)
GAPDH
100 kDa
65 kDa
15 kDa
35 kDa

Fig.5K

Membrane #1

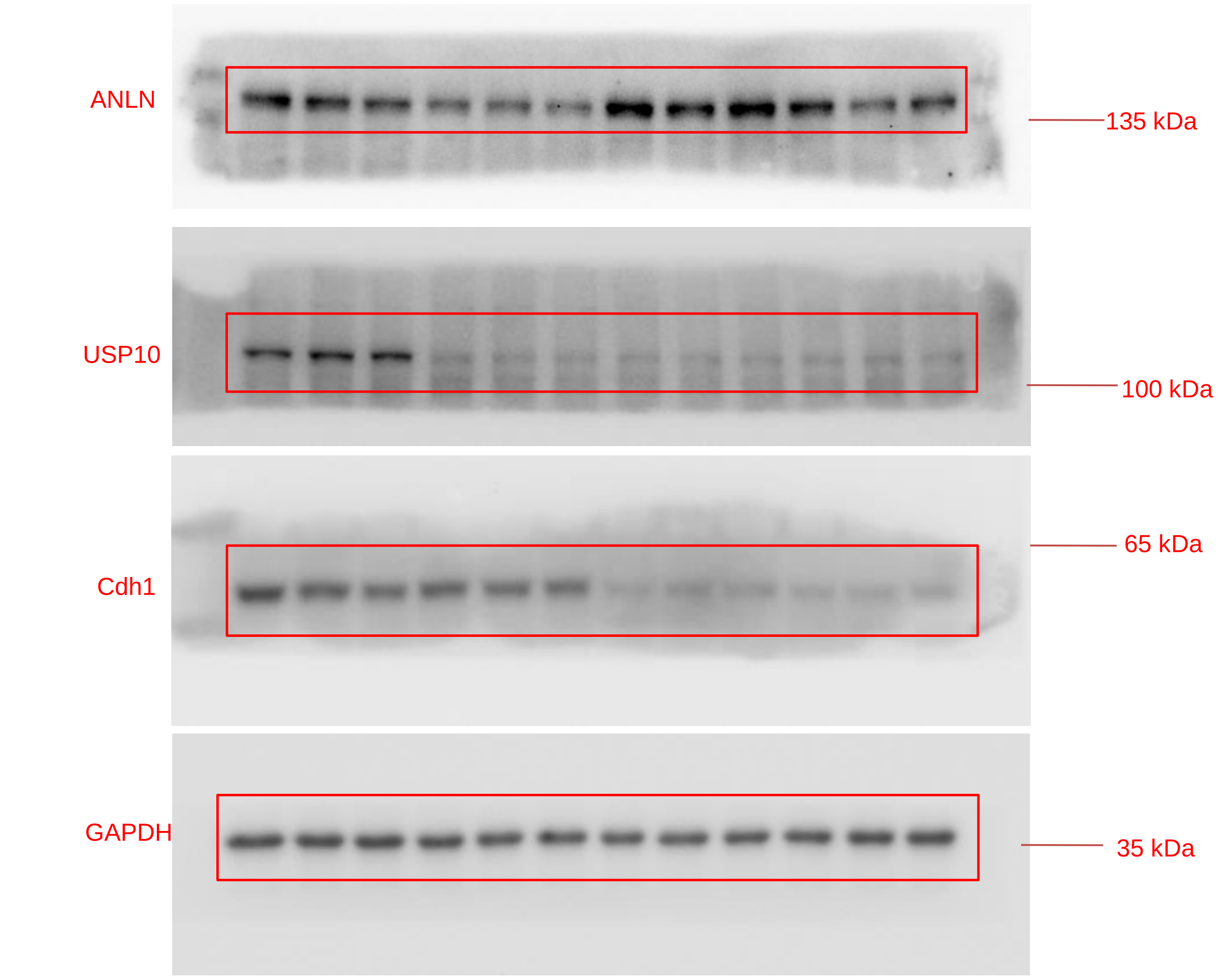


Membrane #2

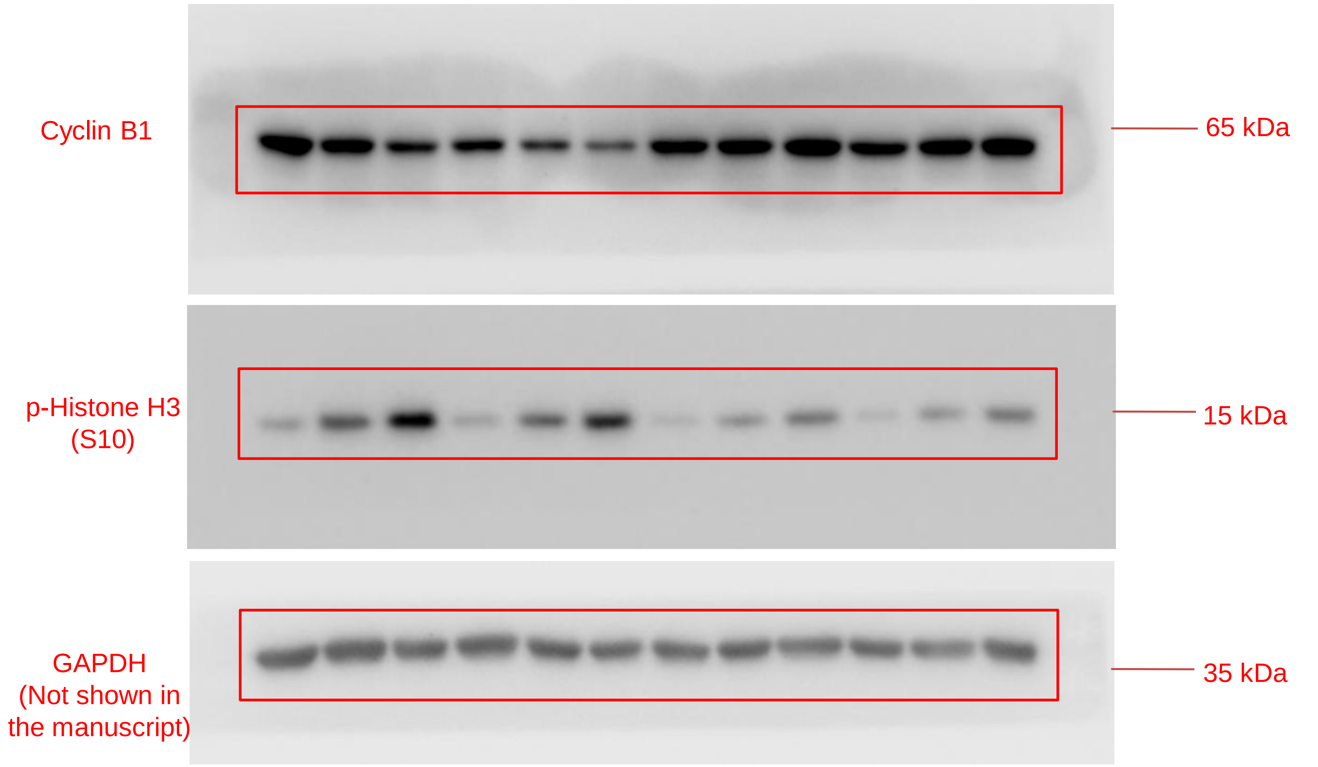

Fig.5K

Membrane #3

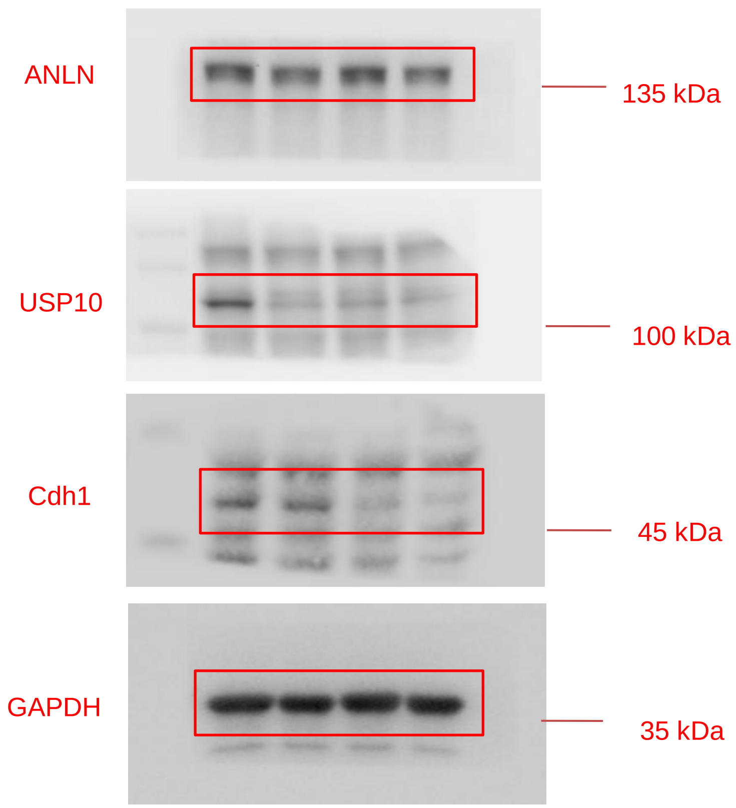


Fig.5L

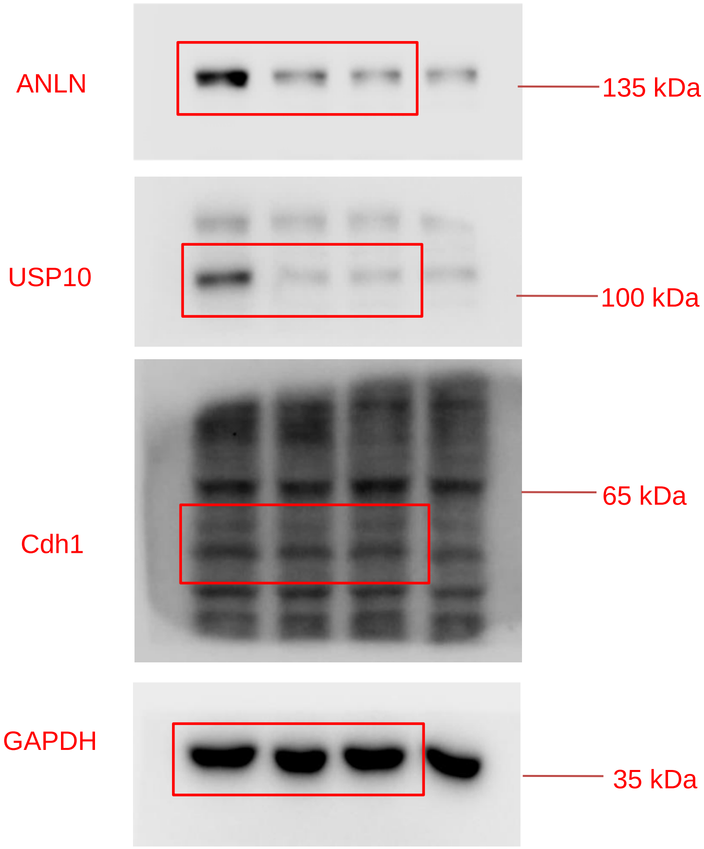

Fig.5M

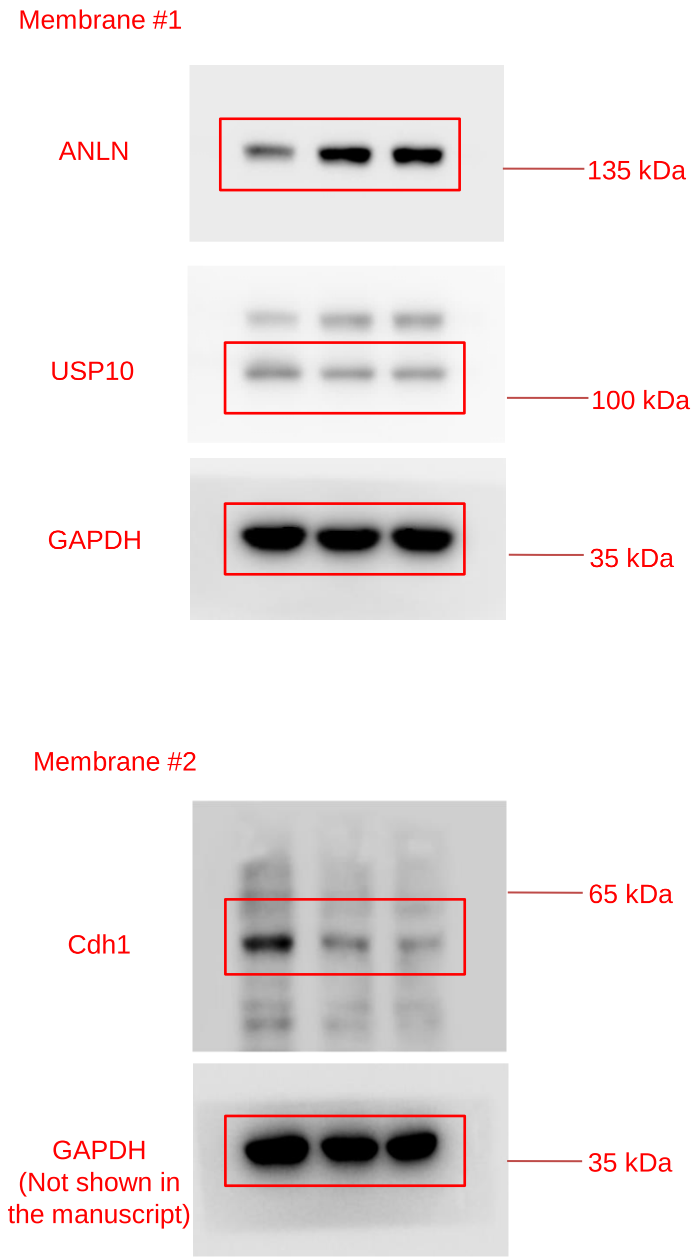

Membrane #1
ANLN
135 kDa
USP10
100 kDa
GAPDH
35 kDa
Membrane #2
Cdh1
65 kDa
GAPDH
(Not shown in
the manuscript)
35 kDa

Fig.5N

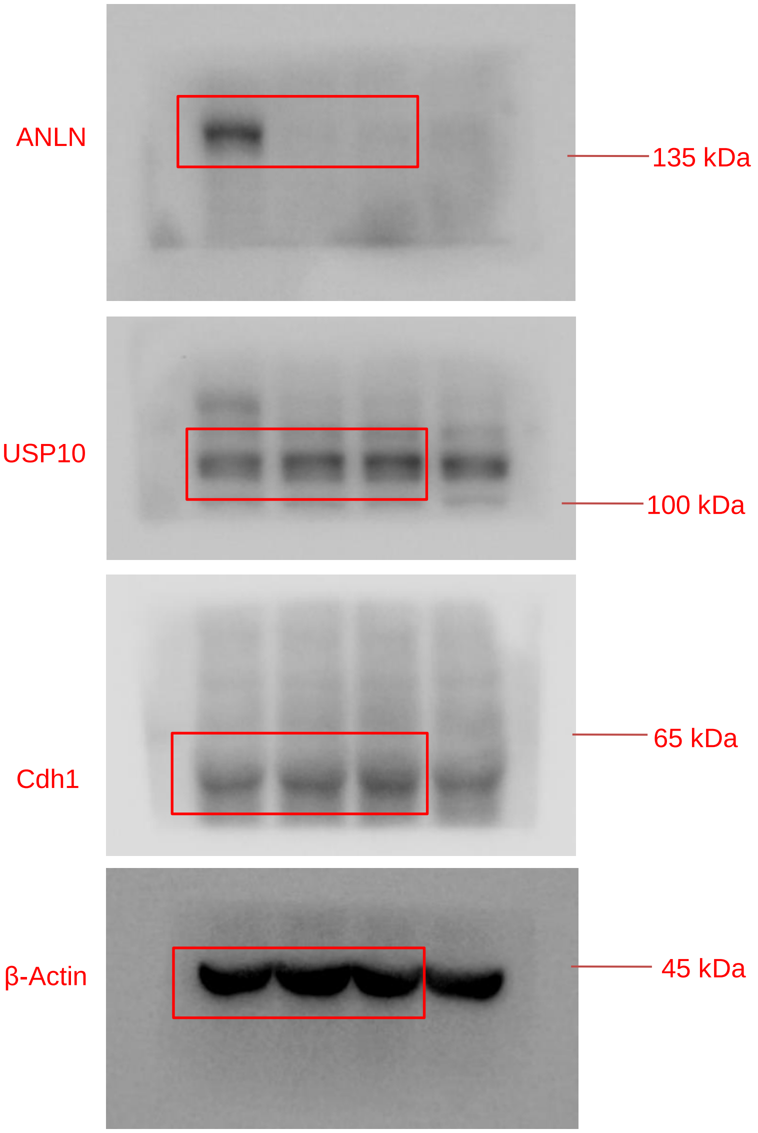
ANLN
135 kDa
USP10
100 kDa
Cdh1
65 kDa
β-Actin
45 kDa

Fig.6B

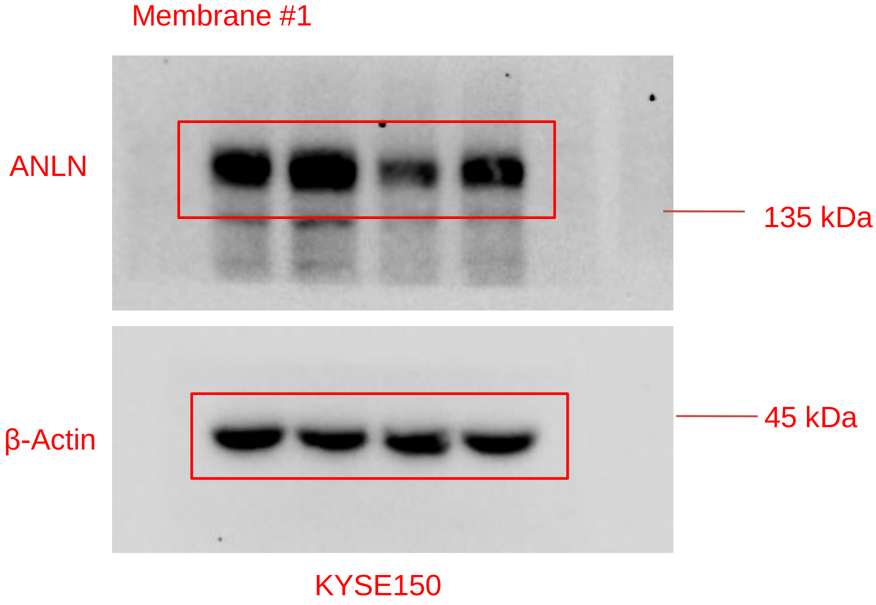
Membrane #1
ANLN
135 kDa
β-Actin
45 kDa
KYSE150

Fig.6B

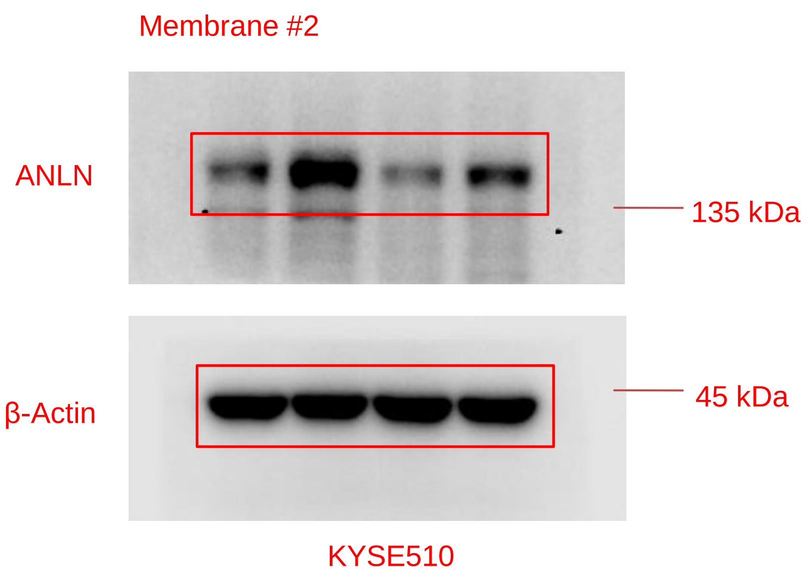
Membrane #2
ANLN
135 kDa
β-Actin
45 kDa
KYSE510

Fig.6C

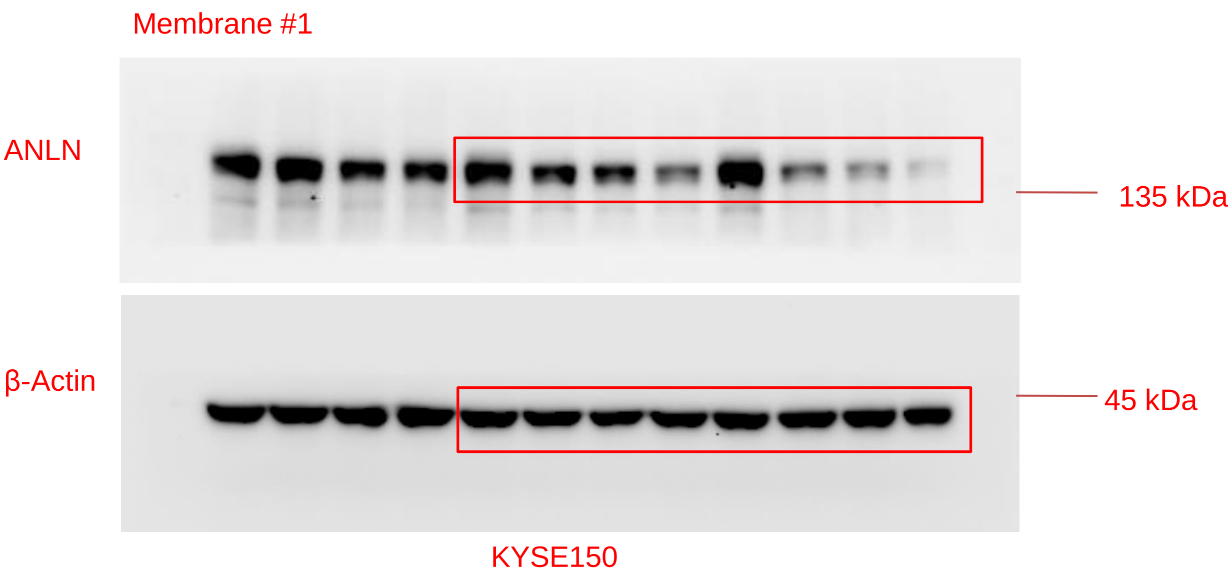
Membrane #1
ANLN
135 kDa
β-Actin
45 kDa
KYSE150

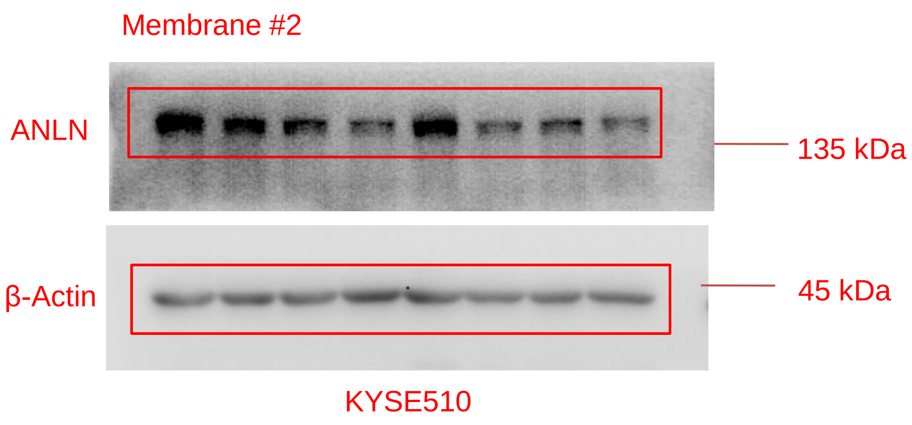
Membrane #2
ANLN
135 kDa
β-Actin
45 kDa
KYSE510

Fig.6D

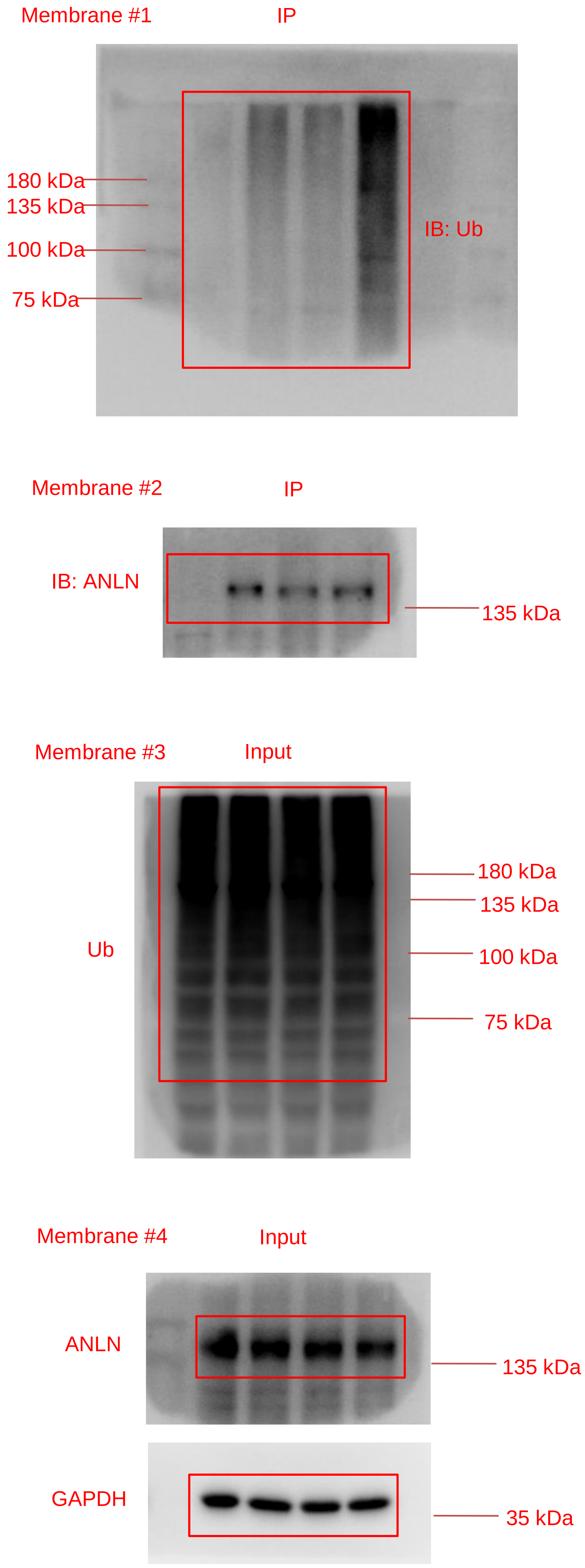

Membrane #1
IP
180 kDa
135 kDa
100 kDa
75 kDa
IB: Ub
Membrane #2
IP
IB: ANLN
135 kDa
Membrane #3
Input
Ub
180 kDa
135 kDa
100 kDa
75 kDa
Membrane #4
Input
ANLN
135 kDa
GAPDH
35 kDa

Fig.6E

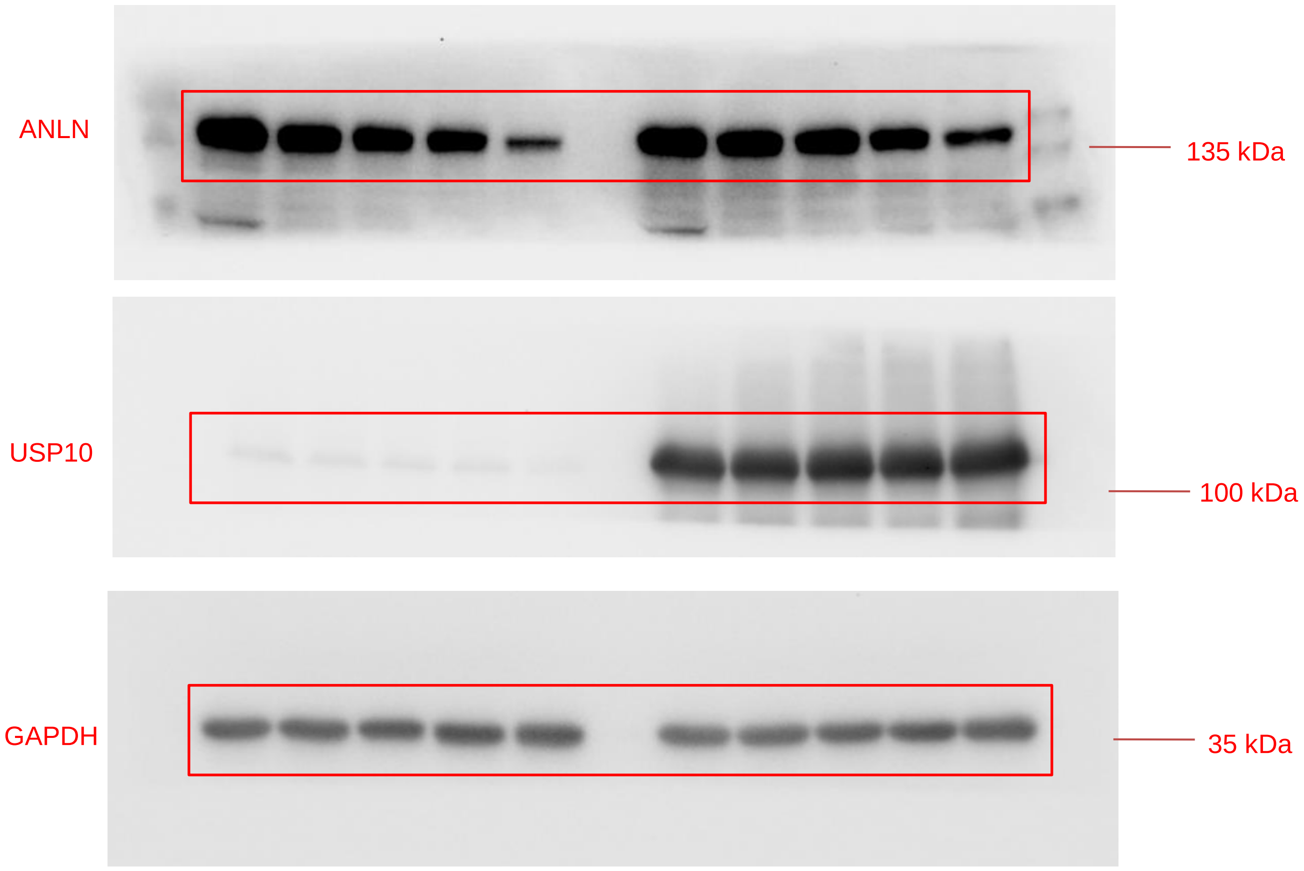
ANLN
135 kDa
USP10
100 kDa
GAPDH
35 kDa

Fig.6F

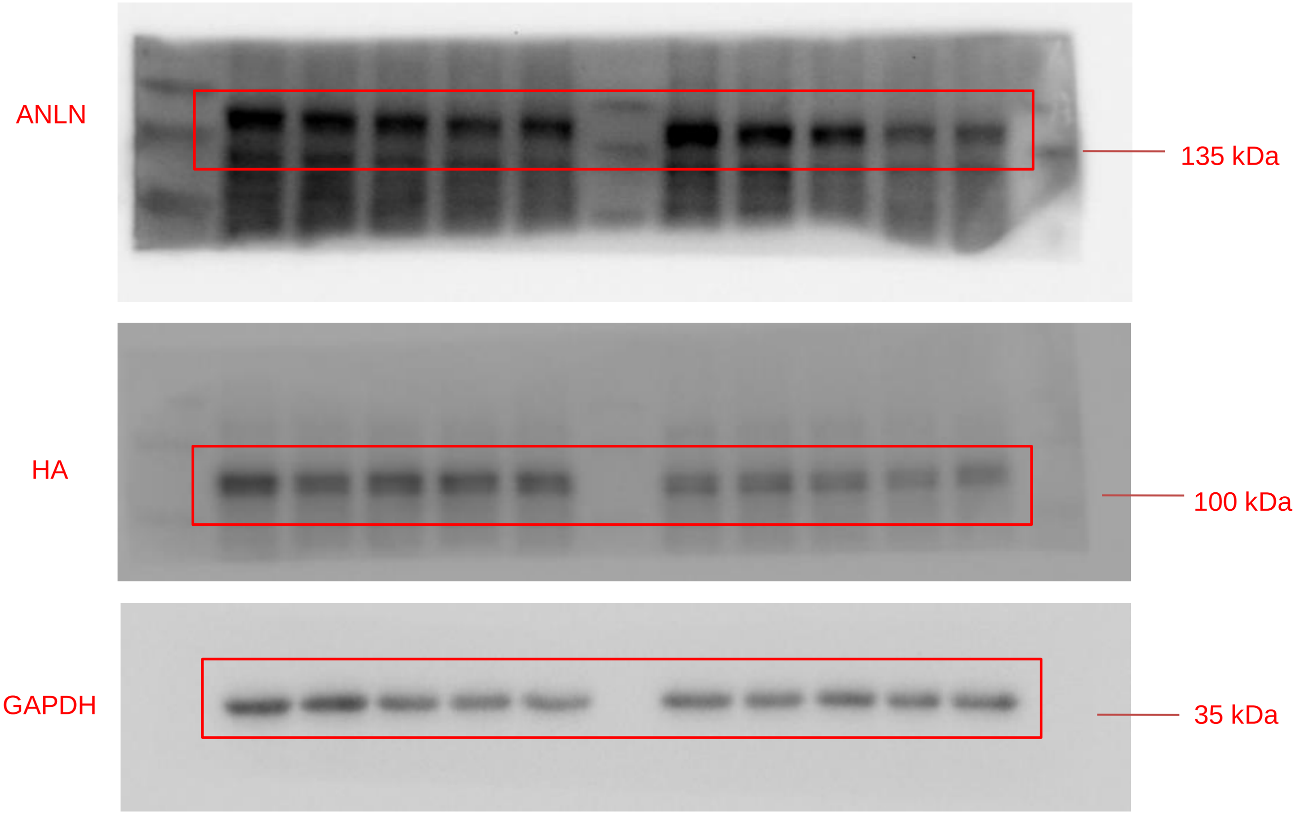
ANLN
135 kDa
HA
100 kDa
GAPDH
35 kDa

Fig.6G

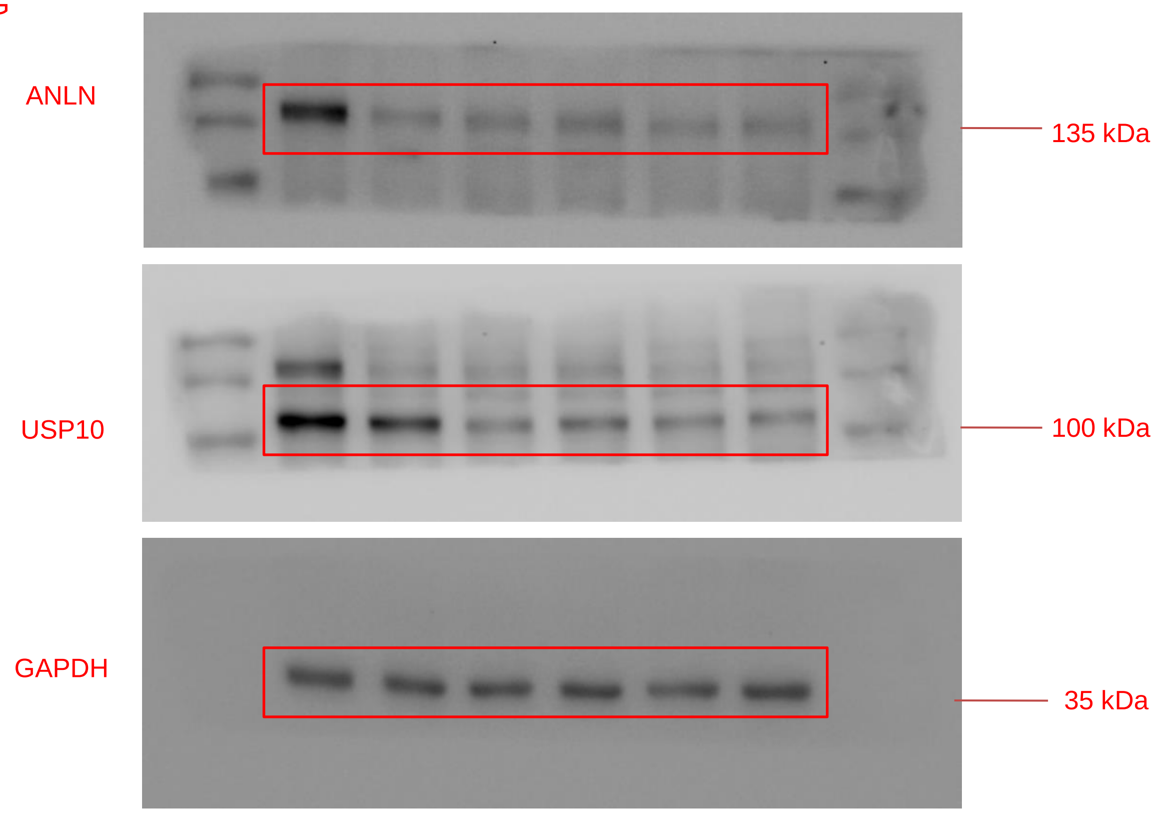

ANLN
135 kDa
USP10
100 kDa
GAPDH
35 kDa


Fig.6H

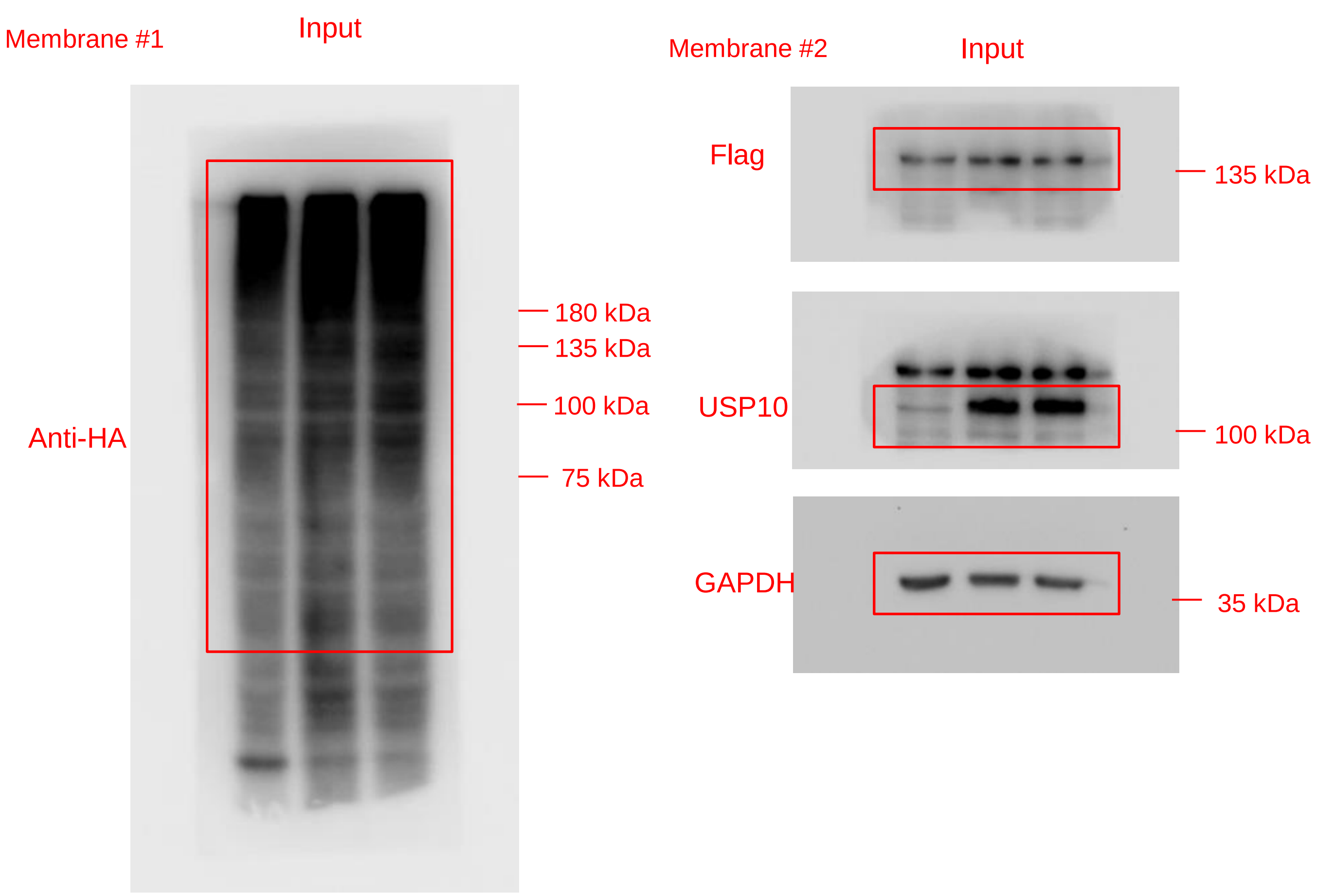

Membrane #1
Input
Anti-HA
180 kDa
135 kDa
100 kDa
75 kDa
Membrane #2
Input
Flag
135 kDa
USP10
100 kDa
GAPDH
35 kDa

Fig.6H

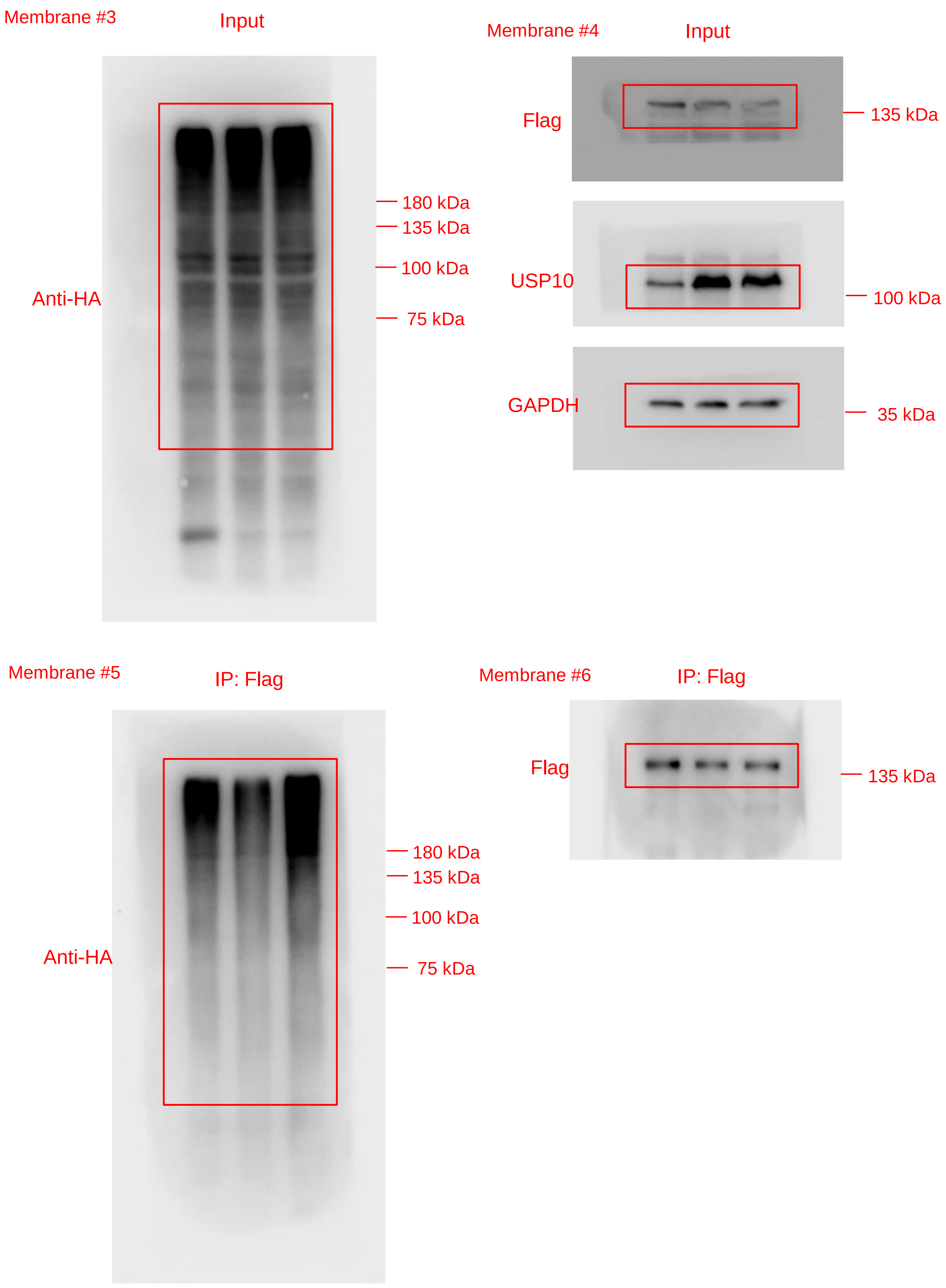
Membrane #3
Input
Anti-HA
180 kDa
135 kDa
100 kDa
75 kDa
Membrane #4
Input
Flag
135 kDa
USP10
100 kDa
GAPDH
35 kDa
Membrane #5
IP: Flag
Anti-HA
180 kDa
135 kDa
100 kDa
75 kDa
Membrane #6
IP: Flag
Flag
135 kDa

Fig.6H

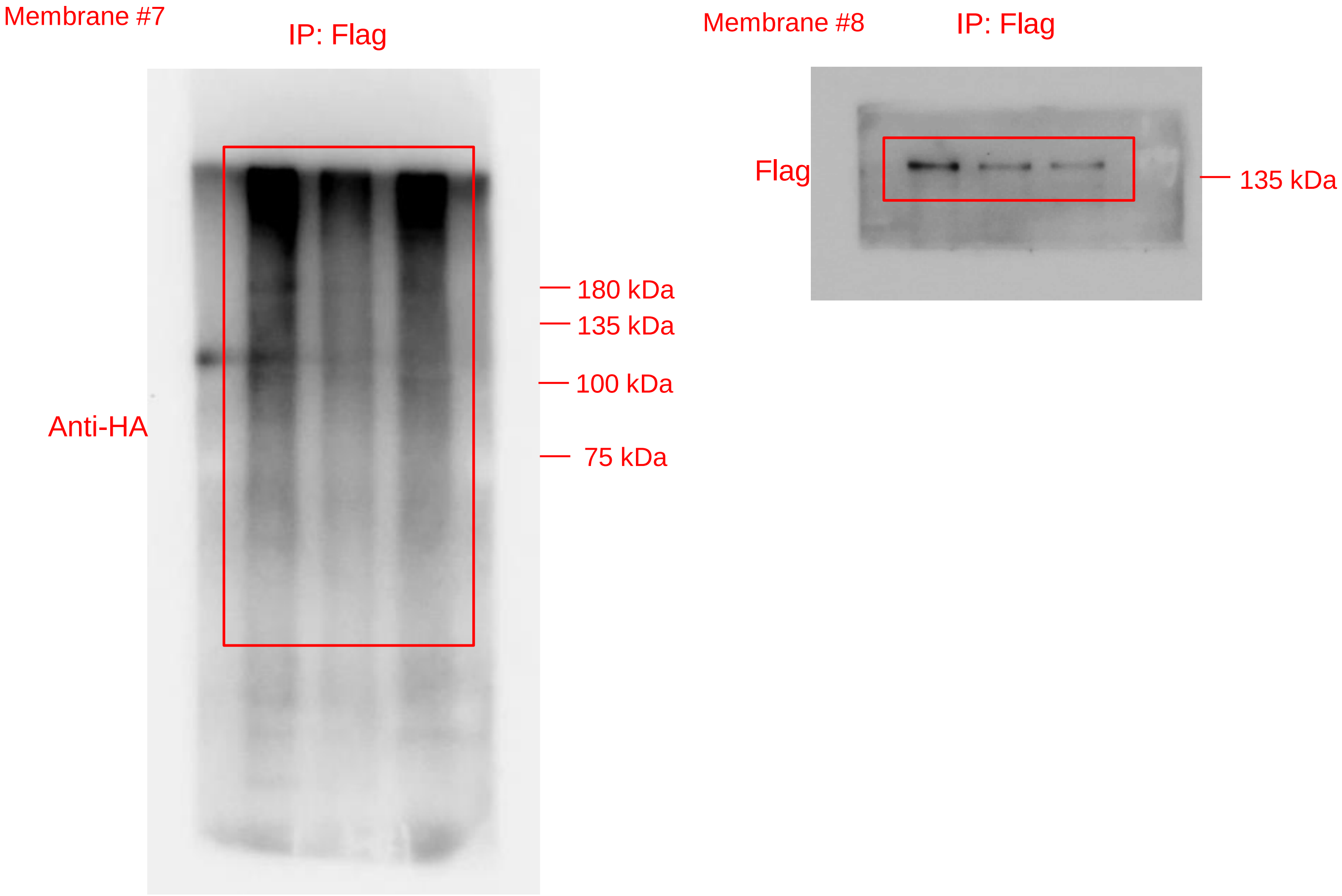

Membrane #7
IP: Flag
Anti-HA
180 kDa
135 kDa
100 kDa
75 kDa
Membrane #8
IP: Flag
Flag
135 kDa


Fig.6I

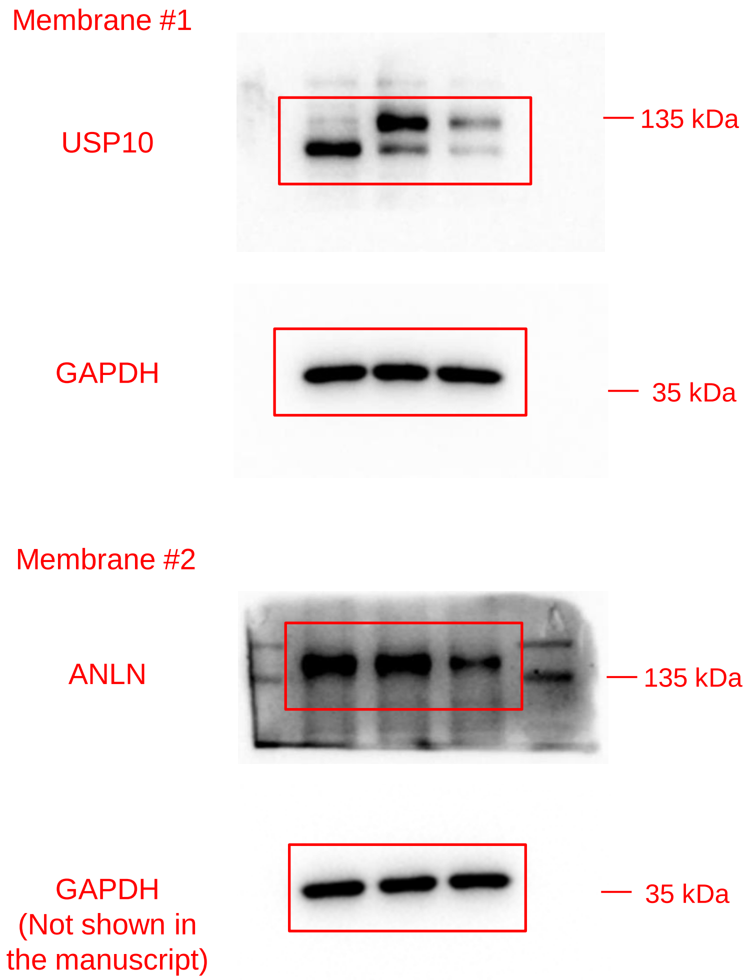

Membrane #1
USP10
135 kDa
GAPDH
35 kDa
Membrane #2
ANLN
135 kDa
GAPDH
(Not shown in
the manuscript)
35 kDa

Fig.6J

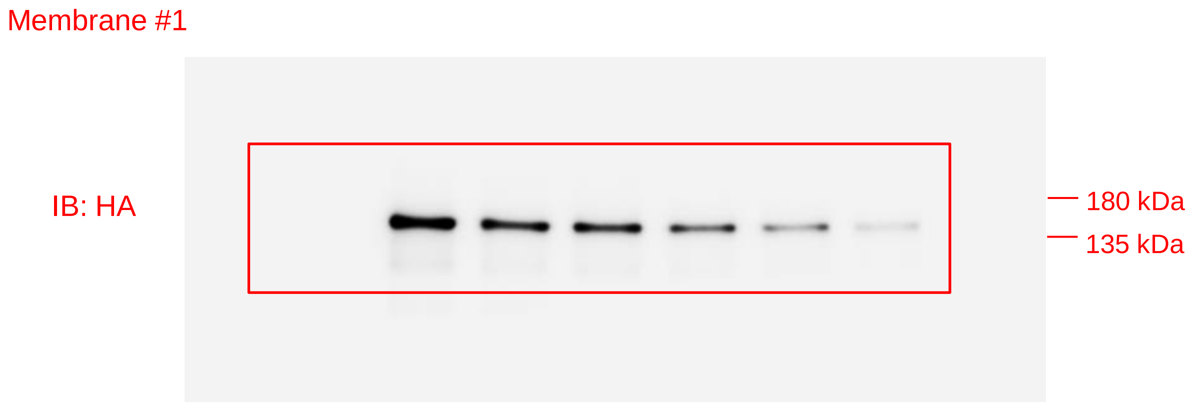


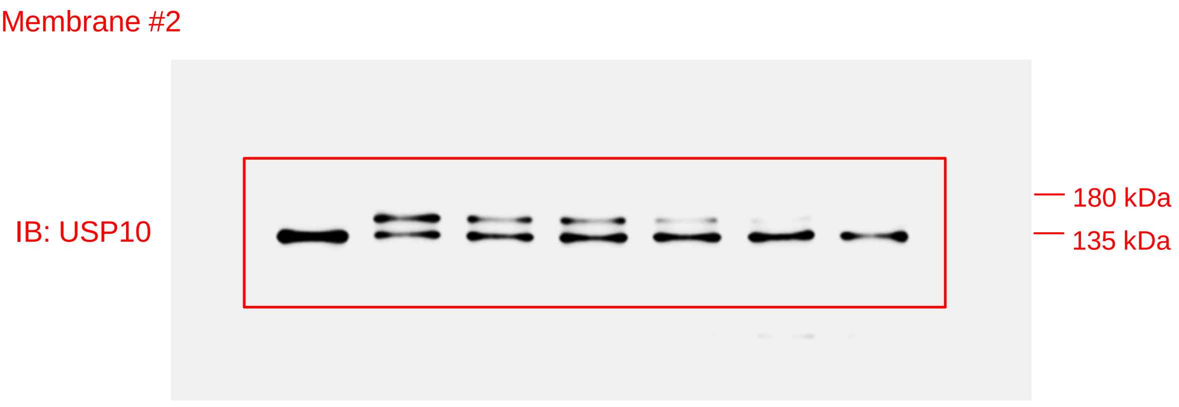


Fig.6K

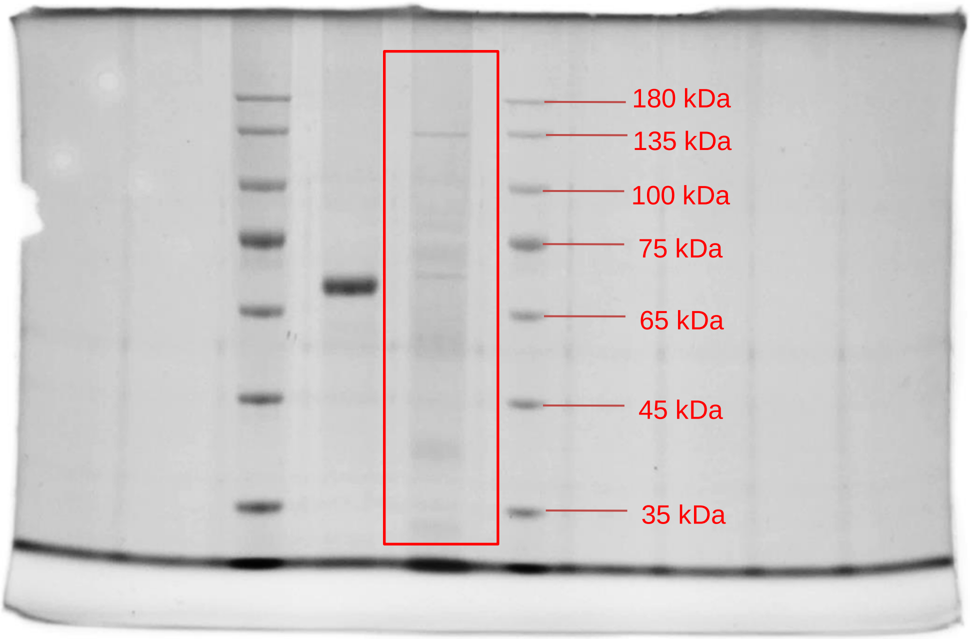


CBB

Fig.7B

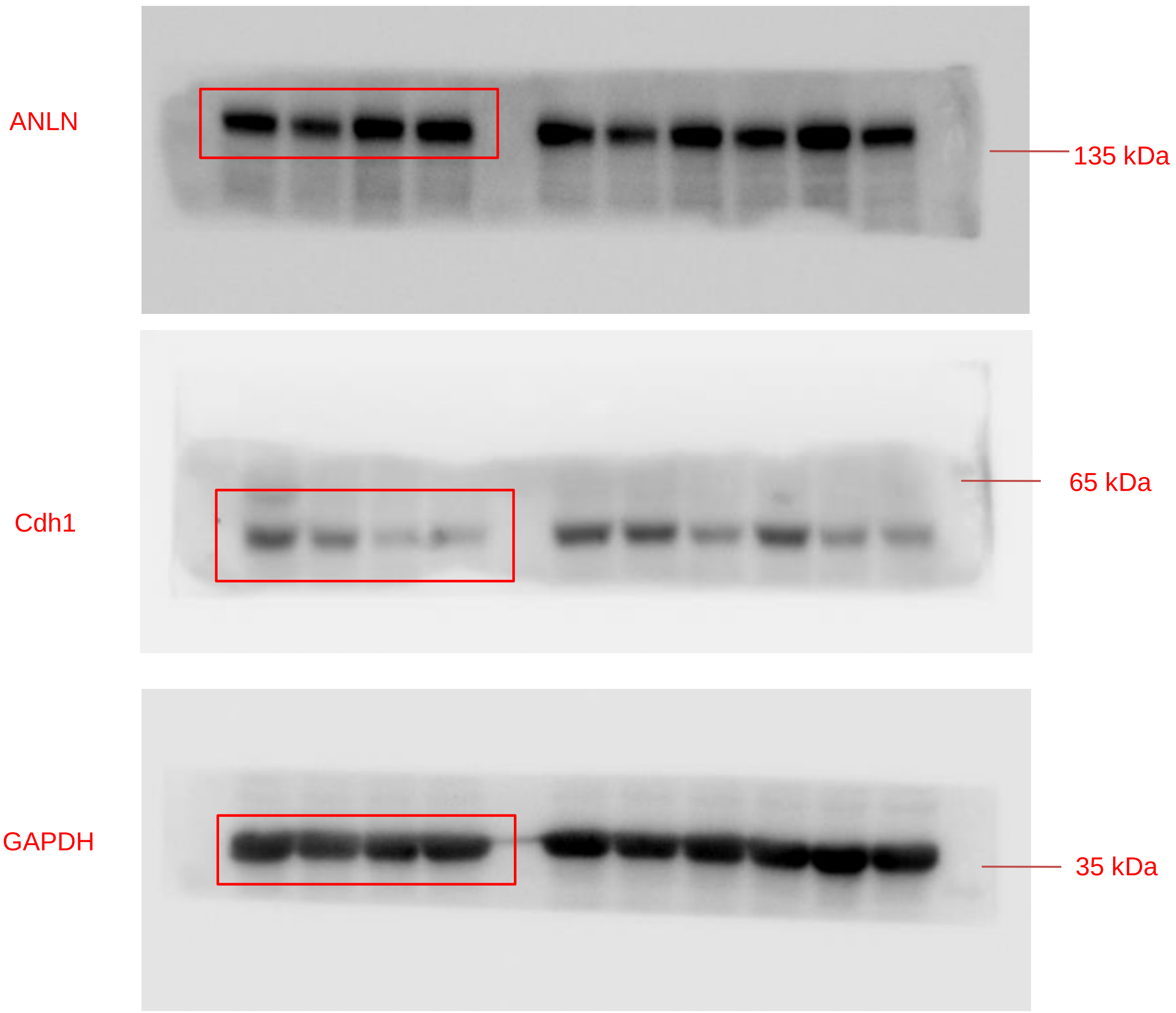

Fig.7C

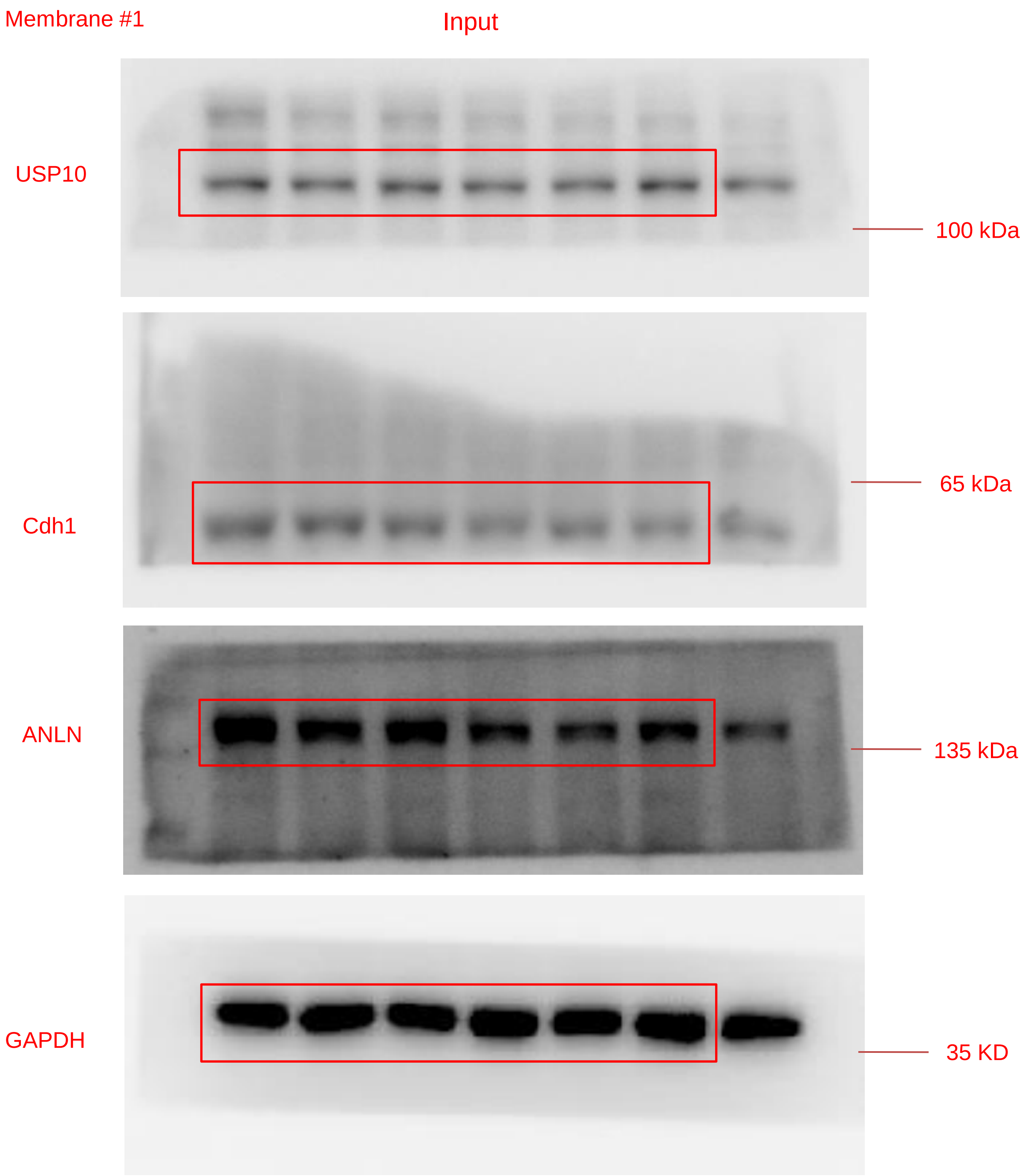

Membrane #1
Input
USP10
100 kDa
Cdh1
65 kDa
ANLN
135 kDa
GAPDH
35 KD

Fig.7C

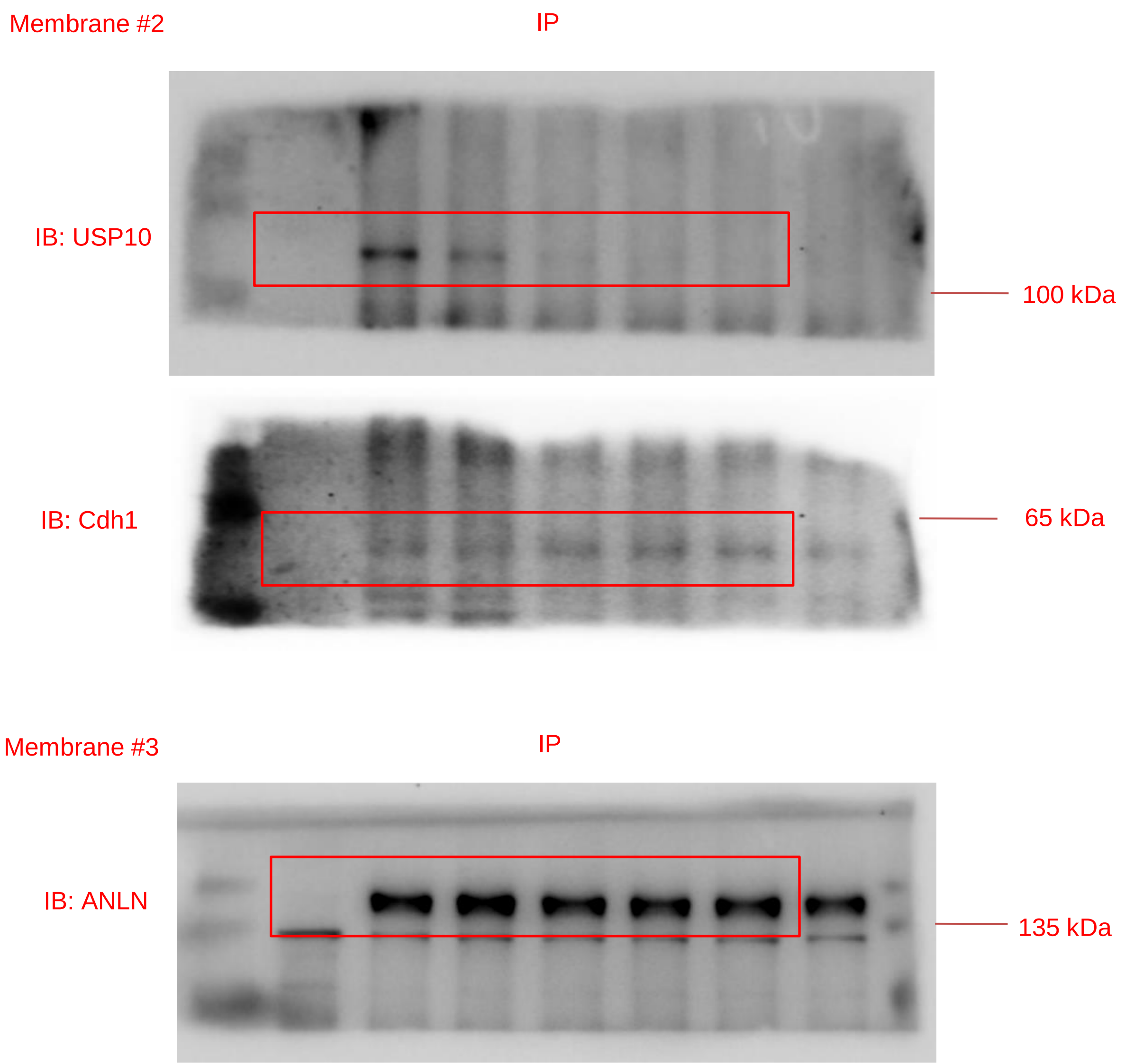

Membrane #2
IP
IB: USP10
100 kDa
IB: Cdh1
65 kDa
Membrane #3
IP
IB: ANLN
135 kDa

Fig.7D

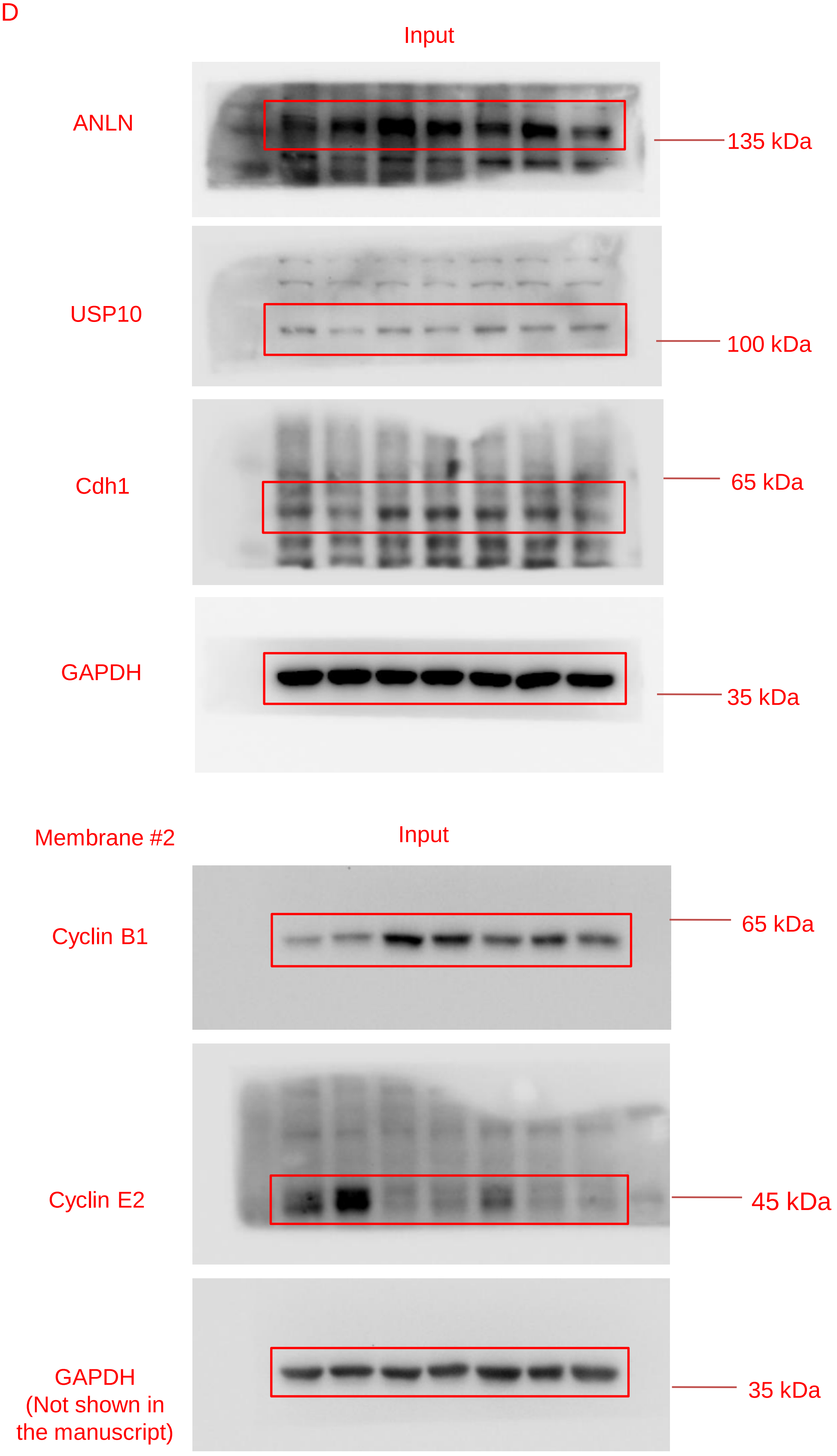
Input
ANLN
135 kDa
USP10
100 kDa
Cdh1
65 kDa
GAPDH
35 kDa
Membrane #2
Input
Cyclin B1
65 kDa
Cyclin E2
45 kDa
GAPDH
(Not shown in
the manuscript)
35 kDa

Fig.7D

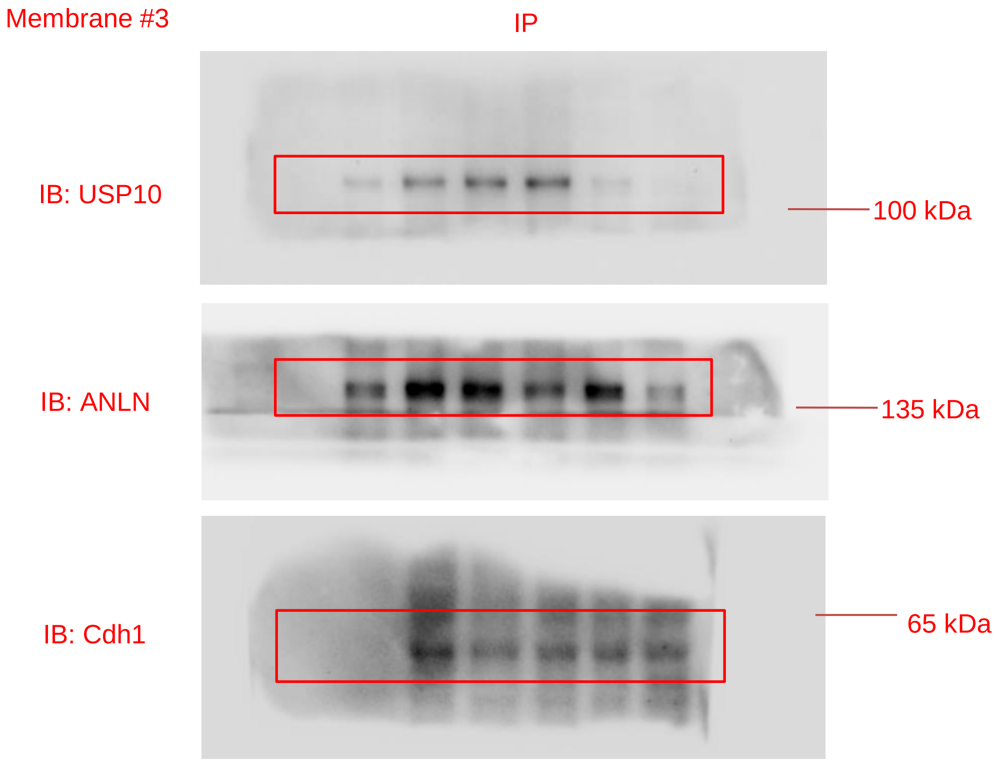
Membrane #3
IP
IB: USP10
100 kDa
IB: ANLN
135 kDa
IB: Cdh1
65 kDa

Fig.7E

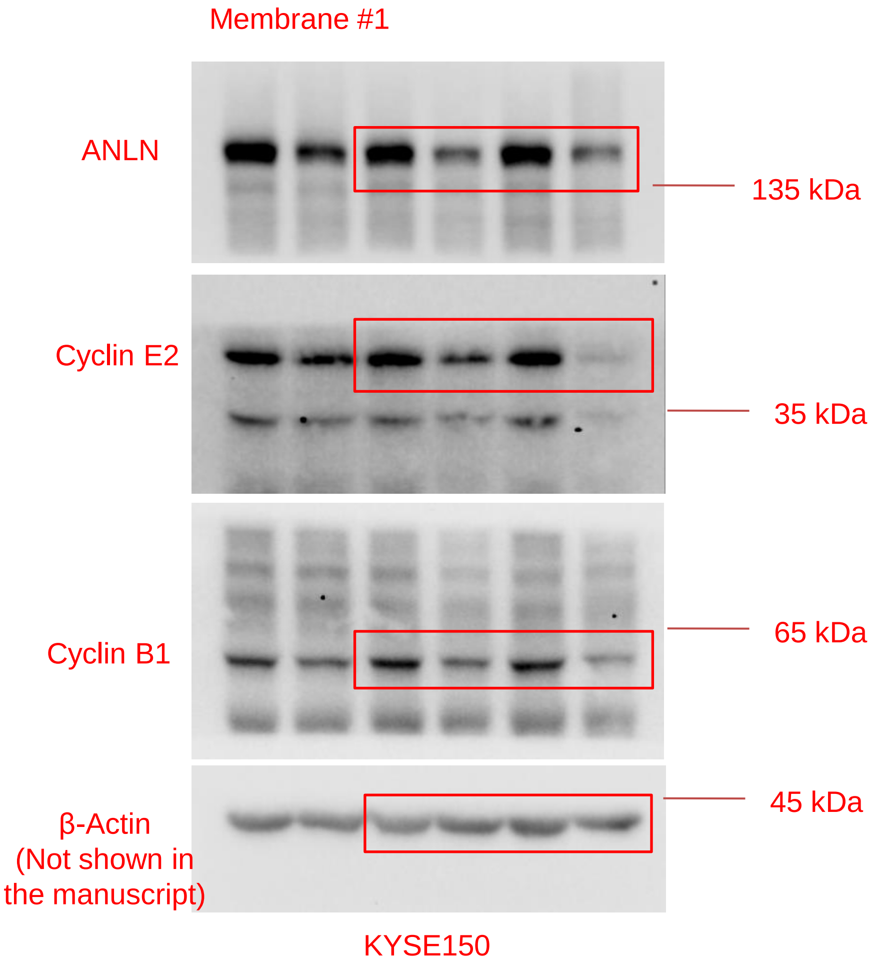
Membrane #1
ANLN
135 kDa
Cyclin E2
35 kDa
Cyclin B1
65 kDa
β-Actin
(Not shown in
the manuscript)
45 kDa
KYSE150

Fig.7E

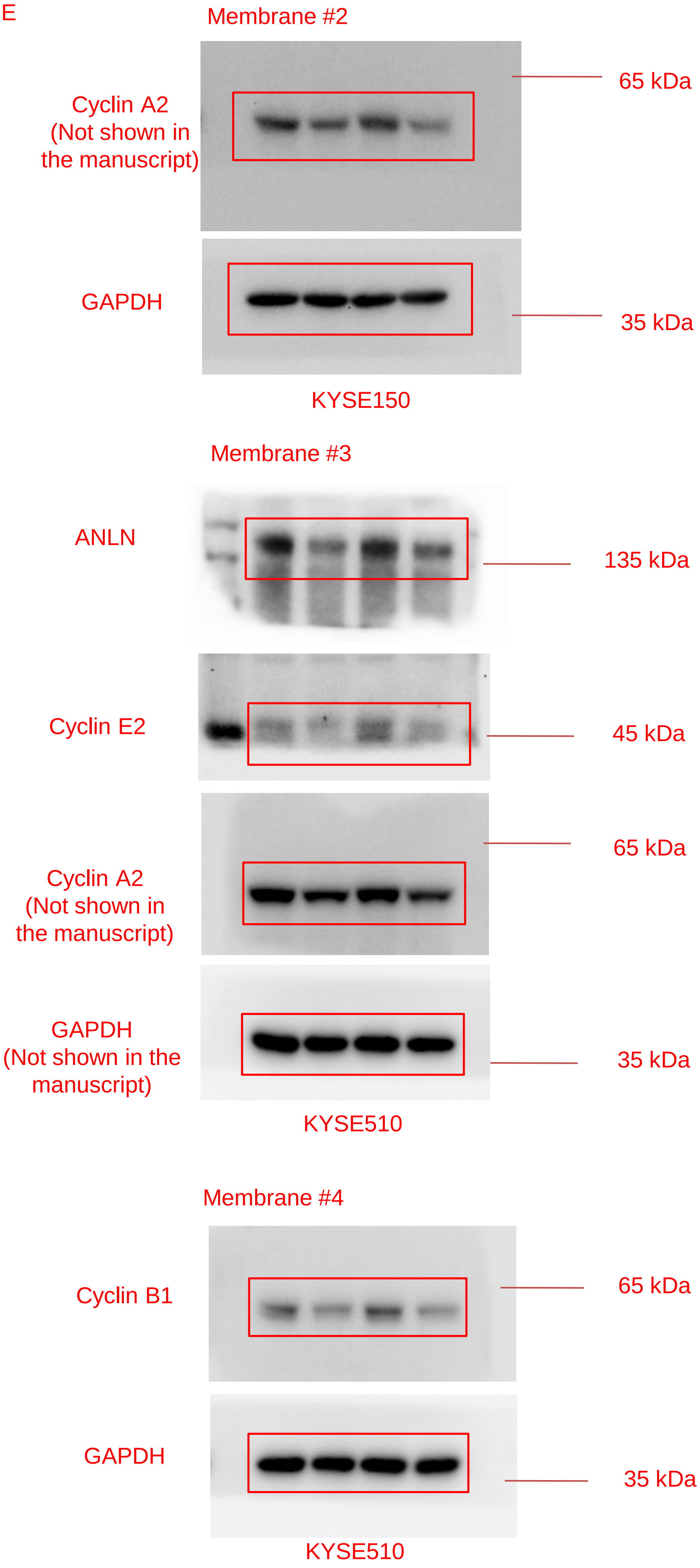
Membrane #2
Cyclin A2
(Not shown in
the manuscript)
65 kDa
GAPDH
35 kDa
KYSE150
Membrane #3
ANLN
135 kDa
Cyclin E2
45 kDa
Cyclin A2
(Not shown in
the manuscript)
65 kDa
GAPDH
(Not shown in the
manuscript)
35 kDa
KYSE510
Membrane #4
Cyclin B1
65 kDa
GAPDH
35 kDa
KYSE510

Fig.S1I

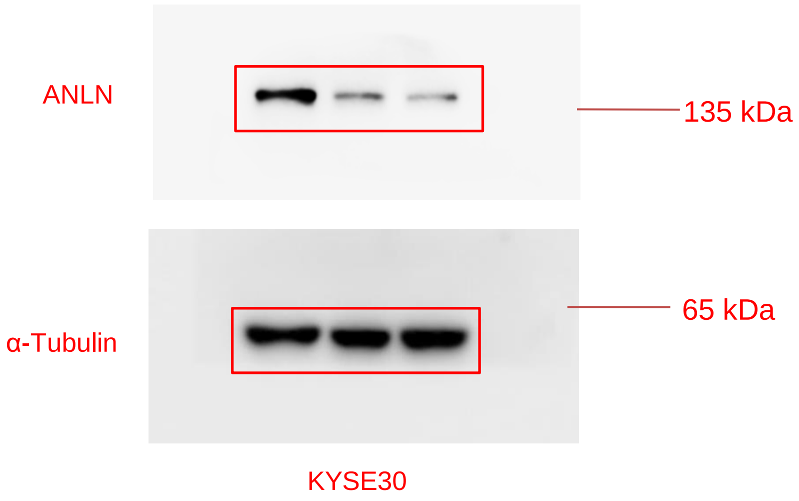


Fig.S1J

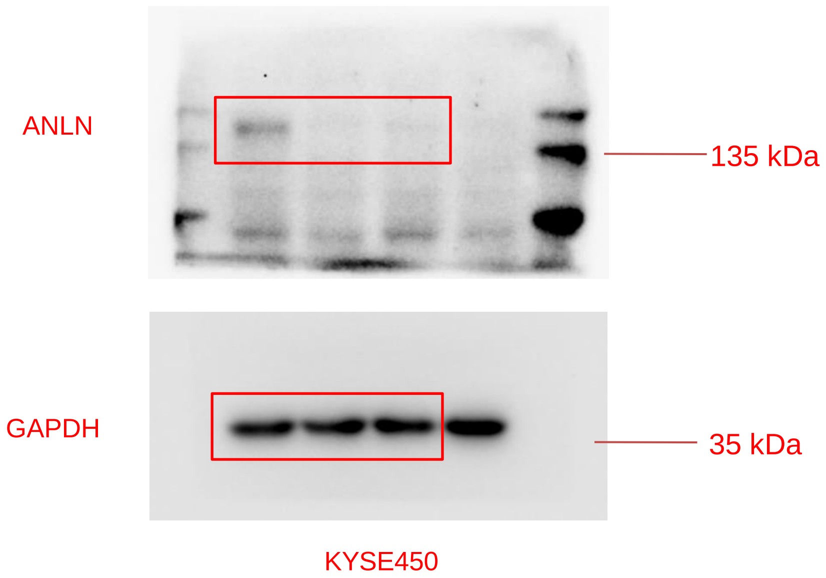

Fig.S2B

Membrane #1

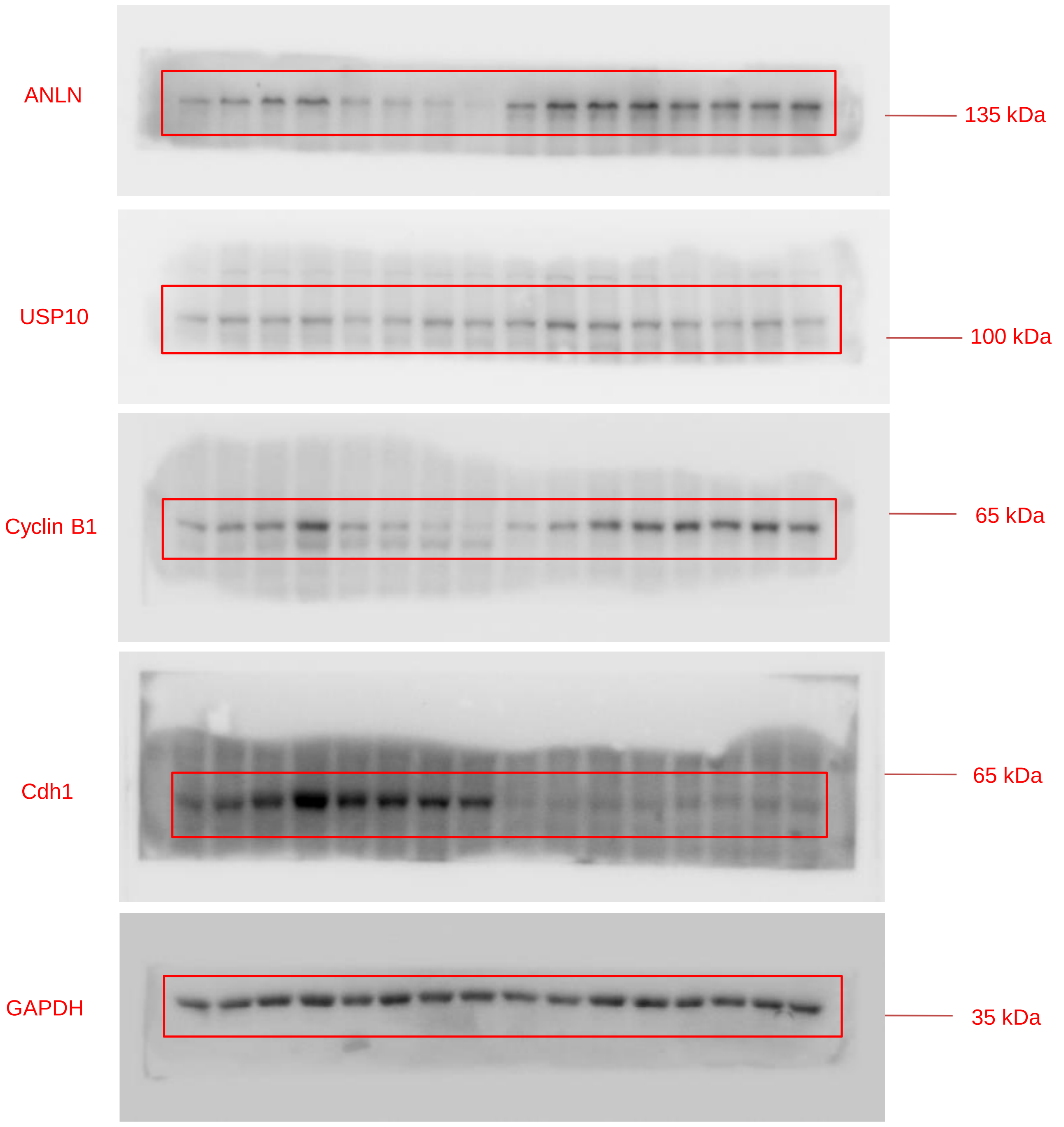

Fig.S2B

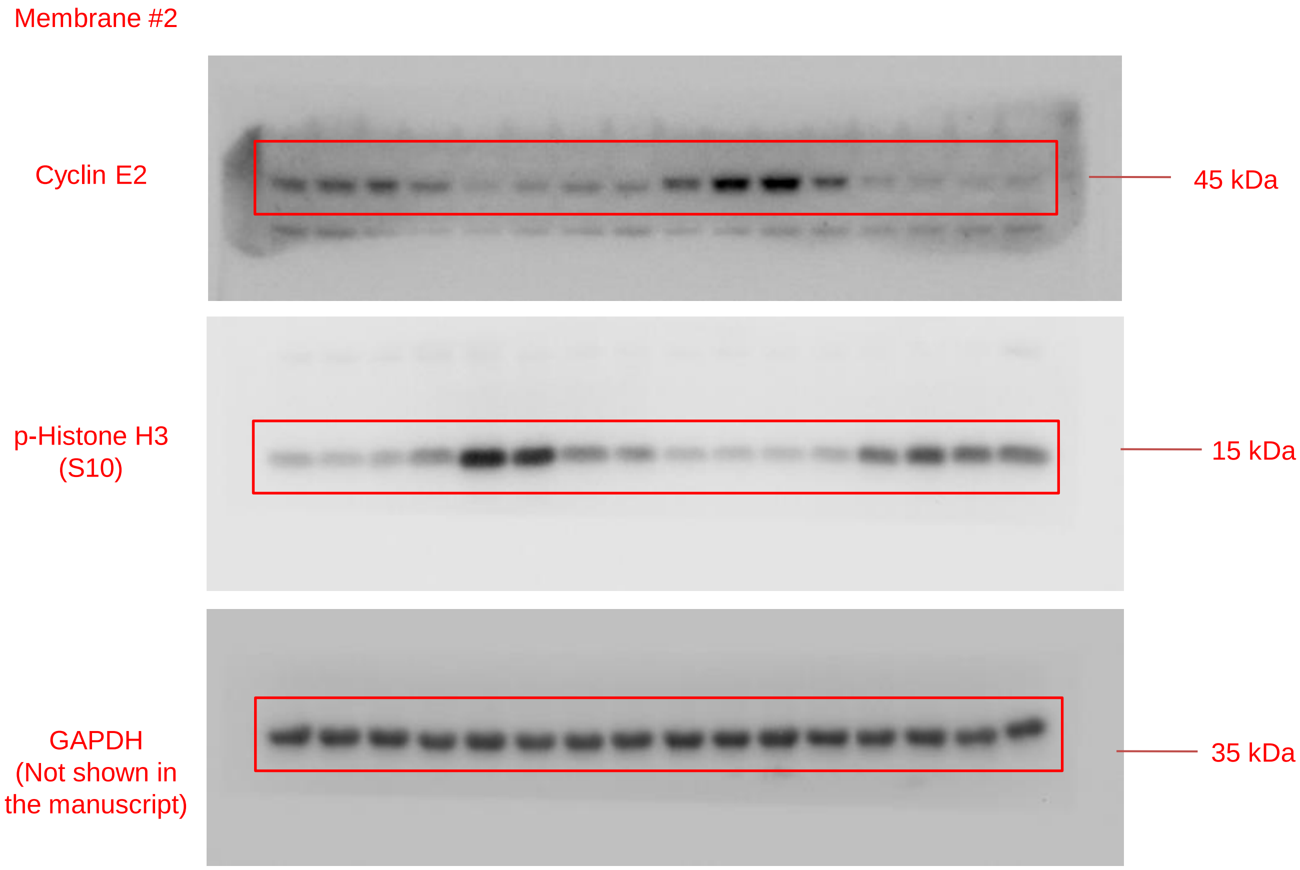


Fig.S3G

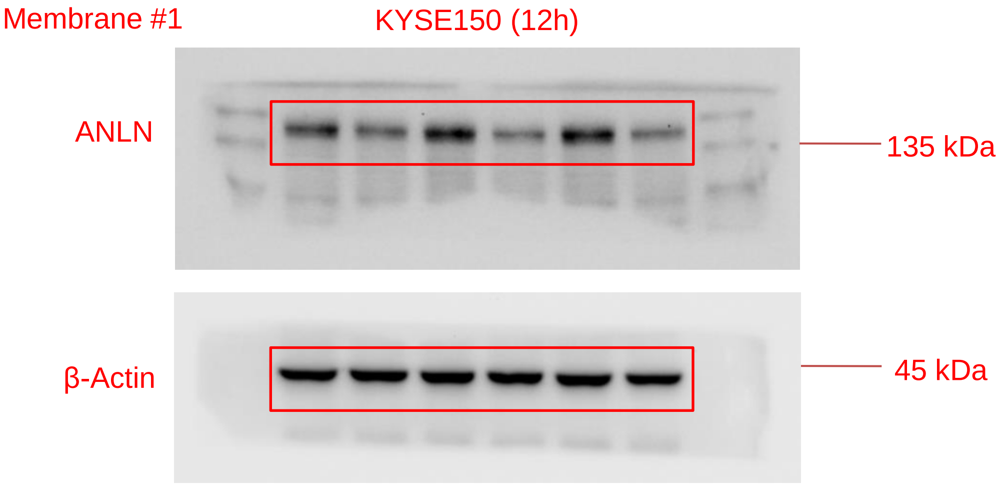

Fig.S3G

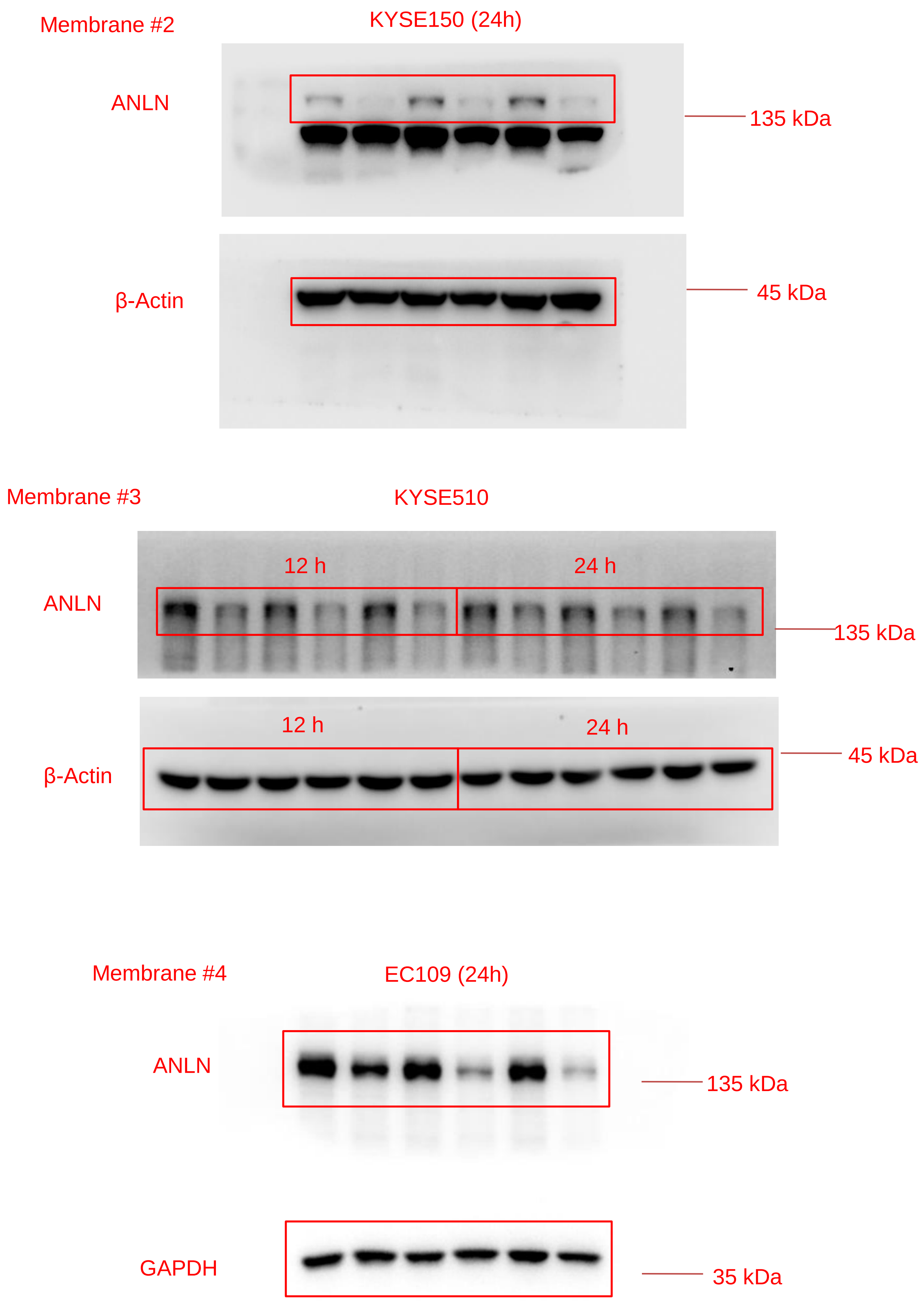

Membrane #2
KYSE150 (24h)
ANLN
135 kDa
β-Actin
45 kDa
Membrane #3
KYSE510
ANLN
12 h
24 h
135 kDa
β-Actin
12 h
24 h
45 kDa
Membrane #4
EC109 (24h)
ANLN
135 kDa
GAPDH
35 kDa

Fig.S3H

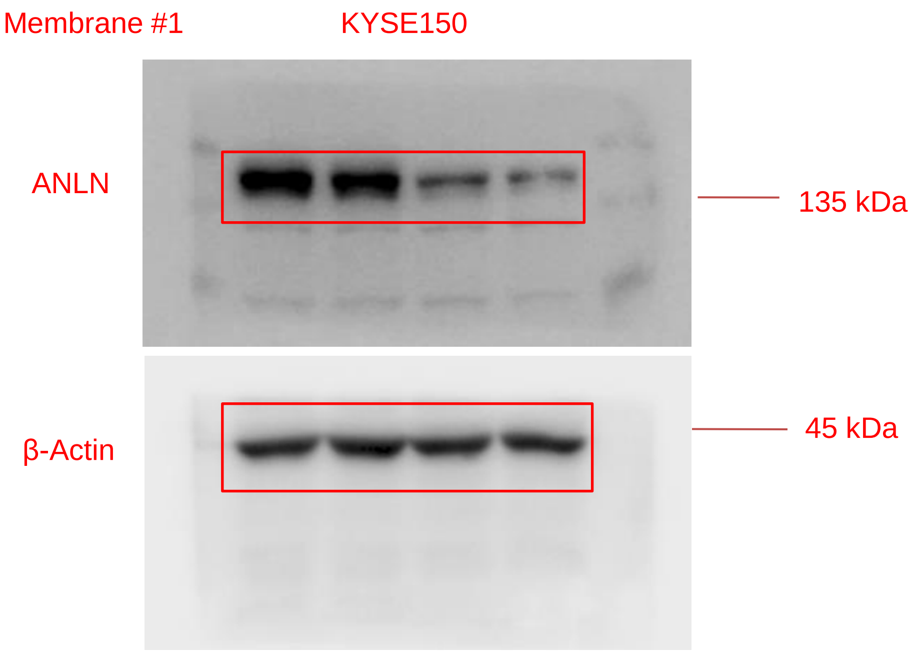
Membrane #1
KYSE150
ANLN
135 kDa
β-Actin
45 kDa

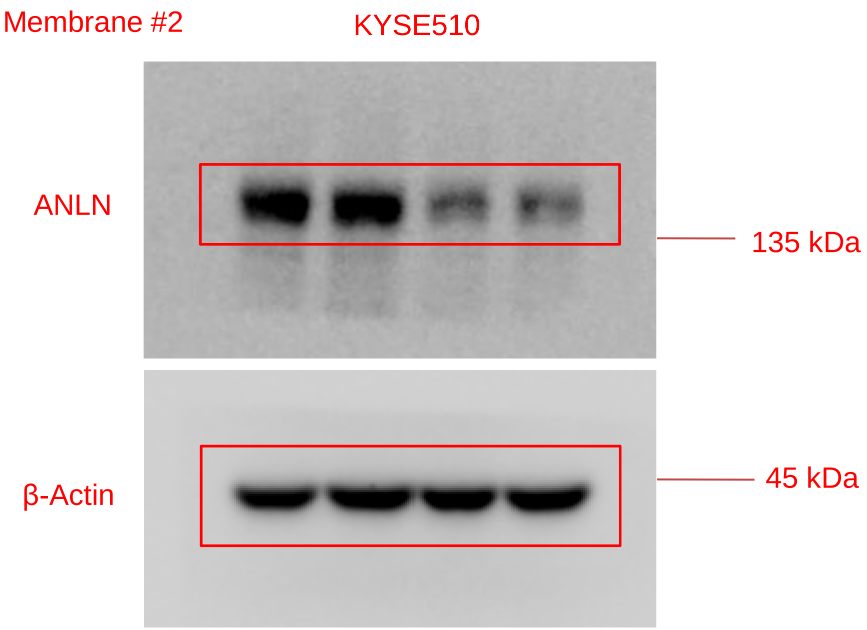
Membrane #2
KYSE510
ANLN
135 kDa
β-Actin
45 kDa

Fig.S3I

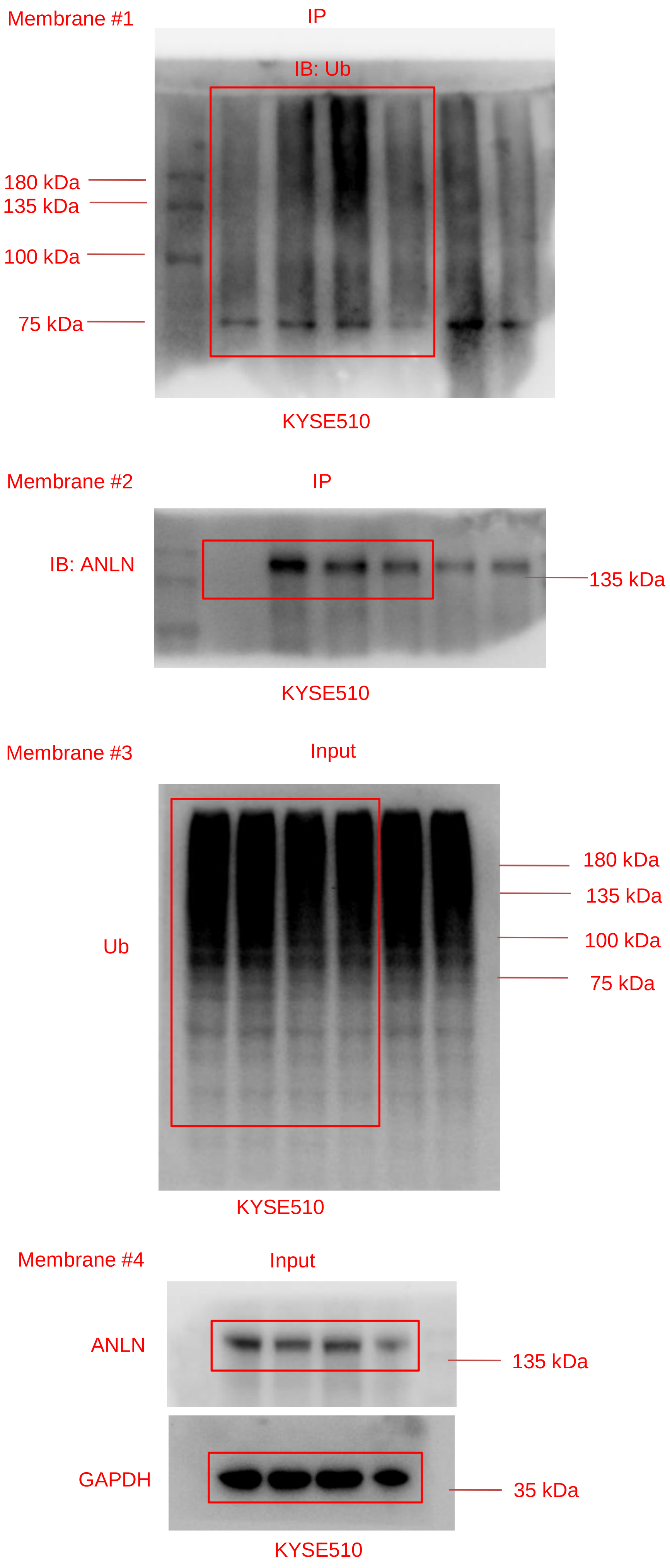

Membrane #1
IP
IB: Ub
180 kDa
135 kDa
100 kDa
75 kDa
KYSE510
Membrane #2
IP
IB: ANLN
135 kDa
KYSE510
Membrane #3
Input
Ub
180 kDa
135 kDa
100 kDa
75 kDa
KYSE510
Membrane #4
Input
ANLN
135 kDa
GAPDH
35 kDa
KYSE510

Fig.S3J

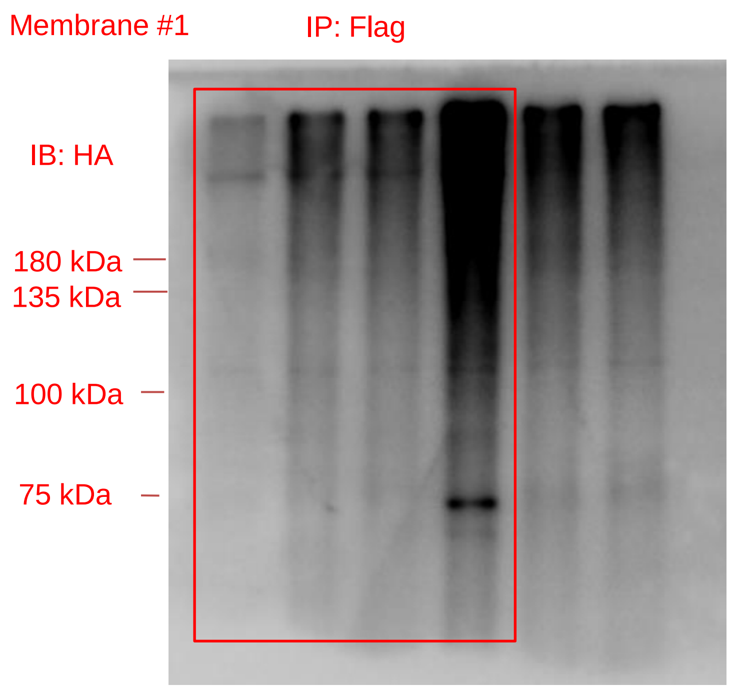
Membrane #1
IP: Flag
IB: HA
180 kDa
135 kDa
100 kDa
75 kDa

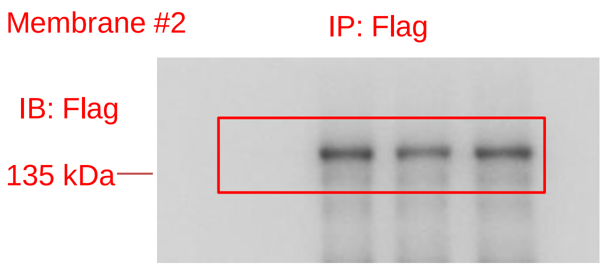
Membrane #2
IP: Flag
IB: Flag
135 kDa

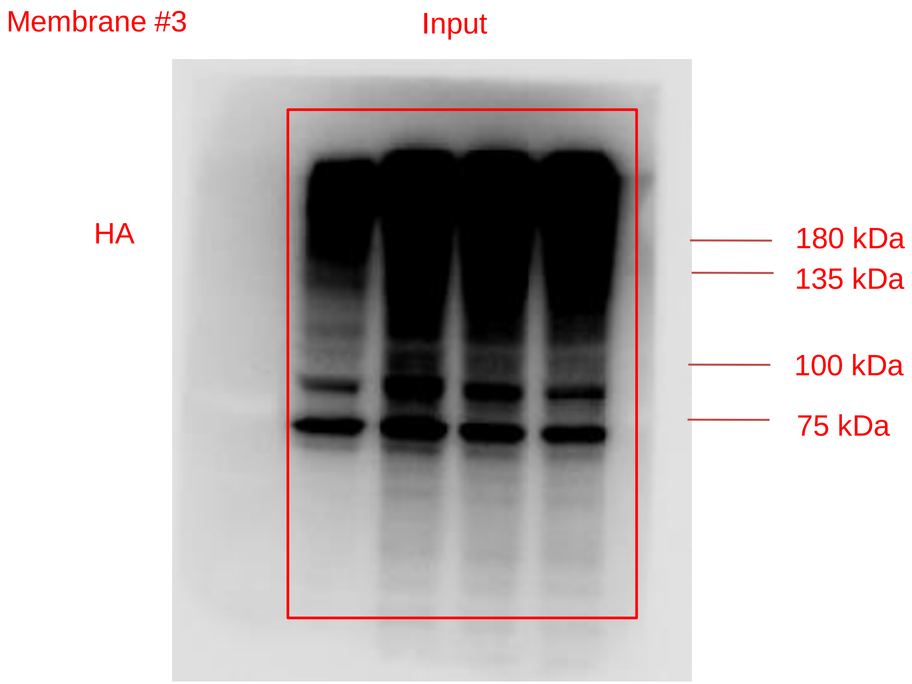
Membrane #3
Input
HA
180 kDa
135 kDa
100 kDa
75 kDa

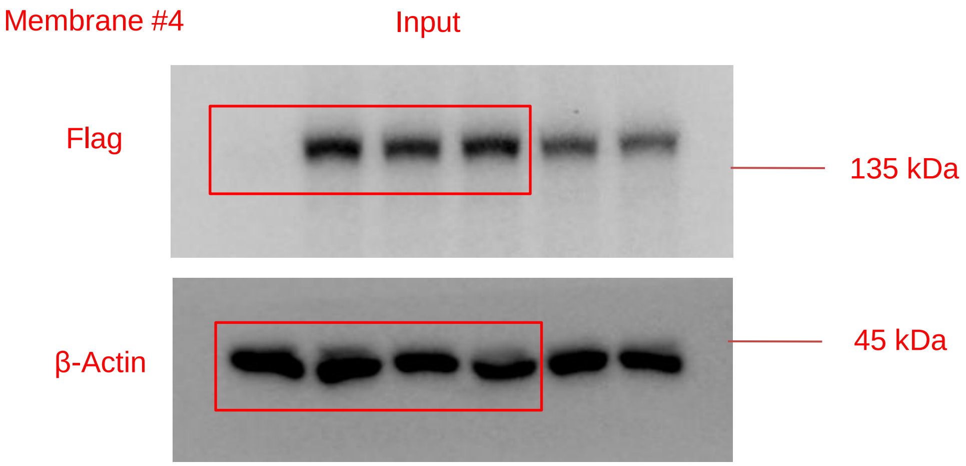
Membrane #4
Input
Flag
135 kDa
β-Actin
45 kDa

Fig.S4A

Membrane #1

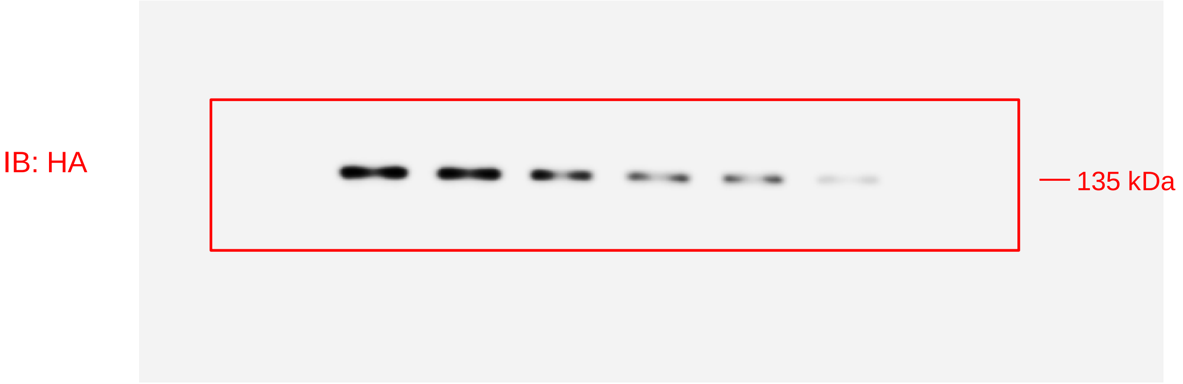


Membrane #2

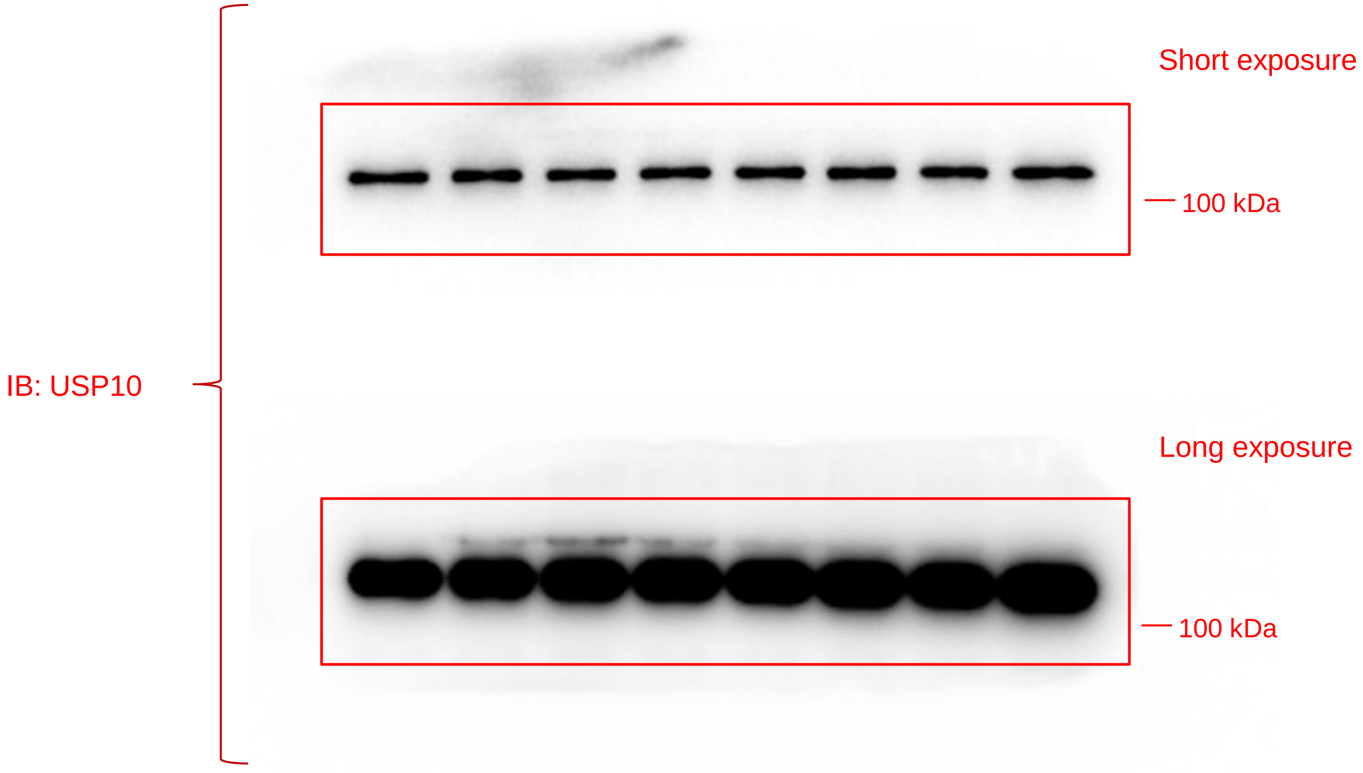

Fig.S4B

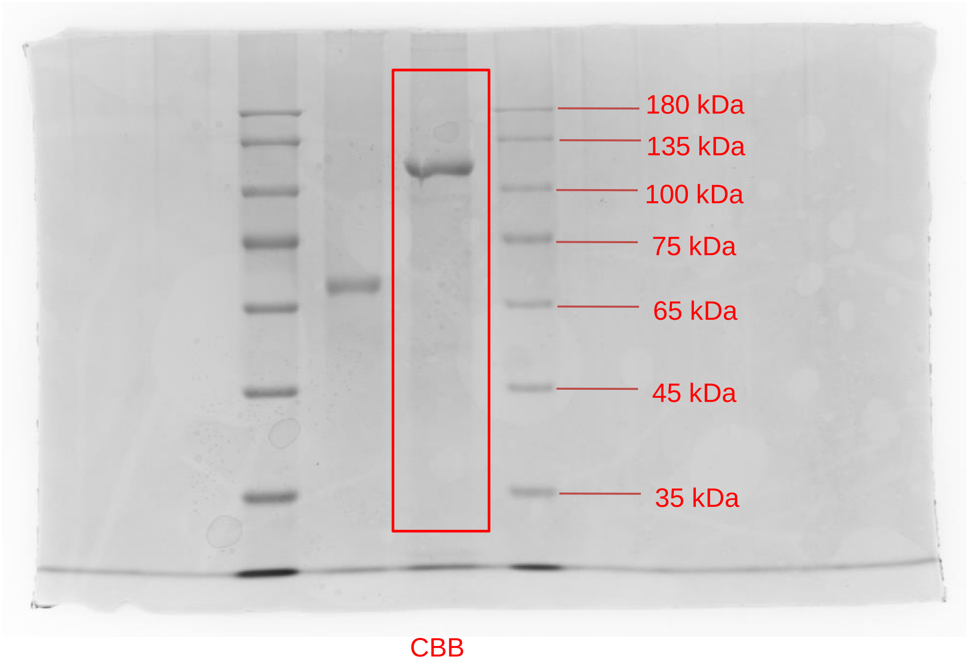


CBB

Fig.S5B

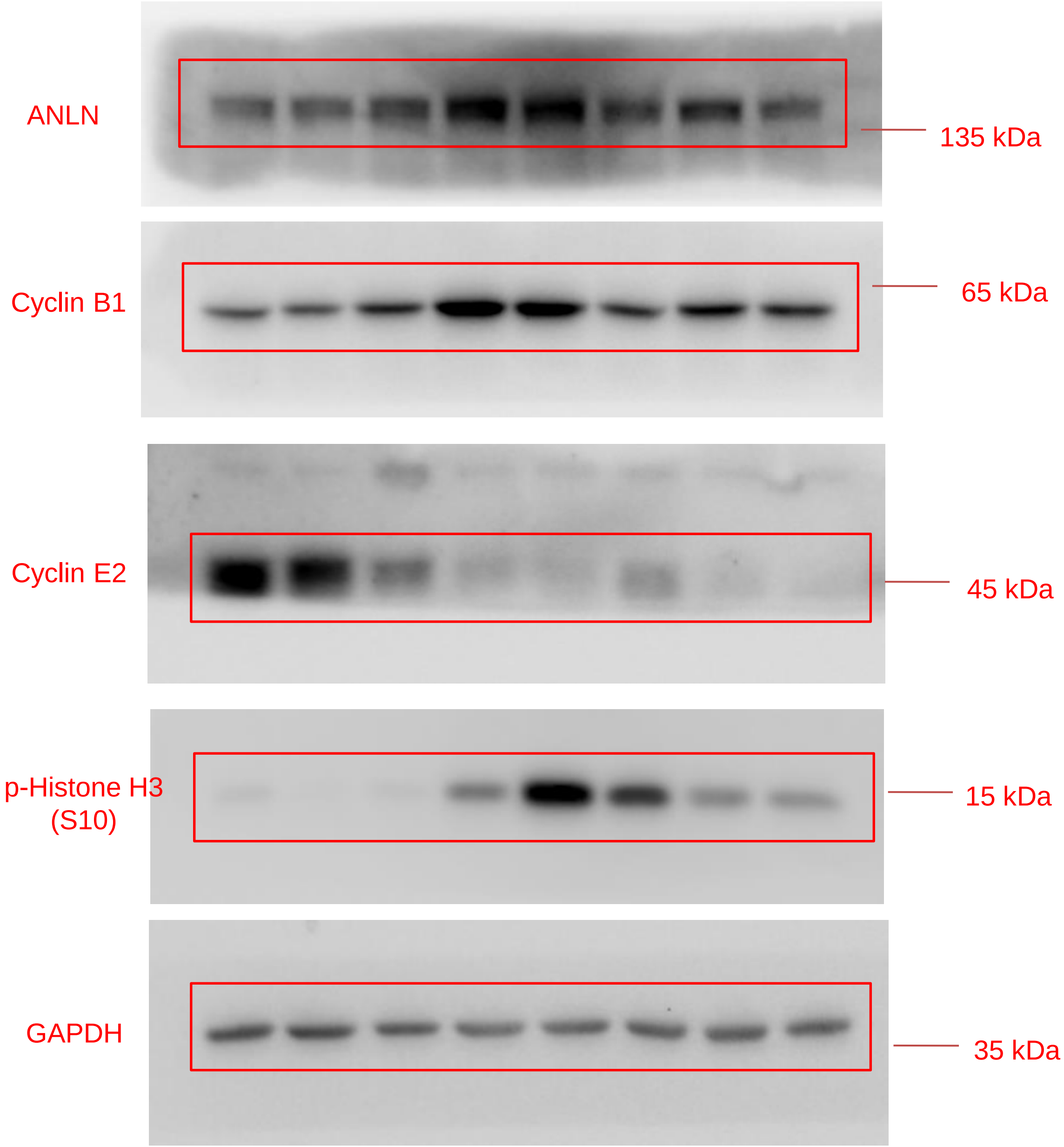

Fig.S6

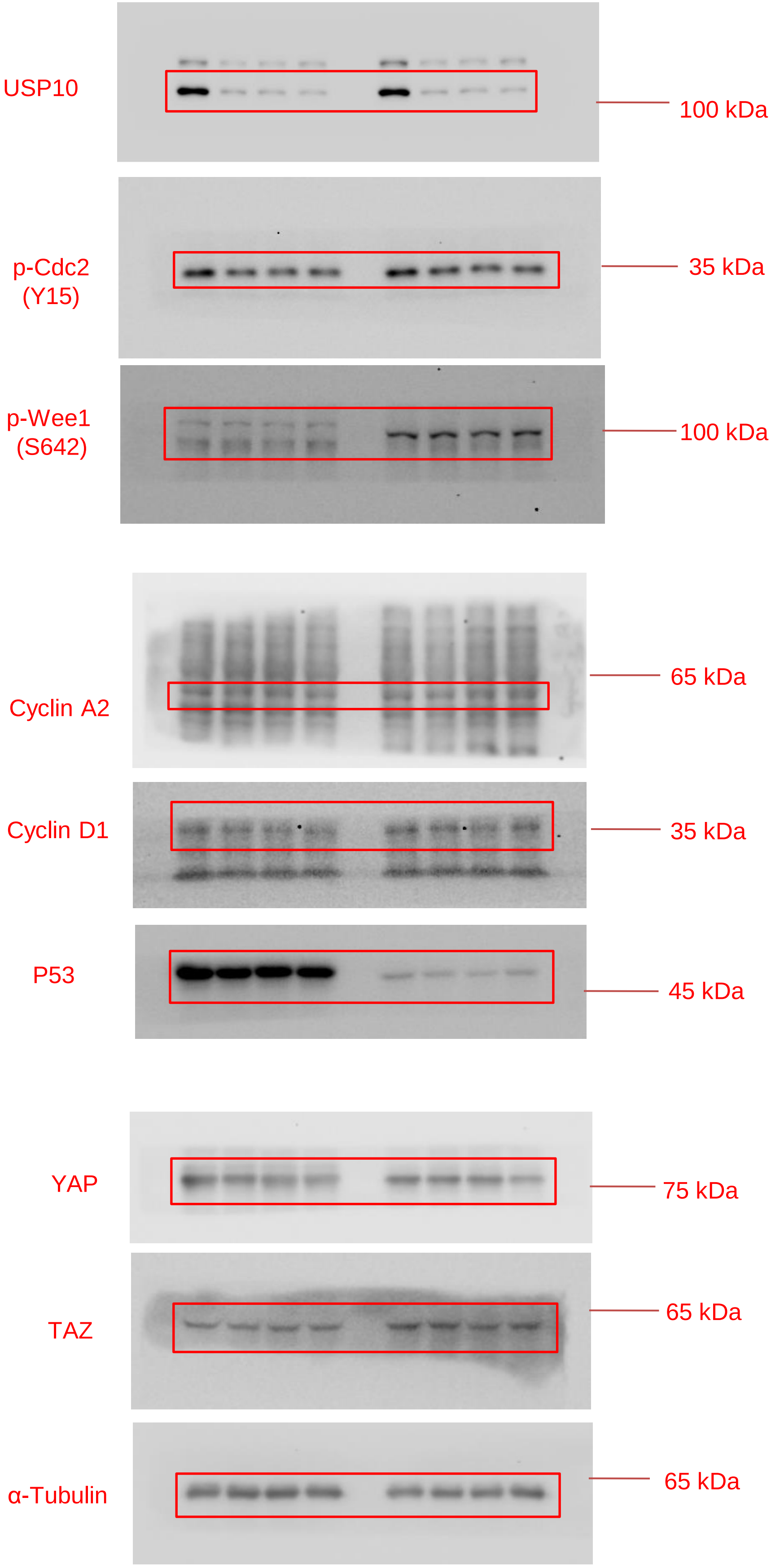
USP10
100 kDa
p-Cdc2
(Y15)
35 kDa
p-Wee1
(S642)
100 kDa
Cyclin A2
65 kDa
Cyclin D1
35 kDa
P53
45 kDa
YAP
75 kDa
TAZ
65 kDa
α-Tubulin
65 kDa
