# Supplementary material for: Targeting USP10 induces degradation of oncogenic ANLN in esophageal squamous cell carcinoma
Source: Cell Death Differ. 2022 Dec 16;30(2):527–43. doi: 10.1038/s41418-022-01104-x (PMC9950447; doi:10.1038/s41418-022-01104-x)
Supplement: Supplementary file 11 — Reproducibility Checklist form [file 41418_2022_1104_MOESM11_ESM.doc]

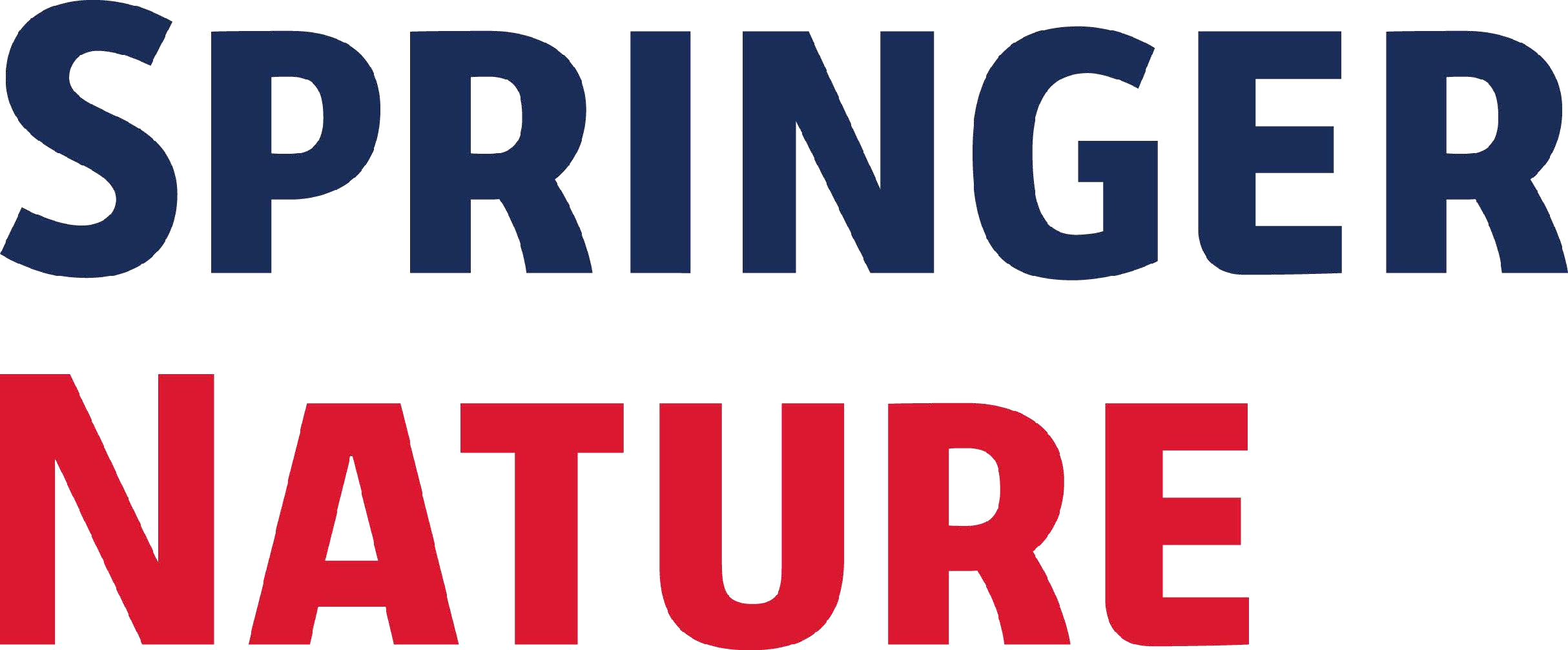
**Corresponding Author Name:** En-Min Li

**Manuscript Number:** CDD-22-1474RR

Reporting Summary

***Springer Nature wishes to improve the reproducibility of the work that we publish. This checklist is used to ensure good reporting standards and to improve the reproducibility. Please respond completely to all questions relevant to your manuscript. For more information, please read the journal’s Guide to Authors.***


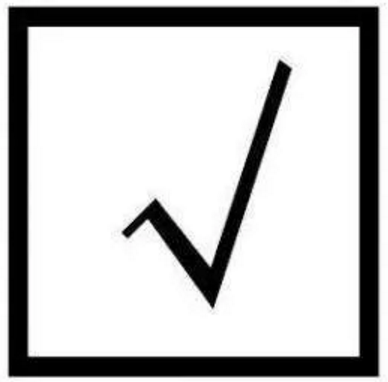
 **Check here to confirm that the following information is available in the Material & Methods section:**

- - **The exact sample size (*n)*** for each experimental group/condition, given as a number, not a range
  - **A description of the sample collection** allowing the reader to understand whether the samples represent technical or biological replicates (including how many animals, litters, culture, etc.)
  - **A statement of how many times the experiment shown was replicated in the laboratory**
  - **Definitions of statistical methods and measures**: For small sample sizes (n<5) descriptive statistics are not appropriate, instead plot individual data points

1. Very common tests, such as *t*-test, simple χ^2^ tests, Wilcoxon and Mann-Whitney tests, can be unambiguously identified by name only, but more complex techniques should be described in the methods section

o Are tests one-sided or two-sided?

o Are there adjustments for multiple comparisons? o **Statistical test results**, e.g., ***P*** **values**

o Definition of **‘center values’** as **median or mean**; o Definition of **error bars as s.d. or s.e.m. or c.i.**

***Please ensure that the answers to the following questions are reported in the manuscript itself. We encourage you to include a specific subsection in the methods section for statistics, reagents and animal models. Below, provide the page number or section and paragraph number.***

Statistics and general methods **Reported in section/paragraph or page #**

No statistical method was used to predetermine sample size. The biological experiments were performed with at least three biological replicates to allow statistical significance testing through student t-test.

1. How was the sample size chosen to ensure

adequate power to detect a pre-specified effect size? (Give section/paragraph or page #)

Sample size of animal studies is described on page 19-20, line 421-433 in the manuscript.

For animal studies, include a statement about sample size estimate even if no statistical methods were used.

Inclusion/exclusion criteria are described on page 18, lines 386-392in the manuscript.

1. Describe inclusion/exclusion criteria if samples or animals were excluded from the analysis. Were the criteria pre-established? (Give section/paragraph or page #)

The method of randomization of animals is described in the manuscript on page 20, lines 421-433.

1. If a method of randomization was used to determine how samples/animals were allocated to experimental groups and processed, describe it. (Give section/paragraph or page #)

The method of randomization of animals is described in the manuscript on page 20, lines 421-433.

For animal studies, include a statement about randomization even if no randomization was used.

*1*

Academic Journals Reporting Checklist, November 2020

The group allocation of animals is described in the manuscript on page 20, lines 421-433.

1. If the investigator was blinded to the group allocation during the experiment and/or when assessing the outcome, state the extent of blinding. (Give section/paragraph or page #)

Statement about blinding for animal studies is described on page 20, lines 421-433 in the manuscript.

For animal studies, include a statement about blinding even if no blinding was done.

Yes, the description of statistical analysis is in the manuscript on page 29-30 lines 639-646.

1. For every figure, are statistical tests justified as appropriate?

All data meet the assumptions of the tests, the description of statistical analysis is in the manuscript on page 29-30 lines 639-646.

Do the data meet the assumptions of the tests (e.g., normal distribution)?

Yes, this information is described on page 29-30 in the manuscript, lines 639-646.

Is there an estimate of variation within each group of data?

Is the variance similar between the groups that are being statistically compared? (Give section/paragraph or page #)

Yes, this information is described on page 29-30 in the manuscript, lines 639-646.

^Reagents^ **Reported in section/paragraph or page #**

The sources of all antibodies are described in the manuscript on pages 22, lines 474-486.

1. Report the source of antibodies (vendor and catalog number)

Cell lines were routinely tested for mycoplasma contamination and were authenticated by STR profiling. STR report has been uploaded to Supplementary Data 5. (Page 19, lines 418-420)

1. Identify the source of cell lines and report if they were recently authenticated (e.g., by STR profiling) and tested for mycoplasma contamination

Animal Models **Reported in section/paragraph or page #**

This information is described on page 20 in the manuscript, lines 421-433.

1. Report species, strain, sex and age of animals
2. For experiments involving live vertebrates, include a statement of compliance with ethical regulations and identify the committee(s) approving the experiments.

Animal experiments were carried out according to the program approved by Animal Research Committee of Shantou administrative center.

1. We recommend consulting the ARRIVE guidelines [(*PLoS Biol.* **8**(6), e1000412,2010)](http://www.ncbi.nlm.nih.gov/pubmed/20613859) to ensure that other relevant aspects of animal studies are adequately reported.

*2*

Academic Journals Reporting Checklist, November 2020

Human subjects **Reported in section/paragraph or page #**

This information is described on page 20 in the manuscript, lines 423-437.

1. Identify the committee(s) approving the study protocol.

This information is described on page 39 in the manuscript, lines 876-879.

1. Include a statement confirming that informed consent was obtained from all subjects.

No patient photos were published in this study.

1. For publication of patient photos, include a statement confirming that consent to publish was obtained.

There is no clinical trial in this study.

1. Report the clinical trial registration number (at [ClinicalTrials.gov](http://clinicaltrials.gov/) or equivalent).
2. For phase II and III randomized controlled trials, please refer to the [CONSORT statement](http://www.consort-statement.org/) and submit the CONSORT checklist with your submission.
3. For tumor marker prognostic studies, we recommend that you follow the [REMARK reporting guidelines.](http://www.nature.com/nrclinonc/journal/v2/n8/full/ncponc0252.html)

Data deposition **Reported in section/paragraph or page #**

All accession codes for deposited data are in lines 616-638 on pages 28-29 in the manuscript. Source data has been submitted to Supplementary data.

1. Provide accession codes for deposited data. Data deposition in a public repository is mandatory for:
   1. Protein, DNA and RNA sequences
   2. Macromolecular structures
   3. Crystallographic data for small molecules
   4. Microarray data

Deposition is strongly recommended for many other datasets for which structured public repositories exist; more details on our data policy are available in the Guide to Authors. We encourage the provision of other source data in supplementary information or in unstructured repositories such as [Figshare](http://www.figshare.com/) and [Dryad.](http://datadryad.org/) We encourage publication of Data Descriptors (see [Scientific Data)](http://www.nature.com/sdata/) to maximize data reuse.

No computer code was used in this study.

1. If computer code was used to generate results that are central to the paper’s conclusions, include a statement in the Methods section under “**Code availability”** to indicate whether and how the code can be accessed. Include version information as necessary and any restrictions on availability.

*3*

Academic Journals Reporting Checklist, November 2020
