# Supplementary material for: Targeting USP10 induces degradation of oncogenic ANLN in esophageal squamous cell carcinoma
Source: Cell Death Differ. 2022 Dec 16;30(2):527–43. doi: 10.1038/s41418-022-01104-x (PMC9950447; doi:10.1038/s41418-022-01104-x)
Supplement: Supplementary file 12 — Author contribution form [file 41418_2022_1104_MOESM12_ESM.doc]

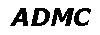
**DECLARATION OF CONTRIBUTIONS TO ARTICLE**

**
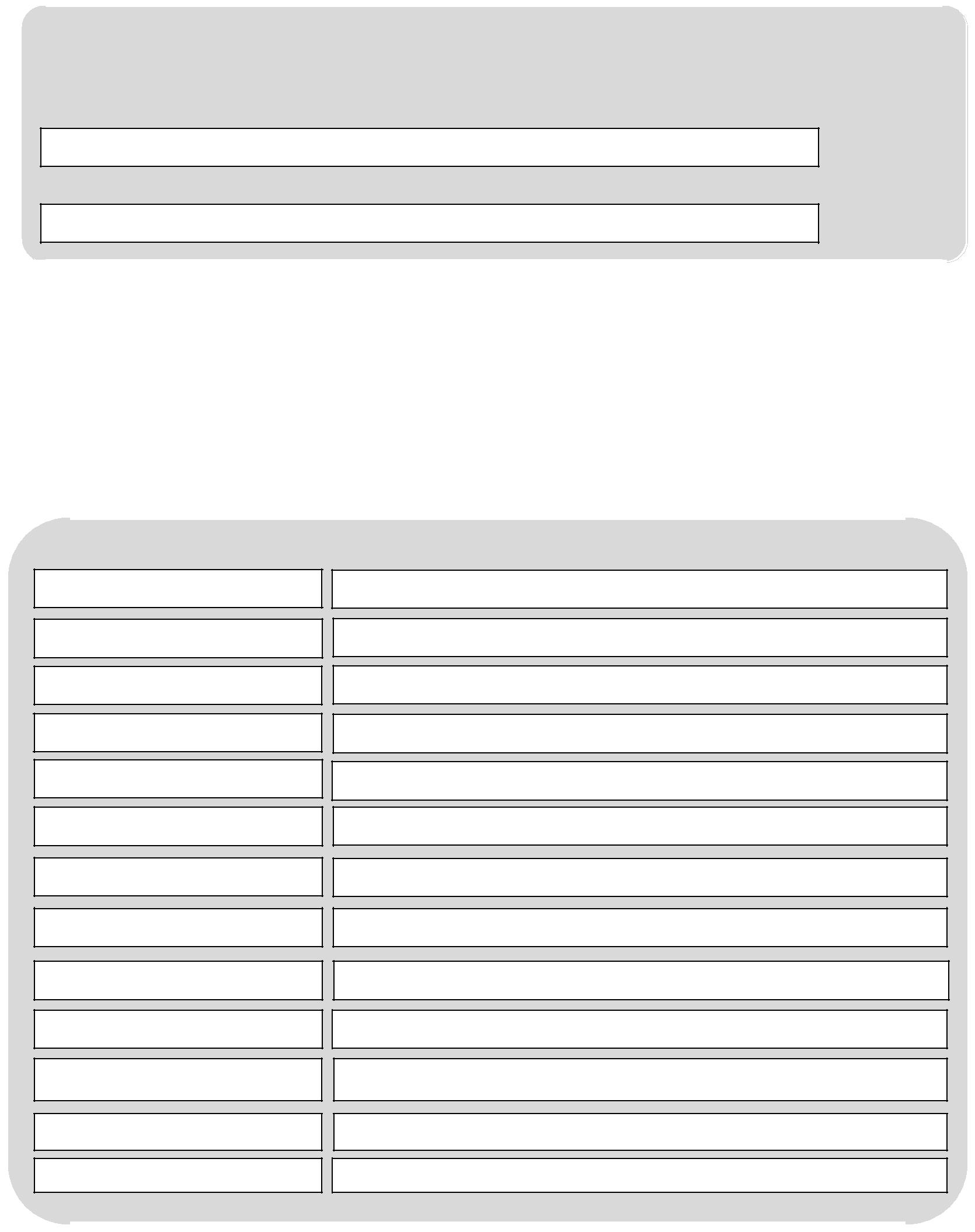
**

Manuscript Number: Journal Name:

CDD-22-1474RR

*Cell Death & Differentiation*

|  |  | *Cell Death & Differentiation* | (the ‘Journal’) |
| --- | --- | --- | --- |
|  |  |  |  |

Proposed Title of the Contribution:

Targeting USP10 induces degradation of oncogenic ANLN in esophageal squamous cell carcinoma

(the ‘Contribution)

Author(s):

Yu-Fei Cao, Lei Xie, Bei-Bei Tong, Man-Yu Chu, Wen-Qi Shi, Xiang Li, Jian-Zhong He, Shao-Hong

Wang, Zhi-Yong Wu, Dan-Xia Deng, Ya-Qi Zheng, Zhi-Mao Li, Xiu-E Xu, Lian-Di Liao, Yin-Wei Cheng, Li-Yan Li, Li-Yan Xu, En-Min Li

(the ‘Authors’)

For all *CDD* articles, each person named as an author in the published version must be able to show he or she has contributed substantially to the article.

Authorship credit should be based on 1) substantial contributions to conception and design, acquisition of data, or analysis and interpretation of data; 2) drafting the article or revising it critically for important intellectual content; and 3) final approval of the version to be published. Authors should meet conditions 1, 2 and 3.

Any person who cannot be shown to have made a substantial contribution to the article cannot be listed as an author in the final version. The name of any person who is deemed to have made a minor contribution can, however, appear in the Acknowledgments section of the article.

Please complete the table below to indicate the contributions of all named authors to the manuscript.

Author Full Name: Specification of Contribution to the Manuscript:

Yu-Fei Cao

Wrote the manuscript and performed most of the experiments

Lei Xie

Wrote the manuscript and performed most of the experiments

Bei-Bei Tong

Plasmids constructed and clinical data analyzed

Man-Yu Chu

Clinical data analyzed

Wen-Qi Shi

Plasmids constructed and bioinformatics analyses

Xiang Li

Animal experiments

Jian-Zhong He

Clinical data analyzed

Shao-Hong Wang

Provide clinical samples

Zhi-Yong Wu

Provide clinical samples

Dan-Xia Deng

Bioinformatics analyses

Ya-Qi Zheng

Clinical data analyzed

Zhi-Mao Li

Clinical data analyzed

Xiu-E Xu

Immunohistochemistry

Lian-Di Liao

Cell culture

Yin-Wei Cheng

Revised the manuscript

Li-Yan Li

Animal experiments

Li-Yan Xu

Conceived and designed the project, and revised the manuscript

En-Min Li

Conceived and designed the project, and revised the manuscript

Please complete the table below to indicate the contributions of all named authors to the figures.


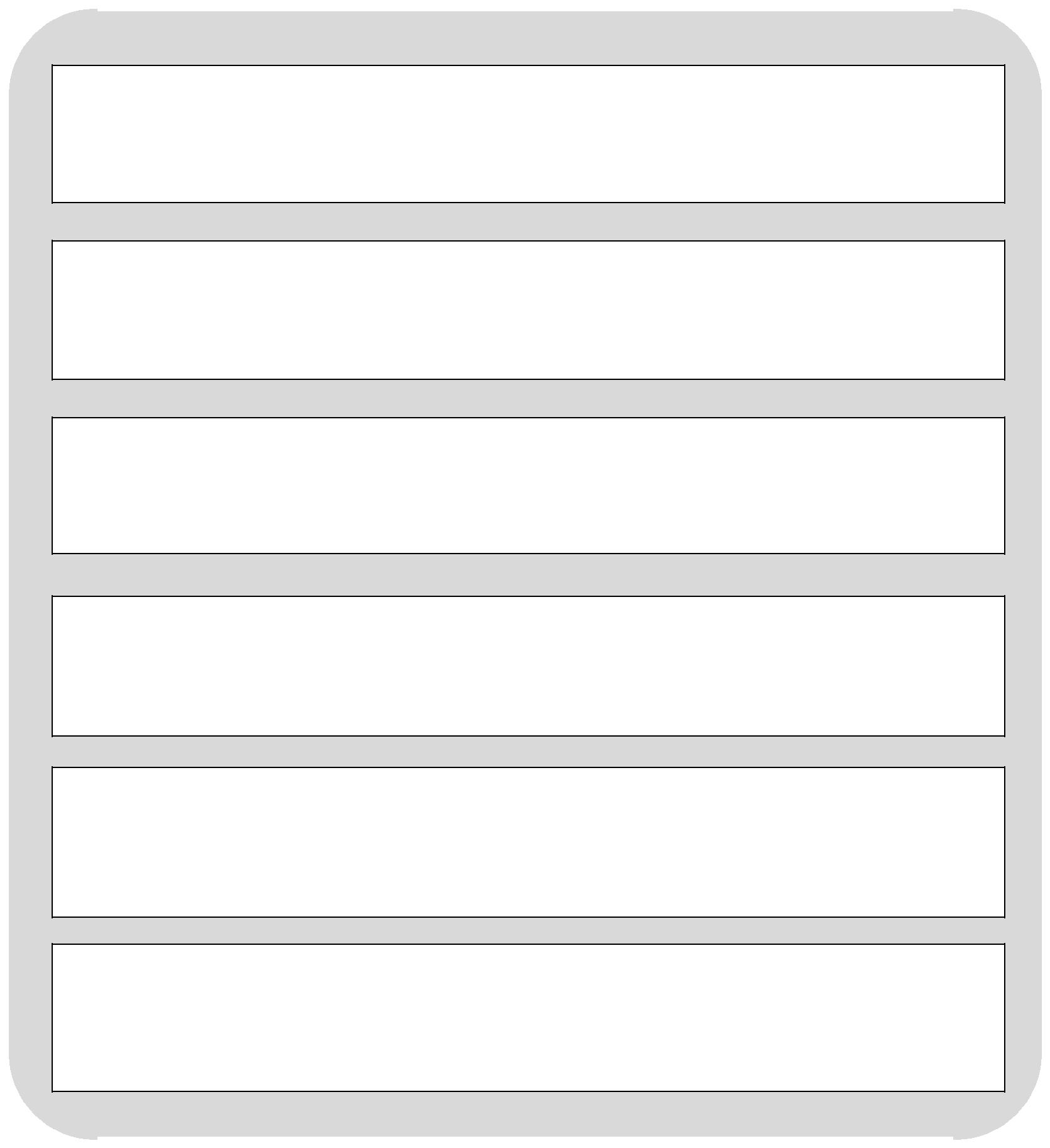


Figure 1:

In Figure 1, Shao-Hong Wang and Zhi-Yong Wu provided clinical samples. Immunohistochemistry for ANLN was performed by Xiu-E Xu. Man-Yu Chu, Ya-Qi Zheng, Zhi-Mao Li, Jian-Zhong He and Bei-Bei Tong generated the immunehistochemistry data and labelled the image (Fig.1A-C). Wen-Qi Shi constructed the plasmids. All experiments in Fig. 1D-L were performed by Yu-Fei Cao. Cells and culture medium supplied by Lian-Di Liao. Yu-Fei Cao and Lei Xie wrote the manuscript. Li-Yan Xu, En-Min Li, and Yin-Wei Cheng revised this part in manuscript, and all authors read and approved the final version.

Figure 2:

In Figure 2, Yu-Fei Cao and Lei Xie prepared protein profiling samples and prepared panel A and B. Bei-Bei Tong and Wen-Qi Shi constructed the plasmids. All experiments in Fig. 1C-N were performed by Yu-Fei Cao. Cells and culture medium supplied by Lian-Di Liao. Yu-Fei Cao and Lei Xie wrote the manuscript. Li-Yan Xu, En-Min Li, and Yin-Wei Cheng revised this part in manuscript, and all authors read and approved the final version.

Figure 3:

In Figure 3, all experiments were performed by Yu-Fei Cao. Bei-Bei Tong constructed the plasmids. Cells and culture medium supplied by Lian-Di Liao. Yu-Fei Cao and Lei Xie wrote the manuscript. Li-Yan Xu, En-Min Li, and Yin-Wei Cheng revised this part in manuscript, and all authors read and approved the final version.

Figure 4:

In Figure 4, all experiments were performed by Yu-Fei Cao. Wen-Qi Shi constructed the plasmids. Cells and culture medium supplied by Lian-Di Liao. Yu-Fei Cao and Lei Xie wrote the manuscript. Li-Yan Xu, En-Min Li, and Yin-Wei Cheng revised this part in manuscript, and all authors read and approved the final version.

Figure 5:

In Figure 5, all experiments were performed by Yu-Fei Cao. Bei-Bei Tong and Wen-Qi Shi constructed the plasmids. Cells and culture medium supplied by Lian-Di Liao. Yu-Fei Cao and Lei Xie wrote the manuscript. Li-Yan Xu, En-Min Li, and Yin-Wei Cheng revised this part in manuscript, and all authors read and approved the final version.

Figure 6:

Figure 7:

In Figure 6, Xiang Li and Li-Yan Li performed the animal experiments in panel A. For panels B-L, Bei-Bei Tong and Wen-Qi Shi constructed the plasmids, Lei Xie prepared the F806 solution and Yu-Fei Cao performed the experiments. Cells and culture medium supplied by Lian-Di Liao. Yu-Fei Cao and Lei Xie wrote the manuscript. Li-Yan Xu, En-Min Li, and Yin-Wei Cheng revised this part in manuscript, and all authors read and approved the final version.

Figure 7:

In Figure 7, Lei Xie prepared the F806 solution and Yu-Fei Cao performed the experiments. Yu-Fei Cao and Lei Xie wrote the manuscript. Li-Yan Xu, En-Min Li, and Yin-Wei Cheng revised this part in manuscript, and all authors read and approved the final version.

Figure 8:

In Figure 8, Shao-Hong Wang and Zhi-Yong Wu provided clinical samples. Immunohistochemistry for USP10 was performed by Xiu-E Xu. Ya-Qi Zheng, Zhi-Mao Li, and Jian-Zhong He generated the immunehistochemistry data and labelled the image, Bei-Bei Tong assembled the panels A, B, F and J. 124 ESCC proteomic-data in panels C, G and K was analyzed by Bei-Bei Tong and Dan-Xia Deng. Wen-Qi Shi performed bioinformatics analysis and prepared panels D, E, H, I and L. Yu-Fei Cao and Lei Xie wrote the manuscript. Li-Yan Xu, En-Min Li, and Yin-Wei Cheng revised this part in manuscript, and all authors read and approved the final version.


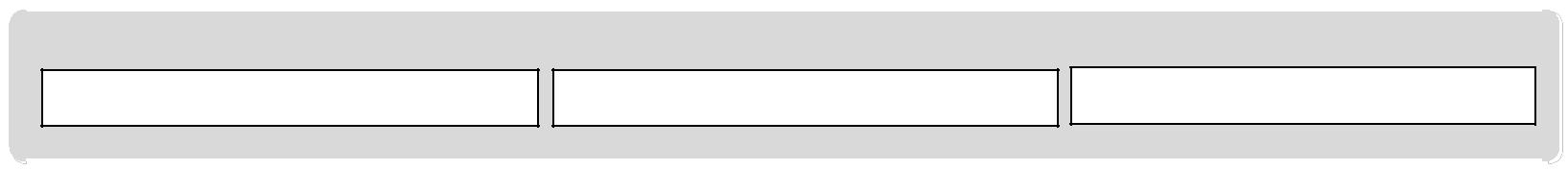


Signed for and on behalf of the Author(s): Print Name:Yu-Fei Cao, Li-Yan Xu, En-Min Li Date: *2022.11.17*
