# Supplementary material for: Targeting USP10 induces degradation of oncogenic ANLN in esophageal squamous cell carcinoma
Source: Cell Death Differ. 2022 Dec 16;30(2):527–43. doi: 10.1038/s41418-022-01104-x (PMC9950447; doi:10.1038/s41418-022-01104-x)
Supplement: Supplementary file 13 — English Editing Certificate [file 41418_2022_1104_MOESM13_ESM.doc]

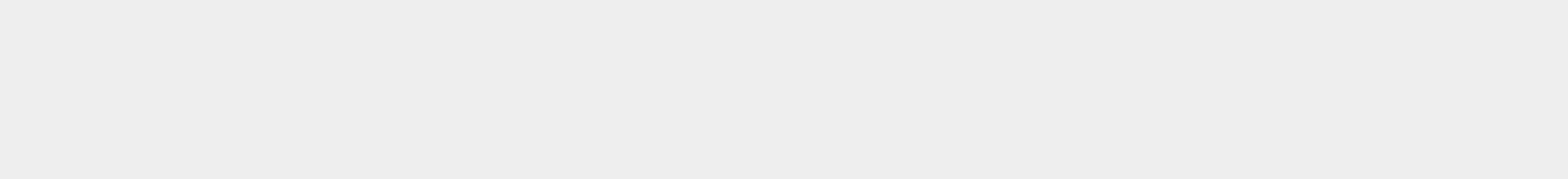
Wiley Editing Services

**ENGLISH EDITING**

**CERTIFICATE**

**
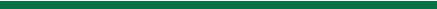
**

This document certifies that the manuscript listed below was edited for proper English language, grammar, punctuation, spelling, and overall style by one or more of the highly qualified native English speaking editors at Wiley Editing Services


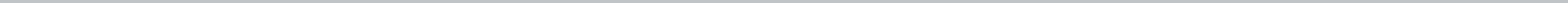


**Manuscript title**

Targeting USP10 induces degradation of oncogenic ANLN in esophageal squamous cell carcinoma

**Authors**

Yu-Fei Cao, Lei Xie, Bei-Bei Tong, Man-Yu Chu, Wen-Qi Shi, Xiang Li, Jian-Zhong He, Shao-Hong Wang, Zhi-Yong Wu, Dan-Xia Deng, Ya-Qi Zheng, Zhi-Mao Li, Xiu-E Xu, Lian-Di Liao, Yin-Wei Cheng, Li-Yan Li, Li-Yan Xu, En-Min Li

**Order No**

BICOT_1_2

**Date Issued**

September 13, 2021


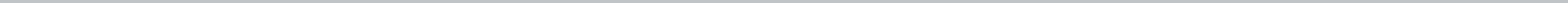

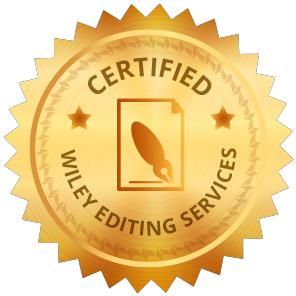


This document certifies that the manuscript listed above was edited for proper English language, grammar, punctuation, spelling, and overall style. Neither the research content nor the authors' intentions were altered in any way during the editing process. Documents receiving this certification should be English-ready for publication; however, the author has the ability to accept or reject our suggestions and changes. If you have any questions or [concerns about this document or certification, please contact help-cn@wileyeditingservices.com](mailto:help-cn@wileyeditingservices.com).


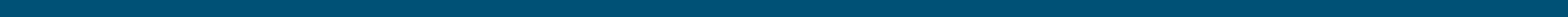


Wiley Publishing Services is a service of Wiley Publishing. Wiley's Scientific, Technical, Medical, and Scholarly (STMS) business serves the world's research and scholarly communities, and is the largest publisher for professional and scholarly societies. Wiley is committed to providing high quality services for researchers. To find out more about Wiley Editing Services, visit [http://wileyeditingservices.com](https://wileyeditingservices.com/). To learn more about our other author services provided by Wiley


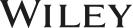


Publishing, visit <https://authorservices.wiley.com/>
